# Anti-inflammatory therapy with low-dose IL-2 in acute coronary syndromes: a randomized phase 2 trial

**DOI:** 10.1038/s41591-025-04090-y

**Published:** 2026-01-08

**Authors:** Rouchelle S. Sriranjan-Rothwell, Tian X. Zhao, Stephen P. Hoole, Simon J. Bond, Jason M. Tarkin, Jacob Brubert, Annette Hubsch, Joanna Helmy, Elaine Bumanlag-Amis, Navazh Jalaludeen, Heike Templin, Wei Jiang, Alain Tedgui, Xiaohui Zhao, Meritxell Nus, Victoria Warnes, Unni Krishnan, James W. O’Brien, Christopher Wall, James H. F. Rudd, Joseph Cheriyan, Ziad Mallat

**Affiliations:** 1 Division of Cardiology, Royal Papworth Hospital, Cambridge, UK; 2 Cambridge University Hospitals NHS Foundation Trust, Cambridge, UK; 3 Division of Cardiovascular Medicine, Department of Medicine, University of Cambridge, Cambridge, UK; 4 Cambridge Clinical Trials Unit, Cambridge University Hospitals NHS Foundation Trust, Cambridge, UK; 5 Division of Experimental Medicine and Immunotherapeutics, Department of Medicine, University of Cambridge, Cambridge, UK; 6 INSERM U970, Université Paris Cité, Paris, France; 7 Present Address: Division of Cardiovascular Medicine, Department of Medicine, University of Cambridge, Cambridge, UK; 8 National Institute for Health and Care Research (NIHR) Cambridge Clinical Research Facility, Cambridge, UK; 9 Department of Radiology (Nuclear Medicine), University of Cambridge, Cambridge, UK

**Keywords:** Randomized controlled trials, Myocardial infarction

## Abstract

Regulatory T (T_reg_) cells are powerful endogenous modulators of the immune response and their levels are reduced in patients with acute coronary syndromes (ACSs). Low-dose interleukin-2 (IL-2) has been shown to increase T_reg_ cell levels, potentially providing an immunomodulatory strategy in ACSs. The IVORY trial was a double-blind, placebo-controlled, phase 2 trial in which patients presenting with ACSs and high-sensitivity C-reactive protein levels >2 mg l^−1^ were randomized in a 1:1 ratio to receive subcutaneous low-dose IL-2 (1.5 × 10^6^ IU) or placebo for 8 weeks. [^18^F]Fluorodeoxyglucose positron emission tomography–computed tomography of the ascending aorta and carotid arteries was performed before and after treatment. Here the primary outcome was the difference in arterial inflammation in the index vessel (the vessel with the highest average maximum target-to-background ratio pre-treatment) on follow-up imaging between the two groups (placebo = 29 (female-to-male ratio (F-to-M) = 6:23); low-dose IL-2 = 31 (F-to-M = 3:28)). At the end of treatment, arterial inflammation was −0.171 (−7.7%) lower in the low-dose IL-2 group compared to the placebo group (95% confidence interval −0.308 to −0.034, *P* = 0.015). In secondary efficacy analyses, the difference in arterial inflammation between the low-dose IL-2 and placebo groups was greater (−8.3%, *P* = 0.009) in more inflamed segments and low-dose IL-2 treatment increased T_reg_ cell levels compared to placebo (*P* < 0.0001). Low-dose IL-2 treatment appeared to be safe, with no major adverse cardiovascular events at the 2-year follow-up, compared to three patients with such events in the placebo group. In conclusion, in patients with ACSs, low-dose IL-2 safely increases T_reg_ cell levels and reduces arterial inflammation. The clinical benefit of low-dose IL-2 requires validation in larger studies. ClinicalTrials.gov registration: NCT04241601.

## Abstract (web summary)

In a randomized phase 2 trial in patients with acute coronary syndrome and high levels of the inflammation biomarker C-reactive protein, treatment with low-dose interleukin-2 increased the numbers of regulatory T cells and reduced arterial inflammation, compared to placebo.

## Main

Patients who have residual inflammation following acute coronary syndromes (ACSs) are at high risk of further major adverse cardiovascular events (MACEs)^1–4^. Canakinumab and colchicine are anti-inflammatory agents that target the innate immune system and have been shown to reduce MACEs in patients with chronic coronary artery disease. In the ACS population, the evidence for colchicine reducing MACEs has been less robust, with recent data showing that it was ineffective in ACSs^5,6^. Furthermore, both agents have significant side effects. An unmet clinical need therefore exists to identify a well-tolerated drug that regulates the immune system and effectively reduces residual inflammation in ACSs^7^.

Regulatory T (T_reg_) cells are powerful endogenous immune modulators. They exert their effects through diverse mechanisms, including effector T (T_eff_) cell immunosuppression through direct cell contact, deprivation of survival factors, promotion of tolerogenic dendritic cells and production of immunosuppressive cytokines (for example, interleukin (IL)-10 and transforming growth factor-β), among others^8,9^. T_reg_ cells are implicated in tissue homeostasis, healing and repair. Studies have demonstrated that T_reg_ cell numbers are reduced and their function impaired in ACSs^10,11^. In pre-clinical studies, increasing T_reg_ cells led to reduced atherosclerosis^12–15^, smaller myocardial infarct size and improved myocardial function^16,17^. Therefore, increasing endogenous T_reg_ cells could provide a new targeted anti-inflammatory and tissue repair strategy in ACSs.

IL-2 at high doses activates T_eff_ cells and is currently licensed for the treatment of metastatic renal cell carcinoma and metastatic melanoma. As T_reg_ cells are enriched for the high-affinity, trimeric IL-2 receptor compared to T_eff_ cells, they can be selectively increased with low doses of IL-2^18^. We previously demonstrated that low-dose IL-2 was safe and led to a selective statistically significant increase in T_reg_ cells in patients with both ACS and stable ischemic heart disease^19^. In the trial of low-dose IL-2 for the reduction of vascular inflammation in ACSs (IVORY), we hypothesized that low-dose IL-2 would reduce arterial inflammation compared to placebo in patients presenting with ACSs who have residual inflammation detected by high-sensitivity C-reactive protein (hsCRP) levels >2 mg l^−1^. We used [^18^F]fluorodeoxyglucose positron emission tomography–computed tomography ([^18^F]FDG PET–CT) to quantify arterial inflammation. [^18^F]FDG PET–CT is a validated and highly reproducible technique, which is the current gold standard for quantifying arterial inflammation noninvasively^20–23^. Furthermore, vascular [^18^F]FDG uptake has also been related to future cardiovascular risk^24–27^. The IVORY-clinical outcomes and follow-up (IVORY-FINALE) study aims to report cardiovascular clinical outcomes for these patients for up to 5 years.

## Results

### Patients

**Fig. 1:**
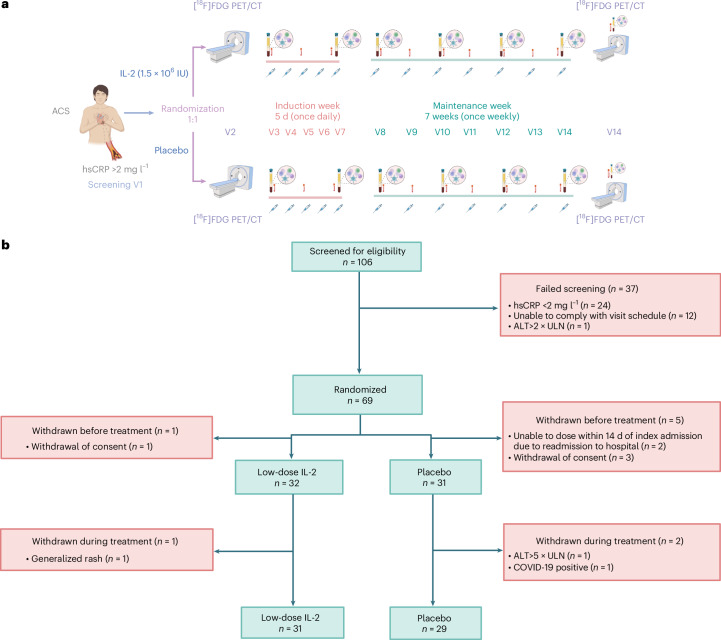
IVORY trial design and participant flow. **a**, Design of the IVORY trial. Patients presenting with ACSs and hsCRP >2mg l^−1^ who passed screening were randomized to either subcutaneously administered 1.5 × 10^6^ IU of low-dose IL-2 or placebo (volume equivalent of 5% dextrose). An [^18^F]FDG PET–CT scan of the ascending aorta and carotids was carried out before the start of treatment. The first dose was administered within 14 days of admission of the patient to hospital. Patients received treatment once daily for 5 days in the induction phase, followed by once-weekly injections for 7 weeks in the maintenance phase. The dosing visits were identical in the maintenance phase, except that complete blood counts, liver and renal function tests were checked before administration of IL-2 or placebo. FACS for T cell subsets was carried out within 4 h of the blood draw. Blood samples for immunological analyses and analyses of peripheral blood mononuclear cells were collected and stored at visit 3 (V3) before treatment administration, as well as at V7, V8, V10, V12, V14 and V15. V2–V16 were carried out on an outpatient basis. V16 was the last trial encounter and was performed remotely unless an in-person visit was required for safety reasons. **b**, Participant flow in the IVORY trial. ALT, alanine transaminase, COVID-19, coronavirus disease 2019; ULN, upper limit of normal. Created with BioRender.com.

Patients were recruited to the IVORY trial (Fig. 1a) between August 2020 and November 2022; the last trial visit took place in January 2023. Patients presenting with ACSs (*n* = 106) were screened for eligibility during their index admission (Fig. 1b). Patients with unstable angina, non-ST-elevation myocardial infarction (NSTEMI) and ST-elevation myocardial infarction (STEMI) were eligible for recruitment into the IVORY trial. Most patients failed screening due to hsCRP levels <2 mg l^−1^, accounting for 65% of screen failures. Sixty-nine patients underwent randomization to either 1.5 × 10^6^ IU of IL-2 or placebo; of these individuals, 63 received at least 1 dose of the drug (Fig. 1b). The reasons for drop-outs at this stage were withdrawal of consent (*n* = 4) and inability to dose within 14 days of the index admission to hospital (*n* = 2). Of the patients dosed, 32 received subcutaneously administered, low-dose IL-2 and 31 received placebo (subcutaneously administered 5% glucose). The median time from the date of index admission to the first dose was 11 days (interquartile range (IQR) 9–12 days). One patient from the low-dose IL-2 arm and two from the placebo arm were withdrawn from the study for safety reasons, discussed in more detail below. A total of 60 patients completed the IVORY trial (low-dose IL-2 = 31; placebo = 29).

Baseline demographic and clinical characteristics were well balanced between the groups (Table 1). These patients had a median age of 55 years with a mean body mass index (BMI) of 29.7 kg m^−^^2^ and had multiple cardiovascular risk factors. As recruitment was restricted to postmenopausal or perimenopausal women due to the paucity of safety data in women of childbearing potential, there were fewer women in this trial than in a nonselective ACS population.

**Table 1:** Demographic and clinical characteristics of the patients

Characteristic	Placebo (*n* = 31)	Low-dose IL-2 (*n* = 32)
Median age (range), years	55 (39–75)	56.5 (34–73)
Sex, no. (%)		
Female	7 (23)	3 (9)
Male	24 (77)	29 (91)
BMI, kg m^−2^ (mean ± s.d.)	29.6 ± 5.8	29.8 ± 5.7
Ethnicity, no. (%)		
White	30 (97)	27 (84)
Asian	1 (3)	3 (9)
Black	0 (0)	0 (0)
Mixed	0 (0)	1 (3)
Other	0 (0)	1 (3)
Diagnosis at admission, no. (%)		
Unstable angina	0 (0)	2 (6)
NSTEMI	15 (48)	11(34)
STEMI	16 (52)	19 (59)
Median hsCRP level, mg l^−1^ (interquartile range)	10.1 (5.0–29.1)	8.4 (4.4–19.2)
Median high-sensitivity troponin I at the first dosing visit (interquartile range), ng l^−1^ (range) (reference range 0–54 ng l^−1^)	59 (20–346)	67 (28–305)
Median low-density lipoprotein–cholesterol level, mmol l^−1^ (interquartile range)	1.8 (1.40–2.70)	2.10 (1.68–2.40)
GRACE score at admission, mean ± s.d.	96.1 ± 19	95.4 ± 25
Cardiovascular history, no. (%)		
Hypertension	29 (94)	32 (100)
Hypercholesterolemia	29 (94)	32 (100)
Previous angioplasty	2 (6)	5 (16)
Stroke	0 (0)	2 (6)
Type 2 diabetes	0 (0)	2 (6)
Medication history, no. (%)		
Aspirin	31 (100)	32 (100)
Clopidogrel or ticagrelor	31 (100)	32 (100)
Statin	31 (100)	31 (97)
β-Blocker	30 (97)	28 (88)
Renin–angiotensin inhibitor	30 (97)	29 (91)
Smoking status, no. (%)		
Never smoked	12 (39)	12 (38)
Current smoker	12 (39)	10 (31)
Previous smoker	7 (22)	10 (31)
Baseline arterial inflammation, mean TBR_max_	2.39 ± 0.28	2.26 ± 0.27
Treatment given for index ACS admission, no. (%)		
Percutaneous coronary angioplasty	31 (100)	30 (94)
Medical management	0 (0)	2 (6)
Culprit vessel leading to index ACS admission, no. of patients (%)		
Left anterior descending artery	16 (52)	17 (53)
Left circumflex artery	6 (19)	6 (19)
Right coronary artery	9 (29)	9 (28)

No. (%) is the number of participants (percentage of participants).

GRACE, global registry of acute coronary events.

All patients were on dual antiplatelet therapy and 62 (98%) patients were on high-dose statin therapy at baseline. All patients had an invasive coronary angiogram and a culprit lesion defined on angiography; 61 (97%) patients had intervention with drug-eluting stents and 2 (3%) were medically managed. The median hsCRP for the trial population at screening was 9.55 mg l^−1^ (IQR 4.82–21.80 mg l^−1^). There were no significant differences between the groups at baseline in hsCRP and arterial inflammation as measured by the mean of the maximum target-to-background ratio (mean TBR_max_) in the index vessel.

### Efficacy of low-dose IL-2 in ACS

#### Primary efficacy analyses

**Fig. 2:**
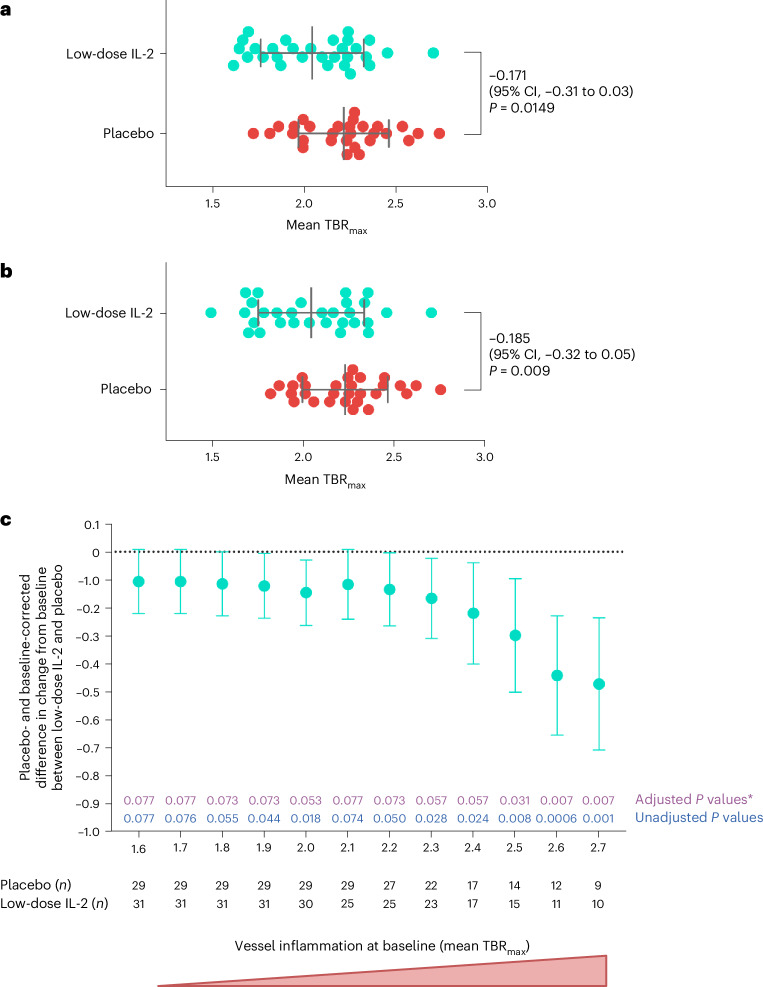
Changes in arterial inflammation. **a**, Arterial inflammation in the index vessel (whole vessel, all segments) in patients who received low-dose IL-2 (*n* = 31) and placebo (*n* = 29), at the end of the 8-week treatment period. Arterial inflammation was −0.171 (7.7%) lower in the low-dose IL-2 group compared to placebo (*P* = 0.0149). The dot represents individual patient values, the longest vertical line the mean for each group and the error bars the s.d. **b**, Arterial inflammation at the end of the 8-week treatment period in segments of the index vessel predefined as active or inflamed (TBR_max_ > 2) at baseline in patients who received low-dose IL-2 (*n* = 30) or placebo (*n* = 29). One patient in the IL-2 group did not have inflamed segments in the index vessel at baseline. Arterial inflammation in the low-dose IL-2 group was −0.185 (8.3%) lower compared to placebo, at the end of the treatment period (*P* = 0.009). The dot represents individual patient values, the longest vertical line the mean for each group and the error bars the s.d. No adjustments for multiple testing were made in these analyses. **c**, Placebo-corrected and baseline-corrected treatment effect of low-dose IL-2 at various baseline levels of inflammation. The dots represent the estimate of the difference between low-dose IL-2 and placebo and the error bars are the 95% CIs for this estimate. ^*^P values were adjusted for multiple testing. Linear regression models were used in reporting the estimated coefficients and their s.e. values. Some models used treatment only as a predictor, which is identical to an unpaired Student’s *t*-test (**a** and **b**); others also adjusted for baseline value which is identical to a baseline-corrected analysis of covariance (**c**). CIs and two-sided *P* values were provided based on the estimates following a *t*-distribution.

In each arm, a significant reduction in arterial inflammation from baseline to follow-up was observed (Extended Data Table 1a), with this difference being greater in the low-dose IL-2 arm (−0.216, 95% confidence interval (CI) −0.31 to −0.12, *P* < 0.001) than in placebo (−0.175, 95% CI −0.27 to −0.08, *P* < 0.001). The sample size for the IVORY trial was based on an absolute difference of 0.2 in mean TBR_max_ of the index vessel between placebo and low-dose IL-2 at the end of the treatment period. This is equivalent to the effect observed with high-dose statin therapy (when compared to low-dose statin therapy), which reduces MACEs in ACSs^28–30^. As such, the primary outcome of the trial was the difference between the groups in arterial inflammation at the end of treatment as measured by [^18^F]FDG PET–CT. At the end of 8 weeks of treatment, the mean TBR_max_ in the index vessel was lower (−0.171, 95% CI −0.308 to −0.034, *P* = 0.015) in patients treated with low-dose IL-2 (2.04 ± 0.28) compared to placebo (2.22 ± 0.25) (Fig. 2a). In the index vessel, arterial inflammation was 7.7% lower in patients treated with low-dose IL-2 compared to placebo. In addition, the difference in change from baseline between the groups (Extended Data Table 1a) showed a trend toward a reduction in arterial inflammation in the low-dose IL-2 group compared to placebo, but did not reach statistical significance (−0.103, 95% CI −0.22 to 0.02, *P* = 0.086).

The post-hoc analysis demonstrated that there was no evidence that the treatment effect was significantly different between arterial regions (Extended Data Table 1b).

#### Secondary efficacy analyses

##### Arterial inflammation

**Extended Data Fig. 1:**
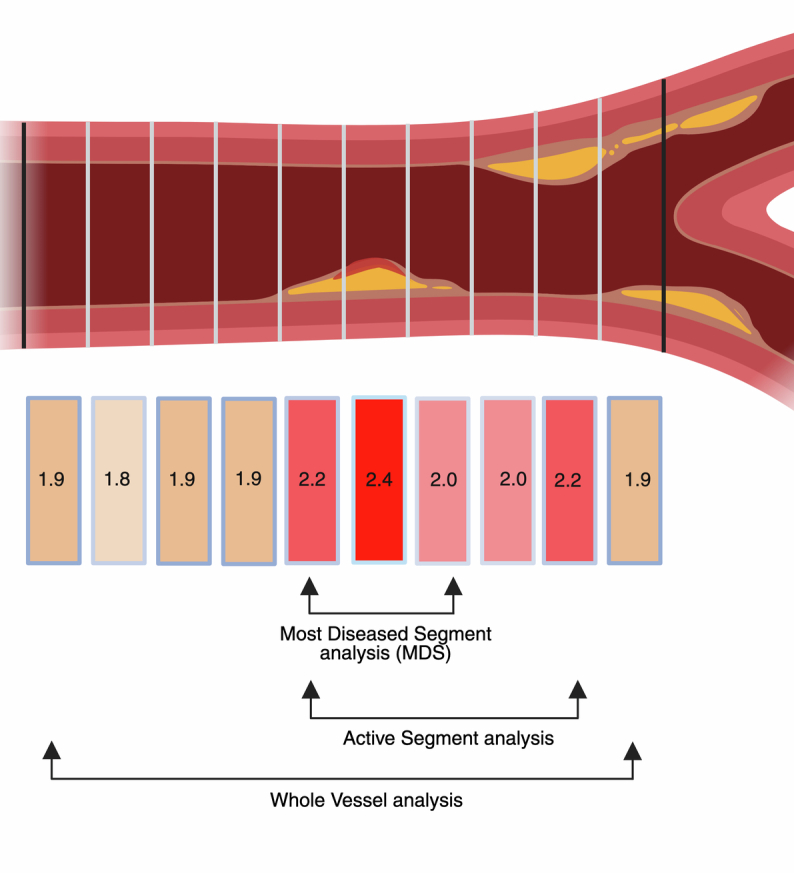
Methods of analysis of arterial inflammation using ^18^F-FDG PET imaging. In the most disease segment analysis (MDS) the most inflamed part of the index vessel is analyzed. In the active segment (AS) analysis, all parts of the vessel that are inflamed are analyzed. The whole vessel analysis looks at the ^18^F-FDG PET uptake entire length of the vessel. Created with BioRender.com.

When areas of the index vessel with a TBR_max_ > 2 at baseline (active segments) were analyzed (Extended Data Fig. 1) at the end of treatment, the difference between the two groups was greater, with an 8.3% difference in arterial inflammation between the low-dose IL-2 group and the placebo group (−0.185, 95% CI −0.323 to −0.048, *P* = 0.009) (Fig. 2b). When the most diseased (inflamed) segment of the index vessel was compared between groups, the difference in arterial inflammation was even more pronounced, with the arterial inflammation in the low-dose IL-2 group being 9.3% (−0.213, 95% CI −0.372 to 0.054, *P* = 0.010) lower than the placebo group. Changes in arterial inflammation from baseline were also compared between the groups for the active segments and most diseased segments of the index vessel (Extended Data Table 1a). Corrected for baseline and placebo, a statistically significant difference between the groups was observed for the active and most diseased segments of the index vessel (active segment analysis = 6%, *P* = 0.018; most diseased segment analysis = 7.3%, *P* = 0.013).

The treatment effect of low-dose IL-2 corrected for placebo was also studied for various thresholds of baseline inflammation. Overall, when corrected for baseline and placebo, the higher the arterial inflammation at baseline, the greater the anti-inflammatory effect observed with low-dose IL-2 (Fig. 2c).

##### Effects on circulating immune cells

**Fig. 3:**
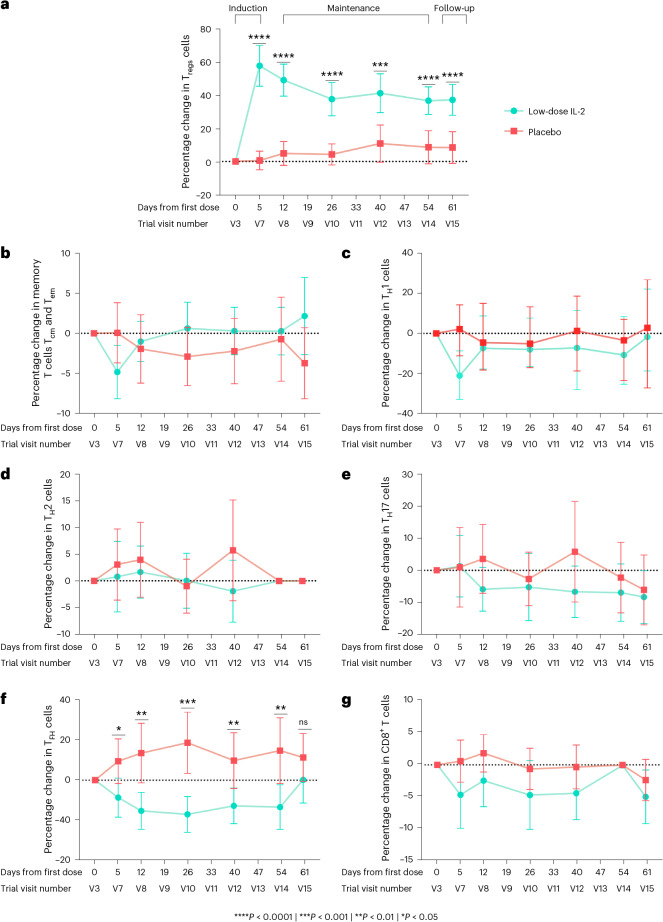
Changes in circulating T cells. **a**, Percentage changes from baseline in T_reg_ cells (measured as a percentage of CD4^+^ T cells) in the low-dose IL-2 and placebo groups at the indicated treatment visits. Values were significantly different between the two groups at all visits apart from baseline. **b**–**g**, Percentage changes from baseline for circulating T_CM_ cells and T_EM_ cells (**b**), T_H_1 cells (**c**), T_H_2 cells (**d**), T_H_17 cells (**e**), T_FH_ cells (**f**) and CD8^+^ T cells (**g**). In **b**–**g**, cell populations were measured as a percentage of CD4^+^ T cells, except for CD8^+^ cells, which were measured as a percentage of T cells, in the low-dose IL-2 and placebo groups at the indicated treatment visits. For all panels, V3 was the start of induction dosing, V7 was the last day of the induction week, V8–V14 were for maintenance dosing (seven weekly dosing visits) and V15 was a follow-up visit, carried out approximately 1 week after the last dosing visit. Immunophenotyping was done before dosing at each visit, so the results shown represent trough levels for the measured cells. In each panel (apart from **c**), the points correspond to means and the error bars indicate 95% CIs. In **c**, the points correspond to the median and the error bars indicate the IQR. The *P* values are for statistical comparisons between placebo and low-dose IL-2 for the visit. For all panels, linear regression models were used for reporting the estimated coefficients and their s.e. values. CIs and two-sided *P* values were provided based on the estimates following a *t*-distribution. For **c**, the Mann–Whitney *U*-test was used. No adjustments for multiple testing were made for these analyses. For all panels, low-dose IL-2, *n* = 30 and placebo, *n* = 29. For **a**, the exact statistically significant *P* values for differences between the groups are as follows: V7: *P* = 1.1 × 10^−10^; V8: *P* = 6.3 × 10^−10^; V10: *P* = 6.2 × 10^−7^; V12: *P* = 3.1 × 10^−4^; V14: *P* = 4.5 × 10^−5^; V15: *P* = 4.6 × 10^−5^. For **f**, V7: *P* = 0.015; V8: *P* = 0.0014; V10: *P* = 0.0002; V12: *P* = 0.007; and V14: *P* = 0.005.

**Extended Data Fig. 2:**
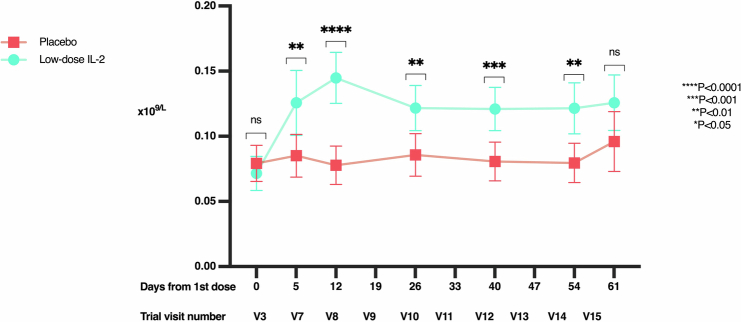
Absolute Treg numbers by trial visit. Visit 3 (V3) was the start of induction dosing. Visit (V7) was the last day of the induction week. Visits V8 to V14 were visits done for maintenance dosing (7 weekly dosing visits). Immunophenotyping was done prior to dosing at each visit, therefore these numbers represent trough levels for measured cells. V15 was a follow-up visit, carried out approximately 1 week after the last dosing visit. The points correspond to means and the error bars indicate 95% confidence intervals. Placebo n = 29, Low-dose IL-2 n = 30. Linear regression models were used for reporting the estimated coefficients and their standard errors. Confidence intervals and 2-sided P values were provided based on the estimates following a t-distribution. P values are for statistical comparison between placebo and low-dose IL-2 for the visit. The exact statistically significant P values for differences between the groups are as follows. V7 P = 0.007, V8 P = 7.4 ×10^−7^, V10 P = 0.003, V12 P = 0.0005, V14 P = 0.001.

At all treatment visits after baseline, there was a significant increase from baseline in T_reg_ cells in the low-dose IL-2 group compared to placebo (Fig. 3a and Extended Data Fig. 2). After 4 days of treatment in the induction phase, a 57% increase in T_reg_ cells from baseline was observed compared to an 0.6% increase in placebo (Δ = 56.73%, 95% CI 43.42–70.04). At the end of the maintenance phase (V8–V14), T_reg_ cells were 34% (95% CI 27.58–40.45) higher in the low-dose IL-2 arm compared to placebo.

With respect to the effector arm of the CD4^+^ T cell response (Fig. 3b–f), no differences were observed between the two treatment groups for percentage change from baseline in CD4^+^ T central memory (T_CM_) and T effector memory (T_EM_) (Fig. 3b), T helper type 2 (T_H_2) (Fig. 3d) and T helper type 17 (T_H_17) cells (Fig. 3e). T helper type 1 (T_H_1) cells remained unchanged in the placebo arm, whereas, in the low-dose IL-2, there was a decrease (Fig. 3c) pooled across all visits (*P* = 0.016). T follicular helper (T_FH_) cells increased from baseline in the placebo arm whereas they decreased in the low-dose IL-2 arm (Fig. 3f). The difference between the groups, pooled across all visits, was statistically significant (Δ = −24%, 95% C1 −30.97 to −17.45).

The percentage of cytotoxic CD8^+^ T cells was reduced from baseline in the low-dose IL-2 arm compared to the placebo arm (Fig. 3g). The difference between the groups was again statistically significant when pooled across all visits (Δ = −3.39%, 95% CI −5.408 to −1.376).

### Exploratory analyses

There were no differences between the groups for circulating neutrophils, basophils or monocytes (Extended Data Table 2a). There was an increase in circulating eosinophils in the low-dose IL-2 group compared to placebo at the end of treatment and when pooled across all visits (*P* < 0.001).

**Extended Data Fig. 3:**
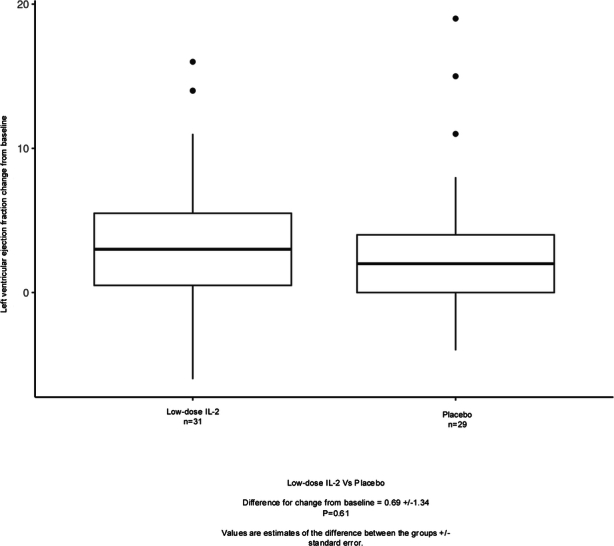
Changes in left ventricular ejection fraction (%) as measured by transthoracic echocardiography. The mid-line is the median, and the box limits are the 25^th^ and 75^th^ percentile. The upper whisker extends to the largest value no further than 1.5xIQR from the 75^th^ percentile, and similarly the lower whisker extends to the smallest value at most 1.5 x IQR of the 25th percentile. Data beyond the end of the whiskers are plotted individually. Placebo n = 29, Low-dose IL-2 n = 31. Linear regression models were used for reporting the estimated coefficients and their standard errors. Confidence intervals and 2-sided p-values were provided based on the estimates following a t-distribution.

There were no differences between the groups in hsCRP levels (Extended Data Table 2b), total cholesterol, low-density lipoprotein–cholesterol and triglycerides (Extended Data Table 2c), and left ventricular ejection fraction assessed by echocardiography (Extended Data Fig. 3), at the end of the treatment period.

### Safety

Assessing the safety of low-dose IL-2 was a secondary outcome of the IVORY trial. Table 2 summarizes adverse events (AEs) observed in all patients who received at least one dose of treatment. One serious AE (SAE) occurred in a patient in the placebo arm who was hospitalized for a NSTEMI requiring unplanned coronary artery revascularization. After commencing treatment, two patients were withdrawn from the placebo arm. One patient was withdrawn due to deranged liver function tests which breached the protocol-defined liver withdrawal criteria. The liver function tests normalized after cessation of statin therapy. The second patient was withdrawn from the placebo arm after contracting COVID-19 during the dosing phase of the trial (which was a withdrawal criterion during the pandemic). The COVID-19 infection was mild and self-limiting. One patient from the low-dose IL-2 arm was withdrawn after receiving two doses of IL-2 due to developing a generalized rash as a precautionary measure. The rash subsided 48 h after cessation of treatment.

**Table 2:** Adverse events

Event	Placebo (*n* = 31)	Low-dose IL-2 (*n* = 32)
Any AE, no. (%)	31 (100)	32 (100)
Maximal severity of any AE, no. (%)		
Mild	28 (90)	23 (72)
Moderate	3 (10)	9 (28)
Severe	0 (0)	0 (0)
AEs related to low-dose IL-2 or placebo, no. (%)	22 (71)	30 (94)
SAEs, no. (%)	1 (3)	0 (0)
AEs that led to interruptions of low-dose IL-2 or placebo, no. (%)	2 (6)	1 (3)
AEs that resulted in death, no. (%)	0 (0)	0 (0)
Infections (overall), no. (%)		
Total	4 (13)	3 (9)
COVID-19 infection	1 (3)	1 (3)
*Candida* Infection	0 (0)	1 (3)
Infected arthropod bite	0 (0)	1 (3)
Lower respiratory tract infection	1 (3)	0 (0)
Tooth infection	1 (3)	0 (0)
Urinary tract infection	1 (3)	0 (0)
Common AEs (>10%), no. (%)		
Administration site reactions^a^	5 (16)	29 (91)
Administration site bruising	25 (81)	18 (56)
Fatigue	8 (26)	10 (31)
Dizziness	9 (29)	7 (22)
Gastroesophageal reflux	1(3)	5 (16)
Noncardiac chest pain	5 (16)	0 (0)
Flu-like illness	2 (6)	4 (12)
Contusions	5 (16)	3 (9)
Backpain	0 (0)	5 (16)
Myalgia	2 (6)	5 (16)
Headache	4 (13)	6 (19)
Postprocedural contusions	7 (23)	5 (16)
Troponin increase	5 (16)	2 (6)

AEs are listed for all patients who had undergone randomization and received at least one dose of low-dose IL-2 or placebo.^a^Administration site reactions consisted of erythema, edema and pruritis of the injection site.

**Extended Data Fig. 4:**
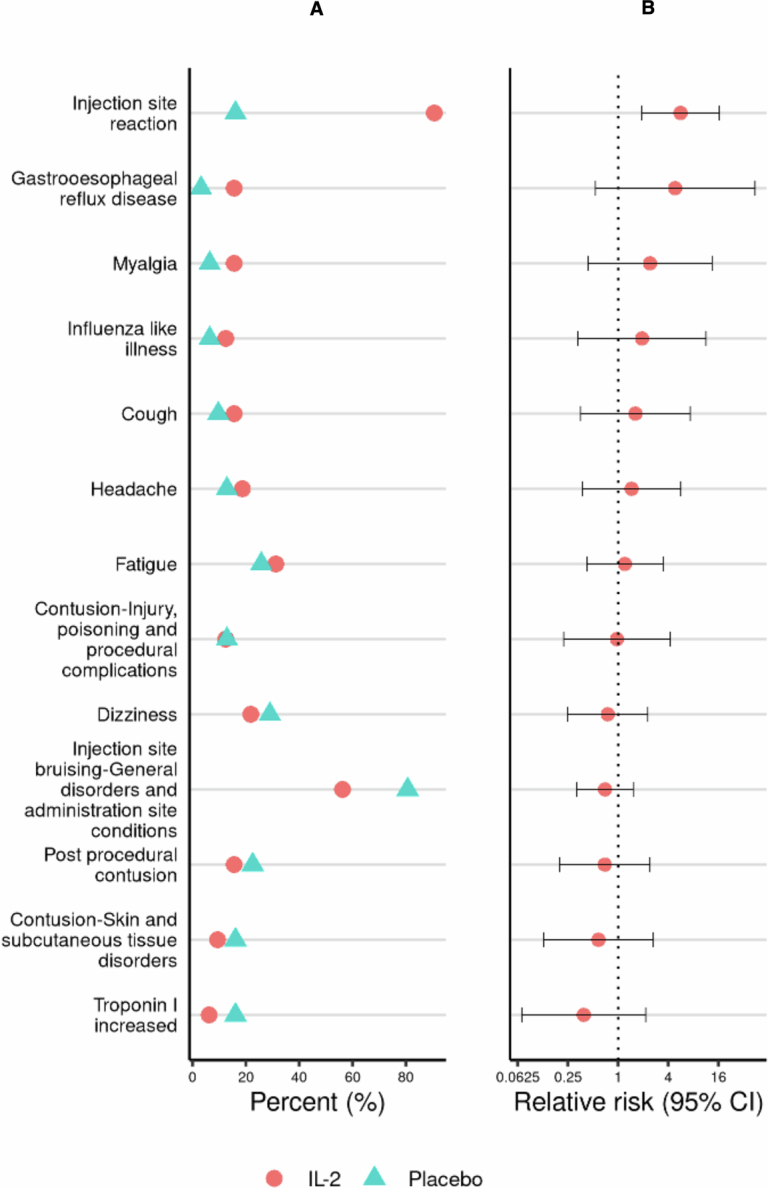
Absolute and relative risk of common ( > 10% at least in one arm) non-serious adverse events. 4 **A**. Absolute risk of non-serious AEs by group. The dots and triangles represent the percentage of patients experiencing AE of those who received low-dose IL-2 (red dot) or placebo (blue triangle). 4 **B**. The dot is the estimate of the relative risk of the AE comparing IL-2 to Placebo. The error bars represent the 95% confidence interval for that estimate. Placebo n = 31, Low-dose IL-2 n = 32.

Injection site reactions (ISRs), which included injection site-related erythema, edema and pruritis, were seen in 91% of patients who received low-dose IL-2 compared to placebo (relative risk = 5.6, *P* < 0.001). ISRs were mild in nature and resolved within 48 h. The frequency of other AEs did not significantly differ between the treatment arms (Supplementary Table 1 and Extended Data Fig. 4). Of note, there was no difference observed in the frequency of infections between the two groups. All infections observed in the trial were categorized as mild.

### IVORY-FINALE

**Fig. 4:**
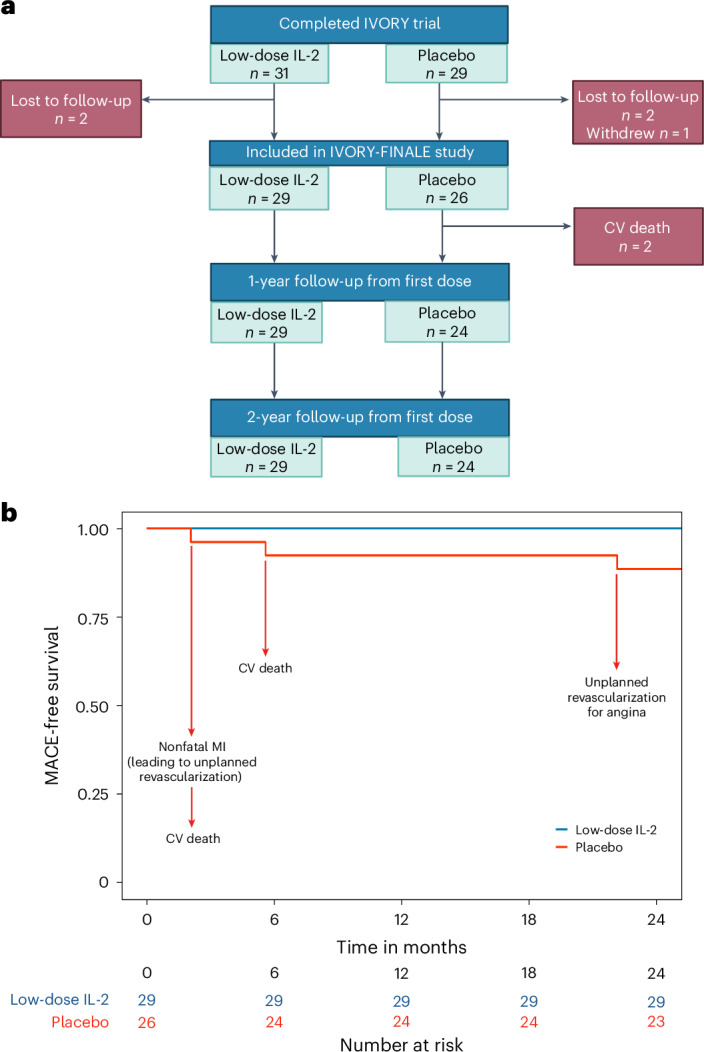
IVORY-FINALE study flow and MACEs at 2 years of follow-up. **a**, Participant flow in the IVORY-FINALE study. All patients who completed the IVORY trial were included in the study, unless they were lost to follow-up, declined to participate or withdrew consent. **b**, MACE-free survival up to 2 years from the first dose of treatment. MACE was defined as death due to a cardiovascular cause, resuscitated cardiac arrest, ischemic stroke, myocardial infarction and unplanned revascularization. MACEs occurred in three patients in the placebo arm and in no patients in the low-dose IL-2 arm. Patients were censored after their first event for the primary outcome analysis. CV, cardiovascular; MI, myocardial infarction. Panel **a** was created with BioRender.com.

**Extended Data Fig. 5:**
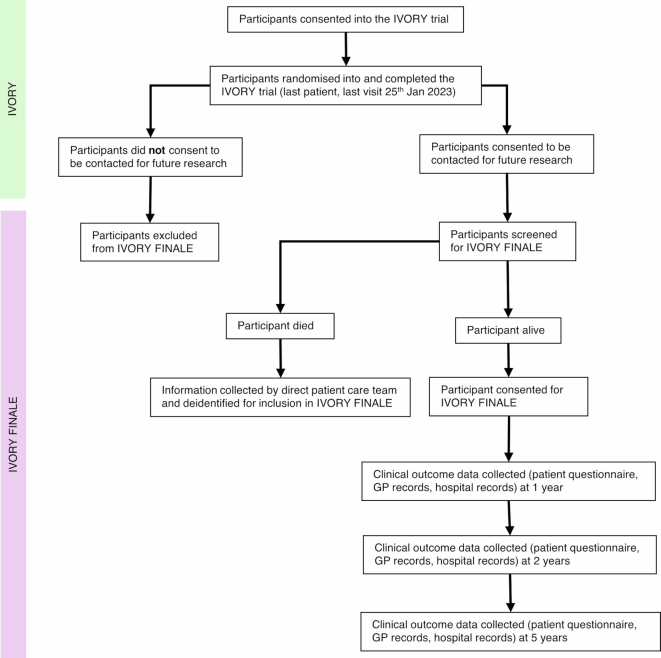
IVORY-FINALE study design. This figure depicts the study design for IVORY-FINALE. Patients who completed the IVORY trial were eligible to be enrolled in the IVORY-FINALE study. Pre-defined clinical cardiovascular outcomes were followed-up for those consented into the study at 1, 3 and 5 years.

The IVORY-FINALE study is a prospective observational study of cardiovascular outcomes in patients who completed the IVORY trial. Of the 60 patients who were eligible for inclusion in the IVORY-FINALE study, cardiovascular clinical outcomes were collected for 55 of them (Fig. 4a and Extended Data Fig. 5). The difference in composite MACE (cardiovascular death, resuscitated cardiac arrest, nonfatal myocardial infarction, ischemic stroke or unplanned coronary revascularization) between low-dose IL-2 and placebo is defined as the primary outcome of the IVORY-FINALE study. At 2 years of follow-up, 4 chronologically distinct MACE occurred in 3 (11%) patients in the placebo group. No MACEs occurred in the low-dose IL-2 arm. Figure 4b shows the MACE-free survival curves for the two groups and describes these MACEs in detail. Secondary outcomes, at 2 years of follow-up, are shown in Extended Data Table 3.

## Discussion

This phase 2, randomized, double-blind placebo-controlled trial met its primary outcome. In the IVORY trial, we demonstrated that treatment with low-dose IL-2 over an 8-week period reduced arterial inflammation in patients presenting with ACSs who had residual systemic inflammation when compared to placebo. The treatment effect of low-dose IL-2 was greater when baseline inflammation was higher. Our data show that harnessing the T_reg_ cells of the adaptive immune system with low-dose IL-2 is an effective and safe anti-inflammatory strategy in a high-risk ACS population. Anti-inflammatory therapies specifically targeting T cells have not been trialed in this population before and these results provide new efficacy data for the anti-inflammatory potential of low-dose IL-2 therapy in ACSs^31^. The difference of 7.3% between low-dose IL-2 and placebo for change in inflammation in the most diseased segments, when corrected for baseline, is comparable to the reduction in arterial inflammation observed for lipid-lowering therapies that have reduced MACEs in ACSs such as high-dose statins (when compared to low-dose stains) and alirocumab, proprotein convertase subtilisin or kexin type 9 antibody (when compared to a statin-naive placebo group)^32,33^. In these studies, differences of −10.6% (*P* = 0.01) and −8.2% (*P* = 0.05) were noted between groups. It is important to note that the targeted anti-inflammatory effect of low-dose IL-2 was observed on top of optimal guideline-directed medical therapy and in addition to the well-known pleiotropic anti-inflammatory effects of high-dose statins in the IVORY trial^32,34–36^.

Low-dose IL-2 increased circulating immunomodulatory T_reg_ cells. We hypothesized that the anti-inflammatory effect of low-dose IL-2 was a result of enhanced T_reg_ cell numbers and function^37^. The reduction in T_FH_ and T_H_1 cells, as well as cytotoxic CD8^+^ T cells observed with low-dose IL-2 therapy, is likely to further shift the immune profile of these individuals with residual inflammation toward a less inflammatory state. Furthermore, we have previously shown that, in patients presenting with ACSs, low-dose IL-2 increased both the number and the function of other immunoregulatory cells such as innate lymphoid cells type 2 and IL-10-producing regulatory B cells^38,39^. These are likely additional mechanisms by which low-dose IL-2 may lead to a reduction in arterial inflammation.

In IVORY, low-dose IL-2 therapy did not lead to a reduction in hsCRP compared to placebo. Although the sample size was not powered to detect a difference in hsCRP, which was an exploratory endpoint in this trial, the lack of significant change could also represent the fact that the low-dose IL-2-mediated anti-inflammatory effects occur through a different mechanism to that involving IL-6 signaling. This could also allow low-dose IL-2 to be used together with future therapies targeting the IL-6 pathway.

Treatment with low-dose IL-2 across 8 weeks was safe and well tolerated in patients with ACSs. Drug discontinuation rates were low (3%, *n* = 1) with IL-2 therapy. Infection rates were similar in both treatment arms (9% in low-dose IL-2 versus 13% in placebo) and they were all classified as mild. The only side effects observed at a significantly higher frequency were mild, transient and self-limiting ISRs, which have previously been observed in other low-dose IL-2 trials^18^. The side-effect profile of IL-2 therapy is dose dependent. Increasing T_reg_ cell numbers with low-dose IL-2 has not been associated with increased frequency of infections despite being trialed in immunocompromised populations such as those with HIV infection and graft-versus-host disease^40–42^. The IVORY trial used doses that were approximately 1% of the maximal dose per day and approximately 1.3% of the cumulative maximal doses used in the setting of metastatic renal cell carcinoma and malignant melanoma^43^. Even in the context of low-dose IL-2 trials, where the daily dose can vary between 0.09 × 10^6^ IU and 3 × 10^6^ IU, the cumulative dose used in IVORY was modest^18^. This likely explains the favorable side-effect profile of low-dose IL-2 in this trial, which is also in line with side effects observed in previous low-dose IL-2 trials that used similar cumulative doses^44,45^.

Furthermore, previous therapeutic agents have employed longer treatment durations. The one-off short treatment regimen in the IVORY trial, which allowed endogenous immunomodulatory mechanisms to reduce vascular inflammation, while sparing the body’s first line of defense against pathogens, could be a major additional benefit of low-dose IL-2 therapy in this patient population, if validated in larger clinical outcome studies.

As per protocol, our aim in this study was not to assess vascular [^18^F]FDG uptake limited to atherosclerotic plaques. The signal that we measured can be related to the presence of advanced atherosclerosis, but could also be related to early or pre-atherosclerotic vascular inflammation, as described in the Progression of Early Subclinical Atherosclerosis (PESA) study cohort^46^. This vascular [^18^F]FDG signal, which was mostly in plaque-free segments, was highly associated with plaque burden, defined by plaque presence, number and volume^46^. Moreover, in the PESA study, high arterial [^18^F]FDG activity was also associated with high bone marrow [^18^F]FDG activity and systemic inflammation^47^, which are all relevant to the progression of atherosclerotic cardiovascular disease.

This phase 2 study has some limitations. Patients with diabetes were underrepresented in this trial for methodological reasons relating to the use of [^18^F]FDG PET–CT, which led to the exclusion of patients with diabetes on insulin therapy. Furthermore, due to myocardial uptake of [^18^F]FDG, this biomarker does not allow the quantification of inflammation in coronary arteries without dietary manipulation. However, other imaging biomarkers for coronary artery inflammation are less validated in interventional clinical trials, which led to our selection of [^18^F]FDG for this trial. Finally, although early data from the IVORY-FINALE suggest a beneficial effect of low-dose IL-2 on MACEs, larger trials powered to assess the effect of low-dose IL-2 on clinical outcomes are needed.

In conclusion, in patients presenting with ACSs and residual systemic inflammation, an 8-week treatment course of low-dose IL-2 safely increased T_reg_ cells and led to a reduction of arterial inflammation, with a potential benefit on MACE recurrence.

## Methods

### Study design and oversight

IVORY was a parallel-group, double-blind, randomized, placebo-controlled, phase 2 trial (ClinicalTrials.gov registration: NCT04241601)^48^. This was an investigator-led trial and the funders had no role in the design, conduct or data analysis of the trial. It was conducted across two sites in the United Kingdom: Cambridge University Hospitals NHS Foundation Trust and Royal Papworth Hospital; 1,383 visits were carried out to complete the trial. The trial protocol was designed by the investigators (see Supplementary Information for the protocol). The trial received favorable ethical opinion from the Yorkshire and Humber—Sheffield ethics committee (19/YH/0171) as well as regulatory approval by the Medicines and Healthcare products Regulatory Authority (MHRA). The trial was conducted in accordance with the principles of the Declaration of Helsinki and the International Council for Harmonisation Guideline for Good Clinical Practice. Written informed consent was obtained from all patients before carrying out any study procedures for both IVORY and IVORY-FINALE. An independent data and safety monitoring committee (M. Marber, M. Dewey, R. Choudhury, G. Lombardi and E. Robinson) reviewed cumulative safety data to safeguard the well-being of the patients.

All patients, clinical investigators and research personnel carrying out study visits or undertaking immune profiling, biomarker and [^18^F]FDG PET–CT image analysis were blinded to treatment allocation. Treatment was administered as subcutaneous injections which looked identical (colorless liquid) at equal dose volumes. The PET–CT scans were analyzed with all patient identifiers (including trial identification numbers) and scan dates removed. Therefore, the PET–CT scan analyzers were blinded to the treatment allocation, patient identification details and dates of the scan.

IVORY-FINALE (ClinicalTrials.gov registration: NCT06427694) is a prospective observational study in which cardiovascular clinical outcome data are collected for patients who completed the IVORY trial (Extended Data Fig. 2). The study received favorable ethical opinion from the West Midlands—Edgbaston ethics committee (24/WM/0059) as well as research governance approval by the Health Research Authority (HRA). All prespecified clinical events for patients who completed the IVORY trial were included in this analysis, from the start of the IVORY trial (V1 onwards). The data were collected and analyzed by a team blinded to treatment allocation. A blinded, independent, clinical endpoint adjudication committee reviewed the MACE data.

Public and patient involvement occurred throughout the IVORY trial and this work was also featured on the BBC.

### Patients

Adults aged 18–85 years presenting with ACSs were eligible for screening. An ACS was defined as a diagnosis of unstable angina, NSTEMI and STEMI, in patients who had elevated troponin levels or dynamic electrocardiographic changes. A key inclusion criterion was a screening hsCRP level >2 mg l^−1^.

Major exclusion criteria included presentation with refractory cardiogenic shock or cardiac arrest, those receiving oral or intravenous immunosuppressive therapy, patients with diabetes on insulin therapy and patients with thyroid disorders. Due to the paucity of safety data in pregnancy for low-dose IL-2, women of childbearing potential were excluded. The full inclusion and exclusion criteria can be found in the trial protocol.

### Procedures

Patients were randomly allocated to subcutaneously administered placebo (5% dextrose) or 1.5 × 10^6^ IU of IL-2 in a 1:1 ratio using an independent, web-based application (www.sealedenvelope.com). Permutated block randomization was used, with random block sizes of 2, 4 and 6. Randomization was stratified by ST-segment elevation status.

After randomization, patients underwent an [^18^F]FDG PET–CT scan of the ascending aorta and carotid arteries in two bed positions, using established and reproducible methods on a dedicated hybrid scanner^20,22^. The imaging protocol used for the patients can be found in the IVORY trial protocol. Patients were fasted for 6 h before the scans and a dose of approximately 240 MBq of [^18^F]FDG was intravenously administered 90 min before image acquisition. The ascending aorta was imaged first, followed by the left and right common carotid arteries. These arterial beds were used because they are the most validated and reproducible in terms of quantifying arterial inflammation in [^18^F]FDG interventional drug trials^20,21^. Arterial [^18^F]FDG TBR_max_ was calculated as the mean arterial maximum standardized uptake value (SUV_max_) divided by the average of venous (SUV_mean_) from both internal jugular veins (for carotids) and the superior vena cava (for the ascending aorta). These methods are detailed further in the IVORY protocol and SAP document.

To ensure accurate serial measures, a number of approaches were employed, such as using a standardized validated imaging protocol with mandated [^18^F]FDG uptake times, injected dose and reconstruction parameters, a single dedicated scanner and a phantom study before the start for quantification accuracy.

**Extended Data Fig. 6:**
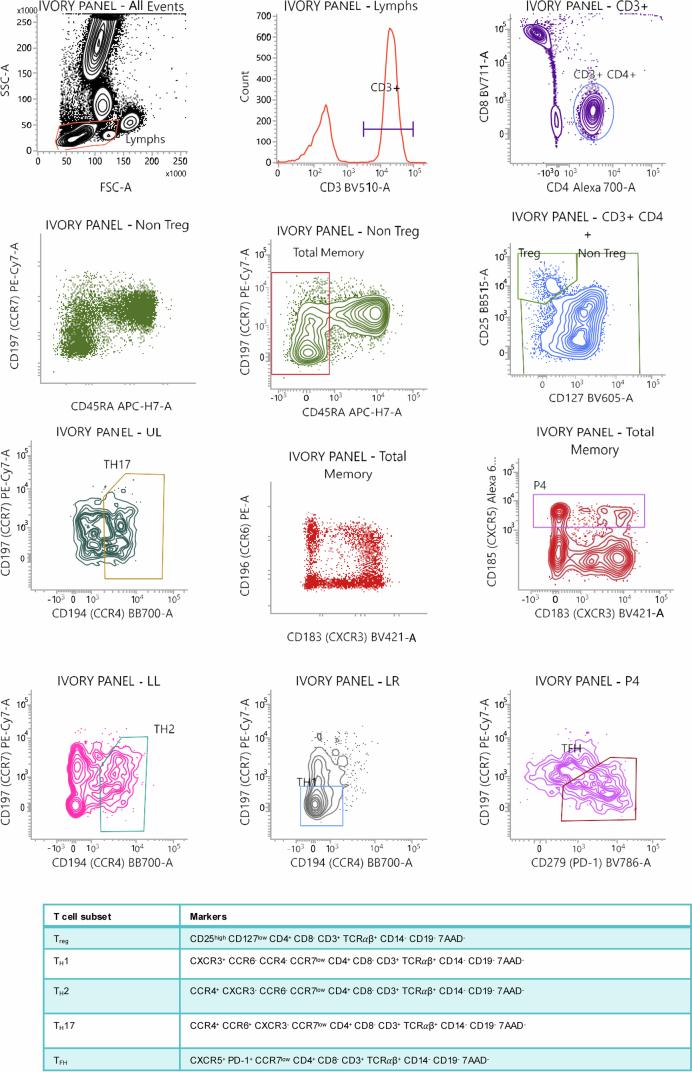
Phenotypic characterization of T lymphocyte subsets used in the fluorescence-activated cell sorting (FACS) analysis and cell surface markers used to define populations. This figure shows an example of contour plots created by FACS carried out in the IVORY trial. The table in the figure shows the cell surface markers used to define T cell populations in the IVORY trial.

Dosing commenced within 14 days of admission with ACSs. On completion of dosing, a post-treatment [^18^F]FDG PET–CT scan was undertaken. Immunophenotyping (Extended Data Fig. 6) was carried out at the start and end of the induction phase, at alternate visits during the maintenance phase and at approximately 1 week after cessation of treatment. Information with respect to the antibodies used for the FACS can be found in the Reporting Summary. The following antibodies were used according to the manufacturer’s instructions: CD196 phycoerythrin (PE) (20 μl in 100 μl of blood; BD Pharmingen, cat. no. 551773, clone 11A9), CD25 BB515 (5 μl in 100 μl of blood; BD Horizon, cat. no. 564467, clone 2A3), CD194 BB700 (5 μl in 100 μl of blood; BD Pharmingen, cat no. 566475, clone 1G1), CD197 Pe-Cy7 rat (5 μl in 100 μl of blood; BD Pharmingen, cat. no. 557648, clone 3D12), CD185 AF647 rat (5 μl in 100 μl of blood; BD Pharmingen, cat. no. 558113, clone RF8B2), CD4 AF700 (5 μl in 100 μl of blood; BD Pharmingen, cat. no. 557922, clone RPA-T4), CD45RA APC-H7 (5 μl in 100 μl of blood; BD Pharmingen, cat. no. 560674, clone HI100), CD183 BV421 (5 μl in 100 μl of blood; BD Horizon, cat. no. 562558, clone 1C6/CXCR3), CD3 BV510 (5 μl in 100 μl of blood; BD Horizon, cat. no. 563109, clone UCHT1), CD127 BV605 (5 μl in 100 μl of blood; BD Horizon, cat. no. 562662, clone HIL-7R-M21), CD8 BV711 (5 μl in 100 μl of blood; BD Horizon, cat. no. 563677, clone RPA-T8) and CD279 BV786 (5 μl in 100 μl of blood; BD Horizon, cat. no. 563789, clone EH12.1).

Patients who completed the IVORY trial were subsequently invited and then enrolled into the IVORY-FINALE study once written consent had been obtained. A telephone questionnaire was conducted to collect initial clinical outcome data. This was corroborated with medical notes (primary and/or secondary care) before declaration and reviewed by a clinical endpoint adjudication committee.

### Outcomes

The primary outcome of the trial was the absolute difference in the mean TBR_max_ of the index vessel at the end of treatment between the low-dose IL-2 group and the placebo group. This analysis included all segments of the index vessel. The index vessel was defined as the most inflamed vessel (the vessel with the highest mean TBR_max_) on the pre-treatment [^18^F]FDG PET–CT scans.

The secondary outcomes included the difference in mean TBR_max_ for more inflamed areas (active segments) of the index vessel at the end of treatment. The threshold for reporting the active segment analysis was predefined as segments with a mean TBR_max_ > 2 (ref. ^49^). Additional secondary outcomes included changes in circulating T_reg_ cells, T_eff_ cells and safety or tolerability of treatment and the additive treatment effect of low-dose IL-2 over placebo for various baseline levels of inflammation. The exploratory outcomes included differences between the groups for circulating biomarkers (for example, troponin and hsCRP) and left ventricular ejection fraction.

In the IVORY-FINALE study, the primary outcome was the difference in composite MACEs, including cardiovascular death, nonfatal myocardial infarction, resuscitated cardiac arrest, ischemic stroke or unplanned coronary revascularization, between low-dose IL-2 and placebo at a pre-specified follow-up of 1, 2 and 5 years. The secondary outcomes of the IVORY-FINALE study included differences between the groups in each component of the MACEs, hospitalizations and heart failure, all-cause mortality, amputations and revascularizations for peripheral vascular disease, hemorrhagic stroke, new atrial fibrillation and ventricular arrhythmia.

### Statistical analysis

For IVORY, the sample size was based on an absolute difference of 0.2 in the mean TBR_max_ of the index vessel between placebo and low-dose IL-2 at the end of the treatment period. This is equivalent to a 10% difference from a reference value of 2.02 and similar to the effect observed with high-dose statin therapy^32,50^. Assuming an s.d. of 0.24, 24 patients per arm, testing at a two-sided 5% significance level, provided 80% power. A sample size of 30 completed patients per arm was selected to account for any uninterpretable imaging or participant dropout. Recruitment ceased once 60 patients had completed the study.

For all continuous variables (including the primary outcome), point estimates and corresponding 95% CIs and *P* values are reported. For analyses of imaging data, including the primary analysis, linear regression models were used to estimate the treatment effect, adjusting where necessary for baseline covariates and their interactions with treatment. For secondary and exploratory outcomes that were repeatedly measured over time, linear mixed model repeated measures (MMRM) were used to estimate treatment effects for each time point and pooled across time. Where values for each time point are presented, a MMRM analysis with an unstructured covariance matrix for the within-patient residual errors was fitted between V3 and V15. The model specified fixed effects of treatment, baseline value, timing of assessment and an interaction between treatment allocation and timing. For values presented as pooled across time, a second model was fitted that considers only the treatment period up to V15 and does not have an interaction between treatment and time: this estimated the treatment effects averaged over the treatment period. The statistical package used was R. For the MMRM the ‘gls’ function from the ‘nlme’ package was used. The post-hoc analysis for vascular territory–treatment group interactions was done using a generalized least squares model.

For all imaging analyses, ST-elevation status, baseline hsCRP levels and high-dose versus nonhigh-dose statin use were considered for the main effects and interactions with treatment. In any change from baseline analyses presented, baseline values were used as a covariate. Imaging data and T cell data are presented for the full analysis population (as defined in the statistical analysis plan).

To explore the relationship between treatment effect and baseline inflammation, a sensitivity analysis varied the threshold used to define an active segment and 12 threshold values were considered; adjustment for multiplicity was done using the Benjamini–Hochberg process and adjusted and nonadjusted *P* values are presented for this analysis. A detailed statistical analysis plan can be found in Supplementary Information.

### Reporting summary

Further information on research design is available in the Nature Portfolio Reporting Summary linked to this article.

## Online content

Any methods, additional references, Nature Portfolio reporting summaries, source data, extended data, supplementary information, acknowledgements, peer review information; details of author contributions and competing interests; and statements of data and code availability are available at 10.1038/s41591-025-04090-y.

## Supplementary information

Supplementary Information Appendix 1 Supplementary Table 1, Appendix 2 IVORY trial first and final protocol, IVORY trial first and final SAP and IVORY-FINALE study protocol.

Reporting Summary

## Figures and Tables

**Extended Data Table 1:**
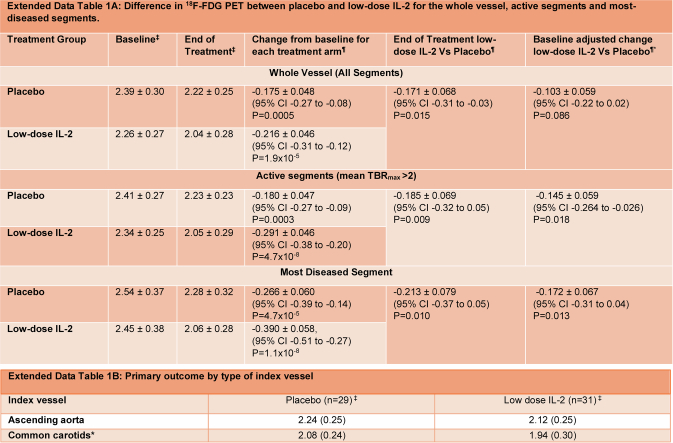
Arterial inflammation: components of the primary and secondary outcomes

Extended Data Table 1a^‡^Mean ± s.d. ^¶^Values are estimates of differences between the groups ± s.e. (95% CI). ^*^The baseline adjustment was done as detailed in SAP v2, where baseline mean TBR_max_ at study V2 (pre-treatment) was used as a covariate_._ The active segments include changes in segments of the vessel with a TBR_max_ > 2 at baseline. In the most disease segment analysis, changes in the segment of the vessel with the highest TBR_max_, along with the segment of the vessel above and below, are tracked to the end of treatment. The average of the three segments is compared pre-treatment and post-treatment. Linear regression models were used in reporting the estimated coefficients and their s.e. values. Some models only used treatment as a predictor, which is identical to an unpaired Student’s *t*-test (end of treatment column); others also adjusted for baseline value, which is identical to a baseline adjusted analysis of covariance (baseline adjusted change column). CIs and two-sided *P* values were provided based on the estimates following a *t*-distribution.

Extended Data Table 1b^*^The common carotids included values of either the right or left common carotid arteries, which were identified as the index vessel. ^‡^Mean (s.d.). A generalized least squares model was used to compare arterial territory–treatment group interactions, with fixed effects for the arterial territory (ascending aorta, common carotids), baseline value, treatment and the interaction between arterial territory and treatment. The interaction coefficient estimates the difference in treatment effect between different arterial territories. There was no evidence that the treatment effect was significantly different between arterial territories (*P* = 0.83).

**Extended Data Table 2:**
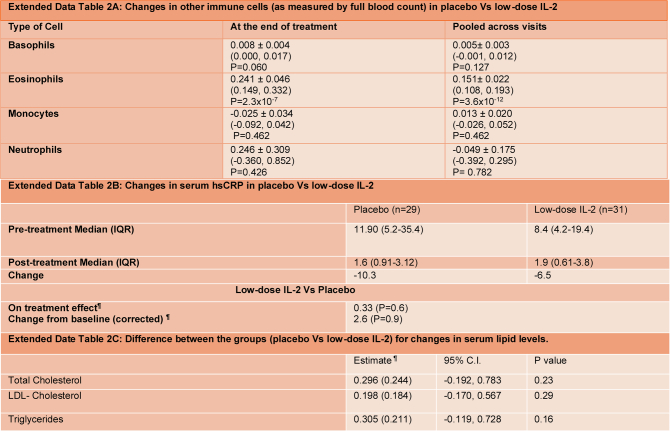
Changes in circulating (non-T) immune cells, hsCRP and lipids

Table 2a Values are estimates of differences between the groups for change from baseline ± s.e. (95% CI). Linear regression models were used in reporting the estimated coefficients and their s.e. values. Models used treatment as a predictor and adjusted for baseline value which is identical to a baseline adjusted analysis of covariance. CIs and two-sided *P* values were provided based on the estimates following a *t*-distribution.

Table 2b^¶^Plus–minus values are estimates of differences between groups. Linear regression models were used in reporting the estimated coefficients and their s.e. values. Some models only used treatment as a predictor, which is identical to an unpaired Student’s *t*-test (on treatment effect); others also adjusted for baseline value which is identical to a baseline adjusted analysis of covariance (change from baseline). CIs and two-sided *P* values were provided based on the estimates following a *t*-distribution.

Table 2c^¶^Plus–minus values are estimates of differences between groups (s.e.). Linear regression models were used reporting the estimated coefficients and their s.e. values. Treatment as a predictor and also adjusted for baseline value which is identical to a baseline adjusted analysis of covariance (change from baseline). CIs and two-sided *P* values were provided based on the estimates following a *t*-distribution.

**Extended Data Table 3:**
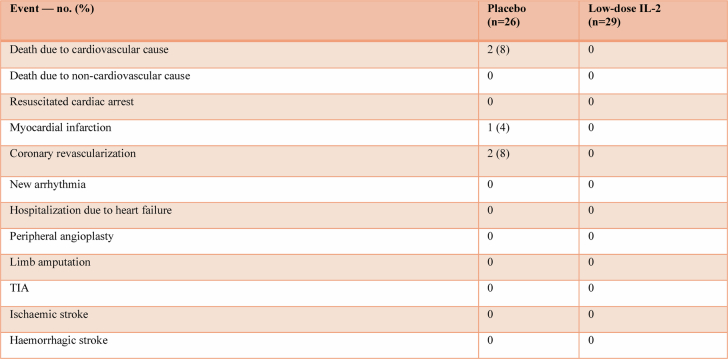
IVORY-FINALE study secondary outcomes

No. (%), number of participants (percentage of participants).

All events that occurred have been declared at 2 years of follow-up. One patient had three secondary outcome events after the index event (myocardial infarction, an unplanned coronary revascularization and death due to a cardiovascular cause).

## Data Availability

Deidentified participant data generated during the trial are available upon reasonable request from academic researchers or clinical researchers affiliated with recognized institutions. The data should be used for the purpose of conducting noncommercial, ethically approved research. A detailed research proposal, curriculum vitae and declaration of nonconflict of interest must be submitted by applicants. Requests must clearly describe the research design, objectives and methodology. They should not be in conflict with the trial objectives or overlap with any planned future research by the trial investigators. The review and approval for data requests will be done by the steering committee of the IVORY trial. Approval is granted based on scientific merit, data availability and intended use of data. Data requests will be considered within 12 months of manuscript publication.
