# Supplementary material for: Anti-inflammatory therapy with low-dose IL-2 in acute coronary syndromes: a randomized phase 2 trial
Source: Nat Med. 2026 Jan 8;32(2):624–32. doi: 10.1038/s41591-025-04090-y (PMC12920103; doi:10.1038/s41591-025-04090-y)
Supplement: Supplementary file 1 — Appendix 1 Supplementary Table 1, Appendix 2 IVORY trial first and final protocol, IVORY trial first and final SAP and IVORY-FINALE study protocol. [file 41591_2025_4090_MOESM1_ESM.pdf]

nature medicine

Supplementary information

https://doi.org/10.1038/s41591-025-04090-y

Anti-inflammatory therapy with low-dose IL-2 in acute coronary syndromes: a randomized phase 2 trial

In the format provided by the authors and unedited

Anti-inflammatory therapy with low-dose IL-2 in Acute Coronary Syndromes: a randomized phase 2 trial

Appendix 1

Supplementary Table 1: Non-serious adverse events by organ system

The percentages (%) refer to the % of patients in the group with the adverse event (AE). In brackets the number of patients who experienced AE is followed by the number of AEs, within the group

System Organ Class	Preferred Term	IL-2 (N = 32)	Placebo (N = 31)
**Blood and lymphatic system disorders**	Anaemia	0% (0, 0)	3% (1, 1)
	Lymphadenopathy	3% (1, 1)	3% (1, 1)
	Thrombocytopenia	0% (0, 0)	3% (1, 1)
**Cardiac disorders**	Angina pectoris	3% (1, 1)	0% (0, 0)
	Chest discomfort	6% (2, 3)	0% (0, 0)
	Chest pain	0% (0, 0)	6% (2, 3)
	Dizziness	29% (9, 12)	22% (7, 7)
	Dyspnoea	0% (0, 0)	9% (3, 3)
	Extrasystoles	0% (0, 0)	3% (1, 1)
	Intracardiac thrombus	10% (3, 3)	3% (1, 1)
	Oedema peripheral	0% (0, 0)	3% (1, 1)
	Palpitations	10% (3, 3)	9% (3, 3)
	Presyncope	3% (1, 1)	0% (0, 0)
	Syncope	0% (0, 0)	3% (1, 1)
**Ear and labyrinth disorders**	Tinnitus	3% (1, 1)	0% (0, 0)
**Endocrine disorders**	Goitre	3% (1, 1)	0% (0, 0)
	Type 2 diabetes mellitus	0% (0, 0)	3% (1, 1)
**Eye disorders**	Eye infection	3% (1, 1)	0% (0, 0)
**Gastrointestinal disorders**	Abdominal discomfort	0% (0, 0)	6% (2, 2)
	Diarrhoea	10% (3, 4)	6% (2, 2)
	Dyspepsia	3% (1, 1)	0% (0, 0)
	Gastroesophageal reflux disease	3% (1, 1)	16% (5, 5)
	Gingival pain	3% (1, 1)	0% (0, 0)
	Nausea	3% (1, 1)	3% (1, 1)
	Rectal haemorrhage	3% (1, 1)	0% (0, 0)
	Tongue ulceration	0% (0, 0)	3% (1, 1)
	Toothache	3% (1, 1)	0% (0, 0)
**General disorders and administration site conditions**	Chest discomfort	3% (1, 1)	6% (2, 2)
	Chills	6% (2, 2)	6% (2, 3)
	Crepitations	0% (0, 0)	3% (1, 1)
	Fatigue	26% (8, 9)	31% (10, 11)
	Feeling hot	3% (1, 1)	0% (0, 0)
	Hyperhidrosis	3% (1, 1)	3% (1, 1)
	Influenza like illness	6% (2, 2)	12% (4, 4)
	Injection site bruising	81% (25, 51)	56% (18, 36)

System Organ Class	Preferred Term	IL-2 (N = 32)	Placebo (N = 31)
	Injection site reaction	16% (5, 7)	91% (29, 135)
	Non-cardiac chest pain	16% (5, 5)	0% (0, 0)
	Peripheral coldness	0% (0, 0)	3% (1, 1)
	Vaccination site pain	0% (0, 0)	3% (1, 1)
	Vessel puncture site bruise	3% (1, 1)	0% (0, 0)
**Hepatobiliary disorders**	Haemangioma of liver	3% (1, 1)	0% (0, 0)
	Hepatic function abnormal	3% (1, 3)	9% (3, 4)
**Immune system disorders**	Allergy to arthropod bite	3% (1, 1)	3% (1, 1)
	Allergy to metals	0% (0, 0)	3% (1, 1)
	Hypersensitivity	3% (1, 1)	0% (0, 0)
	Seasonal allergy	3% (1, 1)	0% (0, 0)
**Infections and infestations**	COVID-19	3% (1, 1)	3% (1, 1)
	Candida infection	0% (0, 0)	3% (1, 1)
	Infected bite	0% (0, 0)	3% (1, 1)
	Lower respiratory tract infection	3% (1, 1)	0% (0, 0)
	Tooth infection	3% (1, 1)	0% (0, 0)
	Urinary tract infection	3% (1, 1)	0% (0, 0)
**Injury, poisoning and procedural complications**	Animal scratch	0% (0, 0)	3% (1, 1)
	Arthropod bite	0% (0, 0)	3% (1, 2)
	Contusion	13% (4, 4)	12% (4, 6)
	Foot fracture	3% (1, 1)	0% (0, 0)
	Incision site haematoma	3% (1, 1)	6% (2, 2)
	Limb injury	3% (1, 1)	0% (0, 0)
	Post procedural contusion	23% (7, 7)	16% (5, 6)
	Procedural pain	3% (1, 1)	3% (1, 1)
	Scratch	3% (1, 1)	0% (0, 0)
	Skin abrasion	3% (1, 1)	0% (0, 0)
	Thermal burn	0% (0, 0)	3% (1, 1)
**Investigations**	Haemangioma of liver	3% (1, 1)	0% (0, 0)
	Laboratory test interference	0% (0, 0)	6% (2, 2)
	Liver function test abnormal	0% (0, 0)	3% (1, 1)
	Pulmonary physical examination abnormal	3% (1, 1)	0% (0, 0)
	Troponin I increased	16% (5, 6)	6% (2, 2)
**Metabolism and nutrition disorders**	Diabetes mellitus	6% (2, 2)	0% (0, 0)
**Musculoskeletal and connective tissue disorders**	Arthralgia	10% (3, 3)	3% (1, 1)
	Back pain	0% (0, 0)	16% (5, 5)

System Organ Class	Preferred Term	IL-2 (N = 32)	Placebo (N = 31)
	Gout	0% (0, 0)	6% (2, 2)
	Muscle spasms	3% (1, 1)	0% (0, 0)
	Musculoskeletal chest pain	6% (2, 2)	3% (1, 1)
	Musculoskeletal pain	10% (3, 3)	9% (3, 3)
	Myalgia	6% (2, 2)	16% (5, 5)
	Neck pain	0% (0, 0)	3% (1, 1)
	Pain in extremity	3% (1, 1)	9% (3, 3)
	Pain in jaw	3% (1, 1)	0% (0, 0)
	Tendonitis	3% (1, 1)	0% (0, 0)
**Nervous system disorders**	Headache	13% (4, 6)	19% (6, 6)
	Migraine	3% (1, 1)	0% (0, 0)
	Neuralgia	0% (0, 0)	3% (1, 1)
	Ophthalmic migraine	3% (1, 1)	0% (0, 0)
	Paraesthesia	6% (2, 3)	3% (1, 1)
	Sciatica	0% (0, 0)	6% (2, 2)
	Somnolence	0% (0, 0)	3% (1, 1)
**Product issues**	Device pacing issue	0% (0, 0)	3% (1, 1)
**Psychiatric disorders**	Anxiety	0% (0, 0)	3% (1, 1)
**Renal and urinary disorders**	Flank pain	3% (1, 1)	0% (0, 0)
	Haematuria	3% (1, 1)	0% (0, 0)
	Nocturia	0% (0, 0)	3% (1, 1)
	Renal impairment	0% (0, 0)	3% (1, 1)
**Reproductive system and breast disorders**	Testicular swelling	0% (0, 0)	3% (1, 1)
**Respiratory, thoracic and mediastinal disorders**	Cough	10% (3, 3)	16% (5, 5)
	Dyspnoea	6% (2, 2)	9% (3, 3)
	Epistaxis	0% (0, 0)	3% (1, 1)
	Hyperventilation	3% (1, 1)	6% (2, 2)
	Lower respiratory tract infection	0% (0, 0)	3% (1, 1)
	Lung opacity	0% (0, 0)	3% (1, 1)
	Oropharyngeal pain	3% (1, 1)	3% (1, 1)
	Pulmonary mass	0% (0, 0)	3% (1, 1)
	Rhinitis	0% (0, 0)	3% (1, 1)
	Rhinorrhoea	0% (0, 0)	6% (2, 2)
**Skin and subcutaneous tissue disorders**	Contusion	16% (5, 14)	9% (3, 6)
	Dermatitis herpetiformis	0% (0, 0)	3% (1, 1)
	Dry skin	3% (1, 1)	0% (0, 0)
	Erythema	0% (0, 0)	6% (2, 2)
	Injection site bruising	3% (1, 1)	0% (0, 0)
	Paraesthesia	3% (1, 2)	6% (2, 2)
	Pruritus	3% (1, 1)	3% (1, 1)
	Rash	10% (3, 3)	9% (3, 4)
	Skin abrasion	3% (1, 1)	0% (0, 0)

System Organ Class	Preferred Term	IL-2 (N = 32)	Placebo (N = 31)
	Skin swelling	0% (0, 0)	3% (1, 1)
	Vessel puncture site bruise	6% (2, 3)	6% (2, 3)

Clinical Trial Protocol

Trial Title:	**Low-dose interleukin-2 for the reduction of vascular inflammation in Acute Coronary Syndromes (IVORY)**
Protocol Number:	IVORY
EudraCT Number:	2017-005130-27
Investigational Product:	Aldesleukin (IL-2)

Chief Investigator:	Dr Joseph Cheriyan
CI Address:	Cambridge University Hospitals NHS Foundation Trust,<br>Box 128, Level 3,<br>ACCI Building,<br>Hills Road,<br>Cambridge,<br>CB2 0QQ
Telephone:	01223 296070
Trial Sponsor:	Cambridge University Hospitals NHS Foundation Trust and the University of Cambridge
SAE Reporting:	IVORY Clinical Trial Coordinator<br>Email cambs.cardiovascular@nhs.net

Protocol Signatures

I give my approval for the attached protocol entitled Low-dose interleukin-2 for the reduction of vascular inflammation in Acute Coronary Syndromes (IVORY) dated 04 October 2019

Chief Investigator

Name: Joseph Cheriyan

Signature: ____

Date:

Site Signatures

I have read the attached protocol entitled "Low-dose interleukin-2 for the reduction of vascular inflammation in Acute Coronary Syndromes (IVORY)" dated 04 October 2019 and agree to abide by all provisions set forth therein.

I agree to comply with the conditions and principles of Good Clinical Practice as outlined in the European Clinical Trials Directives 2001/20/EC and 2005/28/EC, the Medicines for Human Use (Clinical Trials) Regulations 2004 (SI 2004/1031) and any subsequent amendments of the clinical trial regulations, the Sponsor's SOPs, and other regulatory requirements as amended.

I agree to ensure that the confidential information contained in this document will not be used for any other purpose other than the evaluation or conduct of the clinical investigation without the prior written consent of the Sponsor

Principal Investigator

Name:

Signature: ____

Date: ______________________________

1 Protocol Contributors

Chief Investigator:	
Dr Joseph Cheriyan	Cambridge University Hospitals NHS Foundation Trust and Division of Experimental Medicine & Immunotherapeutics, Dept of Medicine, Univ. of Cambridge, Box 128, Hills Road, Cambridge, CB2 0QQ<br>Tel.: 01223 256653
Sub-Investigators / Collaborators:	
Professor Ziad Mallat	Division of Cardiovascular Medicine, Department of Medicine, Univ. of Cambridge, Forvie Site, Robinson Way, Cambridge, CB2 0SZ
Dr Stephen Hoole	Royal Papworth Hospital NHS Foundation Trust, Cambridge Biomedical Campus, Cambridge, CB2 0AY
Dr James Rudd	Division of Cardiovascular Medicine, Department of Medicine, Univ. of Cambridge, Cambridge, CB2 0QQ
Dr Rouchelle Sriranjan	Division of Cardiovascular Medicine, Department of Medicine, Univ. of Cambridge, Forvie Site, Robinson Way, Cambridge, CB2 0SZ
Dr Michalis Kostapanos	Division of Experimental Medicine & Immunotherapeutics, Dept of Medicine, Univ. of Cambridge, Addenbrooke's Hospital, Hills Road, Cambridge, CB2 0QQ
Dr Tian Zhao	Division of Cardiovascular Medicine, Department of Medicine, Univ. of Cambridge, Forvie Site, Robinson Way, Cambridge, CB2 0SZ
Dr Jason Tarkin	Division of Cardiovascular Medicine, Department of Medicine, Univ. of Cambridge, Cambridge, CB2 0QQ

Trial coordination:	
Heike Templin	Cardiovascular Trials Office, Cambridge Clinical Trials Unit, Box 401, Cambridge University Hospitals NHS Foundation Trust, Hills Road, Cambridge, CB2 0QQ<br>Email: heike.templin@addenbrookes.nhs.uk
**Trial statistician:**	
Dr Simon Bond	Cambridge Clinical Trials Unit, Box 401, Cambridge University Hospitals NHS Foundation Trust, Hills Road, Cambridge, CB2 0QQ
**Trial pharmacist:**	
Dr Lynne Whitehead	Central Pharmacy Level 1, Box 55 Cambridge University Hospitals NHS Foundation Trust, Hills Road, Cambridge, CB2 0QQ

Amendment History

Version No.	History	Date
v1.0	Final Protocol	08 May 2019
v1.1	Incorporating REC/HRA comments	15 Jul 2019
v2.0	Incorporation MHRA feedback on initial submission	Aug 2019

2 Table of Contents

Protocol Signatures 2
1 Protocol Contributors 3
2 Table of Contents 5
3 Abbreviations 9
4 Trial Synopsis 10
5 Trial Figures 17
5.1 Trial Design Figure 17
5.2 Trial Design Per Patient 18
6 Introduction 19
6.1 Background 19
6.2 Aldesleukin 21
6.3 Clinical use of IL-2 21
7 Rationale for Trial 24
7.1 Hypotheses 26
8 Trial Design 26
8.1 Statement of Design 26
8.2 Number of Centres 26
8.3 Number of Patients 26
8.4 Trial Duration 26
8.5 Trial Objectives 27
8.5.1 Primary objective 27
8.5.2 Secondary objectives 27
8.5.3 Exploratory objectives 27
8.6 Trial endpoints 27
8.6.1 Primary endpoint: 27
8.6.2 Secondary endpoints: 27
8.6.3 Exploratory endpoints: 28
9 Selection and withdrawal of patients 28
9.1 Inclusion Criteria 28
9.2 Exclusion Criteria 29
9.3 Treatment Assignment and Randomisation Number 30
9.4 Method of Blinding 30
9.5 Patient Withdrawal Criteria 30
9.5.1 Liver withdrawal criteria 30
9.5.2 Renal withdrawal criteria 31
9.5.3 Cardiac withdrawal criteria 31
9.5.4 General withdrawal criteria 32

9.5.5 Management of withdrawal 32
9.6 Trial stopping criteria 32
10 Trial Treatments 33
10.1 Dosage schedules 33
10.1.1 Route of Administration and Maximum dosage allowed 33
10.1.2 Maximum duration of treatment of a patient 33
10.2 Presentation of the drug 33
10.3 Known drug reactions & interaction with other therapies 34
10.4 Dosage modifications 34
10.5 Legal status of the drug 34
10.6 Drug storage and supply 34
10.7 Accountability 34
10.8 Concomitant Therapy 34
10.9 Emergency unblinding 35
11 Procedures and assessments 35
11.1 Patient identification 35
11.2 Consent 35
11.3 Screening evaluation 36
11.3.1 Screening Assessments (**Visit 1**) Day -7 to 0 36
11.3.2 Patient Randomisation 37
11.4 Trial assessments 37
11.4.1 ^{18}F-FDG PET/CT Scan 1 (**Visit 2**) Day -6 to 0 37
11.4.2 Induction phase – **(Visit 3-7)** Day 1-5 38
11.4.3 Maintenance Phase – (**Visits 8 -14)** Day 12-54 39
11.4.4 Post-dose PET/CT – (**Visit 15**) Day 61 40
11.4.5 Follow-up – (**Visit 16**) Day 82 40
11.5 Schedule of Assessments 42
11.6 Trial specific procedures 44
11.7 End of Trial Participation 45
11.8 Trial restrictions 45
12 Assessment of Safety 46
12.1 Definitions 46
12.1.1 Adverse event (AE) 46
12.1.2 Adverse reaction to an investigational medicinal product (AR) 46
12.1.3 Unexpected adverse reaction 46
12.1.4 Serious adverse event or serious adverse reaction (SAE / SAR) 47
12.1.5 Suspected Unexpected Serious Adverse Reaction (SUSAR) 47
12.1.6 Reference Safety Information (RSI) 47
12.2 Expected Adverse Reactions/Serious Adverse Reactions (AR /SARs) 48
12.2.1 Injection site reaction 48

12.3 Expected Adverse Events/Serious Adverse Events (AE/SAE) 49
12.3.1 Hospital admissions 49
12.3.2 Events commonly occurring in patients with ACS 49
12.4 Evaluation of adverse events 50
12.4.1 Assessment of seriousness 50
12.4.2 Assessment of causality 50
12.4.3 Clinical assessment of severity 50
12.4.4 Recording of adverse events 50
12.5 Reporting serious adverse events 51
12.6 Reporting of Suspected Unexpected Serious Adverse Reactions (SUSARs) 51
12.7 Pregnancy Reporting 52
13 Toxicity – Emergency Procedures 53
14 Evaluation of results 53
14.1 Response criteria 53
15 Storage and Analysis of Samples 53
16 Statistics 53
16.1 Statistical methods 53
16.2 Interim analyses 54
16.3 Number of Patients to be enrolled 54
16.3.1 Safety Population 54
16.3.2 Analysis Population 54
16.4 Procedure to account for missing or spurious data 55
16.5 Definition of the end of the trial 55
17 Data handling and record keeping 55
17.1 CRF 55
17.2 Source Data 55
17.3 Data Protection & Patient Confidentiality 56
18 Trial Management Group 56
19 Independent Data Monitoring Committee 56
20 Ethical & Regulatory considerations 56
20.1 Ethical committee review 56
20.2 Regulatory Compliance 56
20.3 Protocol Amendments 57
20.4 Peer Review 57
20.5 Declaration of Helsinki and Good Clinical Practice 57
20.6 GCP Training 57
21 Sponsorship, Financial and Insurance 57
22 Monitoring, Audit & Inspection 57
23 Protocol Compliance and Breaches of GCP 58
24 Publications policy 58

25 References 58

26 Appendices 62

26.1 Appendix 1 – Symptoms and signs documented as AEs in the LILACS trial (MHRA reference 24551/0029/001-0001; REC reference 17/NW/0012; ClinicalTrials.gov Identifier NCT03113773) 62

26.2 Appendix 2 - Safety reporting flow-chart 64

3 Abbreviations

AE/AR	Adverse event/Adverse Reaction
ACS	Acute Coronary Syndrome
ALP	Alkaline phosphatase
ALT	Alanine aminotransferase
AST	Aspartate aminotransferase
BNP	B-type Natriuretic Peptide
CA	Competent Authority
CABG	Coronary artery bypass graft
CCTU	Cambridge Clinical Trials Unit
CI	Chief investigator
CNS	Central nervous system
CRF	Case Report Form
CT	Computed Tomography
CV	cardiovascular
DMC	Data Monitoring Committee
DSUR	Development Safety Update Report
ECG	Electrocardiogram
FDG - PET/CT	Fluorodeoxyglucose - Positron emission tomography/ computed tomography
GCP	Good Clinical Practice
GP	General Practitioner
hs-CRP	High-Sensitivity C-Reactive Protein
ICF	Informed Consent Form
IHD	Ischaemic heart disease
IMP	Investigational Medicinal Product
ISR	Injection site reaction
IU	International Unit
MHRA	Medicines and Healthcare products Regulatory Agency
NIMP	Non Investigational Medicinal Product
NSTEMI	Non-ST Elevation Myocardial Infarction
PBMC	Peripheral Blood Mononuclear Cell assay
PCI	Percutaneous coronary intervention
PET	Positron Emission Tomography
PIS	Patient Information Sheet
QTcB	Corrected QT using Bazett's formula
R&D	Research and Development
RA	Regulatory Agency
REC	Research Ethics Committee
RSI	Reference Safety Information
SAE/SAR	Serious Adverse Event/Serious Adverse Reaction
SmPC	Summary of Product Characteristics
STEMI	ST elevation myocardial infarction
ST	ECG parameter
SUSAR	Suspected Unexpected Serious Adverse Reaction
SUV	Standardized Uptake Value
TBR	Tissue-to-blood ratio
TMG	Trial Management Group
TnI	Troponin I
Treg	Regulatory T cells

TSH	Thyroid Stimulating Hormone
TTE	Transthoracic echocardiogram
ULN	Upper Limit of Normal

4 Trial Synopsis

Title of clinical trial	Low-dose interleukin-2 for the reduction of vascular inflammation in Acute Coronary Syndromes
Sponsor name	Cambridge University Hospitals NHS Foundation Trust and the University of Cambridge
Medical condition or disease under investigation	Acute Coronary Syndrome
Purpose of clinical trial	To investigate the efficacy of repeated low doses of interleukin-2 (IL-2) in reducing vascular inflammation in ACS
Primary objective	To compare the effect of low dose IL-2 against placebo on vascular inflammation using ^{18}F-FDG PET/CT in ACS.
Secondary objective (s)	• To determine if low dose IL-2 can increase Regulatory T cell (Treg) numbers sustainably over extended treatment<br>• To determine the effect of low dose IL-2 on systemic inflammation measured by cardiovascular biomarkers (including but not limited to hsCRP, IL-6, N-terminal pro-brain natriuretic peptide (BNP) and Troponin I.<br>• To determine the safety and tolerability of extended dosing of low dose IL-2 in patients with acute coronary syndrome
Trial Design	Phase 2, randomised, double-blinded, placebo-controlled experimental trial
Trial endpoints	Primary endpoint:<br>Change in vascular inflammation (as measured by mean TBR max in the index vessel) on ^{18}F-FDG PET/CT from baseline to follow up scans<br>Secondary endpoints:<br>• Change in mean TBR_{max} in each arterial region at baseline and follow-up<br>• Change in Lymphocyte subsets (T effector cells (Teffs), natural killer cells and B lymphocytes)<br>• Change in percentage of Treg cell numbers

	• Change in serum cardiac biomarkers<br>•
Sample Size	n=60 (30 per arm)
Summary of eligibility criteria	Inclusion Criteria:<br>• Able to provide written informed consent to participate<br>• Age between 18 and 85<br>Current admission (on the screening visit) with ACS with symptoms suggestive of myocardial ischaemia lasting 10 minutes or longer with the patient at rest or with minimal effort AND<br>i) EITHER elevated levels of TnI on admission<br>OR<br>ii) dynamic changes in ECG (new ST changes, T-wave inversion)<br><br>• Where applicable, to be included in the trial women must be<br>i. Postmenopausal (for the purposes of this trial, postmenopausal is defined as being amenorrhoeic for greater than 2 years with an appropriate clinical profile, e.g. age appropriate, history of vasomotor symptoms)<br>OR<br>ii. Have had a documented hysterectomy and/or bilateral oophorectomy or sterilisation<br>OR<br>iii. Peri-menopausal with a negative pregnancy test at screening (for the purposes of this trial.Peri-menopausal is defined as women with an appropriate clinical profile, e.g. age appropriate, history of vasomotor symptoms, irregular periods). These women will be expected to comply with the use of contraception for the duration of the trial and undergo additional pregnancy tests during and after treatment.<br>iv.<br>• High sensitivity C-reactive protein of >2 mg/l at screening<br>• Willingness and possibility to start dosing within 8 days from initial date of admission to the primary hospital for ACS<br>• Able to comply with all trial mandated visits<br><br>Exclusion Criteria

	• Current presentation (at screening) with cardiogenic shock (systolic blood pressure <80 mm Hg that is unresponsive to fluids or necessitates administration of catecholamines<br>• Current presentation with cardiac arrest<br>• Signs or symptoms of active infection requiring intravenous antibiotic treatment at screening<br>• History of malignancies requiring active treatment. (However, patients with a history of treated localised basal or squamous cell skin cancer are not excluded from participation in this trial.)<br>• History of solid organ transplantation or other bone marrow transplantation<br>• History of recurrent epileptic seizures in the previous 4 years; repetitive or difficult to control seizures, coma or toxic psychosis lasting >48 hours<br>Uncontrolled hypotension (Systolic BP (SBP)<80mmHg or DBP<50mmHg) OR uncontrolled hypertension (SBP>180 or DBP>120 mmHg) at screening<br>• Average corrected QT interval (QTc) > 450 msecs using Bazett's formula from average of triplicate ECGs (or > 480 msecs if bundle branch block)<br>• Renal impairment (Creatinine clearance [Cockcroft-Gault] <45ml/min) at screening<br>• Liver dysfunction (ALT > 2xULN) at screening<br>• Evidence of cholestasis defined as elevated Total Bilirubin Levels, (TBL > 1.5 x ULN) and Alkaline Phosphatase, ALP (ALP > 1.5 x ULN), at screening<br>• Known hypothyroidism/hyperthyroidism<br>• Known autoimmune disease requiring active immunosuppressive treatment<br>• Any regular oral or intravenous immunosuppressive treatment including prednisolone, hydrocortisone or disease modifying drugs. [Inhaled or topical steroids are permissible]<br>• Patients on cytotoxic drugs and interferon-alpha<br>• Known Type 1 or Type 2 diabetes

	mellitus<br>● Contraindication to IL-2 treatment or hypersensitivity to IL-2 or to any of its excipients<br>● Participation in a previous research trial in the last 3 years which involved exposure to significant ionising radiation (i.e. cumulative research radiation dose >5 mSv)<br>● Participation in a clinical trial where the patient has received a drug or new chemical entity within 30 days or 5 half-lives, or twice the duration of the biological effect of the drug (whichever is longer) prior to the first dose of trial medication, Visit 3 (Day 1).<br>● Any medical history or clinically relevant abnormality that is deemed by the principal investigator/delegate to make the patient ineligible for inclusion because of a safety concern<br>● Pregnant women or breast feeding women
Investigational medicinal product and dosage	Aldesleukin (1.5 x 10^6 IU)
Comparator product(s)	Placebo subcutaneous injection (dextrose 5%)
Route(s) of administration	Subcutaneous injection
Maximum duration of treatment of a patient	8 weeks
Procedures: Screening & enrolment	**V1 (Day -7 to 0)**<br>● Demography (date of birth, age, gender and race)<br>● Medical history<br>● Current medication history<br>● Checking of inclusion/exclusion criteria<br>● Physical examination (including cardiovascular, respiratory, gastrointestinal, neurological and skin examinations)<br>● Height and weight<br>● Vital observations (defined henceforth as temperature, blood pressure, heart rate, respiratory rate and oxygen saturations)<br>● 12-lead electrocardiogram (ECG) in triplicate with an average QTcB taken<br>● TTE (in cases where a TTE may not have been obtained by Day 0, this will not delay

	dosing the patient)<br>● Screening bloods (approximately 15mls) including:<br>○ Safety blood tests (defined in section 11.6)<br>○ Thyroid function tests (defined in section 11.6)<br>○ Serum pregnancy test where applicable<br>○ hsCRP<br>○ Full lipid profile
Procedures: Baseline	**V2 (Day -6 to 0)**<br>• Review of eligibility and withdrawal criteria<br>• Adverse events<br>• Concomitant medications<br>• Finger prick blood sugar test<br>• Insertion of IV cannula<br>• FDG-PET/CT scan (lasting approximately 2 hours with a 90-minute break after radioligand injection)
Procedures:<br>Treatment period	**V3-V7 (Day 1-5)**<br>● Review of eligibility and withdrawal criteria at V3 and withdrawal criteria at V3 and all subsequent visits<br>● Adverse events<br>● Concomitant medications<br>● Vital observations<br>● Physical examination<br>● 12-lead ECG with QTcB measurement pre-dosing<br>● Review of safety bloods from the previous visit (V4 onwards)<br>● Pre-dose bloods for V3 and V7 (approximately 55mls):<br>○ Clinical safety bloods<br>○ Treg and Lymphocyte subset analysis<br>○ Peripheral Blood Mononuclear Cell (PBMC) assays<br>○ Cardiac biomarkers<br>● Pre-dose bloods for V4-6 (approximately 15mls):<br>○ Clinical safety bloods<br>● Injection of IMP<br>● 12-lead ECG with QTcB measurement approximately 30 mins post-dosing<br>● Vital observations approximately 30 mins post-dosing

	**V8-V14 (Day 12-54)**<br>● Check withdrawal criteria<br>● Adverse events<br>● Concomitant medications<br>● Vital observation<br>● Brief physical examination<br>● 12-lead ECG with QTcB measurement<br>● Pre-dose blood tests (approx. 20mls in total):<br>● Clinical safety bloods Pre-dose blood tests V8, V10, V12, V14 only:<br>○ Clinical safety bloods<br>○ Treg and Lymphocyte subset analysis<br>○ PBMC assay<br>○ Cardiac biomarkers<br>○ Serum pregnancy test where applicable (V10 and V14 only)<br>● Injection of IMP<br>● 12-lead ECG with QTcB measurement approximately 30 mins post-dosing<br>● Vital observations approximately 30 mins post-dosing<br>● Transthoracic echocardiogram (TTE) (anytime between V14 and V16) (ad hoc visit if required)
Procedures: End of trial	**V15 Day 61 (+/-3)**<br>● Transthoracic echocardiogram (TTE) (if not done before)<br>● Insertion of IV cannula<br>● Pre-scan bloods:<br>○ Cardiac biomarkers<br>○ Treg and Lymphocyte subset analysis<br>○ PBMC assay<br>○ Full lipid profile<br>● Finger prick blood sugar test<br>● Follow up FDG-PET/CT (lasting approximately 2 hours with a 90-minute break after radioligand injection).<br>**V16 Follow-up visit Day 82 (+/- 3)**<br>Patients will have the following assessments during the follow-up visit:<br>● Adverse events<br>● Concomitant medications<br>● Vital observation<br>● Physical examination<br>● 12-lead ECG with QTcB measurement<br>● Follow-up blood tests (55 mls in total):<br>○ Clinical safety bloods<br>○ Tregs and Lymphocyte subset

	analysis<br>o PBMC assay<br>o Cardiac biomarkers<br>o Thyroid function test<br>o Serum pregnancy test where applicable
Procedures for safety monitoring during trial	An unblinded independent DMC will review all safety (but not exploratory) data. There will be no interim analysis.
Criteria for withdrawal of patients	General withdrawal criteria<br>• Cardiorespiratory arrest<br>• Failure to attend two scheduled dosing appointments without adequate reason based on the PI assessment<br>• Severe hypersensitivity reactions will preclude any further drug administration<br>• New seizure activity<br>• Coma<br>• Severe lethargy or somnolence<br>• Respiratory insufficiency requiring intubation<br>• Pregnancy<br>• Withdrawal of consent<br>• PI discretion<br>• Any serious adverse reaction (SAR) or adverse reaction which is deemed by investigators as Severe (AR).<br>• Any significant incidental finding on PET/CT scan clinical governance reports, which in the opinion of the PI, necessitates further investigation and management.<br>• Any medical history, clinically relevant abnormality or reason that is deemed by the principal investigator (PI) to make the patient ineligibile to continue the trial.<br><br>For specific organ based withdrawal criteria see section 9.5

5 Trial Figures

5.1 Trial Design Figure

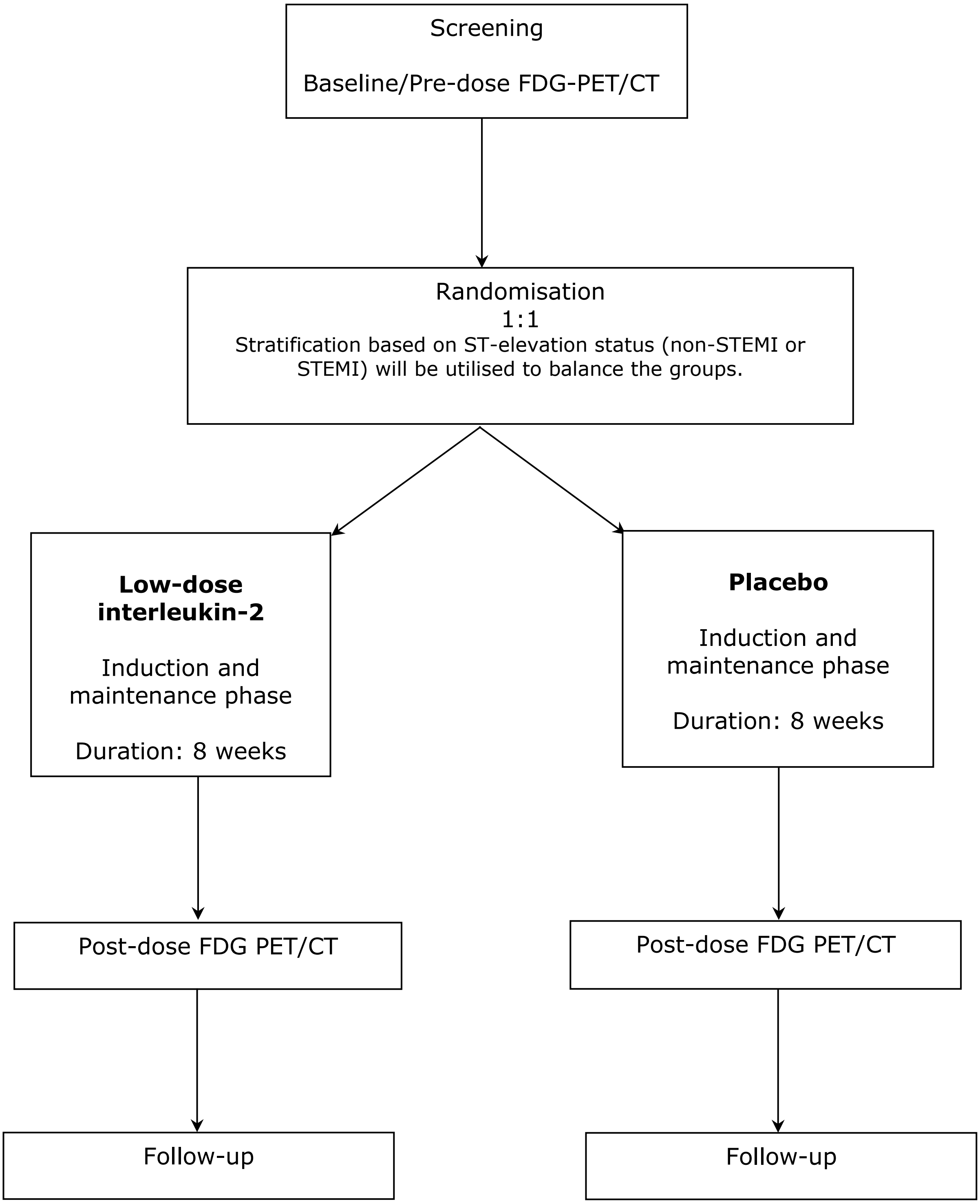

5.2 Trial Design Per Patient

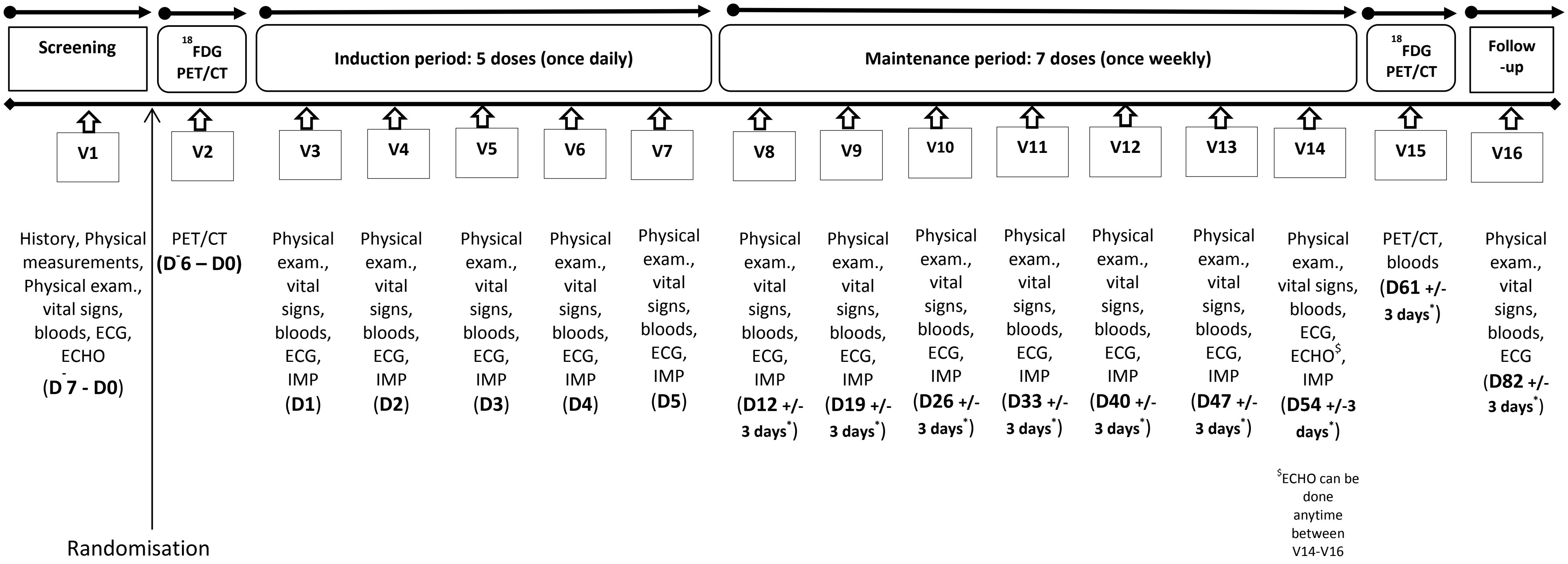

* +/3 days from the intended previous visit

6 Introduction

6.1 Background

Acute coronary syndromes (ACS) result from coronary plaque(s) disruption, which initiates a thrombotic process leading to partial or complete obstruction of the vessel lumen with subsequent myocardial ischaemia and necrosis[1,2]. The mainstay of treatment is currently focussed on the re-establishment and maintenance of coronary artery patency using anti-platelets and anticoagulants with or without mechanical dilatation and stenting of the culprit artery[1,2]. Despite important advances in management, ACS still carries a risk of substantial morbidity and mortality[1]. The improved efficacy of novel anti-platelet and anticoagulant agents have been limited by increased risk of haemorrhagic events[3,4]. Thus, future breakthroughs in management are most likely to arise from targeting other relevant pathophysiological pathways. Particularly, we believe that the immune response is an important process that has been neglected in the management of patients with ACS.

6.1.1 Regulatory T cell responses stabilise atherosclerotic plaques and are defective in ACS

Inflammation plays a central role in the pathophysiology of ACS. Circulating levels of C-reactive protein (CRP) are elevated in ACS patients and are associated with adverse outcome[5]. White blood cells display increased inflammation and activation[6,7]. The culprit coronary plaque is highly inflammatory too[8] and the prevalence of multiple coronary inflammatory plaques is significantly higher in patients with ACS compared to stable coronary patients[7]. Recent experimental studies also suggest an inflammation-driven acceleration of the development of atherosclerotic lesions following experimental myocardial necrosis[9]. Genetic studies imply that both innate and adaptive immune responses triggered by various antigens, including (modified) lipoproteins, contribute to plaque development, progression and disruption[10,11]. Besides mononuclear cell and neutrophil activation in the circulation, several studies reported a perturbation of the T cell repertoire in ACS patients[12] with expansion of an effector and activated T cell subset[13] which is at least in part directed to antigens contained in the disrupted plaque(s)[14]. The previous finding that regulatory T cell (Treg)-mediated immunity tames experimental atherosclerosis and reduces plaque inflammation in mice[13] boosted experimental and translational research into the role of Treg cells in cardiovascular diseases. A particularly interesting aspect of the T cell response in ACS patients is the presence of an imbalance between T effector and Treg cells. In contrast to the effector T cell compartment activation, the percentage and/or function of circulating Tregs appear to be significantly decreased in the setting of ACS[15-18]. Whether this is related to a global Treg defect in these patients, to a defective mobilisation, increased susceptibility to cell death[19] or increased recruitment of Treg cells into sites of unstable coronary arteries is still unknown. Nevertheless, the results strongly suggest an imbalance of the adaptive immune response with potentially important consequences on the progression and destabilisation of coronary plaques. Interestingly, in 700 patients from the Malmö Diet and Cancer Study, low levels of circulating baseline CD4+Foxp3+ Treg cells were associated with an increased risk for the development of future acute coronary events[20], suggesting that defects in Treg-mediated immunity may predispose individuals to increased plaque vulnerability.

6.1.2 Tregs improve heart remodelling after myocardial ischaemic injury

Treg cells not only regulate antigen-specific immunity but also dampen innate immune responses in the local microenvironment through bystander immune suppression, suggesting that they can be involved in modulating the response to post-ischaemic injury. Furthermore, the ischaemic and necrotic myocardial tissue may expose self-

antigens for recognition by the immune system, which may lead to antigen-specific (autoimmune) adaptive responses[21,22]. Recent studies indicate that CD4+ T cells, and more particularly Treg cells, are important for the control of post-ischemic immune responses and the promotion of myocardial healing[21,23-25]. Inhibition of Treg recruitment to the site of myocardial injury resulted in excessive post-ischaemic inflammation, matrix degradation and adverse remodelling[23]. In contrast, in vivo expansion of Treg cells or their therapeutic activation by superagonistic anti-CD28 mAbs attenuated left ventricular remodelling and improved cardiac function[24,25]. Altogether, the studies point to a protective role for Treg immunity in coronary artery disease, limiting atherosclerotic plaque development and vulnerability, and taming the deleterious consequences of post-ischaemic injury. We therefore hypothesise that expansion of Treg cells in patients with ACS dampens the activation of the immune response and promotes both plaque and myocardial healing.

6.1.3 Treg cell expansion in ACS patients

Interestingly, a few therapeutic strategies, which are known for their athero- and cardio-protective effects in patients with ACS, have been shown to promote Treg-dependent immuno-regulatory responses both in experimental and clinical settings. This is the case for statins[26,27] and ACE inhibitors[28]. However, these therapies do not appear to be sufficient enough to promote full recovery of Treg levels and functions in patients with ACS, suggesting the need for alternative strategies. Direct supplementation with exogenous polyclonal Treg cells is not an option, particularly in the acute setting. Hence, there is a need for a simple, feasible and highly effective strategy to promote Treg cells at the acute phase of plaque destabilisation and myocardial infarction. We hypothesise that this can be achieved through subcutaneous administration of low doses of interleukin-2 (IL-2).

6.1.4 Low dose IL-2 and Treg cell expansion

IL-2 supplementation appears to be an attractive therapeutic option for several reasons. IL-2 plays a key role in Treg cell development, expansion, survival and suppressive function[29,30]. Deficiency of IL-2 or IL-2 receptor in mice greatly compromises Treg development and promotes autoimmune responses[31]. Supplementation of mice prone to atherosclerosis with IL-2 substantially increases Treg levels and significantly limits plaque development and inflammation[32,33]. Treg cells show a much lower threshold response to IL-2 receptor signalling compared to effector T cells. This led to the hypothesis that, in contrast to high dose IL-2 designed to activate T effector cells in cancer, supplementation with low doses of IL-2 in the setting of T cell-mediated immune diseases may selectively promote the expansion of Treg cells at the expense of T effector cells, thereby limiting harmful immune responses. Remarkably, this hypothesis was recently confirmed in a few human clinical studies in the setting of graft-versus-host disease[34,35], hepatitis C virus-induced vasculitis[36], or systemic lupus erythematosus[20]. In those studies, administration of low doses of IL-2 (daily administration of $0.3x10^6$ to $3x10^6$ IU IL-2 per square meter of body-surface area for 8 weeks, repetitive 5-day courses of $1x10^6$ to $3x10^6$ IU IL-2, or 3 cycles of $1X10^6$ IU IL-2 every other day for 2 weeks followed by a 2-week break in treatment) led to a rapid and marked expansion of the circulating pool of Treg cells, which were increased by a factor of 2 to 20 without affecting the pool of conventional CD4+ T (i.e. T effector) cells. The expanded Tregs retained potent suppressive functions and the treatment was associated with a reduction in the inflammatory response and a concomitant clinical improvement in a substantial proportion of patients. Treatment with low dose IL-2 was safe and no adverse effects were reported. This strategy is currently being adapted and tested in various disease settings, where Treg cell promotion is believed to be of potential therapeutic benefit[35-37].

6.2 Aldesleukin

Aldesleukin is commercially available and is licensed for the treatment of metastatic renal cell carcinoma in the UK. A single vial unit contains 22 x 10^6 IU aldesleukin. Aldesleukin is produced by recombinant DNA technology using an *Escherichia coli* strain which contains a genetically engineered modification of the human IL-2 gene. The administration of aldesleukin is by either intravenous or subcutaneous routes. Following short intravenous infusion, its pharmacokinetic profile is typified by high plasma concentrations, rapid distribution into the extravascular space and a rapid renal clearance. The recommended doses for continuous infusion and subcutaneous injection (as detailed in the SmPC) are repeated cycles of 18 x 10^6 IU per m^2 per 24-hours for 5 days and repeated doses of 18 x 10^6 IU respectively. Peak plasma levels are reached in 2-6 hours after subcutaneous administration, with bioavailability of aldesleukin ranging between 31 – 47%. The process of absorption and elimination of subcutaneous aldesleukin is described by a one-compartment model, with a 45-minute absorption half-life and a 3-5 hour elimination half-life. For the purposes of this trial, (see section 6.1.3), the drug will be administered to the patients by subcutaneous injection.

6.3 Clinical use of IL-2

The use of IL-2 in low doses as a means of expanding Treg cell populations in autoimmune and allo-inflammatory conditions has been explored. ClinicalTrials.gov currently lists 154 clinical studies using low-dose IL-2 (Figure 3). There have been several completed and published human clinical trials on low dose IL-2 therapy in autoimmune and allo-immune diseases. A few examples are shown in Table 1[38]. In these studies, patients have received at least 1 dose of IL-2 ranging from $0.3x10^6$ IU – $3.0x10^6$ IU. Interestingly, there was a low rate of adverse events (AEs) in all of the studies with the most commonly reported AEs being injection site reactions, fatigue, fever, nausea and vomiting. A low percentage of serious adverse events (SAEs) were recorded in a GVHD-risk study and these SAEs included haemorrhage (CNS), anorexia, and infection (colitis).

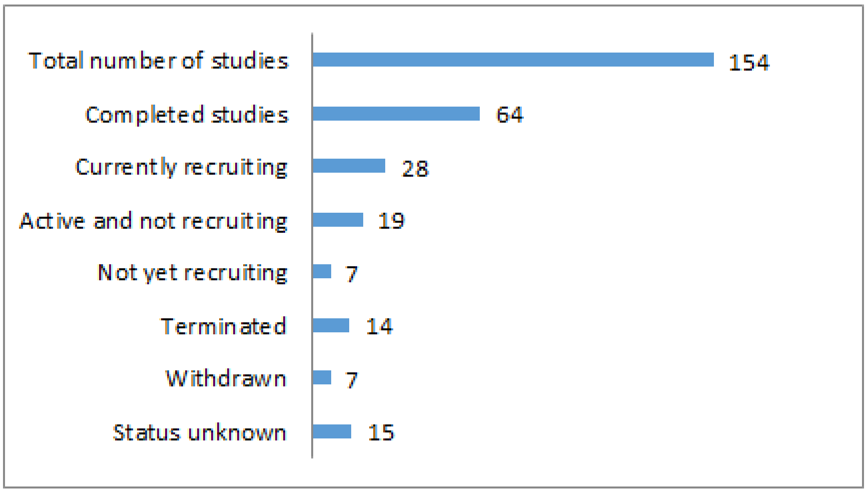

Figure 3: Listed low-dose IL-2 studies on clinicaltrials.gov

**Trial** (*reference*)	**Disease**	**N**	**IL-2 dose (daily, MIU)**	**Course**	**Total IL2 (MIU)** (days)	**Main biological findings**	**Clinical findings**
**Low-dose interleukin-2 treatment selectively modulates CD4(+) T cell subsets in patients with systemic lupus erythematosus**. *He, J; Zhang, I et al. Nat Med 2016, Sep;22(9):991-3. Doi: 10.1038/nm.4148.*	SLE	38	1	3 cycles of: Single dose every other day for 2 weeks, 2 weeks break.	**21** (21)	Increase of Tregs, THF and TH17, but not TH1.	Marked reduction of disease activity in SLE patients.
**Evaluation of clinical efficacy and immunological responses after IL-2 therapy in HCV-related vasculitis patients** *Saadoun, D. et al. Regulatory T-cell responses to low-dose interleukin-2 in HCV-induced vasculitis. N Engl J Med 365, 2067–77 (2011)*	Hepatitis C Virus induced vasculitis	10	1.5 then 3	Four 5-day courses	**52.5** (60)	Increase of CD4+Tregs (x3) and CD8+ Tregs (x8). Increase of NK and cd56 bright NKs; Decrease of B cells.	Grade 1 & 2 AEs; no vasculitis or HCV-replication flares; improvement of the vasculitis in 8/10 patients.
**Dose-effect relationship of low-dose IL-2 in type 1 diabetes** *Hartemann, A. et al. Low-dose interleukin 2 in patients with type 1 diabetes: a phase 1/2 randomised, double-blind, placebo-controlled trial. Lancet Diabetes Endocrinol 1, 295–305 (2013). Rosenzwajg, M. et al. Low-dose interleukin-2 fosters a dose-dependent regulatory T cell tuned milieu in T1D patients. J. Autoimmun. (2015). doi:10.1016/j.jaut.2015.01.001*	Type 1 Diabetes	24	0.3,1,3	One 5-day course	**1.5,5,15** (15)	Dose-dependent increase of CD4+ Tregs and CD8+ Tregs; Dose dependent decrease of B cells; No effects on NK or Teffs. Imprinting of a dose-dependent regulatory-tuned milieu.	Good tolerance; Grade 1 and 2 AEs; more days with AEs in the placebo group than in any other group.
**Dose Finding Study of IL-2 at Ultra-low Dose in Children with Recently Diagnosed Type 1 Diabetes** *NCT01862120*	Type 1 Diabetes	24	0.25,0.5,1	One 5-day course followed by 1 injection every 2 weeks	**7.5,15,30** (365)	Double blind, still in progress.	Good long-term tolerance on the first 18 patients treated for at least 6 months.
**Effects of Low-dose Recombinant IL-2 to Promote Treg in Alopecia Areata** *Castela, E. et al. Effects of low-dose recombinant interleukin 2 to promote T-regulatory cells in alopecia areata. JAMA Dermatol 150, 748–51 (2014).*	Alopecia Aerata	5	1.5 then 3	Four 5-day courses	**52.5** (60)	Increase of Tregs and decrease of CD8+ Teffs in scalp biopsies during and after IL-2 treatment	Regrowth of body or scalp hair in all 5 patients, of scalp hair in 4.
**Induction of Regulatory T Cells by Low Dose IL-2 in Autoimmune and inflammatory Diseases** *Rosenzwajg, M. et al. Immunological and clinical effects of low-dose interleukin-2 across 11 autoimmune diseases in a single, open clinical trial. Ann Rheum Dis 2018, Nov 24. Doi: 10.1136/annrheumdis-2018-214229. [Epub ahead of print]. NCT01988506*	Moderate forms of **Autoimmune and inflammatory diseases	46	1	One 5-day course, followed by 1 injection every 2 weeks for 6 months	**18** (182)	Increase in CD4+ Tregs. No significant increase in Teffs.	Well tolerated in all diseases. Indication of potential clinical efficacy.
**Low-dose IL-2 therapy in one SLE patient refractory to standard therapies** *Humrich, J. Y. et al. Rapid induction of clinical remission by low-dose interleukin-2 in a patient with refractory SLE. Ann. Rheum. Dis. (2015). doi:10.1136/annrheumdis-2014-206506*	Severe SLE	1	1.5 then 3	Four 5-day courses	**52.6** (60)	Treg increase; decreased of anti-dsDNA antibody levels.	Well tolerated; Major clinical improvement

**Low-dose interleukine-2 in active systemic lupus erythematosus** *David Klatzmann & Abul K. Abbas Nature Reviews Immunology Volume: 15, Pages: 283–294 (2015) doi:10.1038/nri3823 (Personal communication with Di Yu and Zhanguo Li)*	SLE	40	1	3 courses of daily injections every other day for 2 weeks	**21** (90)	Treg increase (x2); decreased of anti-dsDNA antibody levels.	Well tolerated; Major clinical improvement in 36/40 patients who showed both clinical and serological remission
**Ultra-Low dose IL-2 for Refractory Chronic Graft Versus Host Disease** *Koreth, J. et al. Interleukin-2 and regulatory T cells in graft-versus-host disease. N Engl J Med 365, 2055–66 (2011). Matsuoka, K. et al. Low-Dose Interleukin-2 Therapy Restores Regulatory T Cell Homeostasis in Patients with Chronic Graft-Versus-Host Disease. Sci. Transl. Med. 5, 179ra43 (2013).*	cGVHD	23	0.3,1,3/m^2 (0.54 to 5.4)	Daily administration for 8 weeks, (4 weeks hiatus, follow-up administration for responders)	**32 to 320** (56 to 365)	CD4 Treg increase (x8); NK cell increase (x2)Asymptomatic peripheral-blood eosinophilia	Grade-3 and -4 AEs; 12 partial response; Tapering of corticosteroids by 60% in responders
**Ultra Low-Dose IL-2 for GVHD Prophylaxis** *Kennedy-Nasser, A. A. et al. Ultra low-dose IL-2 for GVHD prophylaxis after allogeneic hematopoietic stem cell transplantation mediates expansion of regulatory T cells without diminishing antiviral and antileukemic activity. Clin Cancer Res 20, 2215–25 (2014). NCT00539695*	Prevention of GVHD	16	0.1 and 0.2/m^2 (0.18 to 0.36)	3 times per week for 6-12 weeks	**3.3 to 13** (42 to 84)	Expansion of Tregs (x2); No expansion of CD8+ memory Teffs or NK cells	No grade-3 and -4 GVHD; Less infections than in control group
**Ultra-low Dose IL-2 in Healthy Volunteers** *Ito, S. et al. Ultra-low dose interleukin-2 promotes immune-modulating function of regulatory T cells and natural killer cells in healthy volunteers. Mol. Ther. J. Am. Soc. Gene Ther. 22, 1388–1395 (2014).*	Healthy volunteers	21	0.05,0.1,0.2/m^2 (0.09 to 0.36)	One 5-day course	**0.45 to 1.8** (5)	Expansion of Helios+ and Helios– Tregs; Dose dependent increase of CD56 bright NKs; Increase in serum IP10; No increase in IL2, IFNg, IL10, IL115, IL17	Well tolerated<br>All grade-1 AEs, except for 1 grade-2 injection site reaction
**Ultra-Low dose IL-2 for Refractory Chronic Graft Versus Host Disease** *Koreth, J et al. Efficacy, durability and response predictors of low-dose interleukin-2 therapy for chronic graft-versus-host disease. Blood 128, 130-137 (2016). NTC00529035*	cGVHD	33	1	Daily administration for 12 weeks (4 week hiatus, follow-up administration for responders)	**84**	Rapid expansion of Treg and NK cells with peak after 4 week, then plateau.	Low relapse and secondary malignancy rates.

Table 1: Examples of published findings from low dose IL-2 clinical trials
(MIU: million international units, **: Rheumatoid arthritis, Ankylosing spondylitis, SLE, Psoriasis, Behcet's Disease, Wegener's granulomatosis, Takayasu's disease, Crohn's Disease, Ulcerative colitis, Autoimmune hepatitis and Sclerosing cholangitis)

7 Rationale for Trial

The experimental and clinical background in low-dose IL-2 therapy suggests a potential clinical utility of Treg cell expansion in patients with ACS. Administration of low doses of IL-2 in various clinical settings appears to be safe and remarkably efficacious at promoting selective expansion of Treg cells with preserved suppressive function. Circulating Tregs are reduced at admission for ACS and during the first 8 weeks after the index event, but recover to normal levels thereafter[15]. Quick re-establishment and maintenance of 'normal' Treg levels during that phase is paramount. Indeed we speculate that increasing Treg levels to above normal may have beneficial effects on myocardial repair and decrease atherosclerotic progression.

Low dose interleukin-2 in patients with stable ischaemic heart disease and acute coronary syndromes (LILACS) was a, single centre (with an associated shared care site) study conducted in Cambridge. It was a Phase 1/2a double blind, placebo controlled clinical trial (MHRA reference 24551/0029/001-0001; REC reference: 17/NW/0012), where, for the first time, we assessed the safety and biological efficacy of low dose IL-2 therapy in patients with stable ischaemic heart disease and ACS (where the drug is currently contraindicated). For Part A, we dosed 25 patients with stable ischaemic heart disease in 5 escalating dose groups, with doses ranging from 0.3 to $3x10^6$ IU/daily for 5 consecutive days (Table 2). In Part B, we dosed 16 patients who presented with a non-ST elevation acute coronary syndrome (similar to this IVORY trial) in 2 dose escalating groups for 5 consecutive days.

**LILACS Trial Part (A or B)**	**Part A**					**Part B**	
**Group**	Group 1	Group 2	Group 3	Group 4	Group 5	Group 1	Group 2
**Dose**	0.3 $x10^6$IU	0.6 $x10^6$IU	1.2 $x10^6$IU	2.4 $x10^6$IU	3.0 $x10^6$IU	1.5 $x10^6$IU	2.5 $x10^6$IU
**Number of subjects dosed per group**	5	5	5	5	5	8	8
**Ratio of IMP to Placebo (IMP : Placebo)**	3:2	3:2	3:2	3:2	3:2	3:1	3:1

Table 2: LILACS dosing schedule

<u>Serious Adverse Events</u>

There were no serious adverse events (SAEs) in Part A of LILACS.

There were 2 SAEs in LILACS Part B which recruited patients with unstable angina and NSTEMI. The first one was after consent but prior to administration of IMP and therefore not related. The second event was a patient admitted with a non-ST elevation myocardial infarction (NSTEMI). A pre-dose coronary angiogram demonstrated severe three vessel coronary artery disease requiring urgent inpatient coronary artery bypass surgery. This patient was dosed (with either Aldesleukin 2.5 x 10^6 IU/Placebo) whilst awaiting inpatient transfer for cardiac surgery. The patient had transient episodes of chest pain prior to dosing and on the last day of dosing. In the case of the latter

episode of chest pain, no ECG changes were noted, however a rise in troponin suggested further myocardial ischaemia. This was deemed a significant medical event and hence an SAE. There was no clinical consequence of the event, no additional treatment was needed, and patient went on to their planned interventional surgery without complication. This SAE was classed as unrelated to the IMP due to the patient's severe underlying condition for which he was awaiting bypass surgery. Both SAEs have resolved.

AE description	Frequency	Severity	Relatedness
Abdominal pain	1	severe	unrelated
Raised troponin	1	severe	unrelated

Table 3: LILACS symptoms and signs documented as SAEs

Non-Serious Adverse Events

In 41 patients (205 injections in total) in Parts A (stable IHD) and B (ACS patients) of the LILACS trial, 93 injection site reactions in the form of localised skin reactions have been observed. Typically, a patient received 5 injections over the course of the trial and as such more than 1 ISR was observed in a single patient in some instances. The lesions have been limited to a diameter of 3-4 cm and have been self-limiting with full resolution without sequelae within a week. This is a known reaction to subcutaneous IL-2 and can also be attributable to dextrose 5% solution which is the diluent for the IMPs.

Other adverse reactions reported in the LILACS trial included flu like syndrome, fatigue, body ache and nasal congestion. These are possibly related to a systemic reaction to IL-2 and usually occur a few hours after injection of the IMP and can occur together. The symptoms usually resolve overnight and do not interfere with activities of daily living. There are no long lasting sequelae. Other AEs are infrequent.Appendix 1 lists the AEs and their causality as assessed by the blinded investigator.

The primary efficacy outcome for LILACS is to determine the ability of low dose IL-2 to increase circulating levels of Tregs by greater than >75%. When examining aggregated data, this was achieved at doses of between $1.5x10^6$ IU and $3.0x10^6$ IU daily in stable IHD and in ACS patients. In both groups, we did not see an increase in T effector cells.

An analysis of unlocked and blinded (stats unblinded) Treg and safety data for LILACS Part B was carried out. The safety data were deemed acceptable as there were no drug related SAEs as assessed by the investigator. In group B1 (1.5×10^6 IU), an 80% increase in Tregs from baseline was observed between visit 2 and visit 7. The dose 2.5×10^6 IU in group B2 led to an increase in Tregs of 115% between visit 2 and visit 7. At the same time no significant increase in T effector cells was noted.

Based on these emerging data we are confident in the safety and biological efficacy of low dose IL-2 to significantly increase Tregs, and therefore wish to extend its use in ACS patients and further assess its clinical efficacy with repeated doses of 1.5×10^6 IU beyond the induction phase as tested in the LILACS trial.

The aim of the IVORY trial is to assess the efficacy of repeated low doses of IL-2 in reducing vascular inflammation, as assessed by ^{18}F-FDG PET/CT, in patients presenting with ACS.

The planned doses will be given to the trial patients as subcutaneous injections once a day, over five consecutive days for an initial induction phase, and additionally the same

dose once every 7 days for a further 7 doses during a maintenance phase – resulting in a total of 12 doses per patient (*1:1 randomisation of low dose IL-2 vs. placebo).* On the basis of safety and tolerability data from published clinical studies (Table 1), and from our own unpublished safety and tolerability data from the LILACS trial as described above, the dose to be used in this trial will be 1.5×10^6 IU for induction and maintenance phases.

^{18}F-FDG PET/CT has been widely used as a biomarker for drug development in phase II studies[39,40]. ^{18}F-FDG PET/CT has been chosen as the imaging modality because in atherosclerosis, vascular ^{18}F-FDG uptake correlates with immune cell infiltration and glucose consumption[41]. It has been used by ourselves and collaborators worldwide to assess the effect of statins and anti-inflammatory compounds on vascular inflammation[42,43] and predicts subsequent cardiovascular events[44,45].

7.1 Hypotheses

Hypotheses: Treatment of patients with ACS using low-dose IL-2 will reduce vascular inflammation as assessed by ^{18}F-FDG-PET/CT.

A priori hypothesis: Patients presenting with high sensitivity C-reactive protein (hs-CRP) levels >2mg/l have residual systemic inflammation and are at high risk of recurrent CV events. We believe those patients are most likely to benefit from a treatment with low-dose IL-2, which is expected to reduce CV events through the suppression of inflammation. Thus, we will restrict recruitment in this trial to patients with ACS and hsCRP>2mg/l.

8 Trial Design

8.1 Statement of Design

This is a randomised, double-blind, placebo controlled, parallel group experimental medicine trial. The aim of the trial is to test the superiority of low dose IL-2 compared to placebo in reducing vascular inflammation in ACS patients with hs-CRP>2mg/l.

8.2 Number of Centres

There will be 2 hospital sites in Cambridge taking part. Addenbrooke's Hospital, Cambridge, will be the main trial site and Royal Papworth Hospital (which is now situated on the Cambridge Biomedical Campus) will be a shared care site where patients may be recruited and/or have study visits.

8.3 Number of Patients

A sufficient number of patients will be enrolled so that at least 60 patients with data suitable for the primary statistical analysis (approximately 30 per arm) complete the trial. To achieve this, we estimate that we will need to recruit approximately 90-100 patients to account for the percentage of ACS patients with hsCRP>2mg/l (approximately 65% of ACS patients) and potential dropouts/unanalysable data. However, we will continue to recruit patients until target patient completion is achieved.

8.4 Trial Duration

The total trial duration for each patient will be approximately 13 weeks. This will include 1 week for recruitment and screening, 8 weeks of treatment and a follow up period of approximately 4 weeks after the last treatment visit. The follow-up PET/CT will be scheduled 1 week after the last treatment visit.

8.5 Trial Objectives

8.5.1 Primary objective

- To compare the effect of low dose IL-2 against placebo on vascular inflammation using ^{18}F-FDG PET/CT in ACS patients.

8.5.2 Secondary objectives

- To determine if low dose IL-2 can increase Treg cell numbers sustainably over extended treatment
- To determine the effect of low dose IL-2 on systemic inflammation measured by cardiovascular biomarkers (including but not limited to hsCRP, IL-6, BNP, Troponin I).
- To determine the safety and tolerability of extended dosing of low dose IL-2 in patients with an acute coronary syndrome

8.5.3 Exploratory objectives

- To determine the impact of low dose IL-2 on peripheral blood mononuclear cell subsets and Treg function
- To determine the effect of low dose IL-2 on left ventricular systolic function.

8.6 Trial endpoints

8.6.1 Primary endpoint:

Change in vascular inflammation (as measured by mean TBR max in the index vessel) on ^{18}F-FDG PET/CT from baseline to follow up scans.

(In detail, a region of interest (ROI) including arterial wall and lumen will be drawn on each axial slice of artery (ascending aorta and both carotid arteries) on the co-registered PET/CT scan and the maximum standardised uptake value (SUVmax) recorded. Subsequently, each ROI will be normalised by the blood FDG concentration in the superior vena cava or jugular vein (for carotids), to yield an arterial mean maximum tissue-to-blood ratio (TBR max) as a quantitative measure of arterial tracer uptake. The "index vessel" (defined as the arterial territory with the highest mean max TBR at baseline – left carotid, right carotid or ascending aorta) will be the primary outcome variable.

All scans will be analysed by an experienced reader, anonymised to patient identifiable information (name, treatment group, and visit number.)

8.6.2 Secondary endpoints:

1. Change in mean max TBR in each arterial region individually restricted to those slices with TBR>1.6 (as per previous publications[39])

2. Change in lymphocyte subsets (T effector (Teffs) cells, defined as central memory and effector memory T cells in the non-Treg gated T cells), Natural Killer (NK) cells (CD3−CD56+CD16+CD19−) and B lymphocytes (CD19+CD4−)) between low dose IL-2 and placebo throughout the treatment period will be evaluated by flow cytometry.
3. Change in percentage of Treg cells (defined as $CD3^{+}CD4^{+}CD25^{high}CD127^{low}$ cells within the $CD3^{+}CD4^{+}$ T cell gate) between low dose IL-2 and placebo throughout the treatment period will be evaluated by flow cytometry.
4. The change in serum cardiac biomarkers will be evaluated by blood sample analysis.
5. The safety and tolerability of extended dosing of IL-2 in ACS patients will be evaluated by:
    - Adverse events
    - Further cardiovascular events
    - Concomitant medications
    - Physical examination defined as examination of the cardiovascular, respiratory, gastrointestinal, limited skin and brief neurological examinations
    - Examination of injection site reactions
    - Vital observations which include blood pressure, heart rate, temperature, respiratory rate and oxygen saturation
    - Safety clinical blood tests (defined in section 11.6)
    - Thyroid function blood test (defined in section 11.6)
    - 12-lead electrocardiogram (ECGs) recordings

8.6.3 Exploratory endpoints:

1. Change in phenotype and function of peripheral blood mononuclear cell (PBMC) subsets as assessed by flow cytometry, gene expression, and in vitro activation and suppression assays.

2. Change in ejection fraction as measured on transthoracic echocardiograms

9 Selection and withdrawal of patients

9.1 Inclusion Criteria

To be included in the trial the patient must meet the following criteria:

- Able to provide written informed consent to participate
- Aged between 18 and 85
- Current admission (on the screening visit) with an acute coronary syndrome - ST elevation myocardial infarction (STEMI), non-ST elevation myocardial infarction (NSTEMI), or unstable angina (UA) with symptoms suggestive of myocardial ischaemia lasting 10 minutes or longer with the patient at rest or with minimal effort

  AND EITHER

  i. elevated levels of TnI on admission

  OR

  ii. dynamic changes in ECG (new ST-T changes or T-wave inversion).

- Where applicable, to be included in the trial women must be

i) Postmenopausal (for the purposes of this trial, postmenopausal is defined as being amenorrhoeic for greater than 2 years with an appropriate clinical profile, e.g. age appropriate, history of vasomotor symptoms)

1. OR

ii) Have had a documented hysterectomy and/or bilateral oophorectomy or sterilised

1. OR

iii) Peri-menopausal with a negative pregnancy test at screening (for the purposes of inclusion in this trial. Peri-menopausal is defined as women with an appropriate clinical profile, e.g. age appropriate, history of vasomotor symptoms, irregular periods). They will also have to comply with the use of contraception for the duration of the trial and undergo additional pregnancy tests during and after treatment.

- High sensitivity C-reactive protein of >2 mg/L at screening
- Willingness and possibility to start dosing within 8 days from initial date of admission to the primary hospital for ACS
- Able to comply with all trial mandated visits.

9.2 Exclusion Criteria

The presence of any of the following will preclude patient inclusion:

- Current presentation (at screening) with cardiogenic shock (systolic blood pressure <80 mm Hg, unresponsive to fluids, or necessitating catecholamines).
- Current presentation with cardiac arrest
- Signs or symptoms of active infection requiring intravenous antibiotic treatment at screening
- History of malignancies requiring active treatment (However, patients with a history of treated localised basal or squamous cell skin cancer are not excluded from participation in this trial)
- History of solid organ transplantation or other bone marrow transplantation
- History of recurrent epileptic seizures in the previous 4 years; repetitive or difficult to control seizures, coma or toxic psychosis lasting >48 hours
- Uncontrolled hypotension (Systolic BP (SBP)<80mmHg or DBP<50mmHg) OR uncontrolled hypertension (SBP>180 or DBP>120 mmHg) at screening
- Average corrected QT interval (QTc) > 450 msecs using Bazett's formula from average of triplicate ECGs (or > 480 msecs if bundle branch block)
- Renal impairment defined as Creatinine clearance [Cockcroft-Gault] <45ml/min at screening
- Liver dysfunction (defined as ALT > 2xULN) at screening
- Evidence of cholestasis defined as elevated Total Bilirubin Levels, (TBL > 1.5 x ULN) and Alkaline Phosphatase, ALP (ALP > 1.5 x ULN), at screening
- Known hypothyroidism or hyperthyroidism
- Known autoimmune disease requiring active immunosuppressive treatment
- Any oral or intravenous immunosuppressive treatment including regular prednisolone, hydrocortisone or disease modifying drugs. [Inhaled or topical steroids are permissible]

- Patients on cytotoxic drugs and interferon-alpha
- Known Type 1 or Type 2 diabetes mellitus
- Contraindication to IL-2 treatment or hypersensitivity to IL-2 or to any of its excipients
- Participation in a previous research trial in the last 3 years which involved exposure to significant ionising radiation (i.e. cumulative research radiation dose >5 mSv)
- Participation in a clinical trial where the patient has received a drug or new chemical entity within 30 days or 5 half-lives, or twice the duration of the biological effect of the drug (whichever is longer) prior to the first dose of trial medication, Visit 3 (Day 1).
- Any medical history or clinically relevant abnormality that is deemed by the principal investigator/delegate to make the patient ineligible for inclusion because of a safety concern
- Pregnant women or breast feeding women

9.3 Treatment Assignment and Randomisation Number

A sufficient number of patients will be randomised so that approximately 60 patients will complete the trial. Stratification at randomisation based on an ECG based ST-elevation status (to define non-STEMI or STEMI status) will be utilised to balance the groups. Patients will be randomised in a 1:1 fashion to either low dose interleukin-2 or placebo using an online randomisation system (Sealed Envelope).

9.4 Method of Blinding

The trial will be double-blind, with active and placebo doses appearing identical at point of issue and administration. The CUH central pharmacy will be unblinded and provided with a copy of the concealment list. Data analysis for the trial will be performed by a statistician who will be unblinded after the database lock.
The statistician, or delegate, may be unblinded for individual patients after their treatment period has concluded, to facilitate rapid reporting of safety events to the IDMC.

9.5 Patient Withdrawal Criteria

Withdrawn patients may be replaced by recruiting and randomising new patients. Reasons for patient withdrawal will be recorded in the Case Report form (CRF). Reasons for withdrawal may include: adverse event, SAE, SUSAR, withdrawal of consent, lost to follow-up, protocol deviation, patient non-compliance or the trial closing/terminating.

Specific organ based withdrawal criteria are set out below:

9.5.1 <u>Liver withdrawal criteria</u>

Liver chemistry threshold stopping criteria have been designed to assure subject safety and to evaluate liver event aetiology.

Patients will be withdrawn if any of the following liver chemistry stopping criteria are met:

1. ALT ≥ 3xULN and total bilirubin ≥ 2xULN
2. ALT ≥ 5xULN.
3. ALT ≥ 3xULN if associated with symptoms (new or worsening) believed to be related to hepatitis (such as fatigue, nausea, vomiting, right upper quadrant pain or tenderness or jaundice) or hypersensitivity (such as fever, rash or eosinophilia).
4. Isolated ALT ≥ 3xULN persists for ≥ 4 weeks.

9.5.2 Renal withdrawal criteria

Serum creatinine elevation > 2 x baseline screening visit (V1)

9.5.3 Cardiac withdrawal criteria

These assessments will be carried out during patient screening and patients that meet the criteria below will be withdrawn from the trial. The Bazett's QT correction formula should be used to determine inclusion and discontinuation for any individual patient throughout the trial.

- QTcB >500msec OR
- Change from baseline: QTcB >60msec

If a patient has underlying bundle branch block the following withdrawal criteria should be used instead.

Baseline QTcB value (with underlying bundle branch block)	QTcB withdrawal criteria
<450msec	>500msec
450-480msec	>530msec

Withdrawal of patients is to be based on an average QTcB value of triplicate ECGs. If an ECG demonstrates a prolonged QT interval, obtain 2 more ECGs over a brief period (approximately 5 minutes) and then use the averaged QTcB values of the 3 ECGs to determine whether the patient should be discontinued from the trial.

Patients who require CABG (either inpatient or outpatient) during the scheduled dosing period should be withdrawn from the study.

Patients who develop new onset severe pulmonary oedema/new severe congestive heart failure, requiring high dose > 240 mg over 24 hours IV furosemide during admission will also be withdrawn from the trial.

Patients with symptomatic uncontrolled systolic BP < 80 mmHg and/or diastolic BP < 50 mmHg (after at least 2 repeat recordings), or severe hypertension (as defined by BP > 180 mmHg systolic BP or >120 mmHg diastolic BP on at least two readings) will also be withdrawn.

Transient atrial fibrillation is not a reason for withdrawal in this population.
Patients with sustained (>30 sec or symptomatic) ventricular tachycardia and patients with ventricular fibrillation will be withdrawn. Non-sustained ventricular tachycardia is a common occurrence in this population, and is not a reason for withdrawal in this population.

9.5.4 General withdrawal criteria

- Cardiorespiratory arrest
- Failure to attend 2 scheduled dosing appointments without adequate reason based on the PI assessment
- Severe hypersensitivity reactions will preclude any further IMP administration
- New seizure activity
- Coma
- Severe lethargy or somnolence
- Respiratory insufficiency requiring intubation
- Pregnancy
- Withdrawal of consent
- PI discretion
- Any serious adverse reaction (SAR) or adverse reaction which is deemed by investigators as Severe (AR).
- Any significant incidental finding on PET/CT scan clinical governance reports, which in the opinion of the PI, necessitates further investigation and management.
- Any medical history, clinically relevant abnormality or reason that is deemed by the principal investigator (PI) to make the patient ineligibile to continue the trial.

9.5.5 Management of withdrawal

- Withdrawn patients may be replaced with newly recruited patients to maintain the power of the study
- Withdrawn patients will be followed up as per the PI discretion depending on the clinical scenario
- Withdrawn patients will not be subjected to additional research related tests (e.g. blood tests for research purposes, PET/CT scans) from the date of withdrawal but may have safety related tests (e.g. blood tests, ECGs) at the PI's discretion on clinical grounds
- All blood samples or imaging data collected prior to the date of withdrawal may be analysed.

9.6 Trial stopping criteria

For the first 10 patients dosed, if a cumulative total of three events are observed, the trial conduct will be halted (recruitment and dosing of all active patients). These events include serious adverse reactions (SARs) defined as possibly, probably or definitely related to the trial drug or severe adverse reactions (ARs). This will trigger an unscheduled independent DMC meeting to convene to review the trial data to date. Once the trial has halted for the aforementioned reasons, it may only resume after approval of a substantial amendment to the Regulatory Authorities that have approved the trial.

After the tenth (10th) patient has commenced dosing, a percentage basis (30%) of patients who experience pre-specified events (SARs defined as possibly, probably or definitely related to the trial drug or severe adverse reactions) will be used to halt the trial and review the data by the IDMC. Once the trial has halted for the aforementioned reasons, it may only resume after approval of a substantial amendment to the Regulatory Authorities that have approved the trial.

10 Trial Treatments

10.1 Dosage schedules

10.1.1 Route of Administration and Maximum dosage allowed

Patients will be treated with daily subcutaneous injections of IMP in two different phases: induction and maintenance. The dose of IL-2 will be fixed and the same for all patients during all injections and has been defined as $1.5x10^6$ IU per injection based on data available from the LILACS trial.

During the induction phase, patients will be treated with IMP, given subcutaneously, once daily for five consecutive days. This will occur during visits V3-7. The induction phase lasts 5 days.

The maintenance phase will start 1 week after the induction phase. The first dose during this phase occurs 7 (+/-3) days after the last dose of the induction phase. The dose of IMP will be given subcutaneously once weekly (every 7th (+/-3) day) for 7 doses in total. This will occur during visits V8-14.

Therefore, in total, 12 IMP injections will be given across the induction and maintenance phases for each completed patient.

10.1.2 Maximum duration of treatment of a patient

The maximum treatment duration is 8 weeks.

10.2 Presentation of the drug

Active drug description
Commercially available aldesleukin with a UK marketing authorisation will be used and will be initially prepared as per SmPC, with further dilution as described below.

For this trial, the method described for aldesleukin dilution in an SmPC for Ceplene will be utilised, producing a final aldesleukin concentration of 3.3×10^6IU/ml (200µg/ml). Existing (unlicensed) data from several sources supports stability and sterility of reconstituted diluted IL-2 preparations (prepared as per SmPC and then further diluted with dextrose 5%) for up to 21 days at 2-8ºC when syringes are prepared by qualified health-care professionals under aseptic conditions.

Placebo description
Commercially available dextrose 5% injection with a UK marketing authorisation at equivalent dose volume will be used for the placebo formulation.

10.3 Known drug reactions & interaction with other therapies

Significant clinical interactions have been reported when high dose IL-2 (Aldesleukin) has been combined with cytotoxic drugs and interferon-alpha; therefore, patients on these drugs will not be permitted to participate in the trial, nor are these medications permitted during this trial.

10.4 Dosage modifications

This is a fixed dose trial and no dose modifications will be permitted. The dose selected for this trial is $1.5x10^6$ IU per injection and will be used for all patients for all visits.

10.5 Legal status of the drug

Aldesleukin is a commercially available Prescription Only Medicine and is licensed for the treatment of metastatic renal cell carcinoma in the UK.

Within this trial, aldesleukin (interleukin-2, IL-2) and matching placebo are classed as Investigational Medicinal Products.

10.6 Drug storage and supply

Commercial supply of IL-2, from hospital pharmacy stocks, will be used for this trial. Vials will be stored as per SmPC in a secure location with access restricted to appropriate individuals.

Commercial supply of dextrose 5% injection, from hospital pharmacy stocks, will be used for placebo, stored as per labelled instructions.

IMPs will be dispensed by the site pharmacy under aseptic conditions for this trial upon receipt of a suitably signed trial specific prescription.

10.7 Accountability

Records will be maintained by pharmacy to document safe receipt, handling, storage, dispensing and return of unused investigational medicinal products (IMPs) as appropriate.

10.8 Concomitant Therapy

The IMP administration based on data from previous IL-2 studies may result in fever, headache, migraine or diarrhoea, mostly in patients treated with doses higher than the doses to be used in this trial. Concomitant therapy with Paracetamol (up to 1g every 6 hours for 24 hours after IL-2 administration) can be instituted at the time of IL-2 administration to reduce fever. Pethidine may be added to control the rigors associated with fever. Anti-emetics (the use of 5-HT3 receptor antagonists, e.g. Ondansetron, and corticosteroids is not advised) and antidiarrheals may be used as needed to treat other gastrointestinal adverse reactions. Local reactions at injection site (erythema, pain,

pruritus) are the most common non-serious adverse events. Some patients with pruritic rash benefit from concomitant administration of antihistamines.

If a patient is admitted to hospital for any of the planned/elective investigations listed in section 12.3.2, concomitant medications pertaining to that hospital admission will not need to be recorded. All regular medications for home use as part of standard of care will be documented.

10.9 Emergency unblinding

It is the responsibility of the Chief/Principal investigator or sub-investigator to unblind the patient in the case of an adverse event, which in their judgement, requires knowledge of the trial medication received by the subject in order to provide appropriate treatment or management of the adverse event. The investigator or sub-investigator is also responsible for promptly documenting and explaining the unblinding to the Sponsor. Emergency unblinding will be undertaken by an authorised study team member using the online randomisation/unblinding system. As a back-up in the event of failure of the online system, arrangements within the trial pharmacy will be made for 24 hour access to emergency unblinding by providing the Kit Number that the patient was assigned at the point of patient randomisation.

11 Procedures and assessments

Trial assessments and procedures will be performed by suitably qualified and delegated trial personnel as described in the TPM. Informed consent must be obtained from the patient prior to any trial-specific procedures taking place.

11.1 Patient identification

ACS patients will be identified on medical wards at Addenbrooke's and Royal Papworth Hospitals by their treating clinical care team or the trial research team clinical staff. This may be achieved by reviewing inpatient medical notes or by discussion with clinical teams regarding their inpatients.
Permission to approach the patient will be sought by the trial team from the treating clinical care team. The patient will be approached by the usual clinical care team of the patient to determine if they may be interested in hearing about research. If the patient agrees, the trial team will approach the patient. (Rarely some members on the trial team may be on call and therefore be part of the clinical care team - in which case permission to approach the patient will be sought from the clinical consultant in charge of the care of the patient prior to the patient being approached).

Once contact has been made with the patient, the trial team will outline and explain the aims of the trial. A copy of the Patient Information Sheet together with a copy of the Mini Patient Information Sheet will be provided to interested patients, who will then be given the opportunity to consider the information with relatives and then to discuss the trial with trial staff and have any queries answered before consenting to participate in the trial.

11.2 Consent

The Informed Consent form must be approved by the REC and must be in compliance with GCP, local regulatory requirements and legal requirements. The investigator or designee must ensure that each trial patient is fully informed about the nature and objectives of the trial and possible risks associated with their participation.

The investigator or designee will obtain written informed consent from each patient before any trial-specific activity is performed. The informed consent form used for this trial and any change made during the course of this trial, must be prospectively approved by the REC. The investigator will retain the original of each patient signed informed consent form.

Should a patient require a verbal translation of the trial documentation by a locally approved interpreter/translator, it is the responsibility of the individual investigator to use locally approved translators.

Any new information which becomes available, which might affect the patient's willingness to continue participating in the trial will be communicated to the patient as soon as possible. This new information may be conveyed to the patient either over the phone, in writing, or in person at their next trial visit at the discretion of the PI depending on the nature of the new information.

11.3 Screening evaluation

11.3.1 Screening Assessments (**Visit 1**) Day -7 to 0

Trial specific assessments will only be conducted after patients have given written informed consent.

The screening visit can take place at suitably equipped wards or clinical research units at either Addenbrooke's or Royal Papworth Hospitals. The following procedures will be performed at this visit:

- Demography (date of birth, age, gender and race)
- Medical history
- Current medication history
- Physical examination (including cardiovascular, respiratory, gastrointestinal, neurological and skin examinations)
- Height and weight
- Vital observations (defined henceforth as temperature, blood pressure, heart rate, respiratory rate and oxygen saturations)
- 12-lead electrocardiogram (ECG) in triplicate with an average QTcB taken
- Transthoracic echocardiogram (TTE)
- Screening bloods (approximately 15mls) including:
    - Safety blood tests (defined as but not limited to: haematology (full blood count and differential), clinical biochemistry (electrolytes, urea, creatinine, liver function (ALT, ALP, albumin, bilirubin))
    - Thyroid function tests (TSH - V1 and V16 only [fT4 will only be performed if TSH is abnormal])
    - hsCRP
    - Serum pregnancy est where applicable
    - Full lipid profile

11.3.2 Patient Randomisation

All screening tests must be returned and checked by the trial doctor before randomisation. The PI or a delegate must sign the CRF to confirm eligibility after the screening process has been completed.

Randomisation will occur centrally at the main trial site, Addenbrooke's Hospital, and Addenbrooke's Hospital central pharmacy will prepare the IMP for all patients.

11.4 Trial assessments

All trial visits will be held at suitably equipped wards or clinical research units at the trial sites.

11.4.1 ^{18}F-FDG PET/CT Scan 1 (**Visit 2**) Day -6 to 0

The PET/CT scan (S1) must be performed after all other screening tests have been undertaken and after eligibility has been confirmed and signed by the PI or delegate. The scan must occur before the first dosing. Dosing can occur before the PET/CT is reported. Any significant findings at this visit necessitating withdrawal from the trial will be termed an AE unrelated to drug as this scan precedes drug administration. ^{18}F-FDG PET/CT imaging is expected to last for approximately 2 hours with a 90-minute break after radioligand injection.

A finger prick blood sugar test will be performed prior to the PET/CT scan and a cannula inserted. At the discretion of the PI further tests that need repeating from V1 can be performed.

A review of eligibility, withdrawal criteria, adverse events and concomitant medications will also be done at this visit.

Incidental findings on PET/CT scans

PET/CT scans are commonly used in metabolic and oncological scenarios for the diagnosis and follow-up of known conditions. In this clinical trial, the PET/CT scan is not being performed for diagnostic reasons in ACS patients and therefore clinical review of scan results is not usually clinically required prior to initiation of any treatment in this patient population. Thus, clinical governance reporting of scan results will not be required before initiation of low-dose IL-2 or placebo in the present trial. The management of any incidental findings will not be affected by low-dose IL-2, which, in itself, is currently licensed at higher doses for the treatment of certain types of cancer. Following a clinical review of any incidental findings by relevant multidisciplinary teams, if the patient meets any of the exclusion or stopping criteria, or at the discretion of the PI, the patient may have their dosing stopped and therefore will be withdrawn from the trial for further investigation or management of an incidental finding should this be required. Often, incidental findings may just require routine imaging follow-up (e.g. lung nodules) therefore this will be conducted clinically but the patient will not be withdrawn from the trial or from dosing for this reason.

11.4.2 Induction phase – **(Visit 3-7)** Day 1-5

Visit 3 must occur within 8 days of the first day of the patient's admission to the hospital with an ACS. At Royal Papworth Hospital this is defined as admission to their primary hospital and not the date of transfer to Papworth. Subsequent visits should occur on the scheduled day. All trial visits will be held at suitably equipped wards or clinical research units at the trial site(s).

Each visit will last approximately 1 hour and the following procedures will occur:

- (V3 only) – rechecking of inclusion and exclusion
- Checking withdrawal criteria (all visits after V3)
- Adverse event and concomitant medications reporting daily
- Baseline vital observations
- Physical examination
- Baseline 12-lead ECG with QTcB measurement will be performed pre-dosing
- V3 only – Review of safety bloods prior to dosing by the PI or delegate. Dosing decisions may be made on point of care bloods (if available) ahead of review of formal laboratory safety bloods assuming there is no major clinical deviation of parameters from recent blood tests. If POC is not available, formal lab bloods will be reviewed prior to dosing.
- Review of safety bloods from the previous visit (V4 – V7)
- Pre-dose bloods for V3 and V7 (approximately 55mls):
    - Clinical safety bloods
    - Treg and Lymphocyte subset analysis
    - Peripheral Blood Mononuclear Cell (PBMC) assays
- Cardiac biomarkers (hsCRP, IL-6, N-terminal pro-brain natriuretic peptide (BNP), and Troponin I)

- Pre-dose bloods for V4-6 (approximately 15mls):
    - Clinical safety bloods

- Trial patients will have an injection of IMP administered subcutaneously on the abdominal area. The site of injection on the abdominal area should be varied daily to minimise discomfort.
- Further 12-lead ECG with QTcB measurement will be performed approximately 30 minutes post-dosing (+/-5 minutes) (2 further 12 lead ECGs will be performed if the first QTcB meets stopping criteria and an average will be taken)
- Repeat vital observations will be performed approximately 30 minutes (+/- 5 minutes) post-dosing
- After approximately 30 minutes post-dosing, if there are no safety concerns, patients can be discharged back to usual clinical care or discharged home (where appropriate)
- The patients' stay may be extended and further investigations performed at the discretion of the investigator if the clinical scenario dictates this, for example the development of an adverse event (for example: hypotension, chest pain or arrhythmias).

11.4.3 Maintenance Phase – (**Visits 8 -14)** Day 12-54

Visit 8 will occur 7 days after V7. Visits 9-14 should each occur 7 days (+/- 3 days) after the intended previous visit. At the Principal investigator's discretion and with justification, a visit can be +/- 1 day from scheduled date. Scheduling of further visits is based on the intended visit date rather than actual visit date. All trial visits will be held at suitably equipped wards or clinical research units at the trial site. Review of safety bloods will be done prior to dosing by the PI or delegate. Dosing decisions may be made on point of care bloods (if available) ahead of review of formal laboratory safety bloods assuming there is no major clinical deviation of parameters from recent blood tests. If POC is not available, formal lab bloods will be reviewed prior to dosing.

Each visit will last approximately 1 hour and the following procedures will occur:

- Adverse event and concomitant medications reporting
- Baseline vital observations
- Brief physical examination
- Baseline 12-lead ECG with QTcB measurement will be performed pre-dosing
- Pre-dose blood tests for all visits (approximately 20mls in total):
    - Clinical safety bloods
- Trial patients will have an injection of IMP administered subcutaneously on the abdominal area. The site of injection on the abdominal area should be varied at each visit.
- Single 12-lead ECG with QTcB measurement will be performed approximately 30 minutes post-dosing (+/-5 minutes) (2 further 12 lead ECGs will be performed if the first QTcB meets stopping criteria and an average will be taken)
- Repeat vital observation will be performed approximately 30 minutes (+/- 5 minutes) post-dosing
- After approximately 30 minutes post-dosing, if there are no safety concerns, patients can be discharged back to usual clinical care or discharged home (where appropriate)
- The patient's stay may be extended and further investigations performed at the discretion of the investigator if the clinical scenario dictates this, for example the development of an adverse event (for example: hypotension, chest pain or arrhythmias)

For **Visits 8, 10, 12 and 14** additional pre-dose bloods (in addition to those listed above) will be taken (total volume 55mls):

- PBMC assays
- Treg and Lymphocyte subset analysis
- Cardiac biomarkers (hsCRP, IL-6, N-terminal pro-brain natriuretic peptide (BNP), and Troponin I)
- Serum pregnancy test where applicable (V10 and V14 only)

Post-dose transthoracic echocardiogram (TTE) may be undertaken at V14 (or anytime up to V16 including, if necessary, at an *ad hoc* visit.

11.4.4 Post-dose PET/CT – (**Visit 15**) Day 61

Visit 15 will occur 7 (+/-3) days after the intended final dosing visit V14.

A followup post-dose FDG-PET/CT will be performed during this visit. A finger prick blood sugar test will be performed prior to the PET/CT scan.
A cannula will be inserted and the bloods for the following will be taken:

- PBMC assays
- Treg and Lymphocyte subset analysis
- Cardiac biomarkers (hsCRP, IL-6, N-terminal pro-brain natriuretic peptide (BNP), and Troponin I)
- Full lipid profile

The scan is expected to last for approximately 2 hours which includes a 90-minute break after radioligand injection.
Patients may also undergo a follow-up post-dose transthoracic echocardiogram (TTE) if not performed at V14 (or ad hoc post final dose).

The following will also occur at this visit:

- Adverse events reporting
- Concomitant medications reporting

11.4.5 Follow-up – (**Visit 16**) Day 82

This visit will be held at suitably equipped wards or clinical research units at the trial site. It will take place 28 days (+/- 3 days) after the last intended dosing visit.

The visit will last approximately 1 hour and the following will occur:

- Adverse eventsreporting
- Concomitant medications reporting
- Vital observations
- Physical examination
- 12-lead ECG with QTcB measurement will be performed
- Post-dose Transthoracic echocardiogram (TTE) if not already done before
- Follow-up blood tests (55mls in total):
  - Clinical safety bloods
  - Tregs and Lymphocyte subset analysis
  - PBMC assays
  - Cardiac biomarkers (hsCRP, IL-6, N-terminal pro-brain natriuretic peptide (BNP), and Troponin I)
- Thyroid function tests
-
- Serum pregnancy test where applicable

The patient's stay can be extended and further investigations performed at the discretion of the investigator if clinical scenario dictates for examples: hypotension, chest pain or arrhythmias.

The trial will routinely end for individual patients at V16. At the discretion of the PI, further visits or telephone contacts can be arranged to follow up unresolved issues/adverse events until the point of resolution.

11.5 Schedule of Assessments

**Visit no ->**	**1**	**2**	**3**	**4**	**5**	**6**	**7**	**8**	**9**	**10**	**11**	**12**	**13**	**14**	**15**	**16**
**Treatment Day**	**-7 to 0**	**-6 to 0**	**1**	**2**	**3**	**4**	**5**	**12[a]**	**19[a]**	**26[a]**	**33[a]**	**40[a]**	**47[a]**	**54[a]**	**61[a]**	**82[a]**
Informed consent	x															
18F-FDG PET/CT Scan[b]		x													x	
Demography[c]	x															
Height/weight	x															
Medical history	x															
Medication history	x															
Inclusion/exclusion	x	x	x													
Adverse events	x	x	x	x	x	x	x	x	x	x	x	x	x	x	x	x
Concomitant medications	x	x	x	x	x	x	x	x	x	x	x	x	x	x	x	x
Serum pregnancy test	x									x				x		x
Physical examination[d]	x		x	x	x	x	x	x	x	x	x	x	x	x		x
Vital observations[e]	x		x	x	x	x	x	x	x	x	x	x	x	x		x
ECG	X[f]		x	x	x	x	x	x	x	x	x	x	x	x		x
ECHO	X[g]														x[h]	
IMP administration			x	x	x	x	x	x	x	x	x	x	x	x		
Blood Tests																
Safety bloods[i]	x		x	x	x	x	x	x	x	x	x	x	x	x		x
OPTIONAL Point of care bloods			x					x	x	x	x	x	x	x		
TSH	x															x
Tregs and Lymphocytes			x				x	x		x		x		x	x	x
PBMC assay			x				x	x		x		x		x	x	x
Cardiac biomarkers[j]			x				x	x		x		x		x	x	x
hsCRP	x															
Full lipid profile	x														x	

[a] These visits can be scheduled +/- 3 days.
[b] PET/CT assessment to be preceded by a fingerprick blood glucose test.
[c] Including DOB, age, gender, race.
[d] Including cardiovascular, respiratory, gastrointestinal, neurological and skin examinations.
[e] Temperature, blood pressure, heart rate, respiratory rate and oxygen saturations.
[f] 12-lead ECG in triplicate with average QTcB.
[g] May be done anytime after screening at an ad hoc visit and prior to PET/CT, but before eligibility sign-off.
[h] May be done anytime between V14 and V16.
[i] Safety bloods (including but not limited to): haematology (FBC and differentials), clinical biochemistry (electrolytes, urea, creatinine), liver function (ALT, ALP, albumin, bilirubin).
[j] hsCRP, IL-6, BNP, Troponin I.

11.6 Trial specific procedures

Specific details of all procedures will be provided in the trial procedures manual (TPM).

Blood Pressure

- Blood pressure will be measured pre- and post-dosing using a validated automated device.

Brief physical examination

- Brief physical examination includes physical examination of the cardiovascular, gastrointestinal and neurological systems as well as of the lungs and skin.

ECG with QTcB measurement

- 12-lead ECGs will be obtained at each time point during the trial using an ECG machine that automatically calculates the heart rate and measures PR, QRS, QT, and QTcB intervals. All ECGs are manually over read to check QTcB parameters by the investigator or suitably qualified and delegated member of the trial team to ensure the correct calculation of QTcB. All ECGs will be checked and signed and in the circumstance of an over read, this will be clearly documented on the subject's affected ECG.
- ECGs will be obtained in the semi-supine position after the subject has been resting for at least 5 minutes.
- For time points where ECGs are collected in triplicate these will be carried out over a brief period of approximately 5 minutes.

Imaging

- A General Electric Discovery 690 combined positron emission tomography (PET)/computed tomography (CT) scanner (Milwaukee, Wisconsin), or equivalent scanner, will be used for PET/CT imaging. Vascular imaging will be performed using reproducible, validated methods for image acquisition, reconstruction and interpretation as recommended by the European Association of Nuclear Medicine for the conduct of clinical trials using ^{18}F-fluorodeoxyglucose (FDG) PET[46].

- Patients should be fasted for approximately 6 hours prior to ^{18}F-FDG PET imaging and capillary blood glucose concentration should be confirmed as <7.0 mmol/l prior to scanning. If the blood glucose concentration is >11.0 mmol/l, this level should first be repeated, and if confirmed the scan should be re-scheduled as per departmental protocol at the discretion of the supervising trial doctor. For blood glucose values between 7-11 mmol/l, the scan may proceed, be delayed or re-scheduled at the discretion of the Chief Investigator. This may occur outside the trial window as defined in 11.4.1, and is at the discretion of the Chief Investigator.

- A dose of approximately 240Mbq ^{18}F-FDG will be injected through a peripheral venous cannula, followed by 10 mL flush of normal saline, with the patient seated in an armchair, where they will rest in a quiet environment for a period of time (up to 90 minutes). Patients will be requested to avoid excessive speaking or swallowing/chewing to minimise physiological ^{18}F-FDG tracer uptake in the head and neck muscles.

  The ascending aorta will be imaged 90 minutes after the ^{18}F-FDG injection. Attenuation correction and non-contrast CT scans of the ascending aorta will initially be performed. This will be followed by a single bed PET scan acquired in

3D list mode for 10 minutes, with the superior portion of the aortic arch as the upper anatomical landmark of the scan.

- Carotid artery imaging will be undertaken immediately after the PET/CT scans of the ascending aorta. With the patients head and neck placed comfortably in a head holder and arms secured at their sides, attenuation correction and non-contrast CT scans of the neck will be performed. This will be followed by a single bed PET scan acquired in 3D list mode for 15 minutes, with the external auditory meatus as the upper anatomical landmark of the scan.

- PET data for the ascending aorta and carotids will be reconstructed using iterative 3D time of flight ordered-subset expectation maximization with standard corrections applied ± point-spread function modelling to reduce partial volume error.

- Arterial radioactivity concentration (standardized uptake value (SUV)) will be quantified from maximum voxel value within regions of interest drawn on PET-CT images, normalised by blood pool activity and averaged with neighbouring slices to minimise the impact of noise, to derive tissue-to-blood ratios (TBR).

Blood tests

The following routine measurements will be performed in blood samples:

- Safety clinical blood tests defined as (but not limited to): haematology (full blood count and differential), clinical biochemistry (electrolytes, urea, creatinine, liver function (ALT, ALP, albumin, bilirubin)
- Thyroid function blood test (TSH - V1 and V16 only [fT4 will only be performed if TSH is abnormal])
- Cardiac biomarkers (hsCRP, IL-6, N-terminal pro-brain natriuretic peptide (BNP), Troponin I)
- Full lipid profile (at screening and V15 only)
- Serum pregnancy test where applicable
- Treg and Lymphocyte subset analysis
- PBMC assays

11.7 End of Trial Participation

Patients will finish their trial participation at the end of V16. At the discretion of the PI, further visits or telephone contacts can be arranged to follow up unresolved issues or AEs until the point of resolution.

At the end of the trial patients will return to normal standard of care.

11.8 Trial restrictions

Both (sexually active) men and women should use at least two effective methods of contraception during the trial.

Women of childbearing potential are required to use two of the following, reliable forms of contraception for the entire duration of the trial and for 3 months after the completion of the last treatment visit. This includes:

- Intrauterine Device (IUD, coil or intrauterine system)
- Oral contraception (either combined or progestogen alone)
- Contraceptive implant, injections or patches

- Condom **and** cap or diaphragm **plus** spermicide (chemical that kills sperm)
- Male sterilisation

Men are required to use two of the following, reliable forms of contraception for the entire duration of the trial and for 3 months after the completion of the last treatment visit. This includes:

- a condom and spermicide (chemical that kills sperm), with female partner(s)using another method of contraception
- Men should also use a condom to protect male partners, or female partners who are pregnant or breastfeeding, from exposure to the trial medicine in semen.

Trial patients do not need to use contraception if:

- They are a woman who cannot become pregnant (e.g. have had a hysterectomy, removal of ovaries and/or sterilisation; or are post-menopausal for longer than two years)
- If they practice true abstinence (where this is in accordance with the patients preferred and usual lifestyle). If the patient becomes sexually active, they must use two of the methods listed above.

Patients should refrain from donating sperm and blood for the duration of the trial and for 3 months after completion of the last treatment visit.

12 Assessment of Safety

12.1 Definitions

12.1.1 Adverse event (AE)

Any untoward medical occurrence in a patient or clinical trial patient administered a medicinal product and which does not necessarily have a causal relationship with this treatment.

An adverse event can therefore be any unfavourable and unintended sign (including an abnormal laboratory finding), symptom, or disease temporally associated with the use of an investigational medicinal product, whether or not considered related to the investigational medicinal product.

Please note: Recording of all adverse events must start from the point of Informed Consent regardless of whether a patient has yet received a medicinal product.

12.1.2 Adverse reaction to an investigational medicinal product (AR)

All untoward and unintended responses to an investigational medicinal product related to any dose administered. All adverse events judged by either the reporting investigator or the sponsor as having a reasonable causal relationship to a medicinal product qualify as adverse reactions. The expression reasonable causal relationship means to convey in general that there is evidence or argument to suggest a causal relationship.

12.1.3 Unexpected adverse reaction

An adverse reaction, the nature, or severity of which is not consistent with the applicable reference safety information (RSI) (e.g. investigator's brochure for an unapproved investigational product or summary of product characteristics (SmPC)

for an authorised product).

When the adverse reaction is not consistent with the applicable RSI this adverse reaction should be considered as unexpected.

12.1.4 Serious adverse event or serious adverse reaction (SAE / SAR)

Any untoward medical occurrence that at any dose:

- results in death
- is life-threatening
- requires hospitalisation or prolongation of existing inpatients´ hospitalisation
- results in persistent or significant disability or incapacity
- is a congenital anomaly or birth defect.
- is an important medical event - Some medical events may jeopardise the patient or may require an intervention to prevent one of the above characteristics/ consequences. Such events (hereinafter referred to as 'important medical events') should also be considered as 'serious'

Life-threatening in the definition of a serious adverse event or serious adverse reaction refers to an event in which the patient was at risk of death at the time of event; it does not refer to an event which hypothetically might have caused death if it were more severe.

12.1.5 Suspected Unexpected Serious Adverse Reaction (SUSAR)

A serious adverse reaction, the nature and severity of which is not consistent with the information set out in the Reference Safety Information

12.1.6 Reference Safety Information (RSI)

A list of medical events that defines which reactions are expected for the IMP within a given trial and thus determining which Serious Adverse Reactions (SARs) require expedited reporting.

The RSI is contained in a clearly identified section of the Summary of Product Characteristics (SmPC) or the Investigator's Brochure (IB)

For this trial the Reference Safety Information is: Section 4.8 – Undesirable effects, of the SmPC for Proleukin (IL-2) powder for solution for injection or infusion (Clinigen Healthcare Ltd), dated 31 May 2019.

However, occurrence of the following events **if assessed as related to the IMP** and meeting the **Serious** criteria must be reported as Suspected Unexpected Serious Adverse Reactions (SUSARs):

- Tachycardia
- Arrhythmia
- Chest pain
- Cyanosis
- Transient ECG changes
- Myocardial ischaemia
- Palpitations
- Cardiac failure
- Myocarditis
- Cardiomyopathy
- Cardiac arrest
- Pericardial effusion

- Ventricular hypokinesia
- Cardiac tamponade
- Thrombosis
- Hypertension
- Hypotension
- Embolism.

12.2 Expected Adverse Reactions/Serious Adverse Reactions (AR /SARs)

All expected Adverse Reactions are listed in the latest MHRA approved version of the RSI as specified in section 12.1.6. This must be used when making a determination as to the expectedness of the adverse reaction.

The adverse reactions related to low dose IL-2 are generally not serious in nature and include:

- Systemic symptoms

The most frequently observed side effect is a flu-like syndrome characterised by fever, shivering, asthenia, rhinitis, arthralgia, myalgia and headaches. These symptoms are expected to occur in less than 20% of patients at the doses used in the present trial.

- Localised reaction at site of injection

Transient local reaction with symptoms of pain and signs of erythema or nodule: These are the most common non-serious adverse reactions and occur in approximately 50% of patients.

- Generalized gastrointestinal symptoms

Anorexia, nausea, vomiting, abdominal pain and diarrhoea: These symptoms are expected to occur in less than 20% of patients at the doses used in the present trial.

- Haematopoiesis

An initial decrease in absolute lymphocyte count that recovers in most patients after one week post treatment with IL-2: An initial increase in eosinophil count followed by a gradual decline after treatment. A decrease in platelet count of greater than 20% but less than 40% in some patients treated with repeated doses of subcutaneous low dose IL-2.

- Thyroid dysfunction

Thyroid function test (TFT) abnormalities have been noted in some patients treated with IL-2. After cessation of IL-2, TFTs returned to normal. A thyroid function test (TSH) will be checked at entry into the trial and at follow-up.

12.2.1 Injection site reaction

Injection site reactions (ISR) are a common occurrence and, based on data from previous studies, we would expect an incidence of at least 40-50% of all injection sites.

Injection site reaction is defined as: a localised reaction on the skin around the site of subcutaneous injection of the IMP. The reaction is comprised of a red or pink

erythematous rash and localised pruritus can occur. Injection site reactions can sometimes also be associated with temporary bruising or nodule formation.

If any adverse reaction meets the criteria for seriousness, this must be reported as per section 12.5 of the protocol.

12.3 Expected Adverse Events/Serious Adverse Events (AE/SAE)

12.3.1 Hospital admissions

Current hospital admission with ACS is part of the inclusion criteria for this trial; therefore, the hospitalisation in this instance will not be recorded and reported as an SAE unless the patients' hospitalisation is prolonged due to reasons beyond routine clinical care for their condition. However, prolonged admission for social (i.e. non-clinical) reasons will not be reported as SAEs.

Planned/elective hospitalisation for further investigation and management of their disease will not be reported as SAEs. These events will be recorded in the trial CRF but simply not recorded as AEs or SAEs unless the event is deemed by the Principal Investigator to be an Adverse or Serious Adverse Reaction in which case it will be recorded as such. These may include but are not limited to in-patient tests/clinical management such as

1. Diagnostic angiograms +/- percutaneous coronary intervention (PCI)
2. Coronary artery bypass grafting
3. Vascular surgery due to complications of cardiac catheterisation/PCI
4. Other imaging/procedures due to complications of cardiac catheterisation/PCI

These events will be recorded in the trial CRF but not recorded as AEs or SAEs unless the event is deemed by the Principal Investigator to be an AR or SAR in which case it will be recorded/reported as such.

Due to the nature of the local hospital set up, ACS patients at Addenbrooke's routinely are transferred to Papworth for further investigation and treatment. This will not be an SAE.

12.3.2 Events commonly occurring in patients with ACS

The trial patients are acutely unwell and hospitalised therefore there are commonly occurring events which can be defined as:

a. Chest pain which is managed conservatively, by medication or by emergency transfer to another hospital.
b. Common procedure related complications and their treatment e.g. haematoma, pain (e.g analgesia), infection (e.g antibiotics), bleeding at vascular site (e.g blood transfusion), pseudo-aneurysms etc.
c. Post-procedural (post-angiography) troponin and CRP rises
d. Arrhythmias such as non-sustained ventricular tachycardia (NSVT) or temporary heart block
e. Reduction in myocardial systolic function leading to symptoms of heart failure

These events will be reported and causality determined by the CI on an individual basis.

12.4 Evaluation of adverse events

The Sponsor expects that adverse events are recorded from the point of Informed Consent regardless of whether a patient has yet received a medicinal product. Individual adverse events should be evaluated by the investigator. This includes the evaluation of its seriousness, and any relationship between the investigational medicinal product(s) and/or concomitant therapy and the adverse event (causality).

12.4.1 Assessment of seriousness

Seriousness is assessed against the criteria in section 12.1.4. This defines whether the event is an adverse event, serious adverse event or a serious adverse reaction

12.4.2 Assessment of causality

Definitely: A causal relationship is clinically/biologically certain. **This is therefore an Adverse Reaction**

Probable: A causal relationship is clinically / biologically highly plausible and there is a plausible time sequence between onset of the AE and administration of the investigational medicinal product and there is a reasonable response on withdrawal. **This is therefore an Adverse Reaction.**

Possible: A causal relationship is clinically / biologically plausible and there is a plausible time sequence between onset of the AE and administration of the investigational medicinal product. **This is therefore an Adverse Reaction.**

Unlikely: A causal relation is improbable and another documented cause of the AE is most plausible. **This is therefore an Adverse Event.**

Unrelated: A causal relationship can be definitely excluded and another documented cause of the AE is most plausible. **This is therefore an Adverse Event.**

Unlikely and Unrelated causalities are considered NOT to be trial drug related. Definitely, Probable and Possible causalities are considered to be trial drug related.

A pre-existing condition must not be recorded as an AE or reported as an SAE unless the condition worsens during the trial and meets the criteria for reporting or recording in the appropriate section of the CRF.

12.4.3 Clinical assessment of severity

Mild: The patient is aware of the event or symptom, but the event or symptom is easily tolerated

Moderate: The patient experiences sufficient discomfort to interfere with or reduce his or her usual level of activity

Severe: Significant impairment of functioning; the subject is unable to carry out usual activities and / or the patient's life is at risk from the event.

12.4.4 Recording of adverse events

Adverse events and adverse reactions should be recorded in the medical notes and the appropriate section of the CRF and/or AE/AR log. Adverse events will be MedDRA coded and entered into the trial database. Serious Adverse Events and Serious Adverse Reactions should be reported to the sponsor as detailed in section 12.5.

12.5 Reporting serious adverse events

The Principal Investigator needs to record all adverse events on the CRFs (except where **the protocol has stated otherwise - see section 12.3) and report all SAEs to CCTU within 24 hours by email** cambs.cardiovascular@nhs.net

Each Principal Investigator needs to record all adverse events and report serious adverse events to the Chief Investigator using the trial specific SAE form within 24 hours of their awareness of the event.

The Chief Investigator is responsible for ensuring the assessment of all SAEs for expectedness and relatedness is completed and the onward notification of all SAEs to the Sponsor immediately but not more than 24 hours of first notification. The sponsor has to keep detailed records of all SAEs reported to them by the trial team.

The Chief Investigator is also responsible for prompt reporting of all serious adverse event findings to the competent authority (e.g. MHRA) of each concerned Member State if they could:

- adversely affect the health of patients
- impact on the conduct of the trial
- alter the risk to benefit ratio of the trial
- alter the competent authority's authorisation to continue the trial in accordance with Directive 2001/20/EC

12.6 Reporting of Suspected Unexpected Serious Adverse Reactions (SUSARs)

All suspected adverse reactions related to an investigational medicinal product (the tested IMP and comparators) which occur in the concerned trial, and that are both unexpected and serious (SUSARs) are subject to expedited reporting. Please see section 12.1.6 for the Reference Safety Information to be used in this trial.

12.6.1. Who should report and whom to report to?

The Sponsor delegates the responsibility of notification of SUSARs to the Chief Investigator. The Chief Investigator must report all the relevant safety information previously described, to the:

- Sponsor
- competent authorities in the concerned member states (eg MHRA)
- Ethics Committee in the concerned member states

The Chief Investigator shall inform all investigators concerned of relevant information about SUSARs that could adversely affect the safety of patients.

12.6.2. When to report?

12.6.2.1. Fatal or life-threatening SUSARs

All parties listed in 12.6.1 must be notified as soon as possible but no later than **7 calendar days** after the trial team and Sponsor has first knowledge of the minimum criteria for expedited reporting.
In each case relevant follow-up information should be sought and a report completed as soon as possible. It should be communicated to all parties within an additional **8 calendar days**.

12.6.2.2. Non-fatal and non-life-threatening SUSARs

All other SUSARs and safety issues must be reported to all parties listed in 12.6.1 as soon as possible but no later than **15 calendar days** after first knowledge of the minimum criteria for expedited reporting. Further relevant follow-up information should be given as soon as possible.

12.6.3. How to report?

12.6.3.1. Minimum criteria for initial expedited reporting of SUSARs

Information on the final description and evaluation of an adverse reaction report may not be available within the required time frames for reporting. For regulatory purposes, initial expedited reports should be submitted within the time limits as soon as the minimum following criteria are met:

a) a suspected investigational medicinal product
b) an identifiable patient (e.g. trial patient code number)
c) an adverse event assessed as serious and unexpected, and for which there is a reasonable suspected causal relationship
d) an identifiable reporting source

and, when available and applicable:

- an unique clinical trial identification (EudraCT number or in case of non- European Community trials the sponsor's trial protocol code number)
- an unique case identification (i.e. sponsor's case identification number)

12.6.3.2. Follow-up reports of SUSARs

In case of incomplete information at the time of initial reporting, all the appropriate information for an adequate analysis of causality should be actively sought from the reporter or other available sources. Further available relevant information should be reported as follow-up reports.
In certain cases, it may be appropriate to conduct follow-up of the long-term outcome of a particular reaction.

12.6.3.3. Format of the SUSARs reports

Electronic reporting is the expected method for expedited reporting of SUSARs to the competent authority. The format and content as defined by the competent authority should be adhered to.

12.7 Pregnancy Reporting

All patient pregnancies within the trial must be reported to the Chief Investigator and the Sponsor using the relevant Pregnancy Reporting Form within 24 hours of notification. Pregnancies must be reported for all patients for the duration of their trial participation and for 3 months following the last dose of IMP.

Pregnancy is not considered an AE unless a negative or consequential outcome is recorded for the mother or child/foetus. If the outcome meets the serious criteria, this would be considered an SAE.

13 Toxicity – Emergency Procedures

In the event of an acute hypersensitivity reaction to IL-2 administration supportive care will be given to the patient according to local clinical procedures.

14 Evaluation of results

Please refer to section 8.6 Trial endpoints.

14.1 Response criteria

Please refer to section 8.6 Trial endpoints.

15 Storage and Analysis of Samples

Named samples may be directly sent to Cambridge University Hospitals NHS Foundation Trust pathology department or Royal Papworth Hospital pathology department for analysis and then destroyed shortly after. Some samples will be securely stored in freezers located on the Cambridge Biomedical Campus for analysis later. Patients on this trial will be assigned a unique trial number and this number will be used as an identifier for all stored samples collected from them. Authorised staff only will have access to the code and personal information about the donor. Only personnel authorised by the Chief Investigator and/or Principal Investigator(s) will be responsible for the storage, access and release of these samples for analysis. Stored samples may be analysed to fulfil endpoints as part of this trial or may be used in other ethically approved research with the patient's consent. Full details of how samples are processed, stored and analysed and if necessary destroyed for this trial can be found in the IVORY Trial Procedures Manual.

16 Statistics

16.1 Statistical methods

Three approaches will be used to quantify vascular inflammation: 1) The pre-specified whole vessel primary endpoint of change in the average maximum TBR (TBR_{max}) for all segments within the index vessel[47], with a complementary analysis displaying the group distribution of average TBR_{max} using a frequency histogram; 2) An analysis of change from baseline in average TBR_{max} for active segments within the index vessel; and 3) an analysis of the probability of a segment being active within the index vessel.

The vessel with the highest number of slices with max-TBR>1.6 will be identified within each participant at the initial imaging visit (V2) and subsequently followed (V15). This will be termed the "index vessel". An active segment is defined as a segment of a vessel with a TBR of ≥1.6

Change from baseline in average TBR_{max} will be analysed using analysis of covariance, fitting treatment as fixed effect, and including baseline value as a covariate and ST-elevation status as covariate. Point estimates and corresponding 95% confidence intervals (CI) will be constructed for the relevant comparisons of interest.

Average TBR_{max} data from the final visit will be plotted to show the distribution from all segments from all index vessels within each treatment group. The difference between low-dose IL-2 and placebo will be calculated and tested using a nonparametric permutation test at the patient level.

The number of active segments and the total number of segments will be included in logistic regression analyses to model the probability of a segment being active. For baseline correction within each group, a model will be fitted with terms for treatment and day. For placebo and baseline correction, a model will be fitted with treatment term and including the baseline proportion of active segments as covariate. Point estimates and corresponding 95% CI are constructed to establish the odds ratio for the relevant comparisons of interest.

Biomarker data are analysed by analysis of covariance fitting terms for regimen, day, and interaction of day and regimen as fixed effects; patient as a random effect; and baseline biomarker at day 1 as a covariate.

Summary statistics of endpoints broken down by treatment arm and visit where appropriate will be provided. Categorical variables will report percentages and counts (p% (x/n)); continuous variables will report, mean, median, SD, min and max. Exploratory figures in the form of stacked bar-charts and box-and-whisker plots for categorical and continuous variables respectively will be provided.

A detailed statistical analysis plan will be produced before the final database lock.

16.2 Interim analyses

There are no planned interim analyses for this trial.

16.3 Number of Patients to be enrolled

Sample size is based on an absolute difference of 0.2 in the primary endpoint (TBR_{max} in the index vessel) between placebo and active treatment at the end of the treatment period. This is equivalent to a 10% difference from a reference value of 2.02 and equivalent to the size effect observed after atheroprotective therapy[39,42,43]. Interventions that have reported less than 10% difference in TBR_{max} in the index vessel between active treatment and placebo at the end of the treatment period [40,48] failed to reduce CV outcomes[49-51].

Assuming a SD of 0.24[39], 24 patients per arm, testing at 2-sided 5% significance level, will provide 80% power. Therefore, a sample size of 30 completed patients per arm was selected to account for scans which may not be analysable, or poor scans due to patient habitus, movement and so on.

This sample size of n=30 per group also allows the detection of a baseline-corrected 6% difference in TBR_{max} in the index vessel (mean 0.125, SD 0.166)[39] between placebo and active treatment at the end of the treatment period, at 2-sided 5% significance level and 80% power.

16.3.1 Safety Population

Safety reporting will be based upon the safety population defined as any patients who receive any trial drug or placebo dose.

16.3.2 Analysis Population

The reporting of biomarker endpoints, including Tregs, will be based upon the Analysis Population defined as patients who receive trial drug or placebo and complete the treatment course after randomisation.

16.4 Procedure to account for missing or spurious data

We do not anticipate a high level of drop out. Hence we will report the number of non-missing values for comparison to the relevant population size. Statistics will be calculated based on complete cases, which assumes there is no association between a value being unobserved, and the underlying value.

16.5 Definition of the end of the trial

The end of trial will be the date 18 months after the last patient's last visit to allow sufficient time to complete all primary, secondary, and exploratory endpoints and their corresponding analyses, and if applicable, all re-analyses of samples.

17 Data handling and record keeping

17.1 CRF

All data will be transferred into an Case Report Form (CRF) which will be anonymised. All trial data in the CRF must be extracted from and be consistent with the relevant source documents. The CRFs must be completed, dated and signed by the investigator or designee in a timely manner. It remains the responsibility of the investigator for the timing, completeness, legibility and accuracy of the CRF. The CRF will be accessible to trial coordinators, data managers, the investigators, Clinical Trial Monitors, Auditors and Inspectors as required.Completed originals of the CRFs should be sent to the trial coordination centre within timeframes outlined in the Data Management Plan.

The investigator will retain a copy of each completed CRF page at site. The investigator will supply the trial coordination centre with any required, anonymised background information from the medical records as required.

Any trial related documentation that is sent to the trial coordination centre must not contain patient identifiable data.

All CRF pages must be clear, legible and completed in black ink. Any errors should be crossed with a single stroke so that the original entry can still be seen. Corrections should be inserted and the change dated and initialled by the investigator or designee. If it is not clear why the change has been made, an explanation should be written next to the change. Typing correction fluid must not be used.

17.2 Source Data

To enable peer review, monitoring, audit and/or inspection the investigator must agree to keep records of all participating patients (sufficient information to link records e.g. hospital records and samples) and all original signed informed consent forms.

Source data may include but is not limited to:

- Signed informed consent forms
- Patient medical records (electronic or paper)
- Pre-defined sections of the Case Report Forms (CRFs) detailed in the Data Management Plan
- Blood results (electronic or paper)
- Sample logs
- PrescriptionsECHO resultsECG print outs
- ^{18}F-FDG PET/CT results

17.3 Data Protection & Patient Confidentiality

All investigators and trial site staff involved in this trial must comply with the requirements of the Data Protection Act 2018 and Trust Policy with regards to the collection, storage, processing, transfer and disclosure of personal information and will uphold the Act's core principles.

18 Trial Management Group

The Trial Management Group (TMG) will be comprised of blinded individuals who are responsible for the day to day running of the trial.

The TMG will meet on a regular basis and will be responsible for the overall supervision of trial progress. The TMG will make major decisions about the trial including whether protocol amendments are required, and will discuss recommendations from the Data Monitoring Committee (DMC). The TMG will also be notified about all patient withdrawals including those arising from discontinuation of dosing.

The TMG will be comprised of:

- The Chief Investigator and collaborators
- A research nurse
- The trial coordinator

The trial statistician will be unblinded.

19 Independent Data Monitoring Committee

The DMC will be comprised of an unblinded independent group, as defined in a separate charter document which will define the role of the DMC. The charter document will be generated prior to opening the trial.

The DMC will be responsible for the review of all safety (but not exploratory) data and will meet quarterly for the first year of the trial opening to recruitment and every six months until the follow-up visit of the last patient.

Additionally, ad hoc IDMC meetings will be triggered if conditions are met as specified in section 9.6 Trial Stopping Criteria.

20 Ethical & Regulatory considerations

20.1 Ethical committee review

Before the start of the trial or implementation of any amendment we will obtain approval of the trial protocol, protocol amendments, informed consent forms and other relevant documents e.g., advertisements and GP information letters if applicable from the REC. All correspondence with the REC will be retained in the Trial Master File/Investigator Site File.

Annual reports will be submitted to the REC in accordance with national requirements. It is the Chief Investigator's responsibility to produce the annual reports as required.

20.2 Regulatory Compliance

The trial will not commence until a Clinical Trial Authorisation (CTA) is obtained from the MHRA. The protocol and trial conduct will comply with the Medicines for Human Use (Clinical Studies) Regulations 2004 and any relevant amendments.

Development Safety Update Reports (DSURs) will be submitted to the MHRA in accordance with national requirements. It is the Chief Investigator's responsibility to produce the annual reports as required.

20.3 Protocol Amendments

Protocol amendments must be reviewed and agreement received from the Sponsor for all proposed amendments prior to submission.

The only circumstance in which an amendment may be initiated prior to MHRA, HRA and REC approval is where the change is necessary to eliminate apparent, immediate risks to the patients (Urgent Safety Measures). In this case, accrual of new patients will be halted until the MHRA, HRA and REC approval has been obtained.

20.4 Peer Review

This trial protocol has been reviewed by a specialist independent group within the Sponsor organisation (Cambridge University Hospitals NHS Foundation Trust Research Advisory Committee).

20.5 Declaration of Helsinki and Good Clinical Practice

The trial will be performed in accordance with the spirit and the letter of the declaration of Helsinki, the conditions and principles of Good Clinical Practice, the protocol and applicable local regulatory requirements and laws.

20.6 GCP Training

All trial staff must hold evidence of appropriate GCP training or undergo GCP training prior to undertaking any responsibilities on this trial. This training should be updated every 2 years or in accordance with your Trust's policy.

21 Sponsorship, Financial and Insurance

The trial will be sponsored by Cambridge University Hospitals NHS Foundation Trust and University of Cambridge. The trial is funded by the MRC.

Cambridge University Hospitals NHS Foundation Trust, as a member of the NHS Clinical Negligence Scheme for Trusts, will accept full financial liability for harm caused to patients in the clinical trial caused through the negligence of its employees and honorary contract holders. There are no specific arrangements for compensation should a patient be harmed through participation in the trial, but no-one has acted negligently.

22 Monitoring, Audit & Inspection

The investigator must make all trial documentation and related records available should an MHRA Inspection occur. Should a monitoring visit or audit be requested, the investigator must make the trial documentation and source data available to the Sponsor's representative. All patient data must be handled and treated confidentially.

The Sponsor's monitoring frequency will be determined by an initial risk assessment performed prior to the start of the trial. A detailed monitoring plan will be generated detailing the frequency and scope of the monitoring for the trial. Throughout the course of the trial, the risk assessment will be reviewed and the monitoring frequency adjusted as necessary.

23 Protocol Compliance and Breaches of GCP

Prospective, planned deviations or waivers to the protocol are not allowed under the UK regulations on Clinical Studies and must not be used.

Protocol deviations, non-compliances, or breaches are departures from the approved protocol. They can happen at any time, but are not planned. They must be adequately documented on the relevant forms and reported to the Chief Investigator and Sponsor immediately.

Deviations from the protocol which are found to occur constantly again and again will not be accepted and will require immediate action and could potentially be classified as a serious breach.

Any potential/suspected serious breaches of GCP must be reported immediately to the Sponsor without any delay.

24 Publications policy

Ownership of the data arising from this trial resides with the trial team. On completion of the trial the data will be analysed and tabulated and a Final Trial Report prepared.

25 References

1 Libby, P. Mechanisms of acute coronary syndromes and their implications for therapy. *The New England journal of medicine* **368**, 2004-2013, doi:10.1056/NEJMra1216063 (2013).

2 Arbab-Zadeh, A., Nakano, M., Virmani, R. & Fuster, V. Acute coronary events. *Circulation* **125**, 1147-1156, doi:10.1161/circulationaha.111.047431 (2012).

3 Mega, J. L. *et al.* Rivaroxaban in patients with a recent acute coronary syndrome. *The New England journal of medicine* **366**, 9-19, doi:10.1056/NEJMoa1112277 (2012).

4 Wiviott, S. D. *et al.* Prasugrel versus clopidogrel in patients with acute coronary syndromes. *The New England journal of medicine* **357**, 2001-2015, doi:10.1056/NEJMoa0706482 (2007).

5 Liuzzo, G. *et al.* The prognostic value of C-reactive protein and serum amyloid a protein in severe unstable angina. *The New England journal of medicine* **331**, 417-424, doi:10.1056/nejm199408183310701 (1994).

6 Ritchie, M. E. Nuclear factor-kappaB is selectively and markedly activated in humans with unstable angina pectoris. *Circulation* **98**, 1707-1713 (1998).

7 Buffon, A. *et al.* Widespread coronary inflammation in unstable angina. *The New England journal of medicine* **347**, 5-12, doi:10.1056/NEJMoa012295 (2002).

8 Falk, E., Nakano, M., Bentzon, J. F., Finn, A. V. & Virmani, R. Update on acute coronary syndromes: the pathologists' view. *European heart journal* **34**, 719-728, doi:10.1093/eurheartj/ehs411 (2013).

9 Dutta, P. *et al.* Myocardial infarction accelerates atherosclerosis. *Nature* **487**, 325-329, doi:10.1038/nature11260 (2012).

10 Hansson, G. K. Inflammation, atherosclerosis, and coronary artery disease. *The New England journal of medicine* **352**, 1685-1695, doi:10.1056/NEJMra043430 (2005).

11 McPherson, R. & Davies, R. W. Inflammation and coronary artery disease: insights from genetic studies. *The Canadian journal of cardiology* **28**, 662-666, doi:10.1016/j.cjca.2012.05.014 (2012).

12 Liuzzo, G. *et al.* Unusual CD4+CD28null T lymphocytes and recurrence of acute coronary events. *Journal of the American College of Cardiology* **50**, 1450-1458, doi:10.1016/j.jacc.2007.06.040 (2007).

13 Ammirati, E. *et al.* Expansion of T-cell receptor zeta dim effector T cells in acute coronary syndromes. *Arteriosclerosis, thrombosis, and vascular biology* **28**, 2305-2311, doi:10.1161/atvbaha.108.174144 (2008).

14 Caligiuri, G., Paulsson, G., Nicoletti, A., Maseri, A. & Hansson, G. K. Evidence for antigen-driven T-cell response in unstable angina. *Circulation* **102**, 1114-1119 (2000).

15 Ammirati, E. *et al.* Circulating CD4+CD25hiCD127lo regulatory T-Cell levels do not reflect the extent or severity of carotid and coronary atherosclerosis. *Arteriosclerosis, thrombosis, and vascular biology* **30**, 1832-1841, doi:10.1161/atvbaha.110.206813 (2010).

16 Cheng, X. *et al.* The Th17/Treg imbalance in patients with acute coronary syndrome. *Clinical immunology (Orlando, Fla.)* **127**, 89-97, doi:10.1016/j.clim.2008.01.009 (2008).

17 Han, S. F. *et al.* The opposite-direction modulation of CD4+CD25+ Tregs and T helper 1 cells in acute coronary syndromes. *Clinical immunology (Orlando, Fla.)* **124**, 90-97, doi:10.1016/j.clim.2007.03.546 (2007).

18 Mor, A., Luboshits, G., Planer, D., Keren, G. & George, J. Altered status of CD4(+)CD25(+) regulatory T cells in patients with acute coronary syndromes. *European heart journal* **27**, 2530-2537, doi:10.1093/eurheartj/ehl222 (2006).

19 Li, Q. *et al.* Distinct different sensitivity of Treg and Th17 cells to Fas-mediated apoptosis signaling in patients with acute coronary syndrome. *International journal of clinical and experimental pathology* **6**, 297-307 (2013).

20 Wigren, M. *et al.* Low levels of circulating CD4+FoxP3+ T cells are associated with an increased risk for development of myocardial infarction but not for stroke. *Arteriosclerosis, thrombosis, and vascular biology* **32**, 2000-2004, doi:10.1161/atvbaha.112.251579 (2012).

21 Hofmann, U. *et al.* Activation of CD4+ T lymphocytes improves wound healing and survival after experimental myocardial infarction in mice. *Circulation* **125**, 1652-1663, doi:10.1161/circulationaha.111.044164 (2012).

22 Zhang, M. *et al.* Identification of the target self-antigens in reperfusion injury. *The Journal of experimental medicine* **203**, 141-152, doi:10.1084/jem.20050390 (2006).

23 Dobaczewski, M., Xia, Y., Bujak, M., Gonzalez-Quesada, C. & Frangogiannis, N. G. CCR5 signaling suppresses inflammation and reduces adverse remodeling of the infarcted heart, mediating recruitment of regulatory T cells. *The American journal of pathology* **176**, 2177-2187, doi:10.2353/ajpath.2010.090759 (2010).

24 Matsumoto, K. *et al.* Regulatory T lymphocytes attenuate myocardial infarction-induced ventricular remodeling in mice. *International heart journal* **52**, 382-387 (2011).

25 Tang, T. T. *et al.* Regulatory T cells ameliorate cardiac remodeling after myocardial infarction. *Basic research in cardiology* **107**, 232, doi:10.1007/s00395-011-0232-6 (2012).

26 Mausner-Fainberg, K. *et al.* The effect of HMG-CoA reductase inhibitors on naturally occurring CD4+CD25+ T cells. *Atherosclerosis* **197**, 829-839, doi:10.1016/j.atherosclerosis.2007.07.031 (2008).

27 Zhang, D. *et al.* Effect of oral atorvastatin on CD4+CD25+ regulatory T cells, FoxP3 expression, and prognosis in patients with ST-segment elevated myocardial infarction before primary percutaneous coronary intervention. *Journal of cardiovascular pharmacology* **57**, 536-541, doi:10.1097/FJC.0b013e318211d016 (2011).

28 Platten, M. *et al.* Blocking angiotensin-converting enzyme induces potent regulatory T cells and modulates TH1- and TH17-mediated autoimmunity. *Proceedings of the National Academy of Sciences of the United States of America* **106**, 14948-14953, doi:10.1073/pnas.0903958106 (2009).

29 Malek, T. R. & Bayer, A. L. Tolerance, not immunity, crucially depends on IL-2. *Nature reviews. Immunology* **4**, 665-674, doi:10.1038/nri1435 (2004).

30 Wing, K. & Sakaguchi, S. Regulatory T cells exert checks and balances on self tolerance and autoimmunity. *Nature immunology* **11**, 7-13, doi:10.1038/ni.1818 (2010).

31 Malek, T. R. The biology of interleukin-2. *Annual review of immunology* **26**, 453-479, doi:10.1146/annurev.immunol.26.021607.090357 (2008).

32 Dinh, T. N. *et al.* Cytokine therapy with interleukin-2/anti-interleukin-2 monoclonal antibody complexes expands CD4+CD25+Foxp3+ regulatory T cells and attenuates development and progression of atherosclerosis. *Circulation* **126**, 1256-1266, doi:10.1161/circulationaha.112.099044 (2012).

33 Foks, A. C. *et al.* Differential effects of regulatory T cells on the initiation and regression of atherosclerosis. *Atherosclerosis* **218**, 53-60, doi:10.1016/j.atherosclerosis.2011.04.029 (2011).

34 Koreth, J. *et al.* Interleukin-2 and regulatory T cells in graft-versus-host disease. *The New England journal of medicine* **365**, 2055-2066, doi:10.1056/NEJMoa1108188 (2011).

35 Matsuoka, K. *et al.* Low-dose interleukin-2 therapy restores regulatory T cell homeostasis in patients with chronic graft-versus-host disease. *Science translational medicine* **5**, 179ra143, doi:10.1126/scitranslmed.3005265 (2013).

36 Saadoun, D. *et al.* Regulatory T-cell responses to low-dose interleukin-2 in HCV-induced vasculitis. *The New England journal of medicine* **365**, 2067-2077, doi:10.1056/NEJMoa1105143 (2011).

37 Rosenzwajg, M. *et al.* Low-dose interleukin-2 fosters a dose-dependent regulatory T cell tuned milieu in T1D patients. *Journal of autoimmunity* **58**, 48-58, doi:10.1016/j.jaut.2015.01.001 (2015).

38 Klatzmann, D. & Abbas, A. K. The promise of low-dose interleukin-2 therapy for autoimmune and inflammatory diseases. *Nature reviews. Immunology* **15**, 283-294, doi:10.1038/nri3823 (2015).

39 Elkhawad, M. *et al.* Effects of p38 mitogen-activated protein kinase inhibition on vascular and systemic inflammation in patients with atherosclerosis. *JACC. Cardiovascular imaging* **5**, 911-922, doi:10.1016/j.jcmg.2012.02.016 (2012).

40 Fayad, Z. A. *et al.* Safety and efficacy of dalcetrapib on atherosclerotic disease using novel non-invasive multimodality imaging (dal-PLAQUE): a randomised clinical trial. *Lancet (London, England)* **378**, 1547-1559, doi:10.1016/s0140-6736(11)61383-4 (2011).

41 Tarkin, J. M., Joshi, F. R. & Rudd, J. H. PET imaging of inflammation in atherosclerosis. *Nature reviews. Cardiology* **11**, 443-457, doi:10.1038/nrcardio.2014.80 (2014).

42 Maki-Petaja, K. M. *et al.* Anti-tumor necrosis factor-alpha therapy reduces aortic inflammation and stiffness in patients with rheumatoid arthritis. *Circulation* **126**, 2473-2480, doi:10.1161/circulationaha.112.120410 (2012).

43 Tahara, N. *et al.* Simvastatin attenuates plaque inflammation: evaluation by fluorodeoxyglucose positron emission tomography. *Journal of the American College of Cardiology* **48**, 1825-1831, doi:10.1016/j.jacc.2006.03.069 (2006).

44 Figueroa, A. L. *et al.* Measurement of arterial activity on routine FDG PET/CT images improves prediction of risk of future CV events. *JACC. Cardiovascular imaging* **6**, 1250-1259, doi:10.1016/j.jcmg.2013.08.006 (2013).

45 Marnane, M. *et al.* Carotid plaque inflammation on 18F-fluorodeoxyglucose positron emission tomography predicts early stroke recurrence. *Annals of neurology* **71**, 709-718, doi:10.1002/ana.23553 (2012).

46 Bucerius, J. *et al.* Position paper of the Cardiovascular Committee of the European Association of Nuclear Medicine (EANM) on PET imaging of atherosclerosis. *European journal of nuclear medicine and molecular imaging* **43**, 780-792, doi:10.1007/s00259-015-3259-3 (2016).

47 Rudd, J. H. *et al.* Atherosclerosis inflammation imaging with 18F-FDG PET: carotid, iliac, and femoral uptake reproducibility, quantification methods, and recommendations. *Journal of nuclear medicine : official publication, Society of Nuclear Medicine* **49**, 871-878, doi:10.2967/jnumed.107.050294 (2008).

48 Tawakol, A. *et al.* Effect of treatment for 12 weeks with rilapladib, a lipoprotein-associated phospholipase A2 inhibitor, on arterial inflammation as assessed with 18F-fluorodeoxyglucose-positron emission tomography imaging. *Journal of the American College of Cardiology* **63**, 86-88, doi:10.1016/j.jacc.2013.07.050 (2014).

49 O'Donoghue, M. L., Braunwald, E., White, H. D. & et al. Effect of darapladib on major coronary events after an acute coronary syndrome: The solid-timi 52 randomized clinical trial. *JAMA* **312**, 1006-1015, doi:10.1001/jama.2014.11061 (2014).

50 Schwartz, G. G. *et al.* Effects of dalcetrapib in patients with a recent acute coronary syndrome. *The New England journal of medicine* **367**, 2089-2099, doi:10.1056/NEJMoa1206797 (2012).

51 White, H. D. *et al.* Darapladib for preventing ischemic events in stable coronary heart disease. *The New England journal of medicine* **370**, 1702-1711, doi:10.1056/NEJMoa1315878 (2014).

26 Appendices

26.1 Appendix 1 – Symptoms and signs documented as AEs in the LILACS trial

(MHRA reference 24551/0029/001-0001; REC reference 17/NW/0012; ClinicalTrials.gov Identifier NCT03113773)

AE description	Frequency	Severity	Relatedness as determined by the blinded investigator
Chest infection	2	mild	unrelated
Chest tightness	1	mild	unrelated
Diarrhoea	1	mild	unrelated
Dizziness	1	moderate	unrelated
Free T4 low	1	mild	unrelated
Gastro oesophageal reflux	1	mild	unrelated
GI disturbance	1	mild	unrelated
Groin thrush	1	mild	unrelated
Muscle ache	1	mild	unrelated
Productive cough post op.	1	mild	unrelated
Raised BNP	1	mild	unrelated
Raised troponin	1	mild	unrelated
Right arm ache	1	mild	unrelated
Right hand petechiae	1	mild	unrelated
Right scapula ache	1	mild	unrelated
Right ventricular impairment	1	mild	unrelated
TSH low	1	mild	unrelated
Vomiting	1	moderate	unrelated
Chest pain	5	mild	unlikely 2, unrelated 3
Dizziness	3	mild	unlikely 1, unrelated 2
Dry cough	2	mild	unlikely 1, unrelated 1
Angina	2	mild	unlikely
Back pain	1	mild	unlikely
Blocked sinuses	1	mild	unlikely
Fall	1	mild	unlikely
Finger pain	1	mild	unlikely
Gastro oesophageal reflux	1	moderate	unlikely
Gout right toe	1	mild	unlikely
Inflamed haematoma - right forearm	1	mild	unlikely
Nasal congestion	1	mild	unlikely
Non-productive cough	1	mild	unlikely
Pyrexia	1	mild	unlikely
Sinusitis	1	mild	unlikely
Hand joint pain	1	moderate	probably
Injection site adverse reaction	93	mild	probably
Injection site bruise	3	mild	probably
Injection site nodule	12	mild	probably

**Injection site pruritus**	2	mild	probably
**Leg ache (bilateral)**	1	mild	probably
**Fatigue**	10	mild	possibly 6, probably 4
**Tiredness**	4	mild	possibly 2, unlikely 2
**Isolated episode pyrexia**	3	mild	possibly 2, unlikely 1
**Headache**	4	mild	possibly 2, probably 1, unlikely 1
**Body ache**	3	mild	possibly 2, probably 1
**Breathlessness**	2	mild	possibly 1, unrelated 1
**Headache**	2	moderate	possibly 1, unrelated 1
**Flu like syndrome**	9	mild	possibly 1, probably 8
**Elevated CRP**	1	mild	possibly
**Knee pain (intermittent at night)**	1	mild	possibly
**Sore throat**	2	mild	possibly

26.2 Appendix 2 - Safety reporting flow-chart

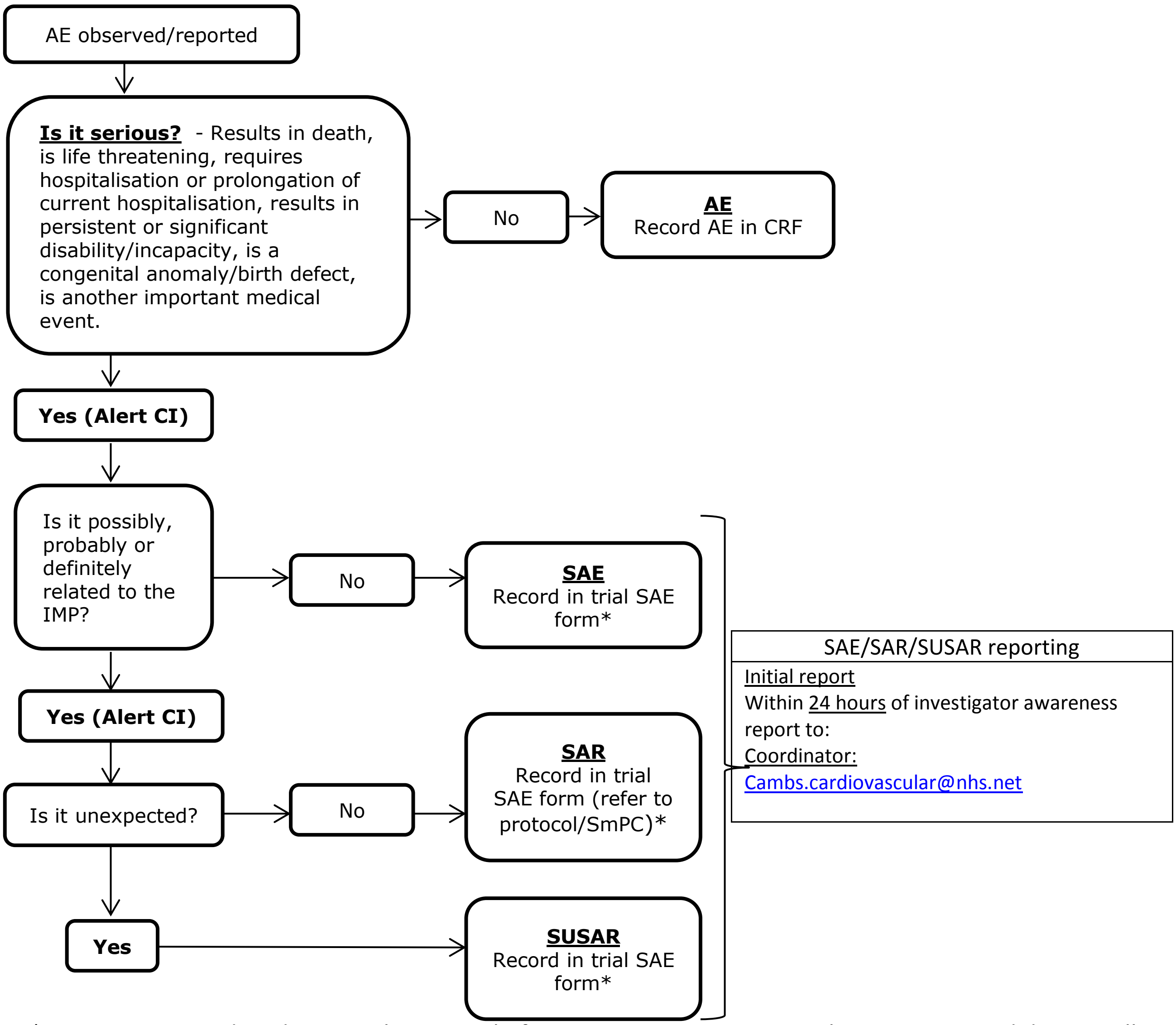

*First 10 patients dosed: A cumulative total of 3 events triggers IDMC; trial recruitment and dosing will halt. After the tenth (10th) patient has started treatment and 30% patients experience pre-specified events (see protocol section 9.6/ SmPC RSI in 4.8 for specified events) trial recruitment and dosing will halt.

SUSAR expedited reporting
The Chief Investigator must report a SUSAR to the Sponsor, REC and MHRA within statutory timelines. Each SUSAR requires the entry of relevant data and information by the Chief Investigator into the eSUSAR reporting system. A copy of this report should be provided to the CCTU PV team, collating these on behalf of the sponsor.

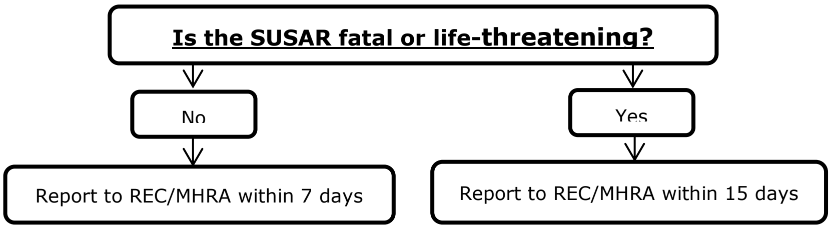

Clinical Trial Protocol

Trial Title: **Low-dose interleukin-2 for the reduction of vascular inflammation in Acute Coronary Syndromes (IVORY)**

Protocol Number: IVORY

EudraCT Number: 2017-005130-27

Investigational Product: Aldesleukin (IL-2)

Chief Investigator: Dr Joseph Cheriyan

CI Address: Cambridge University Hospitals NHS Foundation Trust,
Box 98, Level 3,
ACCI Building,
Hills Road,
Cambridge,
CB2 0QQ

Telephone: 01223 256653

Trial Sponsor: Cambridge University Hospitals NHS Foundation Trust and the University of Cambridge

SAE Reporting: IVORY Clinical Trial Coordinator

Email cambs.cardiovascular@nhs.net

Protocol Signatures

I give my approval for the attached protocol entitled Low-dose interleukin-2 for the reduction of vascular inflammation in Acute Coronary Syndromes (IVORY) dated 16May2023.

Chief Investigator

Name: Dr Joseph Cheriyan

Signature: ____

Date:

Site Signatures

I have read the attached protocol entitled "Low-dose interleukin-2 for the reduction of vascular inflammation in Acute Coronary Syndromes (IVORY)" dated 16May2023 and agree to abide by all provisions set forth therein.

I agree to comply with the conditions and principles of Good Clinical Practice as outlined in the European Clinical Trials Directives 2001/20/EC and 2005/28/EC, the Medicines for Human Use (Clinical Trials) Regulations 2004 (SI 2004/1031) and any subsequent amendments of the clinical trial regulations, the Sponsor's SOPs, and other regulatory requirements as amended.

I agree to ensure that the confidential information contained in this document will not be used for any other purpose other than the evaluation or conduct of the clinical investigation without the prior written consent of the Sponsor

Principal Investigator

Name:

Signature: ____

Date: ____________________________

1 Protocol Contributors

Chief Investigator:	
Dr Joseph Cheriyan	Cambridge University Hospitals NHS Foundation Trust and Division of Experimental Medicine & Immunotherapeutics, Dept of Medicine, Univ. of Cambridge, Box 98, Level 3, ACCI Building, Hills Road, Cambridge, CB2 0QQ<br>Tel.: 01223 256653
Sub-Investigators / Collaborators:	
Professor Ziad Mallat	Division of Cardiovascular Medicine, Dept of Medicine, Level 2, Heart and Lung Research Institute, Biomedical Campus, Papworth Road, Trumpington, Cambridge CB2 0AY
Dr Stephen Hoole	Royal Papworth Hospital NHS Foundation Trust, Cambridge Biomedical Campus, Cambridge, CB2 0AY
Dr James Rudd	Division of Cardiovascular Medicine, Dept of Medicine, Level 2, Heart and Lung Research Institute, Biomedical Campus, Papworth Road, Trumpington, Cambridge CB2 0AY
Dr Rouchelle Sriranjan	Division of Cardiovascular Medicine, Dept of Medicine, Level 2, Heart and Lung Research Institute, Biomedical Campus, Papworth Road, Trumpington, Cambridge CB2 0AY
Dr Michalis Kostapanos	Division of Experimental Medicine & Immunotherapeutics, Dept of Medicine, Univ. of Cambridge, Addenbrooke's Hospital, Hills Road, Cambridge, CB2 0QQ
Dr Tian Zhao	Division of Cardiovascular Medicine, Dept of Medicine, Level 2, Heart and Lung Research Institute, Biomedical Campus, Papworth Road, Trumpington, Cambridge CB2 0AY
Dr Jason Tarkin	Division of Cardiovascular Medicine, Dept of Medicine, Level 2, Heart and Lung Research Institute, Biomedical Campus, Papworth Road, Trumpington, Cambridge CB2 0AY

Trial coordination:	
Heike Templin	Cardiovascular Trials Office, Cambridge Clinical Trials Unit, Box 401, Cambridge University Hospitals NHS Foundation Trust, Hills Road, Cambridge, CB2 0QQ<br>Email: heike.templin@nhs.net
Trial statistician:	
Dr Simon Bond	Cambridge Clinical Trials Unit, Box 401, Cambridge University Hospitals NHS Foundation Trust, Hills Road, Cambridge, CB2 0QQ
Trial pharmacist:	
Robyn Staples	Central Pharmacy Level 1, Box 55 Cambridge University Hospitals NHS Foundation Trust, Hills Road, Cambridge, CB2 0QQ

Amendment History

Version No.	History	Date
v1.0	Final Protocol	08 May 2019
v1.1	Incorporating REC/HRA comments	15 Jul 2019
v2.0	Incorporation MHRA feedback on initial submission	Aug 2019
V3.0	Changes to endpoints, visit schedule	01 March 2021
V4.0	Changes to exploratory endpoints, changes to angiograms, addition of optional stool sample	08Jun2022
V4.1	Clarification of basis on which sample size calculation was made, correction of typographical errors for pre-bloods in induction phase of dosing	01Nov2022
V4.2	Deletion to sentences with transcription errors, addition of a reference to secondary endpoint 1, correction of contact details of investigators	16May2023

2 Table of Contents

Protocol Signatures 2
1 Protocol Contributors 3
2 Table of Contents 5
3 Abbreviations 9
4 Trial Figures 11
4.1 Trial Design Figure 11
4.2 Trial Design Per Patient 12
5 Introduction 13
5.1 Background 13
6.1 Aldesleukin 15
6.2 Clinical use of IL-2 15
7 Rationale for Trial 18
7.1 Hypotheses 20
8 Trial Design 20
8.1 Statement of Design 20
8.2 Number of Centres 20
8.3 Number of Patients 20
8.4 Trial Duration 21
8.5 Trial Objectives 21
8.5.1 Primary objective 21
8.5.2 Secondary objectives 21
8.5.3 Exploratory objectives 21
8.6 Trial endpoints 21
8.6.1 Primary endpoint: 21
8.6.2 Secondary endpoints: 22
8.6.3 Exploratory endpoints: 22
9 Selection and withdrawal of patients 22
9.1 Inclusion Criteria 22
9.2 Exclusion Criteria 23
9.3 Treatment Assignment and Randomisation Number 24
9.4 Method of Blinding 24
9.5 Patient Withdrawal Criteria 25
9.5.1 Liver withdrawal criteria 25
9.5.2 Renal withdrawal criteria 25
9.5.3 Cardiac withdrawal criteria 25
9.5.4 General withdrawal criteria 26
9.5.5 Management of withdrawal 26
9.6 Trial stopping criteria 27

10 Trial Treatments 27
10.1 Dosage schedules 27
10.1.1 Route of Administration and Maximum dosage allowed 27
10.1.2 Maximum duration of treatment of a patient 28
10.2 Presentation of the drug 28
10.3 Known drug reactions & interaction with other therapies 28
10.4 Dosage modifications 28
10.5 Legal status of the drug 28
10.6 Drug storage and supply 28
10.7 Accountability 29
10.8 Concomitant Therapy 29
10.9 Emergency unblinding 29
11 Procedures and assessments 29
11.1 Patient identification 30
11.2 Consent 30
11.3 Screening evaluation 30
11.3.1 Screening Assessments (**Visit 1**) Day -14 to 0 30
11.3.2 Patient Randomisation 31
11.4 Trial assessments 31
11.4.1 ^{18}F-FDG PET/CT Scan 1 (**Visit 2**) Day -13 to 0 31
11.4.2 Induction phase – **(Visit 3-7)** Day 1-5 32
11.4.3 Maintenance Phase – (**Visits 8 -14)** Day 12-54 33
11.4.4 Post-dose PET/CT – (**Visit 15**) Day 61 34
11.4.5 Follow-up – (**Visit 16**) Day 82 35
11.5 Schedule of Assessments 36
11.6 Trial specific procedures 38
11.7 End of Trial Participation 39
11.8 Trial restrictions 40
12 Assessment of Safety 40
12.1 Definitions 40
12.1.1 Adverse event (AE) 40
12.1.2 Adverse reaction to an investigational medicinal product (AR) 41
12.1.3 Unexpected adverse reaction 41
12.1.4 Serious adverse event or serious adverse reaction (SAE / SAR) 41
12.1.5 Suspected Unexpected Serious Adverse Reaction (SUSAR) 41
12.1.6 Reference Safety Information (RSI) 41
12.2 Expected Adverse Reactions/Serious Adverse Reactions (AR /SARs) 42
12.2.1 Injection site reaction 43
12.3 Expected Adverse Events/Serious Adverse Events (AE/SAE) 43
12.3.1 Hospital admissions 43

12.3.2 Events commonly occurring in patients with ACS 44
12.4 Evaluation of adverse events 44
12.4.1 Assessment of seriousness 44
12.4.2 Assessment of causality 44
12.4.3 Clinical assessment of severity 45
12.4.4 Recording of adverse events 45
12.5 Reporting serious adverse events 45
12.6 Reporting of Suspected Unexpected Serious Adverse Reactions (SUSARs) 45
12.7 Pregnancy Reporting 47
13 Toxicity – Emergency Procedures 47
14 Evaluation of results 47
14.1 Response criteria 47
15 Storage and Analysis of Samples 47
16 Statistics 48
16.1 Statistical methods 48
16.2 Interim analyses 48
16.3 Number of Patients to be enrolled 48
16.3.1 Safety Population 49
16.3.2 Analysis Population 49
16.4 Procedure to account for missing or spurious data 49
16.5 Definition of the end of the trial 49
17 Data handling and record keeping 49
17.1 CRF 49
17.2 Source Data 50
17.3 Data Protection & Patient Confidentiality 50
18 Trial Management Group 50
19 Independent Data Monitoring Committee 50
20 Ethical & Regulatory considerations 51
20.1 Ethical committee review 51
20.2 Regulatory Compliance 51
20.3 Protocol Amendments 51
20.4 Peer Review 51
20.5 Declaration of Helsinki and Good Clinical Practice 51
20.6 GCP Training 52
21 Sponsorship, Financial and Insurance 52
22 Monitoring, Audit & Inspection 52
23 Protocol Compliance and Breaches of GCP 52
24 Publications policy 52
25 References 52
26 Appendices 56

26.1 Appendix 1 – Symptoms and signs documented as AEs in the LILACS trial 56
26.2 Appendix 2 - Safety reporting flow-chart 58

3 Abbreviations

AE/AR	Adverse event/Adverse Reaction
ACS	Acute Coronary Syndrome
ALP	Alkaline phosphatase
ALT	Alanine aminotransferase
AST	Aspartate aminotransferase
BNP	B-type Natriuretic Peptide
CA	Competent Authority
CABG	Coronary artery bypass graft
CCTU	Cambridge Clinical Trials Unit
CI	Chief investigator
CNS	Central nervous system
CRF	Case Report Form
CT	Computed Tomography
CTA	Computed Tomography Carotid Angiogram
CTCA	Computed Tomography Coronary Angiogram
CV	cardiovascular
DCCT	Diabetes Control and Complications Trial
DMC	Data Monitoring Committee
DSUR	Development Safety Update Report
ECG	Electrocardiogram
FDG - PET/CT	Fluorodeoxyglucose - Positron emission tomography/ computed tomography
GCP	Good Clinical Practice
GP	General Practitioner
hs-CRP	High-Sensitivity C-Reactive Protein
ICF	Informed Consent Form
IFCC	International Federation of Clinical Chemistry and Laboratory Medicine
IHD	Ischaemic heart disease
IMP	Investigational Medicinal Product
ISR	Injection site reaction
IU	International Unit
MHRA	Medicines and Healthcare products Regulatory Agency
NSTEMI	Non-ST Elevation Myocardial Infarction
PBMC	Peripheral Blood Mononuclear Cell assay
PCI	Percutaneous coronary intervention
PET	Positron Emission Tomography
PIS	Patient Information Sheet
QTcB	Corrected QT using Bazett's formula
R&D	Research and Development
RA	Regulatory Agency
REC	Research Ethics Committee
RSI	Reference Safety Information
SAE/SAR	Serious Adverse Event/Serious Adverse Reaction
SmPC	Summary of Product Characteristics
STEMI	ST elevation myocardial infarction
ST	ECG parameter
SUSAR	Suspected Unexpected Serious Adverse Reaction
SUV	Standardized Uptake Value
TBR	Tissue-to-blood ratio
TMG	Trial Management Group

TnI	Troponin I
Treg	Regulatory T cells
TSH	Thyroid Stimulating Hormone
TTE	Transthoracic echocardiogram
ULN	Upper Limit of Normal

4 Trial Figures

4.1 Trial Design Figure

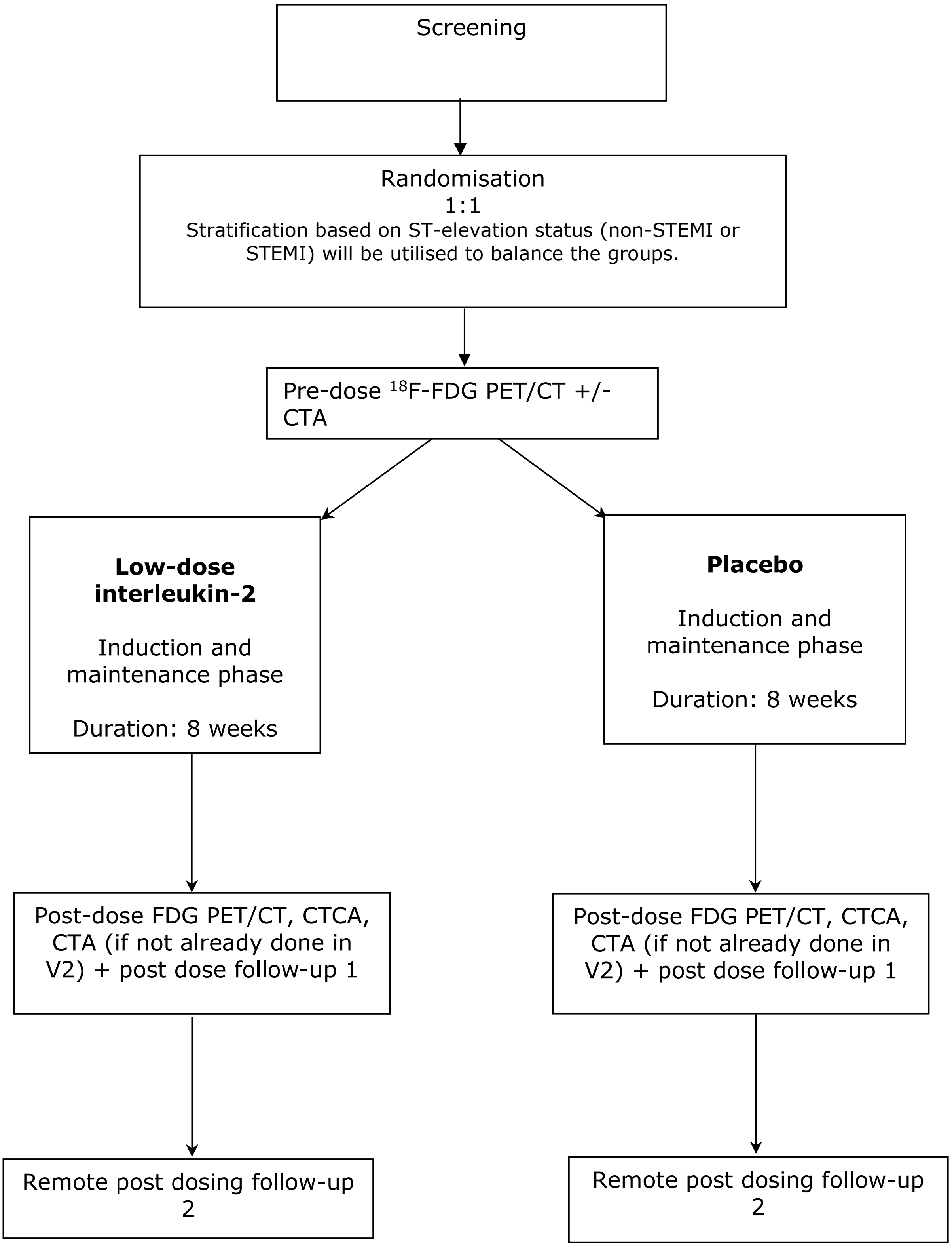

4.2 Trial Design Per Patient

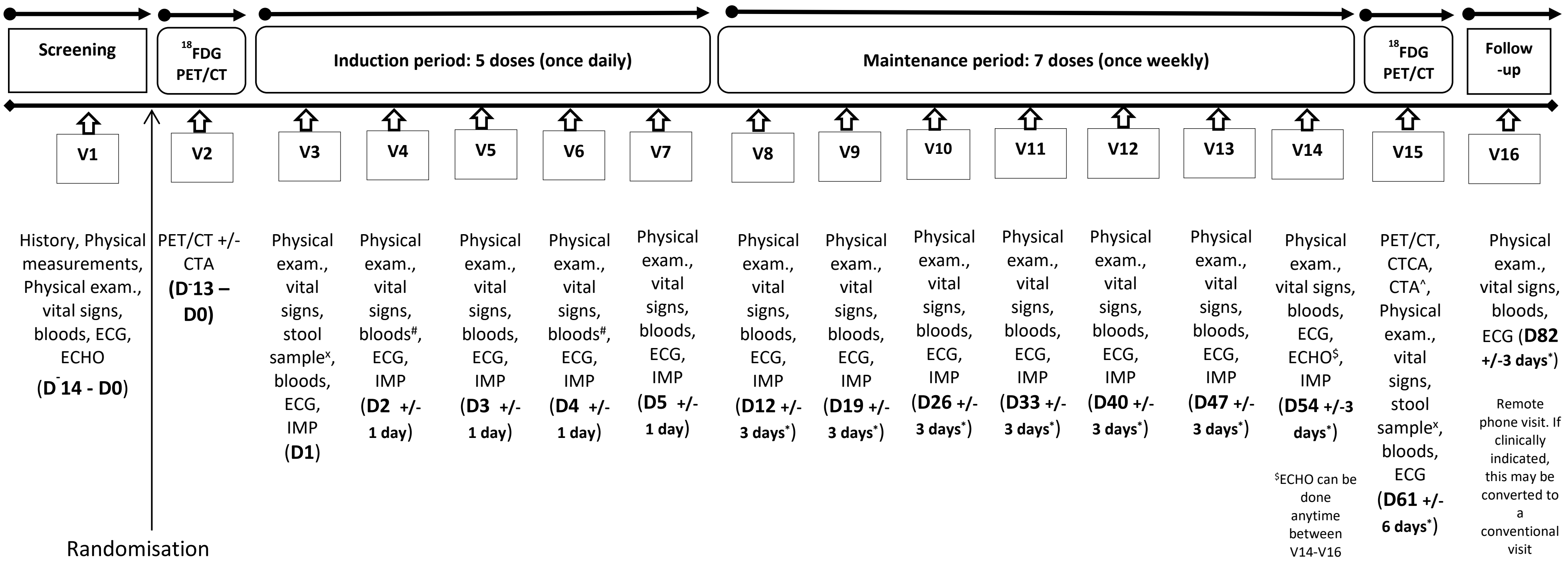

^x Stool sample – this is optional
^# Bloods will only be done in these visits if clinically deemed necessary
* +/3 days from the intended previous visit
^ CTA if not done in V2 will be done in V15

5 Introduction

5.1 Background

Acute coronary syndromes (ACS) result from coronary plaque(s) disruption, which initiates a thrombotic process leading to partial or complete obstruction of the vessel lumen with subsequent myocardial ischaemia and necrosis[1,2]. The mainstay of treatment is currently focussed on the re-establishment and maintenance of coronary artery patency using anti-platelets and anticoagulants with or without mechanical dilatation and stenting of the culprit artery[1,2]. Despite important advances in management, ACS still carries a risk of substantial morbidity and mortality[1]. The improved efficacy of novel anti-platelet and anticoagulant agents have been limited by increased risk of haemorrhagic events[3,4]. Thus, future breakthroughs in management are most likely to arise from targeting other relevant pathophysiological pathways. Particularly, we believe that the immune response is an important process that has been neglected in the management of patients with ACS.

5.1.1 Regulatory T cell responses stabilise atherosclerotic plaques and are defective in ACS

Inflammation plays a central role in the pathophysiology of ACS. Circulating levels of C-reactive protein (CRP) are elevated in ACS patients and are associated with adverse outcome[5]. White blood cells display increased inflammation and activation[6,7]. The culprit coronary plaque is highly inflammatory too[8] and the prevalence of multiple coronary inflammatory plaques is significantly higher in patients with ACS compared to stable coronary patients[7]. Recent experimental studies also suggest an inflammation-driven acceleration of the development of atherosclerotic lesions following experimental myocardial necrosis[9]. Genetic studies imply that both innate and adaptive immune responses triggered by various antigens, including (modified) lipoproteins, contribute to plaque development, progression and disruption[10,11]. Besides mononuclear cell and neutrophil activation in the circulation, several studies reported a perturbation of the T cell repertoire in ACS patients[12] with expansion of an effector and activated T cell subset[13] which is at least in part directed to antigens contained in the disrupted plaque(s)[14]. The previous finding that regulatory T cell (Treg)-mediated immunity tames experimental atherosclerosis and reduces plaque inflammation in mice[13] boosted experimental and translational research into the role of Treg cells in cardiovascular diseases. A particularly interesting aspect of the T cell response in ACS patients is the presence of an imbalance between T effector and Treg cells. In contrast to the effector T cell compartment activation, the percentage and/or function of circulating Tregs appear to be significantly decreased in the setting of ACS[15-18]. Whether this is related to a global Treg defect in these patients, to a defective mobilisation, increased susceptibility to cell death[19] or increased recruitment of Treg cells into sites of unstable coronary arteries is still unknown. Nevertheless, the results strongly suggest an imbalance of the adaptive immune response with potentially important consequences on the progression and destabilisation of coronary plaques. Interestingly, in 700 patients from the Malmö Diet and Cancer Study, low levels of circulating baseline CD4+Foxp3+ Treg cells were associated with an increased risk for the development of future acute coronary events[20], suggesting that defects in Treg-mediated immunity may predispose individuals to increased plaque vulnerability.

5.1.2 Tregs improve heart remodelling after myocardial ischaemic injury

Treg cells not only regulate antigen-specific immunity but also dampen innate immune responses in the local microenvironment through bystander immune suppression, suggesting that they can be involved in modulating the response to post-ischaemic injury. Furthermore, the ischaemic and necrotic myocardial tissue may expose self-

antigens for recognition by the immune system, which may lead to antigen-specific (autoimmune) adaptive responses[21,22]. Recent studies indicate that CD4+ T cells, and more particularly Treg cells, are important for the control of post-ischemic immune responses and the promotion of myocardial healing[21,23-25]. Inhibition of Treg recruitment to the site of myocardial injury resulted in excessive post-ischaemic inflammation, matrix degradation and adverse remodelling[23]. In contrast, in vivo expansion of Treg cells or their therapeutic activation by superagonistic anti-CD28 mAbs attenuated left ventricular remodelling and improved cardiac function[24,25]. Altogether, the studies point to a protective role for Treg immunity in coronary artery disease, limiting atherosclerotic plaque development and vulnerability, and taming the deleterious consequences of post-ischaemic injury. We therefore hypothesise that expansion of Treg cells in patients with ACS dampens the activation of the immune response and promotes both plaque and myocardial healing.

5.1.3 Treg cell expansion in ACS patients

Interestingly, a few therapeutic strategies, which are known for their athero- and cardio-protective effects in patients with ACS, have been shown to promote Treg-dependent immuno-regulatory responses both in experimental and clinical settings. This is the case for statins[26,27] and ACE inhibitors[28]. However, these therapies do not appear to be sufficient enough to promote full recovery of Treg levels and functions in patients with ACS, suggesting the need for alternative strategies. Direct supplementation with exogenous polyclonal Treg cells is not an option, particularly in the acute setting. Hence, there is a need for a simple, feasible and highly effective strategy to promote Treg cells at the acute phase of plaque destabilisation and myocardial infarction. We hypothesise that this can be achieved through subcutaneous administration of low doses of interleukin-2 (IL-2).

5.1.4 Low dose IL-2 and Treg cell expansion

IL-2 supplementation appears to be an attractive therapeutic option for several reasons. IL-2 plays a key role in Treg cell development, expansion, survival and suppressive function[29,30]. Deficiency of IL-2 or IL-2 receptor in mice greatly compromises Treg development and promotes autoimmune responses[31]. Supplementation of mice prone to atherosclerosis with IL-2 substantially increases Treg levels and significantly limits plaque development and inflammation[32,33]. Treg cells show a much lower threshold response to IL-2 receptor signalling compared to effector T cells. This led to the hypothesis that, in contrast to high dose IL-2 designed to activate T effector cells in cancer, supplementation with low doses of IL-2 in the setting of T cell-mediated immune diseases may selectively promote the expansion of Treg cells at the expense of T effector cells, thereby limiting harmful immune responses. Remarkably, this hypothesis was recently confirmed in a few human clinical studies in the setting of graft-versus-host disease[34,35], hepatitis C virus-induced vasculitis[36], or systemic lupus erythematosus[20]. In those studies, administration of low doses of IL-2 (daily administration of $0.3x10^6$ to $3x10^6$ IU IL-2 per square meter of body-surface area for 8 weeks, repetitive 5-day courses of $1x10^6$ to $3x10^6$ IU IL-2, or 3 cycles of $1X10^6$ IU IL-2 every other day for 2 weeks followed by a 2-week break in treatment) led to a rapid and marked expansion of the circulating pool of Treg cells, which were increased by a factor of 2 to 20 without affecting the pool of conventional CD4+ T (i.e. T effector) cells. The expanded Tregs retained potent suppressive functions and the treatment was associated with a reduction in the inflammatory response and a concomitant clinical improvement in a substantial proportion of patients. Treatment with low dose IL-2 was safe and no adverse effects were reported. This strategy is currently being adapted and tested in various disease settings, where Treg cell promotion is believed to be of potential therapeutic benefit[35-37].

6.1 Aldesleukin

Aldesleukin is commercially available and is licensed for the treatment of metastatic renal cell carcinoma in the UK. A single vial unit contains 22 x 10^6 IU aldesleukin. Aldesleukin is produced by recombinant DNA technology using an *Escherichia coli* strain which contains a genetically engineered modification of the human IL-2 gene. The administration of aldesleukin is by either intravenous or subcutaneous routes. Following short intravenous infusion, its pharmacokinetic profile is typified by high plasma concentrations, rapid distribution into the extravascular space and a rapid renal clearance. The recommended doses for continuous infusion and subcutaneous injection (as detailed in the SmPC) are repeated cycles of 18 x 10^6 IU per m^2 per 24-hours for 5 days and repeated doses of 18 x 10^6 IU respectively. Peak plasma levels are reached in 2-6 hours after subcutaneous administration, with bioavailability of aldesleukin ranging between 31 – 47%. The process of absorption and elimination of subcutaneous aldesleukin is described by a one-compartment model, with a 45-minute absorption half-life and a 3-5 hour elimination half-life. For the purposes of this trial, (see section 6.1.3), the drug will be administered to the patients by subcutaneous injection.

6.2 Clinical use of IL-2

The use of IL-2 in low doses as a means of expanding Treg cell populations in autoimmune and allo-inflammatory conditions has been explored. ClinicalTrials.gov currently lists 154 clinical studies using low-dose IL-2 (Figure 3). There have been several completed and published human clinical trials on low dose IL-2 therapy in autoimmune and allo-immune diseases. A few examples are shown in Table 1[38]. In these studies, patients have received at least 1 dose of IL-2 ranging from 0.3x10^6 IU – 3.0x10^6 IU. Interestingly, there was a low rate of adverse events (AEs) in all of the studies with the most commonly reported AEs being injection site reactions, fatigue, fever, nausea and vomiting. A low percentage of serious adverse events (SAEs) were recorded in a GVHD-risk study and these SAEs included haemorrhage (CNS), anorexia, and infection (colitis).

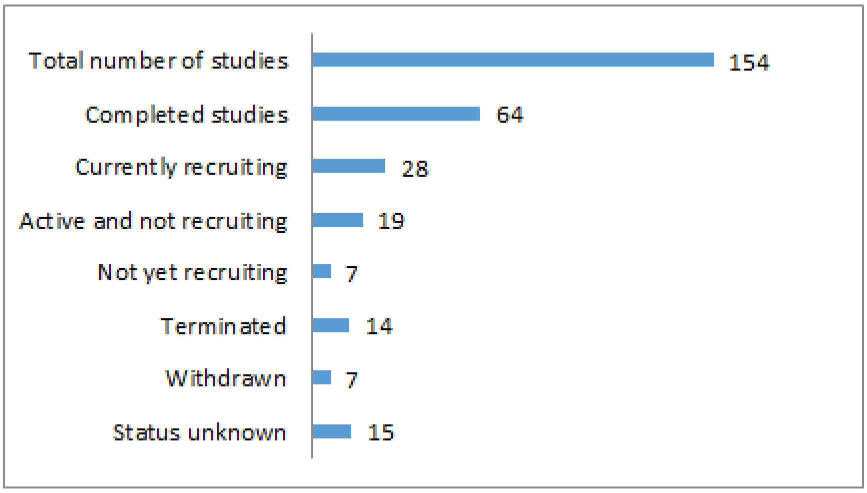

Figure 3: Listed low-dose IL-2 studies on clinicaltrials.gov

**Trial** (*reference*)	**Disease**	**N**	**IL-2 dose (daily, MIU)**	**Course**	**Total IL2 (MIU)** (days)	**Main biological findings**	**Clinical findings**
**Low-dose interleukin-2 treatment selectively modulates CD4(+) T cell subsets in patients with systemic lupus erythematosus**. *He, J; Zhang, I et al. Nat Med 2016, Sep;22(9):991-3. Doi: 10.1038/nm.4148.*	SLE	38	1	3 cycles of: Single dose every other day for 2 weeks, 2 weeks break.	**21** (21)	Increase of Tregs, THF and TH17, but not TH1.	Marked reduction of disease activity in SLE patients.
**Evaluation of clinical efficacy and immunological responses after IL-2 therapy in HCV-related vasculitis patients** *Saadoun, D. et al. Regulatory T-cell responses to low-dose interleukin-2 in HCV-induced vasculitis. N Engl J Med 365, 2067–77 (2011)*	Hepatitis C Virus induced vasculitis	10	1.5 then 3	Four 5-day courses	**52.5** (60)	Increase of CD4+Tregs (x3) and CD8+ Tregs (x8). Increase of NK and cd56 bright NKs; Decrease of B cells.	Grade 1 & 2 AEs; no vasculitis or HCV-replication flares; improvement of the vasculitis in 8/10 patients.
**Dose-effect relationship of low-dose IL-2 in type 1 diabetes** *Hartemann, A. et al. Low-dose interleukin 2 in patients with type 1 diabetes: a phase 1/2 randomised, double-blind, placebo-controlled trial. Lancet Diabetes Endocrinol 1, 295–305 (2013). Rosenzwajg, M. et al. Low-dose interleukin-2 fosters a dose-dependent regulatory T cell tuned milieu in T1D patients. J. Autoimmun. (2015). doi:10.1016/j.jaut.2015.01.001*	Type 1 Diabetes	24	0.3,1,3	One 5-day course	**1.5,5,15** (15)	Dose-dependent increase of CD4+ Tregs and CD8+ Tregs; Dose dependent decrease of B cells; No effects on NK or Teffs. Imprinting of a dose-dependent regulatory-tuned milieu.	Good tolerance; Grade 1 and 2 AEs; more days with AEs in the placebo group than in any other group.
**Dose Finding Study of IL-2 at Ultra-low Dose in Children with Recently Diagnosed Type 1 Diabetes** *NCT01862120*	Type 1 Diabetes	24	0.25,0.5,1	One 5-day course followed by 1 injection every 2 weeks	**7.5,15,30** (365)	Double blind, still in progress.	Good long-term tolerance on the first 18 patients treated for at least 6 months.
**Effects of Low-dose Recombinant IL-2 to Promote Treg in Alopecia Areata** *Castela, E. et al. Effects of low-dose recombinant interleukin 2 to promote T-regulatory cells in alopecia areata. JAMA Dermatol 150, 748–51 (2014).*	Alopecia Aerata	5	1.5 then 3	Four 5-day courses	**52.5** (60)	Increase of Tregs and decrease of CD8+ Teffs in scalp biopsies during and after IL-2 treatment	Regrowth of body or scalp hair in all 5 patients, of scalp hair in 4.
**Induction of Regulatory T Cells by Low Dose IL-2 in Autoimmune and inflammatory Diseases** *Rosenzwajg, M. et al. Immunological and clinical effects of low-dose interleukin-2 across 11 autoimmune diseases in a single, open clinical trial. Ann Rheum Dis 2018, Nov 24. Doi: 10.1136/annrheumdis-2018-214229. [Epub ahead of print]. NCT01988506*	Moderate forms of **Autoimmune and inflammatory diseases	46	1	One 5-day course, followed by 1 injection every 2 weeks for 6 months	**18** (182)	Increase in CD4+ Tregs. No significant increase in Teffs.	Well tolerated in all diseases. Indication of potential clinical efficacy.
**Low-dose IL-2 therapy in one SLE patient refractory to standard therapies** *Humrich, J. Y. et al. Rapid induction of clinical remission by low-dose interleukin-2 in a patient with refractory SLE. Ann. Rheum. Dis. (2015). doi:10.1136/annrheumdis-2014-206506*	Severe SLE	1	1.5 then 3	Four 5-day courses	**52.6** (60)	Treg increase; decreased of anti-dsDNA antibody levels.	Well tolerated; Major clinical improvement

**Low-dose interleukine-2 in active systemic lupus erythematosus** *David Klatzmann & Abul K. Abbas Nature Reviews Immunology Volume: 15, Pages: 283–294 (2015) doi:10.1038/nri3823 (Personal communication with Di Yu and Zhanguo Li)*	SLE	40	1	3 courses of daily injections every other day for 2 weeks	**21** (90)	Treg increase (x2); decreased of anti-dsDNA antibody levels.	Well tolerated; Major clinical improvement in 36/40 patients who showed both clinical and serological remission
**Ultra-Low dose IL-2 for Refractory Chronic Graft Versus Host Disease** *Koreth, J. et al. Interleukin-2 and regulatory T cells in graft-versus-host disease. N Engl J Med 365, 2055–66 (2011). Matsuoka, K. et al. Low-Dose Interleukin-2 Therapy Restores Regulatory T Cell Homeostasis in Patients with Chronic Graft-Versus-Host Disease. Sci. Transl. Med. 5, 179ra43 (2013).*	cGVHD	23	0.3,1,3/m^2 (0.54 to 5.4)	Daily administration for 8 weeks, (4 weeks hiatus, follow-up administration for responders)	**32 to 320** (56 to 365)	CD4 Treg increase (x8); NK cell increase (x2)Asymptomatic peripheral-blood eosinophilia	Grade-3 and -4 AEs; 12 partial response; Tapering of corticosteroids by 60% in responders
**Ultra Low-Dose IL-2 for GVHD Prophylaxis** *Kennedy-Nasser, A. A. et al. Ultra low-dose IL-2 for GVHD prophylaxis after allogeneic hematopoietic stem cell transplantation mediates expansion of regulatory T cells without diminishing antiviral and antileukemic activity. Clin Cancer Res 20, 2215–25 (2014). NCT00539695*	Prevention of GVHD	16	0.1 and 0.2/m^2 (0.18 to 0.36)	3 times per week for 6-12 weeks	**3.3 to 13** (42 to 84)	Expansion of Tregs (x2); No expansion of CD8+ memory Teffs or NK cells	No grade-3 and -4 GVHD; Less infections than in control group
**Ultra-low Dose IL-2 in Healthy Volunteers** *Ito, S. et al. Ultra-low dose interleukin-2 promotes immune-modulating function of regulatory T cells and natural killer cells in healthy volunteers. Mol. Ther. J. Am. Soc. Gene Ther. 22, 1388–1395 (2014).*	Healthy volunteers	21	0.05,0.1,0.2/m^2 (0.09 to 0.36)	One 5-day course	**0.45 to 1.8** (5)	Expansion of Helios+ and Helios– Tregs; Dose dependent increase of CD56 bright NKs; Increase in serum IP10; No increase in IL2, IFNg, IL10, IL115, IL17	Well tolerated<br>All grade-1 AEs, except for 1 grade-2 injection site reaction
**Ultra-Low dose IL-2 for Refractory Chronic Graft Versus Host Disease** *Koreth, J et al. Efficacy, durability and response predictors of low-dose interleukin-2 therapy for chronic graft-versus-host disease. Blood 128, 130-137 (2016). NTC00529035*	cGVHD	33	1	Daily administration for 12 weeks (4 week hiatus, follow-up administration for responders)	**84**	Rapid expansion of Treg and NK cells with peak after 4 week, then plateau.	Low relapse and secondary malignancy rates.

Table 1: Examples of published findings from low dose IL-2 clinical trials
(MIU: million international units, **: Rheumatoid arthritis, Ankylosing spondylitis, SLE, Psoriasis, Behcet's Disease, Wegener's granulomatosis, Takayasu's disease, Crohn's Disease, Ulcerative colitis, Autoimmune hepatitis and Sclerosing cholangitis)

7 Rationale for Trial

The experimental and clinical background in low-dose IL-2 therapy suggests a potential clinical utility of Treg cell expansion in patients with ACS. Administration of low doses of IL-2 in various clinical settings appears to be safe and remarkably efficacious at promoting selective expansion of Treg cells with preserved suppressive function. Circulating Tregs are reduced at admission for ACS and during the first 8 weeks after the index event, but recover to normal levels thereafter[15]. Quick re-establishment and maintenance of 'normal' Treg levels during that phase is paramount. Indeed we speculate that increasing Treg levels to above normal may have beneficial effects on myocardial repair and decrease atherosclerotic progression.

Low dose interleukin-2 in patients with stable ischaemic heart disease and acute coronary syndromes (LILACS) was a, single centre (with an associated shared care site) study conducted in Cambridge. It was a Phase 1/2a double blind, placebo controlled clinical trial (MHRA reference 24551/0029/001-0001; REC reference: 17/NW/0012), where, for the first time, we assessed the safety and biological efficacy of low dose IL-2 therapy in patients with stable ischaemic heart disease and ACS (where the drug is currently contraindicated). For Part A, we dosed 25 patients with stable ischaemic heart disease in 5 escalating dose groups, with doses ranging from 0.3 to $3x10^6$ IU/daily for 5 consecutive days (Table 2). In Part B, we dosed 16 patients who presented with a non-ST elevation acute coronary syndrome (similar to this IVORY trial) in 2 dose escalating groups for 5 consecutive days.

**LILACS Trial Part (A or B)**	**Part A**					**Part B**	
**Group**	Group 1	Group 2	Group 3	Group 4	Group 5	Group 1	Group 2
**Dose**	0.3 $x10^6$IU	0.6 $x10^6$IU	1.2 $x10^6$IU	2.4 $x10^6$IU	3.0 $x10^6$IU	1.5 $x10^6$IU	2.5 $x10^6$IU
**Number of subjects dosed per group**	5	5	5	5	5	8	8
**Ratio of IMP to Placebo (IMP : Placebo)**	3:2	3:2	3:2	3:2	3:2	3:1	3:1

Table 2: LILACS dosing schedule

<u>Serious Adverse Events</u>

There were no serious adverse events (SAEs) in Part A of LILACS.

There were 2 SAEs in LILACS Part B which recruited patients with unstable angina and NSTEMI. The first one was after consent but prior to administration of IMP and therefore not related. The second event was a patient admitted with a non-ST elevation myocardial infarction (NSTEMI). A pre-dose coronary angiogram demonstrated severe three vessel coronary artery disease requiring urgent inpatient coronary artery bypass surgery. This patient was dosed (with either Aldesleukin 2.5 x 10^6 IU/Placebo) whilst awaiting inpatient transfer for cardiac surgery. The patient had transient episodes of chest pain prior to dosing and on the last day of dosing. In the case of the latter

episode of chest pain, no ECG changes were noted, however a rise in troponin suggested further myocardial ischaemia. This was deemed a significant medical event and hence an SAE. There was no clinical consequence of the event, no additional treatment was needed, and patient went on to their planned interventional surgery without complication. This SAE was classed as unrelated to the IMP due to the patient's severe underlying condition for which he was awaiting bypass surgery. Both SAEs have resolved.

AE description	Frequency	Severity	Relatedness
Abdominal pain	1	severe	unrelated
Raised troponin	1	severe	unrelated

Table 3: LILACS symptoms and signs documented as SAEs

Non-Serious Adverse Events

In 41 patients (205 injections in total) in Parts A (stable IHD) and B (ACS patients) of the LILACS trial, 93 injection site reactions in the form of localised skin reactions have been observed. Typically, a patient received 5 injections over the course of the trial and as such more than 1 ISR was observed in a single patient in some instances. The lesions have been limited to a diameter of 3-4 cm and have been self-limiting with full resolution without sequelae within a week. This is a known reaction to subcutaneous IL-2 and can also be attributable to dextrose 5% solution which is the diluent for the IMPs.

Other adverse reactions reported in the LILACS trial included flu like syndrome, fatigue, body ache and nasal congestion. These are possibly related to a systemic reaction to IL-2 and usually occur a few hours after injection of the IMP and can occur together. The symptoms usually resolve overnight and do not interfere with activities of daily living. There are no long lasting sequelae. Other AEs are infrequent. Appendix 1 lists the AEs and their causality as assessed by the blinded investigator.

The primary efficacy outcome for LILACS is to determine the ability of low dose IL-2 to increase circulating levels of Tregs by greater than >75%. When examining aggregated data, this was achieved at doses of between $1.5x10^6$ IU and $3.0x10^6$ IU daily in stable IHD and in ACS patients. In both groups, we did not see an increase in T effector cells[53].

An analysis of unlocked and blinded (stats unblinded) Treg and safety data for LILACS Part B was carried out. The safety data were deemed acceptable as there were no drug related SAEs as assessed by the investigator. In group B1 (1.5×10^6 IU), an 80% increase in Tregs from baseline was observed between visit 2 and visit 7. The dose 2.5 $\times 10^6$ IU in group B2 led to an increase in Tregs of 115% between visit 2 and visit 7. At the same time no significant increase in T effector cells was noted.

Based on these emerging data we are confident in the safety and biological efficacy of low dose IL-2 to significantly increase Tregs, and therefore wish to extend its use in ACS patients and further assess its clinical efficacy with repeated doses of 1.5×10^6 IU beyond the induction phase as tested in the LILACS trial.

The aim of the IVORY trial is to assess the efficacy of repeated low doses of IL-2 in reducing vascular inflammation, as assessed by ^{18}F-FDG PET/CT, in patients presenting with ACS.

The planned doses will be given to the trial patients as subcutaneous injections once a

day, over five consecutive days for an initial induction phase, and additionally the same dose once every 7 days for a further 7 doses during a maintenance phase – resulting in a total of 12 doses per patient (*1:1 randomisation of low dose IL-2 vs. placebo).* On the basis of safety and tolerability data from published clinical studies (Table 1), and from our own unpublished safety and tolerability data from the LILACS trial as described above, the dose to be used in this trial will be $1.5x10^6$ IU for induction and maintenance phases.

^{18}F-FDG PET/CT has been widely used as a biomarker for drug development in phase II studies[39,40]. ^{18}F-FDG PET/CT has been chosen as the imaging modality because in atherosclerosis, vascular ^{18}F-FDG uptake correlates with immune cell infiltration and glucose consumption[41]. It has been used by ourselves and collaborators worldwide to assess the effect of statins and anti-inflammatory compounds on vascular inflammation[42,43] and predicts subsequent cardiovascular events[44,45].

7.1 Hypotheses

Hypotheses: Treatment of patients with ACS using low-dose IL-2 will reduce vascular inflammation as assessed by ^{18}F-FDG-PET/CT.

A priori hypothesis: Patients presenting with high sensitivity C-reactive protein (hs-CRP) levels >2mg/l have residual systemic inflammation and are at high risk of recurrent CV events. We believe those patients are most likely to benefit from a treatment with low-dose IL-2, which is expected to reduce CV events through the suppression of inflammation. Thus, we will restrict recruitment in this trial to patients with ACS and hsCRP>2mg/l.

8 Trial Design

8.1 Statement of Design

This is a randomised, double-blind, placebo controlled, parallel group experimental medicine trial. The aim of the trial is to test the superiority of low dose IL-2 compared to placebo in reducing vascular inflammation in ACS patients with hs-CRP>2mg/l.

8.2 Number of Centres

There will be 2 hospital sites in Cambridge taking part. Addenbrooke's Hospital, Cambridge, will be the main trial site and Royal Papworth Hospital (which is now situated on the Cambridge Biomedical Campus) will be a shared care site where patients may be recruited and/or have study visits.

8.3 Number of Patients

A sufficient number of patients will be enrolled so that at least 60 patients with data suitable for the primary statistical analysis (approximately 30 per arm) complete the trial. To achieve this, we estimate that we will need to recruit approximately 90-100 patients to account for the percentage of ACS patients with hsCRP>2mg/l (approximately 65% of ACS patients) and potential dropouts/unanalysable data. However, we will continue to recruit patients until target patient completion is achieved.

8.4 Trial Duration

The total trial duration for each patient will be approximately 13 weeks. This will include 1 week for recruitment and screening, 8 weeks of treatment and a follow up period of approximately 4 weeks after the last treatment visit. The follow-up PET/CT will be scheduled 1 week after the last treatment visit.

8.5 Trial Objectives

8.5.1 Primary objective

- To compare the effect of low dose IL-2 against placebo on vascular inflammation using ^{18}F-FDG PET/CT in ACS patients.

8.5.2 Secondary objectives

- To determine if low dose IL-2 can increase Treg and alter Teff cell numbers over extended treatment
- To determine the safety and tolerability of extended dosing of low dose IL-2 in patients with an acute coronary syndrome

8.5.3 Exploratory objectives

- To determine the impact of low dose IL-2 on peripheral blood mononuclear cell subsets which may include (but not limited to) B cells and NK cells.

- To determine the effect of low dose IL-2 on left ventricular systolic function.
- To determine the effect of low-dose IL-2 on systemic inflammation measured by cardiovascular biomarkers (including but not limited to hsCRP, IL-6, Troponin I).
- To determine the effect of low-dose IL-2 on the gut microbiome
- To determine the effect of low-dose IL-2 on coronary artery inflammation
- To compare the effect of low-dose IL-2 against placebo on ^{18}F-FDG uptake in bone marrow in ACS patients

8.6 Trial endpoints

8.6.1 Primary endpoint:

Change in vascular inflammation (as measured by mean TBR max in the index vessel) on ^{18}F-FDG PET/CT from baseline to follow up scans.

(In detail, a region of interest (ROI) including arterial wall and lumen will be drawn on each axial slice of artery (ascending aorta and both carotid arteries) on the co-registered PET/CT scan and the maximum standardised uptake value (SUVmax) recorded. Subsequently, each ROI will be normalised by the blood FDG concentration in the superior vena cava or jugular vein (for carotids), to yield an arterial mean maximum tissue-to-blood ratio (TBR max) as a quantitative measure of arterial tracer uptake. The "index vessel" (defined as the arterial territory with the highest mean max TBR at baseline – left carotid, right carotid or ascending aorta) will be the primary outcome variable.

All scans will be analysed by an experienced reader, anonymised to patient identifiable information (name, treatment group, and visit number.)

8.6.2 Secondary endpoints:

1. Change in mean TBR_{max} in each arterial region individually restricted to those slices with TBR>1.6 (as per previous publications[46])
2. Change in lymphocyte subsets (T effector (Teffs) cells, defined as central memory and effector memory T cells in the non-Treg gated T cells will be evaluated by flow cytometry.
3. Change in percentage of Treg cells (defined as $CD3^{+}CD4^{+}CD25^{high}CD127^{low}$ cells within the $CD3^{+}CD4^{+}$ T cell gate) between low dose IL-2 and placebo throughout the treatment period will be evaluated by flow cytometry.
4. The safety and tolerability of extended dosing of IL-2 in ACS patients will be evaluated by:
    - Adverse events
    - Further cardiovascular events
    - Concomitant medications
    - Physical examination defined as examination of the cardiovascular, respiratory, gastrointestinal, limited skin and brief neurological examinations
    - Examination of injection site reactions
    - Vital observations which include blood pressure, heart rate, temperature, respiratory rate and oxygen saturation
    - Safety clinical blood tests (defined in section 11.6)
    - Thyroid function blood test (defined in section 11.6)
    - 12-lead electrocardiogram (ECGs) recordings

8.6.3 Exploratory endpoints:

1. Change in serum cardiac biomarkers.

2. Change in ejection fraction as measured on transthoracic echocardiograms

3. Change in phenotype and function of peripheral blood mononuclear cell (PBMC) subsets (such as B lymphocytes and Natural Killer cells) as assessed by flow cytometry, gene expression, and in vitro activation and suppression assays.

4. Differences in gut microbiota composition between low-dose IL-2 vs placebo will be identified using 16S- RNAseq

5. The effect of low-dose IL-2 on coronary artery inflammation will be measured by perivascular fat attenuation using computed tomography coronary angiography

6. The effect of low-dose IL-2 on ^{18}F-FDG uptake in cervical/thoracic vertebrae.

9 Selection and withdrawal of patients

9.1 Inclusion Criteria

To be included in the trial the patient must meet the following criteria:

- Able to provide written informed consent to participate
- Aged between 18 and 85

- Current admission (on the screening visit) with an acute coronary syndrome - ST elevation myocardial infarction (STEMI), non-ST elevation myocardial infarction (NSTEMI), or unstable angina (UA) with symptoms suggestive of myocardial ischaemia lasting 10 minutes or longer with the patient at rest or with minimal effort

  AND EITHER

  i. elevated levels of TnI on admission

  OR

  ii. dynamic changes in ECG (new ST-T changes or T-wave inversion).

- Where applicable, to be included in the trial women must be

i) Postmenopausal (for the purposes of this trial, postmenopausal is defined as being amenorrhoeic for greater than 2 years with an appropriate clinical profile, e.g. age appropriate, history of vasomotor symptoms)

1. OR

ii) Have had a documented hysterectomy and/or bilateral oophorectomy or sterilised

1. OR

iii) Peri-menopausal with a negative pregnancy test at screening (for the purposes of inclusion in this trial. Peri-menopausal is defined as women with an appropriate clinical profile, e.g. age appropriate, history of vasomotor symptoms, irregular periods). They will also have to comply with the use of contraception for the duration of the trial and undergo additional pregnancy tests during and after treatment.

- High sensitivity C-reactive protein of >2 mg/L, at any point from index admission for acute event to screening (inclusive)
- Willingness and possibility to start dosing within 14 days from initial date of admission to the primary hospital for ACS
- Able to comply with all trial mandated visits.

9.2 Exclusion Criteria

The presence of any of the following will preclude patient inclusion:

- Current presentation (at screening) with cardiogenic shock (systolic blood pressure <80 mm Hg, unresponsive to fluids, or necessitating catecholamines).
- Current presentation with cardiac arrest
- Signs or symptoms of active infection requiring intravenous antibiotic treatment at screening
- History of malignancies requiring active treatment (However, patients with a history of treated localised basal or squamous cell skin cancer are not excluded from participation in this trial)
- History of solid organ transplantation or other bone marrow transplantation
- History of recurrent epileptic seizures in the previous 4 years; repetitive or difficult to control seizures, coma or toxic psychosis lasting >48 hours
- Uncontrolled hypotension (Systolic BP (SBP)<80mmHg or DBP<50mmHg) OR uncontrolled hypertension (SBP>180 or DBP>120 mmHg) at screening

- Average corrected QT interval (QTc) > 450 msecs using Bazett's formula from average of triplicate ECGs (or > 480 msecs if bundle branch block)
- Renal impairment defined as Creatinine clearance [Cockcroft-Gault] <45ml/min at screening
- Liver dysfunction (defined as ALT > 2xULN) at screening
- Evidence of cholestasis defined as elevated Total Bilirubin Levels, (TBL > 1.5 x ULN) and Alkaline Phosphatase, ALP (ALP > 1.5 x ULN), at screening
- Known hypothyroidism or hyperthyroidism
- Known autoimmune disease requiring active immunosuppressive treatment
- Any oral or intravenous immunosuppressive treatment including regular prednisolone, hydrocortisone or disease modifying drugs. [Inhaled or topical steroids are permissible]
- Patients on cytotoxic drugs and interferon-alpha
- Diabetics on oral hypoglycaemics/diet control with HbA1c (DCCT) > 8% (OR HbA1c (IFCC) > 64 mmol/mol), at screening. Diabetics on insulin are excluded from the study.
- Contraindication to IL-2 treatment or hypersensitivity to IL-2 or to any of its excipients
- Participation in a previous research trial in the last 3 years which involved exposure to significant ionising radiation (i.e. cumulative research radiation dose >5 mSv)
- Participation in a clinical trial where the patient has received a drug or new chemical entity within 30 days or 5 half-lives, or twice the duration of the biological effect of the drug (whichever is longer) prior to the first dose of trial medication, Visit 3 (Day 1).
- Any medical history or clinically relevant abnormality that is deemed by the principal investigator/delegate to make the patient ineligible for inclusion because of a safety concern
- Pregnant women or breast feeding women
- Patients who are COVID-19 PCR positive at the time of screening
- Known severe allergy to the CT-contrast agents.

9.3 Treatment Assignment and Randomisation Number

A sufficient number of patients will be randomised so that approximately 60 patients will complete the trial. Stratification at randomisation based on an ECG based ST-elevation status (to define non-STEMI or STEMI status) will be utilised to balance the groups. Patients will be randomised in a 1:1 fashion to either low dose interleukin-2 or placebo using an online randomisation system (Sealed Envelope).

9.4 Method of Blinding

The trial will be double-blind, with active and placebo doses appearing identical at point of issue and administration. The CUH central pharmacy will be unblinded and provided with a copy of the concealment list. Data analysis for the trial will be performed by a statistician who will be unblinded after the database lock.
The statistician, or delegate, may be unblinded for individual patients after their treatment period has concluded, to facilitate rapid reporting of safety events to the IDMC.

9.5 Patient Withdrawal Criteria

Withdrawn patients may be replaced by recruiting and randomising new patients. Reasons for patient withdrawal will be recorded in the Case Report form (CRF). Reasons for withdrawal may include: adverse event, SAE, SUSAR, withdrawal of consent, lost to follow-up, protocol deviation, patient non-compliance or the trial closing/terminating.

Specific organ based withdrawal criteria are set out below:

9.5.1 Liver withdrawal criteria

Liver chemistry threshold stopping criteria have been designed to assure subject safety and to evaluate liver event aetiology.

Patients will be withdrawn if any of the following liver chemistry stopping criteria are met:

1. ALT ≥ 3xULN and total bilirubin ≥ 2xULN
2. ALT ≥ 5xULN.
3. ALT ≥ 3xULN if associated with symptoms (new or worsening) believed to be related to hepatitis (such as fatigue, nausea, vomiting, right upper quadrant pain or tenderness or jaundice) or hypersensitivity (such as fever, rash or eosinophilia).
4. Isolated ALT ≥ 3xULN persists for ≥ 4 weeks.

9.5.2 Renal withdrawal criteria

Serum creatinine elevation > 2 x baseline screening visit (V1)

9.5.3 Cardiac withdrawal criteria

These assessments will be carried out during patient screening and patients that meet the criteria below will be withdrawn from the trial. The Bazett's QT correction formula should be used to determine inclusion and discontinuation for any individual patient throughout the trial.

- QTcB >500msec OR
- Change from baseline: QTcB >60msec

If a patient has underlying bundle branch block the following withdrawal criteria should be used instead.

Baseline QTcB value (with underlying bundle branch block)	QTcB withdrawal criteria
<450msec	>500msec
450-480msec	≥530msec

Withdrawal of patients is to be based on an average QTcB value of triplicate ECGs. If an ECG demonstrates a prolonged QT interval, obtain 2 more ECGs over a brief period (approximately 5 minutes) and then use the averaged QTcB values of the 3 ECGs to determine whether the patient should be discontinued from the trial.

Patients who require CABG (either inpatient or outpatient) during the scheduled dosing period should be withdrawn from the study.

Patients who develop new onset severe pulmonary oedema/new severe congestive heart failure, requiring high dose > 240 mg over 24 hours IV furosemide during admission will also be withdrawn from the trial.

Patients with **symptomatic** hypotension systolic BP < 80 mmHg and/or diastolic BP < 50 mmHg (after at least 2 repeat recordings), or severe uncontrolled hypertension (as defined by BP > 180 mmHg systolic BP or >120 mmHg diastolic BP on at least two readings) will also be withdrawn.

Atrial fibrillation is not a reason for withdrawal in this population.
Patients with sustained (>30 sec or symptomatic) ventricular tachycardia and patients with ventricular fibrillation will be withdrawn. Non-sustained ventricular tachycardia is a common occurrence in this population, and is not a reason for withdrawal in this population.

9.5.4 General withdrawal criteria

- Cardiorespiratory arrest
- Failure to attend 2 scheduled dosing appointments without adequate reason based on the PI assessment
- Severe hypersensitivity reactions will preclude any further IMP administration
- New seizure activity
- Coma
- Severe lethargy or somnolence
- Respiratory insufficiency requiring intubation
- Pregnancy
- Patients who become COVID-19 PCR positive during the dosing phase of the trial. [Note: Patients who have completed their full initial vaccination course and are asymptomatic may continue in the trial upon completion of any government mandated period of self-isolation]
- Withdrawal of consent
- PI discretion
- Any serious adverse reaction (SAR) or adverse reaction which is deemed by investigators as Severe (AR).
- Any significant incidental finding on PET/CT scan clinical governance reports, which in the opinion of the PI, necessitates further investigation and management.
- Any medical history, clinically relevant abnormality or reason that is deemed by the principal investigator (PI) to make the patient ineligible to continue the trial.

9.5.5 Management of withdrawal

- Withdrawn patients may be replaced with newly recruited patients to maintain the power of the study

- Withdrawn patients will be followed up as per the PI discretion depending on the clinical scenario
- Withdrawn patients will not be subjected to additional research related tests (e.g. blood tests for research purposes, PET/CT scans) from the date of withdrawal but may have safety related tests (e.g. blood tests, ECGs) at the PI's discretion on clinical grounds
- All blood samples or imaging data collected prior to the date of withdrawal may be analysed.

9.6 Trial stopping criteria

For the first 10 patients dosed, if a cumulative total of three events are observed, the trial conduct will be halted (recruitment and dosing of all active patients). These events include serious adverse reactions (SARs) defined as possibly, probably or definitely related to the trial drug or severe adverse reactions (ARs). This will trigger an unscheduled independent DMC meeting to convene to review the trial data to date. Once the trial has halted for the aforementioned reasons, it may only resume after approval of a substantial amendment to the Regulatory Authorities that have approved the trial.

After the tenth (10th) patient has commenced dosing, a percentage basis (30%) of patients who experience pre-specified events (SARs defined as possibly, probably or definitely related to the trial drug or severe adverse reactions) will be used to halt the trial and review the data by the IDMC. Once the trial has halted for the aforementioned reasons, it may only resume after approval of a substantial amendment to the Regulatory Authorities that have approved the trial.

10 Trial Treatments

10.1 Dosage schedules

10.1.1 Route of Administration and Maximum dosage allowed

Patients will be treated with daily subcutaneous injections of IMP in two different phases: induction and maintenance. The dose of IL-2 will be fixed and the same for all patients during all injections and has been defined as $1.5x10^6$ IU per injection (or equivalent volume of placebo) based on data available from the LILACS trial.

During the induction phase, patients will be treated with IMP, given subcutaneously, once daily for five days (+/- 1 day is allowed V4-V7 for each visit). This will occur during visits V3-7. The induction phase can last approximately 5 days.

The maintenance phase will start 1 week after the induction phase. The first dose during this phase occurs 7 (+/-3) days after the last dose of the induction phase. The dose of IMP will be given subcutaneously once weekly (every 7th (+/-3) day) for 7 doses in total. This will occur during visits V8-14.

Therefore, in total, 12 IMP injections will be given across the induction and maintenance phases for each completed patient.

10.1.2 Maximum duration of treatment of a patient

The maximum treatment duration is 8 weeks.

10.2 Presentation of the drug

Active drug description
Commercially available aldesleukin with a UK marketing authorisation will be used and will be initially prepared as per SmPC, with further dilution as described below.

For this trial, the method described for aldesleukin dilution in an SmPC for Ceplene will be utilised, producing a final aldesleukin concentration of 3.3 x 10^6IU/ml (200µg/ml). Existing (unlicensed) data from several sources supports stability and sterility of reconstituted diluted IL-2 preparations (prepared as per SmPC and then further diluted with dextrose 5%) for up to 21 days at 2-8ºC when syringes are prepared by qualified health-care professionals under aseptic conditions.

Placebo description
Commercially available dextrose 5% injection with a UK marketing authorisation at equivalent dose volume will be used for the placebo formulation.

10.3 Known drug reactions & interaction with other therapies

Significant clinical interactions have been reported when high dose IL-2 (Aldesleukin) has been combined with cytotoxic drugs and interferon-alpha; therefore, patients on these drugs will not be permitted to participate in the trial, nor are these medications permitted during this trial.

10.4 Dosage modifications

This is a fixed dose trial and no dose modifications will be permitted. The dose selected for this trial is 1.5x10^6 IU per injection (or equivalent volume of placebo) and will be used for all patients for all visits.

10.5 Legal status of the drug

Aldesleukin is a commercially available Prescription Only Medicine and is licensed for the treatment of metastatic renal cell carcinoma in the UK.

Within this trial, aldesleukin (interleukin-2, IL-2) and matching placebo are classed as Investigational Medicinal Products.

10.6 Drug storage and supply

Commercial supply of IL-2, from hospital pharmacy stocks, will be used for this trial. Vials will be stored as per SmPC in a secure location with access restricted to appropriate individuals.

Commercial supply of dextrose 5% injection, from hospital pharmacy stocks, will be used for placebo, stored as per labelled instructions.

IMPs will be dispensed by the site pharmacy for this trial upon receipt of a suitably signed trial specific prescription.

10.7 Accountability

Records will be maintained by pharmacy to document safe receipt, handling, storage, dispensing and return of unused investigational medicinal products (IMPs) as appropriate.

10.8 Concomitant Therapy

The IMP administration based on data from previous IL-2 studies may result in fever, headache, migraine or diarrhoea, mostly in patients treated with doses higher than the doses to be used in this trial. Concomitant therapy with Paracetamol (up to 1g every 6 hours for 24 hours after IL-2 administration) can be instituted at the time of IL-2 administration to reduce fever. Pethidine may be added to control the rigors associated with fever. Anti-emetics (the use of 5-HT3 receptor antagonists, e.g. Ondansetron, and corticosteroids is not advised) and antidiarrheals may be used as needed to treat other gastrointestinal adverse reactions. Local reactions at injection site (erythema, pain, pruritus) are the most common non-serious adverse events. Some patients with pruritic rash benefit from concomitant administration of antihistamines.

If a patient is admitted to hospital for any of the planned/elective investigations listed in section 12.3.2, concomitant medications pertaining to that hospital admission will not need to be recorded. All regular medications for home use as part of standard of care will be documented.

10.9 Emergency unblinding

It is the responsibility of the Chief/Principal investigator or sub-investigator to unblind the patient in the case of an adverse event, which in their judgement, requires knowledge of the trial medication received by the subject in order to provide appropriate treatment or management of the adverse event. The investigator or sub-investigator is also responsible for promptly documenting and explaining the unblinding to the Sponsor. Emergency unblinding will be undertaken by an authorised study team member using the online randomisation/unblinding system. As a back-up in the event of failure of the online system, arrangements within the trial pharmacy will be made for 24 hour access to emergency unblinding by providing the Kit Number that the patient was assigned at the point of patient randomisation.

11 Procedures and assessments

Trial assessments and procedures will be performed by suitably qualified and delegated trial personnel as described in the TPM. Informed consent must be obtained from the patient prior to any trial-specific procedures taking place.

11.1 Patient identification

ACS patients will be identified on medical wards at Addenbrooke's and Royal Papworth Hospitals by their treating clinical care team or the trial research team clinical staff. This may be achieved by reviewing inpatient medical notes or by discussion with clinical teams regarding their inpatients.
Permission to approach the patient will be sought by the trial team from the treating clinical care team. The patient will be approached by the usual clinical care team of the patient to determine if they may be interested in hearing about research. If the patient agrees, the trial team will approach the patient. (Rarely some members on the trial team may be on call and therefore be part of the clinical care team - in which case permission to approach the patient will be sought from the clinical consultant in charge of the care of the patient prior to the patient being approached).

Once contact has been made with the patient, the trial team will outline and explain the aims of the trial. A copy of the Patient Information Sheet together with a copy of the Mini Patient Information Sheet will be provided to interested patients, who will then be given the opportunity to consider the information with relatives and then to discuss the trial with trial staff and have any queries answered before consenting to participate in the trial.

11.2 Consent

The Informed Consent form must be approved by the REC and must be in compliance with GCP, local regulatory requirements and legal requirements. The investigator or designee must ensure that each trial patient is fully informed about the nature and objectives of the trial and possible risks associated with their participation.

The investigator or designee will obtain written informed consent from each patient before any trial-specific activity is performed. The informed consent form used for this trial and any change made during the course of this trial, must be prospectively approved by the REC. The investigator will retain the original of each patient signed informed consent form.

Should a patient require a verbal translation of the trial documentation by a locally approved interpreter/translator, it is the responsibility of the individual investigator to use locally approved translators.

Any new information which becomes available, which might affect the patient's willingness to continue participating in the trial will be communicated to the patient as soon as possible. This new information may be conveyed to the patient either over the phone, in writing, or in person at their next trial visit at the discretion of the PI depending on the nature of the new information.

11.3 Screening evaluation

11.3.1 Screening Assessments (**Visit 1**) Day -14 to 0

Trial specific assessments will only be conducted after patients have given written informed consent.

The screening visit can take place at suitably equipped wards or clinical research units at either Addenbrooke's or Royal Papworth Hospitals. The following procedures will be performed at this visit:

- Demography (date of birth, age, gender and race)
- Medical history
- Current medication history
- Physical examination (including cardiovascular, respiratory, gastrointestinal, neurological and skin examinations)
- Height and weight
- Vital observations (defined henceforth as temperature, blood pressure, heart rate, respiratory rate and oxygen saturations)
- 12-lead electrocardiogram (ECG) in triplicate with an average QTcB taken
- Transthoracic echocardiogram (TTE) – This can be done at any point between screening and V3, prior to dosing. If a research ECHO cannot be performed, a clinical ECHO undertaken during the index admission can be used.
- Screening bloods (approximately 15mls) including:
    - Safety blood tests (defined as but not limited to: haematology (full blood count and differential), clinical biochemistry (electrolytes, urea, creatinine, liver function (ALT, ALP, albumin, bilirubin))
    - Thyroid function tests (TSH - V1 and V16 only [fT4 will only be performed if TSH is abnormal])
    - hsCRP
    - Serum and/or pregnancy test where applicable
    - HbA1c in known T2DM patients who are diet controlled or on oral hypoglycaemics

11.3.2 <u>Patient Randomisation</u>

All screening tests must be returned and checked by the trial doctor before randomisation. The PI or a delegate must sign the CRF to confirm eligibility after the screening process has been completed.

Randomisation will occur centrally at the main trial site, Addenbrooke's Hospital, and Addenbrooke's Hospital central pharmacy will prepare the IMP for all patients.

11.4 Trial assessments

All trial visits will be held at suitably equipped wards or clinical research units at the trial sites.

Due to the ongoing COVID-19 pandemic and tiered/national lockdowns, patients may also be expected to have COVID-19 swabs as directed by local clinical guidelines/ policies. These are in addition to the research protocol as per current clinical requirements which are subject to change over time.

11.4.1 ^{18}F-FDG PET/CT Scan 1 (**Visit 2**) Day -13 to 0

The PET/CT scan (S1) must be performed after all other screening tests have been undertaken and after eligibility has been confirmed and signed by the PI or delegate. The scan must occur before the first dosing. Dosing can occur before the PET/CT is reported. Any significant findings at this visit necessitating withdrawal from the trial will be termed an AE unrelated to drug as this scan precedes drug administration. ^{18}F-FDG PET/CT imaging is expected to last for approximately 2 hours with a 90-minute break after radioligand injection.

A finger prick blood sugar test will be performed prior to the PET/CT scan and a cannula inserted. At the discretion of the PI further tests that need repeating from V1 can be performed.

If time permits the CT carotid angiogram can be done in this visit.

A review of eligibility, withdrawal criteria, adverse events and concomitant medications will also be done at this visit.

Incidental findings on PET/CT scans

PET/CT scans are commonly used in metabolic and oncological scenarios for the diagnosis and follow-up of known conditions. In this clinical trial, the PET/CT scan is not being performed for diagnostic reasons in ACS patients and therefore clinical review of scan results is not usually clinically required prior to initiation of any treatment in this patient population. Thus, clinical governance reporting of scan results will not be required before initiation of low-dose IL-2 or placebo in the present trial. The management of any incidental findings will not be affected by low-dose IL-2, which, in itself, is currently licensed at higher doses for the treatment of certain types of cancer. Following a clinical review of any incidental findings by relevant multidisciplinary teams, if the patient meets any of the exclusion or stopping criteria, or at the discretion of the PI, the patient may have their dosing stopped and therefore will be withdrawn from the trial for further investigation or management of an incidental finding should this be required. Often, incidental findings may just require routine imaging follow-up (e.g. lung nodules) therefore this will be conducted clinically but the patient will not be withdrawn from the trial or from dosing for this reason.

11.4.2 Induction phase – **(Visit 3-7)** Day 1-5

Visit 3 must occur within 14 days of the first day of the patient's admission to the hospital with an ACS. At Royal Papworth Hospital this is defined as admission to their primary hospital and not the date of transfer to Papworth. Subsequent visits should occur on the scheduled day (+/- 1 day). All trial visits will be held at suitably equipped wards or clinical research units at the trial site(s).

Each visit will last approximately 1 hour and the following procedures will occur:

- (V3 only) – rechecking of inclusion and exclusion
- Checking withdrawal criteria (all visits after V3)
- Adverse event and concomitant medications reporting daily
- Baseline vital observations
- Physical examination
- Baseline 12-lead ECG with QTcB measurement will be performed pre-dosing
- V3 only

        - Review of safety bloods prior to dosing by the PI or delegate. Due to the COVID pandemic, efforts to reduce patient waiting times may be employed. Dosing decisions may be made on point of care bloods (if available, and as a minimum, review of Hb, sodium, potassium, creatinine should be sufficient) ahead of review of formal laboratory safety bloods assuming there is no major clinical deviation of parameters from recent blood tests. If POC is not available, formal lab bloods will be reviewed prior to dosing.
        - If feasible, a pre-dose stool sample will be collected.
- V4 – V7 only: Review of safety bloods from the previous visit where safety bloods were done.
- Pre-dose bloods for V3 and V7 (approximately 55mls):
    - Clinical safety bloods
    - Treg and Lymphocyte subset analysis
    - Peripheral Blood Mononuclear Cell (PBMC) assays
    - Full lipid profile (V3 only)
    - Cardiac biomarkers (hsCRP, IL-6, , and Troponin I)

- Pre-dose bloods for V5 (approximately 15mls):
    - Clinical safety bloods

- Trial patients will have an injection of IMP administered subcutaneously on the abdominal area. The site of injection on the abdominal area should be varied daily to minimise discomfort.
- Further 12-lead ECG with QTcB measurement will be performed approximately 30 minutes post-dosing (+/-5 minutes) (2 further 12 lead ECGs will be performed if the first QTcB meets stopping criteria and an average will be taken)
- Repeat vital observations will be performed approximately 30 minutes (+/- 5 minutes) post-dosing
- After approximately 30 minutes post-dosing, if there are no safety concerns, patients can be discharged back to usual clinical care or discharged home (where appropriate)
- The patients' stay may be extended and further investigations performed at the discretion of the investigator if the clinical scenario dictates this, for example the development of an adverse event (for example: hypotension, chest pain or arrhythmias).

11.4.3 Maintenance Phase – (**Visits 8 -14)** Day 12-54

Visits 8-14 should each occur 7 days (+/- 3 days) after the intended previous visit. Scheduling of further visits is based on the intended visit date rather than actual visit date. All trial visits will be held at suitably equipped wards or clinical research units at the trial site. Review of safety bloods from the current visit will be done prior to dosing by the PI or delegate. Due to the COVID pandemic, efforts to reduce patient waiting times may be employed. Dosing decisions may be made on point of care bloods (if available, and as a minimum, review of Hb, sodium, potassium, creatinine should be sufficient) ahead of review of formal laboratory safety bloods assuming there is no

major clinical deviation of parameters from recent blood tests. If POC is not available, formal lab bloods will be reviewed prior to dosing.

Each visit will last approximately 1 hour and the following procedures will occur:

- Adverse event and concomitant medications reporting
- Baseline vital observations
- Brief physical examination
- Baseline 12-lead ECG with QTcB measurement will be performed pre-dosing
- Pre-dose blood tests for all visits (approximately 20mls in total):
    - Clinical safety bloods
- Trial patients will have an injection of IMP administered subcutaneously on the abdominal area. The site of injection on the abdominal area should be varied at each visit.
- Single 12-lead ECG with QTcB measurement will be performed approximately 30 minutes post-dosing (+/-5 minutes) (2 further 12 lead ECGs will be performed if the first QTcB meets stopping criteria and an average will be taken)
- Repeat vital observation will be performed approximately 30 minutes (+/- 5 minutes) post-dosing
- After approximately 30 minutes post-dosing, if there are no safety concerns, patients can be discharged back to usual clinical care or discharged home (where appropriate)
- The patient’s stay may be extended and further investigations performed at the discretion of the investigator if the clinical scenario dictates this, for example the development of an adverse event (for example: hypotension, chest pain or arrhythmias)

For **Visits 8, 10, 12 and 14** additional pre-dose bloods (in addition to those listed above) will be taken (total volume 55mls):

- PBMC assays
- Treg and Lymphocyte subset analysis
- Cardiac biomarkers (hsCRP, IL-6, and Troponin I)
- Serum or urinary pregnancy test where applicable (V10)

Post-dose transthoracic echocardiogram (TTE) may be undertaken at V14 or V15 or at an *ad hoc* visit.

11.4.4 Post-dose PET/CT – (**Visit 15**) Day 61

Visit 15 will occur 7 (+/-6) days after the intended final dosing visit V14.

A follow-up post-dose FDG-PET/CT will be performed during this visit. A finger prick blood sugar test will be performed prior to the PET/CT scan.

Following the post-dose PET/CT scan, the CT carotid angiogram (if not already done in visit 2) and the CT coronary angiogram (CTCA) will be performed during this visit or as separate visits.

A cannula will be inserted and the bloods for the following will be taken:

- Clinical safety bloods
- PBMC assays
- Treg and Lymphocyte subset analysis
- Cardiac biomarkers (hsCRP, IL-6, and Troponin I)
- Full lipid profile
- Thyroid function test (TSH)
- If possible, a stool sample will also be collected at this visit.

The scan is expected to last for approximately 2 hours which includes a 90-minute break after radioligand injection.

The following will also occur at this visit:

- Adverse events reporting
- Concomitant medications reporting
- Vital observations
- Physical examination
- 12-lead ECG with QTcB measurement

11.4.5 Follow-up – (**Visit 16**) Day 82

This visit will be conducted remotely (via telephone) unless there is a specific indication (for example safety blood tests) or to perform a serum / urinary pregnancy test for peri-menopausal women, in which case an in-person visit may be undertaken It will take place 28 days (+/- 3 days) after the last intended dosing visit.

The visit will last approximately 1 hour and the following will occur:

- Adverse events reporting
- Concomitant medications reporting
- Serum or urinary pregnancy test where applicable

The trial will routinely end for individual patients at V16. At the discretion of the PI, further visits or telephone contacts can be arranged to follow up unresolved issues/adverse events until the point of resolution.

11.5 Schedule of Assessments

**Visit no ->**	**1**	**2**	**3**	**4**	**5**	**6**	**7**	**8**	**9**	**10**	**11**	**12**	**13**	**14**	**15**	**16 (Remote where possible)**
**Treatment Day**	**-14 to 0**	**-13 to 0**	**1 ^**	**2 ^**	**3 ^**	**4 ^**	**5 ^**	**12[a]**	**19[a]**	**26[a]**	**33[a]**	**40[a]**	**47[a]**	**54[a]**	**61#**	**82[a]**
Informed consent	x															
18F-FDG PET/CT Scan[b]		x													X#	
Demography[c]	x															
Height/weight	x															
Medical history	x															
Medication history	x															
Inclusion/exclusion	x	x	x													
Adverse events	x	x	x	x	x	x	x	x	x	x	x	x	x	x	x	x
Concomitant medications	x	x	x	x	x	x	x	x	x	x	x	x	x	x	x	x
Serum / urinary pregnancy test	x									x						X*
HbA1c	X															
Physical examination[d]	x		x	x	x	x	x	x	x	x	x	x	x	x	x	X*
Vital observations[e]	x		x	x	x	x	x	x	x	x	x	x	x	x	x	X*
ECG	x[f]		x	x	x	x	x	x	x	x	x	x	x	x	x	X*
ECHO	x[g]													x[h]	x[h]	
IMP administration			x	x	x	x	x	x	x	x	x	x	x	x		
Safety bloods[i]	x		x	X +	x	X +	x	x	x	x	x	x	x	x	x	X*
OPTIONAL Point of care bloods			x					x	x	x	x	x	x	x		
TSH	x														x	X*
Tregs and Lymphocytes			x				x	x		x		x		x	x	X*
PBMC assay			x				x	x		x		x		x	x	X*
Cardiac biomarkers[j]			x				x	x		x		x		x	x	X*

hsCRP	x															
Full lipid profile			X												x	
Stool sample (where feasible/possible)			X												X	
CTA															X[k]	
CTCA															X[l]	

^ These visits can be scheduled +/- 1 day.
[a] These visits can be scheduled +/- 3 days.
[b] PET/CT assessment to be preceded by a fingerprick blood glucose test.
[c] Including DOB, age, gender, race.
[d] Including cardiovascular, respiratory, gastrointestinal, neurological and skin examinations.
[e] Temperature, blood pressure, heart rate, respiratory rate and oxygen saturations.
[f] 12-lead ECG in triplicate with average QTcB.
[g] May be done anytime after screening prior to dosing (this can occur at an ad hoc visit). If a research ECHO cannot be performed, a clinical ECHO undertaken during the index admission can be used.
[h] May be done anytime between V14 and V15.
[i] Safety bloods (including but not limited to): haematology (FBC and differentials), clinical biochemistry (electrolytes, urea, creatinine), liver function (ALT, ALP, albumin, bilirubin). POC bloods may be done to aid dosing administration prior to review of formal bloods - as a minimum, review of Hb, sodium, potassium, creatinine should be sufficient.
[j] hsCRP, IL-6, Troponin I.
[k] May be done as separate visits. The CT carotid angiogram can be done in visit 2.
[l] Can be done as a separate visit.
* Assessments to be done physically at the hospital only if it is clinically indicated.
[#] PET/CT can be scheduled +/- 6 days.
[+] Safety bloods will only be done if deemed clinically necessary.

11.6 Trial specific procedures

Specific details of all procedures will be provided in the trial procedures manual (TPM).

Blood Pressure

- Blood pressure will be measured pre- and post-dosing using a validated automated device.

Brief physical examination

- Brief physical examination includes physical examination of the cardiovascular, gastrointestinal and neurological systems as well as of the lungs and skin.

ECG with QTcB measurement

- 12-lead ECGs will be obtained at each time point during the trial using an ECG machine that automatically calculates the heart rate and measures PR, QRS, QT, and QTcB intervals. All ECGs are manually over read to check QTcB parameters by the investigator or suitably qualified and delegated member of the trial team to ensure the correct calculation of QTcB. All ECGs will be checked and signed and in the circumstance of an over read, this will be clearly documented on the subject's affected ECG.
- ECGs will be obtained in the semi-supine position after the subject has been resting for at least 5 minutes.
- For time points where ECGs are collected in triplicate these will be carried out over a brief period of approximately 5 minutes.

Imaging

- A General Electric Discovery 690 combined positron emission tomography (PET)/computed tomography (CT) scanner (Milwaukee, Wisconsin), or equivalent scanner, will be used for PET/CT imaging. Vascular imaging will be performed using reproducible, validated methods for image acquisition, reconstruction and interpretation as recommended by the European Association of Nuclear Medicine for the conduct of clinical trials using ^{18}F-fluorodeoxyglucose (FDG) PET[47].

- Patients should be fasted for approximately 6 hours prior to ^{18}F-FDG PET imaging and capillary blood glucose concentration should be confirmed as <7.0 mmol/l prior to scanning. If the blood glucose concentration is >11.0 mmol/l, this level should first be repeated, and if confirmed the scan should be re-scheduled as per departmental protocol at the discretion of the supervising trial doctor. For blood glucose values between 7-11 mmol/l, the scan may proceed, be delayed or re-scheduled at the discretion of the Chief Investigator. This may occur outside the trial window as defined in 11.4.1, and is at the discretion of the Chief Investigator.

- A dose of ~ 240Mbq ^{18}F-FDG will be injected through a peripheral venous cannula, followed by ~ 10 mL flush of normal saline, with the patient seated in an armchair, where they will rest in a quiet environment for a period of time (up to 90 minutes). Patients will be requested to avoid excessive speaking or swallowing/chewing to minimise physiological ^{18}F-FDG tracer uptake in the head and neck muscles.

- The ascending aorta will be imaged ~ 90 minutes after the ^{18}F-FDG injection. Attenuation correction and non-contrast CT scans of the ascending aorta will initially be performed. This will be followed by a single bed PET scan will be acquired with the superior portion of the aortic arch as the upper anatomical landmark of the scan.

- Carotid artery imaging will be undertaken immediately after the PET/CT scans of the ascending aorta. With the patients head and neck placed comfortably in a head holder and arms secured at their sides, attenuation correction and non-contrast CT scans of the neck will be performed. This will be followed by a single bed PET scan acquired with the external auditory meatus as the upper anatomical landmark of the scan.

- PET data for the ascending aorta and carotids will be reconstructed using iterative 3D time of flight ordered-subset expectation maximization with standard corrections applied ± point-spread function modelling to reduce partial volume error.

- Arterial radioactivity concentration (standardized uptake value (SUV)) will be quantified from maximum voxel value within regions of interest drawn on PET-CT images, normalised by blood pool activity and averaged with neighbouring slices to minimise the impact of noise, to derive tissue-to-blood ratios (TBR).

- CT carotid angiography will be performed after PET scanning, with contrast-enhanced CT images acquired from the aortic arch to the circle of Willis, using bolus tracking (the scan is bolus triggered on the aortic arch at 100HU) with an ROI placed in the aortic arch. This scan can be done in visit 2 or visit 15.

- CT coronary angiograms will be performed after the PET scanning or as a separate visit, with contrast enhanced CT images acquired following a standard clinical protocol with either prospective or retrospective gating. This will be done in V15 or as a separate visit.

Blood tests
The following routine measurements will be performed in blood samples:

- Safety clinical blood tests defined as (but not limited to): haematology (full blood count and differential), clinical biochemistry (electrolytes, urea, creatinine, liver function (ALT, ALP, albumin, bilirubin)
- Thyroid function blood test (TSH - V1 and V16 only [fT4 will only be performed if TSH is abnormal])
- Cardiac biomarkers (hsCRP, IL-6, Troponin I)
- Full lipid profile (at screening and V15 only)
- Serum pregnancy test where applicable
- Treg and Lymphocyte subset analysis
- PBMC assays

COVID-19 PCR testing

- COVID-19 swabs (nasal and throat) may be done as directed by local clinical guidelines/policies. These are in addition to the research protocol as per current clinical requirements which are subject to change over time.

11.7 End of Trial Participation

Patients will finish their trial participation at the end of V16. At the discretion of the PI, further visits or telephone contacts can be arranged to follow up unresolved issues or AEs until the point of resolution.

At the end of the trial patients will return to normal standard of care.

11.8 Trial restrictions

Both (sexually active) men and women should use at least two effective methods of contraception during the trial.

Women of childbearing potential (Peri-menopausal in the current trial) are required to use two of the following, reliable forms of contraception for the entire duration of the trial and for 1 month after the completion of the last treatment visit. This includes:

- Intrauterine Device (IUD, coil or intrauterine system)
- Oral contraception (either combined or progestogen alone)
- Contraceptive implant, injections or patches
- Condom **and** cap or diaphragm **plus** spermicide (chemical that kills sperm)
- Male sterilisation

Men are required to use two of the following reliable forms of contraception for the entire duration of the trial and for 1 month after the completion of the last treatment visit. This includes:

- a condom and spermicide (chemical that kills sperm), with female partner(s)using another method of contraception
- Men should also use a condom to protect male partners, or female partners who are pregnant or breastfeeding, from exposure to the trial medicine in semen.

Trial patients do not need to use contraception if:

- They are a woman who cannot become pregnant (e.g. have had a hysterectomy, removal of ovaries and/or sterilisation; or are post-menopausal for longer than two years)
- If they practice true abstinence (where this is in accordance with the patients preferred and usual lifestyle). If the patient becomes sexually active, they must use two of the methods listed above.

Patients should refrain from donating sperm and blood for the duration of the trial and for 1 month after completion of the last treatment visit.

12 Assessment of Safety

12.1 Definitions

12.1.1 Adverse event (AE)

Any untoward medical occurrence in a patient or clinical trial patient administered a medicinal product and which does not necessarily have a causal relationship with this treatment.

An adverse event can therefore be any unfavourable and unintended sign (including an abnormal laboratory finding), symptom, or disease temporally associated with the

use of an investigational medicinal product, whether or not considered related to the investigational medicinal product.

Please note: Recording of all adverse events must start from the point of Informed Consent regardless of whether a patient has yet received a medicinal product.

12.1.2 Adverse reaction to an investigational medicinal product (AR)

All untoward and unintended responses to an investigational medicinal product related to any dose administered. All adverse events judged by either the reporting investigator or the sponsor as having a reasonable causal relationship to a medicinal product qualify as adverse reactions. The expression reasonable causal relationship means to convey in general that there is evidence or argument to suggest a causal relationship.

12.1.3 Unexpected adverse reaction

An adverse reaction, the nature, or severity of which is not consistent with the applicable reference safety information (RSI) (e.g. investigator's brochure for an unapproved investigational product or summary of product characteristics (SmPC) for an authorised product).

When the adverse reaction is not consistent with the applicable RSI this adverse reaction should be considered as unexpected.

12.1.4 Serious adverse event or serious adverse reaction (SAE / SAR)

Any untoward medical occurrence that at any dose:

- results in death
- is life-threatening
- requires hospitalisation or prolongation of existing inpatients´ hospitalisation
- results in persistent or significant disability or incapacity
- is a congenital anomaly or birth defect.
- is an important medical event - Some medical events may jeopardise the patient or may require an intervention to prevent one of the above characteristics/ consequences. Such events (hereinafter referred to as 'important medical events') should also be considered as 'serious'

Life-threatening in the definition of a serious adverse event or serious adverse reaction refers to an event in which the patient was at risk of death at the time of event; it does not refer to an event which hypothetically might have caused death if it were more severe.

12.1.5 Suspected Unexpected Serious Adverse Reaction (SUSAR)

A serious adverse reaction, the nature and severity of which is not consistent with the information set out in the Reference Safety Information

12.1.6 Reference Safety Information (RSI)

A list of medical events that defines which reactions are expected for the IMP within a given trial and thus determining which Serious Adverse Reactions (SARs) require expedited reporting.

The RSI is contained in a clearly identified section of the Summary of Product Characteristics (SmPC) or the Investigator's Brochure (IB)

For this trial the Reference Safety Information is: Section 4.8 – Undesirable effects, of the SmPC for Proleukin (IL-2) powder for solution for injection or infusion (Clinigen Healthcare Ltd), dated 31 May 2019.

However, occurrence of the following events **if assessed as related to the IMP** and meeting the **Serious** criteria must be reported as Suspected Unexpected Serious Adverse Reactions (SUSARs):

- Tachycardia
- Arrhythmia
- Chest pain
- Cyanosis
- Transient ECG changes
- Myocardial ischaemia
- Palpitations
- Cardiac failure
- Myocarditis
- Cardiomyopathy
- Cardiac arrest
- Pericardial effusion
- Ventricular hypokinesia
- Cardiac tamponade
- Thrombosis
- Hypertension
- Hypotension
- Embolism.

12.2 Expected Adverse Reactions/Serious Adverse Reactions (AR /SARs)

All expected Adverse Reactions are listed in the latest MHRA approved version of the RSI as specified in section 12.1.6. This must be used when making a determination as to the expectedness of the adverse reaction.

The adverse reactions related to low dose IL-2 are generally not serious in nature and include:

- Systemic symptoms

The most frequently observed side effect is a flu-like syndrome characterised by fever, shivering, asthenia, rhinitis, arthralgia, myalgia and headaches. These symptoms are expected to occur in less than 20% of patients at the doses used in the present trial.

- Localised reaction at site of injection

Transient local reaction with symptoms of pain and signs of erythema or nodule: These are the most common non-serious adverse reactions and occur in approximately 50% of patients.

- Generalized gastrointestinal symptoms

Anorexia, nausea, vomiting, abdominal pain and diarrhoea: These symptoms are expected to occur in less than 20% of patients at the doses used in the present trial.

- Haematopoiesis

An initial decrease in absolute lymphocyte count that recovers in most patients after one week post treatment with IL-2: An initial increase in eosinophil count followed by a gradual decline after treatment. A decrease in platelet count of greater than 20% but less than 40% in some patients treated with repeated doses of subcutaneous low dose IL-2.

- Thyroid dysfunction

Thyroid function test (TFT) abnormalities have been noted in some patients treated with IL-2. After cessation of IL-2, TFTs returned to normal. A thyroid function test (TSH) will be checked at entry into the trial and at follow-up.

12.2.1 Injection site reaction

Injection site reactions (ISR) are a common occurrence and, based on data from previous studies, we would expect an incidence of at least 40-50% of all injection sites.

Injection site reaction is defined as: a localised reaction on the skin around the site of subcutaneous injection of the IMP. The reaction is comprised of a red or pink erythematous rash and localised pruritus can occur. Injection site reactions can sometimes also be associated with temporary bruising or nodule formation.

If any adverse reaction meets the criteria for seriousness, this must be reported as per section 12.5 of the protocol.

12.3 Expected Adverse Events/Serious Adverse Events (AE/SAE)

12.3.1 Hospital admissions

Current hospital admission with ACS is part of the inclusion criteria for this trial; therefore, the hospitalisation in this instance will not be recorded and reported as an SAE unless the patients' hospitalisation is prolonged due to reasons beyond routine clinical care for their condition. However, prolonged admission for social (i.e. non-clinical) reasons will not be reported as SAEs.

Planned/elective hospitalisation for further investigation and management of their disease will not be reported as SAEs. These events will be recorded in the trial CRF but simply not recorded as AEs or SAEs unless the event is deemed by the Principal Investigator to be an Adverse or Serious Adverse Reaction in which case it will be recorded as such. These may include but are not limited to in-patient tests/clinical management such as

1. Diagnostic angiograms +/- percutaneous coronary intervention (PCI)
2. Coronary artery bypass grafting
3. Vascular surgery due to complications of cardiac catheterisation/PCI
4. Other imaging/procedures due to complications of cardiac catheterisation/PCI

These events will be recorded in the trial CRF but not recorded as AEs or SAEs unless the event is deemed by the Principal Investigator to be an AR or SAR in which case it will be recorded/reported as such.

Due to the nature of the local hospital set up, ACS patients at Addenbrooke's routinely are transferred to Papworth for further investigation and treatment. This will not be an SAE.

12.3.2 Events commonly occurring in patients with ACS

The trial patients are acutely unwell and hospitalised therefore there are commonly occurring events which can be defined as:

a. Chest pain which is managed conservatively, by medication or by emergency transfer to another hospital.
b. Common procedure related complications and their treatment e.g. haematoma, pain (e.g analgesia), infection (e.g antibiotics), bleeding at vascular site (e.g blood transfusion), pseudo-aneurysms etc.
c. Post-procedural (post-angiography) troponin and CRP rises
d. Arrhythmias such as non-sustained ventricular tachycardia (NSVT) or temporary heart block
e. Reduction in myocardial systolic function leading to symptoms of heart failure

These events will be reported and causality determined by the CI on an individual basis.

12.4 Evaluation of adverse events

The Sponsor expects that adverse events are recorded from the point of Informed Consent regardless of whether a patient has yet received a medicinal product. Individual adverse events should be evaluated by the investigator. This includes the evaluation of its seriousness, and any relationship between the investigational medicinal product(s) and/or concomitant therapy and the adverse event (causality).

12.4.1 Assessment of seriousness

Seriousness is assessed against the criteria in section 12.1.4. This defines whether the event is an adverse event, serious adverse event or a serious adverse reaction

12.4.2 Assessment of causality

Definitely: A causal relationship is clinically/biologically certain. **This is therefore an Adverse Reaction**

Probable: A causal relationship is clinically / biologically highly plausible and there is a plausible time sequence between onset of the AE and administration of the investigational medicinal product and there is a reasonable response on withdrawal. **This is therefore an Adverse Reaction.**

Possible: A causal relationship is clinically / biologically plausible and there is a plausible time sequence between onset of the AE and administration of the investigational medicinal product. **This is therefore an Adverse Reaction.**

Unlikely: A causal relation is improbable and another documented cause of the AE is most plausible. **This is therefore an Adverse Event.**

Unrelated: A causal relationship can be definitely excluded and another documented cause of the AE is most plausible. **This is therefore an Adverse Event.**

Unlikely and Unrelated causalities are considered NOT to be trial drug related. Definitely, Probable and Possible causalities are considered to be trial drug related.

A pre-existing condition must not be recorded as an AE or reported as an SAE unless the condition worsens during the trial and meets the criteria for reporting or recording in the appropriate section of the CRF.

12.4.3 Clinical assessment of severity

Mild: The patient is aware of the event or symptom, but the event or symptom is easily tolerated

Moderate: The patient experiences sufficient discomfort to interfere with or reduce his or her usual level of activity

Severe: Significant impairment of functioning; the subject is unable to carry out usual activities and / or the patient's life is at risk from the event.

12.4.4 Recording of adverse events

Adverse events and adverse reactions should be recorded in the medical notes and the appropriate section of the CRF and/or AE/AR log. Adverse events will be MedDRA coded and entered into the trial database. Serious Adverse Events and Serious Adverse Reactions should be reported to the sponsor as detailed in section 12.5.

12.5 Reporting serious adverse events

The Principal Investigator needs to record all adverse events on the CRFs (except where **the protocol has stated otherwise - see section 12.3) and report all SAEs to CCTU within 24 hours by email** cambs.cardiovascular@nhs.net

Each Principal Investigator needs to record all adverse events and report serious adverse events to the Chief Investigator using the trial specific SAE form within 24 hours of their awareness of the event.

The Chief Investigator is responsible for ensuring the assessment of all SAEs for expectedness and relatedness is completed and the onward notification of all SAEs to the Sponsor immediately but not more than 24 hours of first notification. The sponsor has to keep detailed records of all SAEs reported to them by the trial team.

The Chief Investigator is also responsible for prompt reporting of all serious adverse event findings to the competent authority (e.g. MHRA) of each concerned Member State if they could:

- adversely affect the health of patients
- impact on the conduct of the trial
- alter the risk to benefit ratio of the trial
- alter the competent authority's authorisation to continue the trial in accordance with Directive 2001/20/EC

12.6 Reporting of Suspected Unexpected Serious Adverse Reactions (SUSARs)

All suspected adverse reactions related to an investigational medicinal product (the tested IMP and comparators) which occur in the concerned trial, and that are both unexpected and serious (SUSARs) are subject to expedited reporting. Please see section 12.1.6 for the Reference Safety Information to be used in this trial.

12.6.1. Who should report and whom to report to?

The Sponsor delegates the responsibility of notification of SUSARs to the Chief Investigator. The Chief Investigator must report all the relevant safety information previously described, to the:

- Sponsor
- competent authorities in the concerned member states (eg MHRA)

- Ethics Committee in the concerned member states

The Chief Investigator shall inform all investigators concerned of relevant information about SUSARs that could adversely affect the safety of patients.

12.6.2. When to report?

12.6.2.1. Fatal or life-threatening SUSARs

All parties listed in 12.6.1 must be notified as soon as possible but no later than **7 calendar days** after the trial team and Sponsor has first knowledge of the minimum criteria for expedited reporting.
In each case relevant follow-up information should be sought and a report completed as soon as possible. It should be communicated to all parties within an additional **8 calendar days**.

12.6.2.2. Non-fatal and non-life-threatening SUSARs

All other SUSARs and safety issues must be reported to all parties listed in 12.6.1 as soon as possible but no later than **15 calendar days** after first knowledge of the minimum criteria for expedited reporting. Further relevant follow-up information should be given as soon as possible.

12.6.3. How to report?

12.6.3.1. Minimum criteria for initial expedited reporting of SUSARs

Information on the final description and evaluation of an adverse reaction report may not be available within the required time frames for reporting. For regulatory purposes, initial expedited reports should be submitted within the time limits as soon as the minimum following criteria are met:

a) a suspected investigational medicinal product
b) an identifiable patient (e.g. trial patient code number)
c) an adverse event assessed as serious and unexpected, and for which there is a reasonable suspected causal relationship
d) an identifiable reporting source

and, when available and applicable:

- an unique clinical trial identification (EudraCT number or in case of non- European Community trials the sponsor's trial protocol code number)
- an unique case identification (i.e. sponsor's case identification number)

12.6.3.2. Follow-up reports of SUSARs

In case of incomplete information at the time of initial reporting, all the appropriate information for an adequate analysis of causality should be actively sought from the reporter or other available sources. Further available relevant information should be reported as follow-up reports.
In certain cases, it may be appropriate to conduct follow-up of the long-term outcome of a particular reaction.

12.6.3.3. Format of the SUSARs reports

Electronic reporting is the expected method for expedited reporting of SUSARs to the competent authority. The format and content as defined by the competent authority should be adhered to.

12.7 Pregnancy Reporting

All patient pregnancies within the trial must be reported to the Chief Investigator and the Sponsor using the relevant Pregnancy Reporting Form within 24 hours of notification. Pregnancies must be reported for all patients for the duration of their trial participation and for 1 month following the last dose of IMP.

Pregnancy is not considered an AE unless a negative or consequential outcome is recorded for the mother or child/foetus. If the outcome meets the serious criteria, this would be considered an SAE.

13 Toxicity – Emergency Procedures

In the event of an acute hypersensitivity reaction to IL-2 administration supportive care will be given to the patient according to local clinical procedures.

14 Evaluation of results

Please refer to section 8.6 Trial endpoints.

14.1 <u>Response criteria</u>

Please refer to section 8.6 Trial endpoints.

15 Storage and Analysis of Samples

Named samples may be directly sent to Cambridge University Hospitals NHS Foundation Trust pathology department or Royal Papworth Hospital pathology department for analysis and then destroyed shortly after. Some samples will be securely stored in freezers located on the Cambridge Biomedical Campus for analysis later. Patients on this trial will be assigned a unique trial number and this number will be used as an identifier for all stored samples collected from them. Authorised staff only will have access to the code and personal information about the donor. Only personnel authorised by the Chief Investigator and/or Principal Investigator(s) will be responsible for the storage, access and release of these samples for analysis. Stored samples may be analysed to fulfil endpoints as part of this trial or may be used in other ethically approved research with the patient's consent. Full details of how samples are processed, stored and analysed and if necessary destroyed for this trial can be found in the IVORY Trial Procedures Manual.

Blood samples from patients who were enrolled in the LILACS trial (EudraCT 2014-004979-23; NCT03113773) and who had provided consent for their blood samples to be stored for use in other ethically approved medical research after the end of the

LILACS trial will also be stored securely in freezers on the Cambridge Biomedical Campus.

16 Statistics

16.1 Statistical methods

Three approaches will be used to quantify vascular inflammation: 1) The pre-specified whole vessel primary endpoint of change in the average maximum TBR (TBR_{max}) for all segments within the index vessel[48], with a complementary analysis displaying the group distribution of average TBR_{max} using a frequency histogram; 2) An analysis of change from baseline in average TBR_{max} for active segments within the index vessel; and 3) an analysis of the probability of a segment being active within the index vessel.

Change from baseline in average TBR_{max} will be analysed using analysis of covariance, fitting treatment as fixed effect, and including baseline value as a covariate and ST-elevation status as covariate. Point estimates and corresponding 95% confidence intervals (CI) will be constructed for the relevant comparisons of interest.

Average TBR_{max} data from the final visit will be plotted to show the distribution from all segments from all index vessels within each treatment group. The difference between low-dose IL-2 and placebo will be calculated and tested using a nonparametric permutation test at the patient level.

The number of active segments and the total number of segments will be included in logistic regression analyses to model the probability of a segment being active. For baseline correction within each group, a model will be fitted with terms for treatment and day. For placebo and baseline correction, a model will be fitted with treatment term and including the baseline proportion of active segments as covariate. Point estimates and corresponding 95% CI are constructed to establish the odds ratio for the relevant comparisons of interest.

Biomarker data are analysed by analysis of covariance fitting terms for regimen, day, and interaction of day and regimen as fixed effects; patient as a random effect; and baseline biomarker at day 1 as a covariate.

Summary statistics of endpoints broken down by treatment arm and visit where appropriate will be provided. Categorical variables will report percentages and counts (p% (x/n)); continuous variables will report, mean, median, SD, min and max. Exploratory figures in the form of stacked bar-charts and box-and-whisker plots for categorical and continuous variables respectively will be provided.

A detailed statistical analysis plan will be produced before the final database lock.

16.2 Interim analyses

There are no planned interim analyses for this trial.

16.3 Number of Patients to be enrolled

Sample size is based on an absolute difference of 0.2 in the primary endpoint (TBR_{max} in the index vessel) between placebo and active treatment at the end of the treatment period. This is equivalent to a 10% difference from a reference value of 2.02 and equivalent to the size effect observed after atheroprotective therapy[39,42,43]. Interventions that have reported less than 10% difference in TBR_{max} in the index vessel

between active treatment and placebo at the end of the treatment period [40,49] failed to reduce CV outcomes[50-52].

Assuming a SD of 0.24[39], 24 patients per arm, testing at 2-sided 5% significance level, will provide 80% power. Therefore, a sample size of 30 completed patients per arm was selected to account for scans which may not be analysable, or poor scans due to patient habitus, movement and so on.

This sample size of n=30 per group allows the detection of a baseline-corrected 6% difference in TBR_{max} in the index vessel for the trial primary endpoint (mean 0.125, SD 0.166)[39] between placebo and active treatment at the end of the treatment period, at 2-sided 5% significance level and 80% power.

16.3.1 Safety Population

Safety reporting will be based upon the safety population defined as any patients who receive any trial drug or placebo dose.

16.3.2 Analysis Population

The reporting of biomarker endpoints, including Tregs, will be based upon the Analysis Population defined as patients who receive trial drug or placebo and complete the treatment course after randomisation.

16.4 Procedure to account for missing or spurious data

We do not anticipate a high level of drop out. Hence we will report the number of non-missing values for comparison to the relevant population size. Statistics will be calculated based on complete cases, which assumes there is no association between a value being unobserved, and the underlying value.

16.5 Definition of the end of the trial

The end of trial will be the date 18 months after the last patient's last visit to allow sufficient time to complete all primary, secondary, and exploratory endpoints and their corresponding analyses, and if applicable, all re-analyses of samples.

17 Data handling and record keeping

17.1 CRF

All data will be transferred into a Case Report Form (CRF) which will be anonymised. All trial data in the CRF must be extracted from and be consistent with the relevant source documents. The CRFs must be completed, dated and signed by the investigator or designee in a timely manner. It remains the responsibility of the investigator for the timing, completeness, legibility and accuracy of the CRF. The CRF will be accessible to trial coordinators, data managers, the investigators, Clinical Trial Monitors, Auditors and Inspectors as required. Completed originals of the CRFs should be sent to the trial coordination centre within timeframes outlined in the Data Management Plan.

The investigator will retain a copy of each completed CRF page at site. The investigator will supply the trial coordination centre with any required, anonymised background information from the medical records as required.

Any trial related documentation that is sent to the trial coordination centre must not contain patient identifiable data.

All CRF pages must be clear, legible and completed in black ink. Any errors should be crossed with a single stroke so that the original entry can still be seen. Corrections should be inserted and the change dated and initialled by the investigator or designee. If it is not clear why the change has been made, an explanation should be written next to the change. Typing correction fluid must not be used.

17.2 Source Data

To enable peer review, monitoring, audit and/or inspection the investigator must agree to keep records of all participating patients (sufficient information to link records e.g. hospital records and samples) and all original signed informed consent forms.

Source data may include but is not limited to:

- Signed informed consent forms
- Patient medical records (electronic or paper)
- Pre-defined sections of the Case Report Forms (CRFs) detailed in the Data Management Plan
- Blood results (electronic or paper)
- Sample logs
- Prescriptions
- ECHO results
- ECG print outs
- ^{18}F-FDG PET/CT results

17.3 Data Protection & Patient Confidentiality

All investigators and trial site staff involved in this trial must comply with the requirements of the Data Protection Act 2018 and Trust Policy with regards to the collection, storage, processing, transfer and disclosure of personal information and will uphold the Act’s core principles.

18 Trial Management Group

The Trial Management Group (TMG) will be comprised of blinded individuals who are responsible for the day to day running of the trial.

The TMG will meet on a regular basis and will be responsible for the overall supervision of trial progress. The TMG will make major decisions about the trial including whether protocol amendments are required, and will discuss recommendations from the Data Monitoring Committee (DMC). The TMG will also be notified about all patient withdrawals including those arising from discontinuation of dosing.

The TMG will be comprised of:

- The Chief Investigator and collaborators
- A research nurse
- The trial coordinator

The trial statistician will be unblinded.

19 Independent Data Monitoring Committee

The DMC will be comprised of an unblinded independent group, as defined in a separate charter document which will define the role of the DMC. The charter document will be generated prior to opening the trial.

The DMC will be responsible for the review of all safety (but not exploratory) data and will meet quarterly for the first year of the trial opening to recruitment and every six months until the follow-up visit of the last patient.

Additionally, ad hoc IDMC meetings will be triggered if conditions are met as specified in section 9.6 Trial Stopping Criteria.

20 Ethical & Regulatory considerations

20.1 Ethical committee review

Before the start of the trial or implementation of any amendment we will obtain approval of the trial protocol, protocol amendments, informed consent forms and other relevant documents e.g., advertisements and GP information letters if applicable from the REC. All correspondence with the REC will be retained in the Trial Master File/Investigator Site File.

Annual reports will be submitted to the REC in accordance with national requirements. It is the Chief Investigator's responsibility to produce the annual reports as required.

20.2 Regulatory Compliance

The trial will not commence until a Clinical Trial Authorisation (CTA) is obtained from the MHRA. The protocol and trial conduct will comply with the Medicines for Human Use (Clinical Studies) Regulations 2004 and any relevant amendments.

Development Safety Update Reports (DSURs) will be submitted to the MHRA in accordance with national requirements. It is the Chief Investigator's responsibility to produce the annual reports as required.

20.3 Protocol Amendments

Protocol amendments must be reviewed and agreement received from the Sponsor for all proposed amendments prior to submission.

The only circumstance in which an amendment may be initiated prior to MHRA, HRA and REC approval is where the change is necessary to eliminate apparent, immediate risks to the patients (Urgent Safety Measures). In this case, accrual of new patients will be halted until the MHRA, HRA and REC approval has been obtained.

20.4 Peer Review

This trial protocol has been reviewed by a specialist independent group within the Sponsor organisation (Cambridge University Hospitals NHS Foundation Trust Research Advisory Committee).

20.5 Declaration of Helsinki and Good Clinical Practice

The trial will be performed in accordance with the spirit and the letter of the declaration of Helsinki, the conditions and principles of Good Clinical Practice, the protocol and applicable local regulatory requirements and laws.

20.6 GCP Training

All trial staff must hold evidence of appropriate GCP training or undergo GCP training prior to undertaking any responsibilities on this trial. This training should be updated every 2 years or in accordance with your Trust's policy.

21 Sponsorship, Financial and Insurance

The trial will be sponsored by Cambridge University Hospitals NHS Foundation Trust and University of Cambridge. The trial is funded by the MRC.

Cambridge University Hospitals NHS Foundation Trust, as a member of the NHS Clinical Negligence Scheme for Trusts, will accept full financial liability for harm caused to patients in the clinical trial caused through the negligence of its employees and honorary contract holders. There are no specific arrangements for compensation should a patient be harmed through participation in the trial, but no-one has acted negligently.

22 Monitoring, Audit & Inspection

The investigator must make all trial documentation and related records available should an MHRA Inspection occur. Should a monitoring visit or audit be requested, the investigator must make the trial documentation and source data available to the Sponsor's representative. All patient data must be handled and treated confidentially.

The Sponsor's monitoring frequency will be determined by an initial risk assessment performed prior to the start of the trial. A detailed monitoring plan will be generated detailing the frequency and scope of the monitoring for the trial. Throughout the course of the trial, the risk assessment will be reviewed and the monitoring frequency adjusted as necessary.

23 Protocol Compliance and Breaches of GCP

Prospective, planned deviations or waivers to the protocol are not allowed under the UK regulations on Clinical Studies and must not be used.

Protocol deviations, non-compliances, or breaches are departures from the approved protocol. They can happen at any time, but are not planned. They must be adequately documented on the relevant forms and reported to the Chief Investigator and Sponsor immediately.

Deviations from the protocol which are found to occur constantly again and again will not be accepted and will require immediate action and could potentially be classified as a serious breach.

Any potential/suspected serious breaches of GCP must be reported immediately to the Sponsor without any delay.

24 Publications policy

Ownership of the data arising from this trial resides with the trial team. On completion of the trial the data will be analysed and tabulated and a Final Trial Report prepared.

25 References

1 Libby, P. Mechanisms of acute coronary syndromes and their implications for therapy. *The New England journal of medicine* **368**, 2004-2013, doi:10.1056/NEJMra1216063 (2013).

2 Arbab-Zadeh, A., Nakano, M., Virmani, R. & Fuster, V. Acute coronary events. *Circulation* **125**, 1147-1156, doi:10.1161/circulationaha.111.047431 (2012).

3 Mega, J. L. *et al.* Rivaroxaban in patients with a recent acute coronary syndrome. *The New England journal of medicine* **366**, 9-19, doi:10.1056/NEJMoa1112277 (2012).

4 Wiviott, S. D. *et al.* Prasugrel versus clopidogrel in patients with acute coronary syndromes. *The New England journal of medicine* **357**, 2001-2015, doi:10.1056/NEJMoa0706482 (2007).

5 Liuzzo, G. *et al.* The prognostic value of C-reactive protein and serum amyloid a protein in severe unstable angina. *The New England journal of medicine* **331**, 417-424, doi:10.1056/nejm199408183310701 (1994).

6 Ritchie, M. E. Nuclear factor-kappaB is selectively and markedly activated in humans with unstable angina pectoris. *Circulation* **98**, 1707-1713 (1998).

7 Buffon, A. *et al.* Widespread coronary inflammation in unstable angina. *The New England journal of medicine* **347**, 5-12, doi:10.1056/NEJMoa012295 (2002).

8 Falk, E., Nakano, M., Bentzon, J. F., Finn, A. V. & Virmani, R. Update on acute coronary syndromes: the pathologists' view. *European heart journal* **34**, 719-728, doi:10.1093/eurheartj/ehs411 (2013).

9 Dutta, P. *et al.* Myocardial infarction accelerates atherosclerosis. *Nature* **487**, 325-329, doi:10.1038/nature11260 (2012).

10 Hansson, G. K. Inflammation, atherosclerosis, and coronary artery disease. *The New England journal of medicine* **352**, 1685-1695, doi:10.1056/NEJMra043430 (2005).

11 McPherson, R. & Davies, R. W. Inflammation and coronary artery disease: insights from genetic studies. *The Canadian journal of cardiology* **28**, 662-666, doi:10.1016/j.cjca.2012.05.014 (2012).

12 Liuzzo, G. *et al.* Unusual CD4+CD28null T lymphocytes and recurrence of acute coronary events. *Journal of the American College of Cardiology* **50**, 1450-1458, doi:10.1016/j.jacc.2007.06.040 (2007).

13 Ammirati, E. *et al.* Expansion of T-cell receptor zeta dim effector T cells in acute coronary syndromes. *Arteriosclerosis, thrombosis, and vascular biology* **28**, 2305-2311, doi:10.1161/atvbaha.108.174144 (2008).

14 Caligiuri, G., Paulsson, G., Nicoletti, A., Maseri, A. & Hansson, G. K. Evidence for antigen-driven T-cell response in unstable angina. *Circulation* **102**, 1114-1119 (2000).

15 Ammirati, E. *et al.* Circulating CD4+CD25hiCD127lo regulatory T-Cell levels do not reflect the extent or severity of carotid and coronary atherosclerosis. *Arteriosclerosis, thrombosis, and vascular biology* **30**, 1832-1841, doi:10.1161/atvbaha.110.206813 (2010).

16 Cheng, X. *et al.* The Th17/Treg imbalance in patients with acute coronary syndrome. *Clinical immunology (Orlando, Fla.)* **127**, 89-97, doi:10.1016/j.clim.2008.01.009 (2008).

17 Han, S. F. *et al.* The opposite-direction modulation of CD4+CD25+ Tregs and T helper 1 cells in acute coronary syndromes. *Clinical immunology (Orlando, Fla.)* **124**, 90-97, doi:10.1016/j.clim.2007.03.546 (2007).

18 Mor, A., Luboshits, G., Planer, D., Keren, G. & George, J. Altered status of CD4(+)CD25(+) regulatory T cells in patients with acute coronary syndromes. *European heart journal* **27**, 2530-2537, doi:10.1093/eurheartj/ehl222 (2006).

19 Li, Q. *et al.* Distinct different sensitivity of Treg and Th17 cells to Fas-mediated apoptosis signaling in patients with acute coronary syndrome. *International journal of clinical and experimental pathology* **6**, 297-307 (2013).

20 Wigren, M. *et al.* Low levels of circulating CD4+FoxP3+ T cells are associated with an increased risk for development of myocardial infarction but not for stroke. *Arteriosclerosis, thrombosis, and vascular biology* **32**, 2000-2004, doi:10.1161/atvbaha.112.251579 (2012).

21 Hofmann, U. *et al.* Activation of CD4+ T lymphocytes improves wound healing and survival after experimental myocardial infarction in mice. *Circulation* **125**, 1652-1663, doi:10.1161/circulationaha.111.044164 (2012).
22 Zhang, M. *et al.* Identification of the target self-antigens in reperfusion injury. *The Journal of experimental medicine* **203**, 141-152, doi:10.1084/jem.20050390 (2006).
23 Dobaczewski, M., Xia, Y., Bujak, M., Gonzalez-Quesada, C. & Frangogiannis, N. G. CCR5 signaling suppresses inflammation and reduces adverse remodeling of the infarcted heart, mediating recruitment of regulatory T cells. *The American journal of pathology* **176**, 2177-2187, doi:10.2353/ajpath.2010.090759 (2010).
24 Matsumoto, K. *et al.* Regulatory T lymphocytes attenuate myocardial infarction-induced ventricular remodeling in mice. *International heart journal* **52**, 382-387 (2011).
25 Tang, T. T. *et al.* Regulatory T cells ameliorate cardiac remodeling after myocardial infarction. *Basic research in cardiology* **107**, 232, doi:10.1007/s00395-011-0232-6 (2012).
26 Mausner-Fainberg, K. *et al.* The effect of HMG-CoA reductase inhibitors on naturally occurring CD4+CD25+ T cells. *Atherosclerosis* **197**, 829-839, doi:10.1016/j.atherosclerosis.2007.07.031 (2008).
27 Zhang, D. *et al.* Effect of oral atorvastatin on CD4+CD25+ regulatory T cells, FoxP3 expression, and prognosis in patients with ST-segment elevated myocardial infarction before primary percutaneous coronary intervention. *Journal of cardiovascular pharmacology* **57**, 536-541, doi:10.1097/FJC.0b013e318211d016 (2011).
28 Platten, M. *et al.* Blocking angiotensin-converting enzyme induces potent regulatory T cells and modulates TH1- and TH17-mediated autoimmunity. *Proceedings of the National Academy of Sciences of the United States of America* **106**, 14948-14953, doi:10.1073/pnas.0903958106 (2009).
29 Malek, T. R. & Bayer, A. L. Tolerance, not immunity, crucially depends on IL-2. *Nature reviews. Immunology* **4**, 665-674, doi:10.1038/nri1435 (2004).
30 Wing, K. & Sakaguchi, S. Regulatory T cells exert checks and balances on self tolerance and autoimmunity. *Nature immunology* **11**, 7-13, doi:10.1038/ni.1818 (2010).
31 Malek, T. R. The biology of interleukin-2. *Annual review of immunology* **26**, 453-479, doi:10.1146/annurev.immunol.26.021607.090357 (2008).
32 Dinh, T. N. *et al.* Cytokine therapy with interleukin-2/anti-interleukin-2 monoclonal antibody complexes expands CD4+CD25+Foxp3+ regulatory T cells and attenuates development and progression of atherosclerosis. *Circulation* **126**, 1256-1266, doi:10.1161/circulationaha.112.099044 (2012).
33 Foks, A. C. *et al.* Differential effects of regulatory T cells on the initiation and regression of atherosclerosis. *Atherosclerosis* **218**, 53-60, doi:10.1016/j.atherosclerosis.2011.04.029 (2011).
34 Koreth, J. *et al.* Interleukin-2 and regulatory T cells in graft-versus-host disease. *The New England journal of medicine* **365**, 2055-2066, doi:10.1056/NEJMoa1108188 (2011).
35 Matsuoka, K. *et al.* Low-dose interleukin-2 therapy restores regulatory T cell homeostasis in patients with chronic graft-versus-host disease. *Science translational medicine* **5**, 179ra143, doi:10.1126/scitranslmed.3005265 (2013).
36 Saadoun, D. *et al.* Regulatory T-cell responses to low-dose interleukin-2 in HCV-induced vasculitis. *The New England journal of medicine* **365**, 2067-2077, doi:10.1056/NEJMoa1105143 (2011).
37 Rosenzwajg, M. *et al.* Low-dose interleukin-2 fosters a dose-dependent regulatory T cell tuned milieu in T1D patients. *Journal of autoimmunity* **58**, 48-58, doi:10.1016/j.jaut.2015.01.001 (2015).
38 Klatzmann, D. & Abbas, A. K. The promise of low-dose interleukin-2 therapy for autoimmune and inflammatory diseases. *Nature reviews. Immunology* **15**, 283-294, doi:10.1038/nri3823 (2015).

39 Elkhawad, M. *et al.* Effects of p38 mitogen-activated protein kinase inhibition on vascular and systemic inflammation in patients with atherosclerosis. *JACC. Cardiovascular imaging* **5**, 911-922, doi:10.1016/j.jcmg.2012.02.016 (2012).
40 Fayad, Z. A. *et al.* Safety and efficacy of dalcetrapib on atherosclerotic disease using novel non-invasive multimodality imaging (dal-PLAQUE): a randomised clinical trial. *Lancet (London, England)* **378**, 1547-1559, doi:10.1016/s0140-6736(11)61383-4 (2011).
41 Tarkin, J. M., Joshi, F. R. & Rudd, J. H. PET imaging of inflammation in atherosclerosis. *Nature reviews. Cardiology* **11**, 443-457, doi:10.1038/nrcardio.2014.80 (2014).
42 Maki-Petaja, K. M. *et al.* Anti-tumor necrosis factor-alpha therapy reduces aortic inflammation and stiffness in patients with rheumatoid arthritis. *Circulation* **126**, 2473-2480, doi:10.1161/circulationaha.112.120410 (2012).
43 Tahara, N. *et al.* Simvastatin attenuates plaque inflammation: evaluation by fluorodeoxyglucose positron emission tomography. *Journal of the American College of Cardiology* **48**, 1825-1831, doi:10.1016/j.jacc.2006.03.069 (2006).
44 Figueroa, A. L. *et al.* Measurement of arterial activity on routine FDG PET/CT images improves prediction of risk of future CV events. *JACC. Cardiovascular imaging* **6**, 1250-1259, doi:10.1016/j.jcmg.2013.08.006 (2013).
45 Marnane, M. *et al.* Carotid plaque inflammation on 18F-fluorodeoxyglucose positron emission tomography predicts early stroke recurrence. *Annals of neurology* **71**, 709-718, doi:10.1002/ana.23553 (2012).
46 van der Valk, F. M. *et al*. Thresholds for Arterial Wall Inflammation Quantified by 18F-FDG PET-CT Imaging: Implications for Vascular Interventional Studies. *JACC. Cardiovascular Imaging*, **9**, 1198-1207. Doi:10.1016/j.jcmg.2016.04.007 (2016).
47 Bucerius, J. *et al.* Position paper of the Cardiovascular Committee of the European Association of Nuclear Medicine (EANM) on PET imaging of atherosclerosis. *European journal of nuclear medicine and molecular imaging* **43**, 780-792, doi:10.1007/s00259-015-3259-3 (2016).
48 Rudd, J. H. *et al.* Atherosclerosis inflammation imaging with 18F-FDG PET: carotid, iliac, and femoral uptake reproducibility, quantification methods, and recommendations. *Journal of nuclear medicine : official publication, Society of Nuclear Medicine* **49**, 871-878, doi:10.2967/jnumed.107.050294 (2008).
49 Tawakol, A. *et al.* Effect of treatment for 12 weeks with rilapladib, a lipoprotein-associated phospholipase A2 inhibitor, on arterial inflammation as assessed with 18F-fluorodeoxyglucose-positron emission tomography imaging. *Journal of the American College of Cardiology* **63**, 86-88, doi:10.1016/j.jacc.2013.07.050 (2014).
50 O'Donoghue, M. L., Braunwald, E., White, H. D. & et al. Effect of darapladib on major coronary events after an acute coronary syndrome: The solid-timi 52 randomized clinical trial. *JAMA* **312**, 1006-1015, doi:10.1001/jama.2014.11061 (2014).
51 Schwartz, G. G. *et al.* Effects of dalcetrapib in patients with a recent acute coronary syndrome. *The New England journal of medicine* **367**, 2089-2099, doi:10.1056/NEJMoa1206797 (2012).
52 White, H. D. *et al.* Darapladib for preventing ischemic events in stable coronary heart disease. *The New England journal of medicine* **370**, 1702-1711, doi:10.1056/NEJMoa1315878 (2014).
53 Zhao, T., Sriranjan, R., Tuong, K., Lu, Y., Sage, A., Nus Chimeno, M., Hubsch, A., et al. Regulatory T Cell Response to Low-Dose IL-2 in Ischemic Heart Disease. *New England Journal of Medicine Evidence* https://doi.org/10.17863/CAM.77239

26 Appendices

26.1 Appendix 1 – Symptoms and signs documented as AEs in the LILACS trial

(MHRA reference 24551/0029/001-0001; REC reference 17/NW/0012; ClinicalTrials.gov Identifier NCT03113773)

AE description	Frequency	Severity	Relatedness as determined by the blinded investigator
**Chest infection**	2	mild	unrelated
**Chest tightness**	1	mild	unrelated
**Diarrhoea**	1	mild	unrelated
**Dizziness**	1	moderate	unrelated
**Free T4 low**	1	mild	unrelated
**Gastro oesophageal reflux**	1	mild	unrelated
**GI disturbance**	1	mild	unrelated
**Groin thrush**	1	mild	unrelated
**Muscle ache**	1	mild	unrelated
**Productive cough post op.**	1	mild	unrelated
**Raised BNP**	1	mild	unrelated
**Raised troponin**	1	mild	unrelated
**Right arm ache**	1	mild	unrelated
**Right hand petechiae**	1	mild	unrelated
**Right scapula ache**	1	mild	unrelated
**Right ventricular impairment**	1	mild	unrelated
**TSH low**	1	mild	unrelated
**Vomiting**	1	moderate	unrelated
**Chest pain**	5	mild	unlikely 2, unrelated 3
**Dizziness**	3	mild	unlikely 1, unrelated 2
**Dry cough**	2	mild	unlikely 1, unrelated 1
**Angina**	2	mild	unlikely
**Back pain**	1	mild	unlikely
**Blocked sinuses**	1	mild	unlikely
**Fall**	1	mild	unlikely
**Finger pain**	1	mild	unlikely
**Gastro oesophageal reflux**	1	moderate	unlikely
**Gout right toe**	1	mild	unlikely
**Inflamed haematoma - right forearm**	1	mild	unlikely
**Nasal congestion**	1	mild	unlikely
**Non-productive cough**	1	mild	unlikely
**Pyrexia**	1	mild	unlikely
**Sinusitis**	1	mild	unlikely
**Hand joint pain**	1	moderate	probably
**Injection site adverse reaction**	93	mild	probably
**Injection site bruise**	3	mild	probably
**Injection site nodule**	12	mild	probably
**Injection site pruritus**	2	mild	probably

**Leg ache (bilateral)**	1	mild	probably
**Fatigue**	10	mild	possibly 6, probably 4
**Tiredness**	4	mild	possibly 2, unlikely 2
**Isolated episode pyrexia**	3	mild	possibly 2, unlikely 1
**Headache**	4	mild	possibly 2, probably 1, unlikely 1
**Body ache**	3	mild	possibly 2, probably 1
**Breathlessness**	2	mild	possibly 1, unrelated 1
**Headache**	2	moderate	possibly 1, unrelated 1
**Flu like syndrome**	9	mild	possibly 1, probably 8
**Elevated CRP**	1	mild	possibly
**Knee pain (intermittent at night)**	1	mild	possibly
**Sore throat**	2	mild	possibly

26.2 Appendix 2 - Safety reporting flow-chart

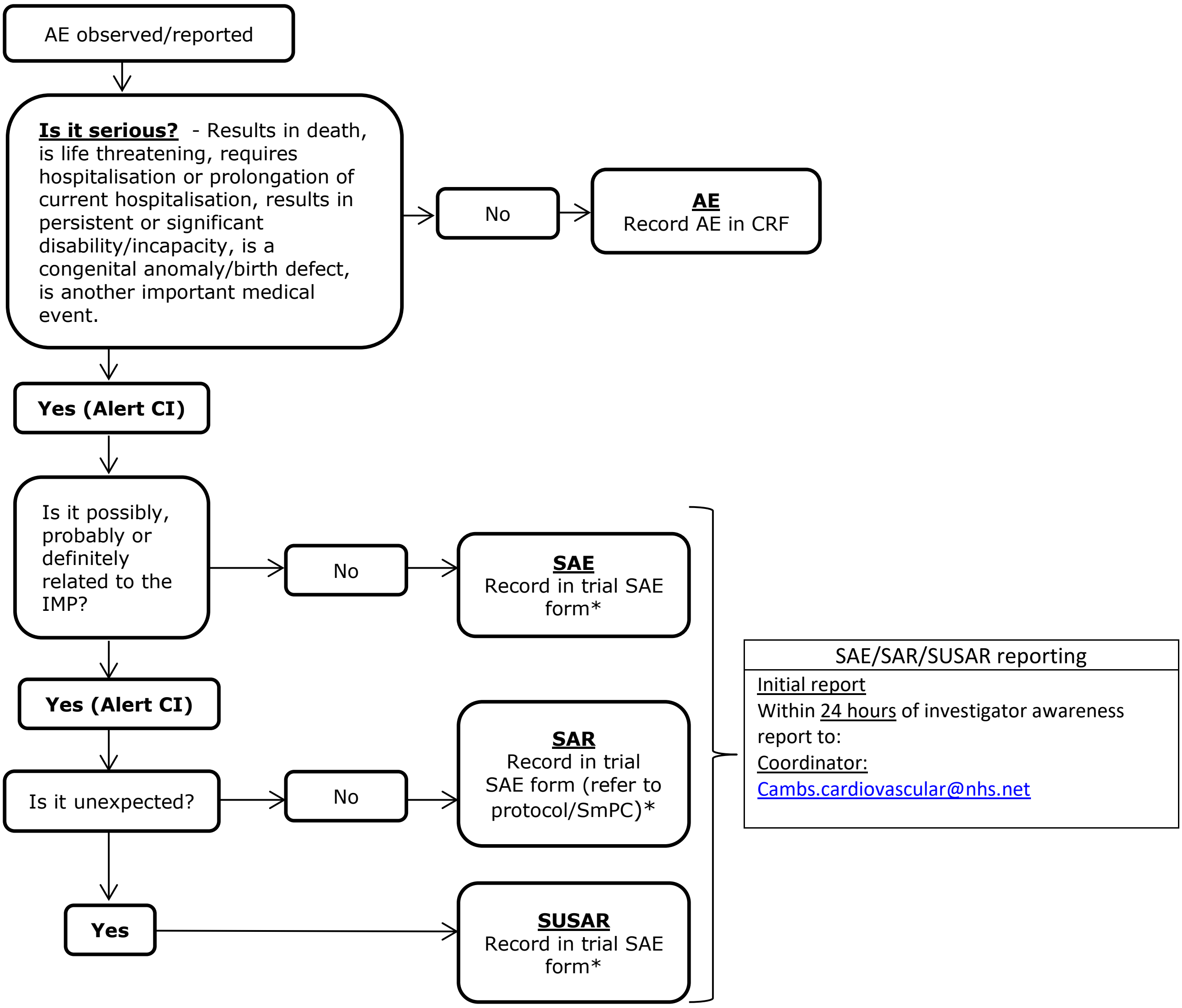

*First 10 patients dosed: A cumulative total of 3 events triggers IDMC; trial recruitment and dosing will halt. After the tenth (10th) patient has started treatment and 30% patients experience pre-specified events (see protocol section 9.6/ SmPC RSI in 4.8 for specified events) trial recruitment and dosing will halt.

SUSAR expedited reporting
The Chief Investigator must report a SUSAR to the Sponsor, REC and MHRA within statutory timelines. Each SUSAR requires the entry of relevant data and information by the Chief Investigator into the eSUSAR reporting system. A copy of this report should be provided to the CCTU PV team, collating these on behalf of the sponsor.

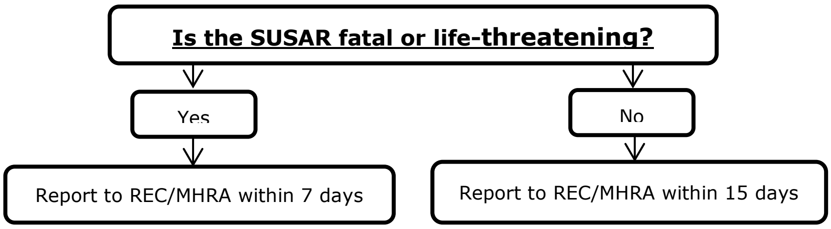

,
CCTU/TPL007 V5 Approved: 27/09/2021 Reviewed: 27/09/2021

Statistical Analysis Plan

TRIAL FULL TITLE	Low-dose interleukin-2 for the reduction of vascular inflammation in Acute Coronary Syndromes (IVORY)
IRAS ID	220945
SAP VERSION	2.0
ISRCTN NUMBER	
SAP VERSION DATE	
TRIAL STATISTICIAN	Simon Bond
TRIAL CHIEF INVESTIGATOR	Dr Joseph Cheriyan
SAP AUTHOR	Simran Vaja , Simon Bond

1 SAP Signatures

I give my approval for the attached SAP entitled IVORY dated 18OCT2023

Chief Investigator

Name: Dr Joseph Cheriyan

Signature: ______________________________

Date: ______________________

Statistician

Name: Dr Simon Bond

Signature: ______________________

Date: ______________________

2 Table of Contents

1 SAP Signatures 1
2 Table of Contents 2
3 Abbreviations and Definitions 4
4 Introduction 6
4.1 Preface 6
4.2 Purpose of the analyses 6
5 Study Objectives and Endpoints 6
5.1 Study Objectives 6
5.2 Endpoints 7
5.2.1 Primary endpoint: 7
5.2.2 Secondary endpoints: 8
5.2.3 Exploratory endpoints: 8
6 Study Methods 9
6.1 General Study Design and Plan 9
6.2 Inclusion-Exclusion Criteria and General Study Population 10
6.2.1 Inclusion Criteria 10
6.2.2 Exclusion Criteria 11
6.3 Randomisation and Blinding 13
6.4 Study Variables 13
7 Sample Size 15
8 General Considerations 15
8.1 Timing of Analyses 15
8.2 Analysis Populations 16
8.2.1 Full Analysis Population 16
8.2.2 Randomised Population 16
8.2.3 Safety population 16
8.2.4 Non-safety population 16

8.3 Covariates and Subgroups 17
8.3.1 Subgroup analysis 17
8.4 Missing Data 17
9 Summary of Study Data 17
9.1 Participant Disposition 18
9.2 Derived variables 19
9.2.1 Primary endpoint 19
9.2.2 Active slice analysis 20
9.2.3 Slice Matching 20
9.2.4 High Dose Statins 21
9.2.5 CTCA, stool etc. 21
9.3 Protocol Deviations 21
9.4 Demographic and Baseline Variables 21
9.4.1 Demographics 21
9.4.2 Medical history 22
9.4.3 Intervention log details 22
9.5 Concurrent Illnesses and Medical Conditions 22
9.6 Treatment Compliance 22
10 Efficacy Analyses 22
10.1 Primary Efficacy Analysis 22
10.2 Secondary Analyses 23
10.2.1 Baseline-Active Slice Analysis using mean TBRmax 23
10.2.2 Probability of a slice being active within the index vessel 25
10.2.3 Subgroup Analysis 26
10.2.4 Sensitivity Analyses: 26
10.3 Other analyses using the index vessel 27
10.3.1 The distribution of all slices from the index vessels 27
10.4 All-vessel analysis 27

10.5 Lymphocyte Subsets 27
10.6 Exploratory Efficacy Analyses 28
10.6.1 Cardiac Biomarkers 28
10.6.2 Ejection Fraction 29
10.6.3 Full Lipid Profile 29
11 Safety Analyses 29
11.1 Adverse Events 29
11.2 Deaths, Serious Adverse Events and other Significant Adverse Events 29
11.3 Pregnancies 29
11.4 Clinical Laboratory Evaluations 29
11.4.1 Safety bloods 30
11.4.2 Vital signs 30
11.5 Other Safety Measures 30
11.5.1 Physical examination 30
11.5.2 Concomitant medications 30
11.5.3 12-lead electrocardiogram (ECGs) recordings 31
12 Figures 31
13 Reporting Conventions 31
14 Technical Details 31
15 Summary of Changes to the Protocol or from Previous Version of the SAP 32
15.1 Changes from SAP V1 33
16 References 33

3 Abbreviations and Definitions

AE/AR	Adverse event/Adverse Reaction
ACS	Acute Coronary Syndrome
ALP	Alkaline phosphatase
ALT	Alanine aminotransferase
AST	Aspartate aminotransferase

BNP	B-type Natriuretic Peptide
CA	Competent Authority
CABG	Coronary artery bypass graft
CCTU	Cambridge Clinical Trials Unit
CI	Chief investigator
CNS	Central nervous system
CRF	Case Report Form
CT	Computed Tomography
CV	cardiovascular
DMC	Data Monitoring Committee
DSUR	Development Safety Update Report
ECG	Electrocardiogram
FDG - PET/CT	Fluorodeoxyglucose - Positron emission tomography/ computed tomography
GCP	Good Clinical Practice
GP	General Practitioner
hs-CRP	High-Sensitivity C-Reactive Protein
ICF	Informed Consent Form
IHD	Ischaemic heart disease
IMP	Investigational Medicinal Product
ISR	Injection site reaction
IU	International Unit
MHRA	Medicines and Healthcare products Regulatory Agency
NIMP	Non Investigational Medicinal Product
NSTEMI	Non-ST Elevation Myocardial Infarction
PBMC	Peripheral Blood Mononuclear Cell assay
PCI	Percutaneous coronary intervention
PET	Positron Emission Tomography
PIS	Patient Information Sheet
QTcB	Corrected QT using Bazett's formula
R&D	Research and Development
RA	Regulatory Agency
REC	Research Ethics Committee
RSI	Reference Safety Information
SAE/SAR	Serious Adverse Event/Serious Adverse Reaction
SmPC	Summary of Product Characteristics
STEMI	ST elevation myocardial infarction
ST	ECG parameter
SUSAR	Suspected Unexpected Serious Adverse Reaction
SUV	Standardized Uptake Value

TBR	Tissue-to-blood ratio
TMG	Trial Management Group
TnI	Troponin I
Treg	Regulatory T cells
TSH	Thyroid Stimulating Hormone
TTE	Transthoracic echocardiogram
ULN	Upper Limit of Normal

4 Introduction

4.1 Preface

Acute coronary syndromes (ACS) result from coronary plaque(s) disruption, which initiates a thrombotic process leading to partial or complete obstruction of the vessel lumen with subsequent myocardial ischaemia and necrosis[1,2]. The mainstay of treatment is currently focussed on the re-establishment and maintenance of coronary artery patency using anti-platelets and anticoagulants with or without mechanical dilatation and stenting of the culprit artery[1,2]. Despite important advances in management, ACS still carries a risk of substantial morbidity and mortality[1]. The improved efficacy of novel anti-platelet and anticoagulant agents have been limited by increased risk of haemorrhagic events[3,4]. Thus, future breakthroughs in management are most likely to arise from targeting other relevant pathophysiological pathways. Particularly, we believe that the immune response is an important process that has been neglected in the management of patients with ACS.

4.2 Purpose of the analyses

To investigate the efficacy of repeated low doses of interleukin-2 (IL-2) in reducing vascular inflammation in ACS.

5 Study Objectives and Endpoints

5.1 Study Objectives

Primary objective

- To compare the effect of low dose IL-2 against placebo on vascular inflammation using 18F-FDG PET/CT in ACS patients.

Secondary objectives

- To determine if low dose IL-2 can increase Treg and alter Teff cell numbers over extended treatment
- To determine the safety and tolerability of extended dosing of low dose IL-2 in patients with an acute coronary syndrome

Exploratory objectives
- To determine the impact of low dose IL-2 on peripheral blood mononuclear cell subsets which may include (but not limited to) B cells, NK cells, Th1, Th2, Th17 and TFH cells.
- To determine the effect of low dose IL-2 on left ventricular systolic function.
- To determine the effect of low-dose IL-2 on systemic inflammation measured by cardiovascular biomarkers (including but not limited to hsCRP, IL-6, Troponin I).
- To determine the effect of low-dose IL-2 on the gut microbiome
- To determine the effect of low-dose IL-2 on coronary artery inflammation
- To compare the effect of low-dose IL-2 against placebo on 18F-FDG uptake in bone marrow in ACS patients

5.2 Endpoints

5.2.1 Primary endpoint:

Change in vascular inflammation (as measured by mean maximum tissue-to-blood ratio (mean TBR_{max}) in the index vessel) on ^{18}F-FDG PET/CT from baseline to follow up scans.

(In detail, a region of interest (ROI) including arterial wall and lumen will be drawn on each axial slice of artery (ascending aorta and both carotid arteries) on the co-registered PET/CT scan and the maximum standardised uptake value (SUVmax) recorded. Subsequently, each ROI will be normalised by the blood FDG concentration in the superior vena cava or jugular vein (for carotids), to yield an arterial mean maximum tissue-to-blood ratio (TBRmax) as a quantitative measure of arterial tracer uptake. The "index vessel" (defined as the arterial territory with the highest mean TBR_{max} at baseline - left carotid, right carotid or ascending aorta) will be the primary outcome variable.

All scans will be analysed by an experienced reader, anonymised to patient identifiable information (name, treatment group, and visit number.)

Per protocol, the study was powered to detect an absolute difference of 0.2 in mean TBRmax in the index vessel between placebo and active treatment at the end of the treatment period (2-sided 5% significance level, and 80% power). It is also noted that in several documents related to this study the terms "slice" and "segment" have been used interchangeably. We clarify that there is no distinction intended between these two terms.

5.2.2 Secondary endpoints:

1. Change in mean TBR_{max} in each arterial region individually restricted to slices designated as active at baseline.

However in this acute coronary syndrome population, the proportion of slices meeting this definition is anticipated to be too high, and near 100%, thus offering no level of stratification. Hence the analyses will consider a range of thresholds greater than 1.6 to consider how the treatment effects vary according to the choice of threshold. The preferred threshold for reporting the active slice analysis if the almost all slices are >1.6, is 2 as per previous publications [16,6,17], however values will be reported for a range of thresholds as above.

2. Change in lymphocyte subsets: T effector (Teffs) cells, defined as central memory and effector memory T cells in the non-Treg gated T cells will be evaluated by flow cytometry, Tfh, Th17, Th1, and Th2 cells.
3. Change in percentage of Treg cells (defined as CD3+CD4+CD25highCD127low cells within the CD3+CD4+ T cell gate) between low dose IL-2 and placebo throughout the treatment period will be evaluated by flow cytometry.
4. The safety and tolerability of extended dosing of IL-2 in ACS patients will be evaluated by:
   - Adverse events
   - Further cardiovascular events
   - Concomitant medications.
   - Physical examination defined as examination of the cardiovascular, respiratory, gastrointestinal, limited skin and brief neurological examinations
   - Examination of injection site reactions
   - Vital observations which include blood pressure, heart rate, temperature, respiratory rate and oxygen saturation
   - Safety clinical blood tests (defined in Protocol section 11.4)
   - Thyroid function blood test (defined in Protocol section 11.4)
   - 12-lead electrocardiogram (ECGs) recordings

5.2.3 Exploratory endpoints:

1. Change in serum cardiac biomarkers.

2. Change in ejection fraction as measured on transthoracic echocardiograms
3. Change in phenotype and function of peripheral blood mononuclear cell (PBMC) subsets (such as B lymphocytes and Natural Killer cells) as assessed by flow cytometry, gene expression, and in vitro activation and suppression assays.
4. Differences in gut microbiota composition between low-dose IL-2 vs placebo will be identified using 16S- RNAseq
5. The effect of low-dose IL-2 on coronary artery inflammation will be measured by perivascular fat attenuation using computed tomography coronary angiography
6. The effect of low-dose IL-2 on 18F-FDG uptake in thoracic vertebrae (bone marrow).

6 Study Methods

6.1 General Study Design and Plan

This is a randomised, double-blind, placebo controlled, parallel group experimental medicine trial. The aim of the trial is to test the superiority of low dose IL-2 compared to placebo in reducing vascular inflammation in ACS patients with hs-CRP>2mg/l.

In order to reduce heterogeneity in this small Phase 2 experimental medicine clinical trial, stratification at randomisation based on an ECG based ST-elevation status will be utilised to balance the groups. Patients will be randomised in a 1:1 fashion to either low dose interleukin-2 or placebo using an online randomisation system (Sealed Envelope).

The trial will be double-blind, with active and placebo doses appearing identical at point of issue and administration. The CUH central pharmacy will be unblinded and provided with a copy of the concealment list.

The study flow chart includes details of when participants are randomised to relevant treatments, events and study periods.

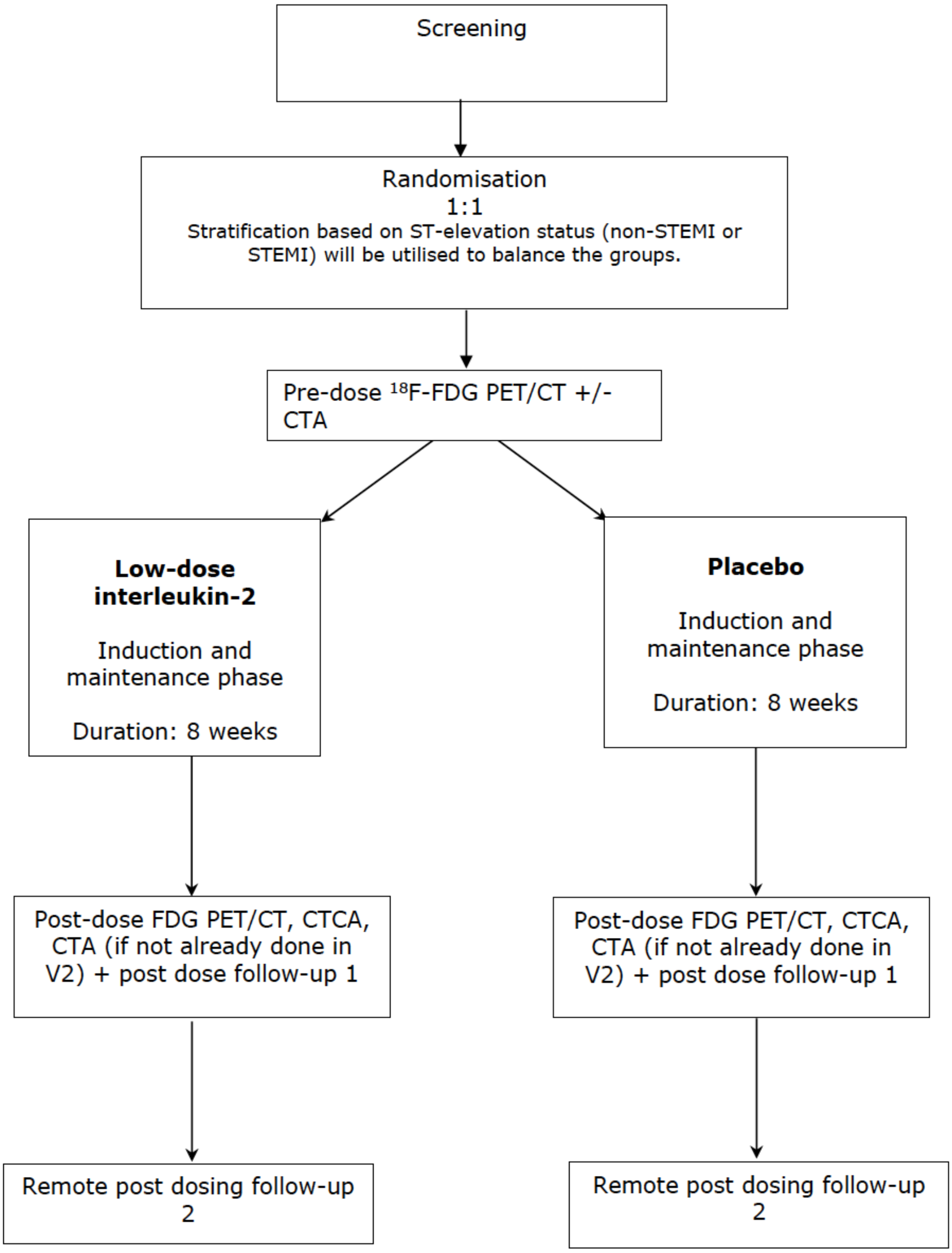

6.2 Inclusion–Exclusion Criteria and General Study Population

(ICH E3; 9.3. ICH E9; 2.2.1)

6.2.1 Inclusion Criteria

To be included in the trial the patient must meet the following criteria:

- Able to provide written informed consent to participate

- Aged between 18 and 85
- Current admission (on the screening visit) with an acute coronary syndrome - ST elevation myocardial infarction (STEMI), non-ST elevation myocardial infarction (NSTEMI), or unstable angina (UA) with symptoms suggestive of myocardial ischaemia lasting 10 minutes or longer with the patient at rest or with minimal effort

  AND EITHER

  i. elevated levels of TnI on admission

  OR

  ii. dynamic changes in ECG (new ST-T changes or T-wave inversion).
- Where applicable, to be included in the trial women must be

1) Postmenopausal (for the purposes of this trial, postmenopausal is defined as being amenorrhoeic for greater than 2 years with an appropriate clinical profile, e.g. age appropriate, history of vasomotor symptoms)

   OR

2) Have had a documented hysterectomy and/or bilateral oophorectomy or sterilised

   OR

3) Peri-menopausal with a negative pregnancy test at screening (for the purposes of inclusion in this trial. Peri-menopausal is defined as women with an appropriate clinical profile, e.g. age appropriate, history of vasomotor symptoms, irregular periods). They will also have to comply with the use of contraception for the duration of the trial and undergo additional pregnancy tests during and after treatment.

- High sensitivity C-reactive protein of >2 mg/L at screening
- Willingness and possibility to start dosing within 8 days from initial date of admission to the primary hospital for ACS
- Able to comply with all trial mandated visits.

6.2.2 Exclusion Criteria

The presence of any of the following will preclude patient inclusion:

- Current presentation (at screening) with cardiogenic shock (systolic blood pressure <80 mm Hg, unresponsive to fluids, or necessitating catecholamines).
- Current presentation with cardiac arrest

- Signs or symptoms of active infection requiring intravenous antibiotic treatment at screening
- History of malignancies requiring active treatment (However, patients with a history of treated localised basal or squamous cell skin cancer are not excluded from participation in this trial)
- History of solid organ transplantation or other bone marrow transplantation
- History of recurrent epileptic seizures in the previous 4 years; repetitive or difficult to control seizures, coma or toxic psychosis lasting >48 hours
- Uncontrolled hypotension (Systolic BP (SBP)<80mmHg or DBP<50mmHg) OR uncontrolled hypertension (SBP>180 or DBP>120 mmHg) at screening
- Average corrected QT interval (QTc) > 450 msecs using Bazett's formula from average of triplicate ECGs (or > 480 msecs if bundle branch block)
- Renal impairment defined as Creatinine clearance [Cockcroft-Gault] <45ml/min at screening
- Liver dysfunction (defined as ALT > 2xULN) at screening
- Evidence of cholestasis defined as elevated Total Bilirubin Levels, (TBL > 1.5 x ULN) and Alkaline Phosphatase, ALP (ALP > 1.5 x ULN), at screening
- Known hypothyroidism or hyperthyroidism
- Known autoimmune disease requiring active immunosuppressive treatment
- Any oral or intravenous immunosuppressive treatment including regular prednisolone, hydrocortisone or disease modifying drugs. [Inhaled or topical steroids are permissible]
- Patients on cytotoxic drugs and interferon-alpha
- Diabetics on oral hypoglycaemics/diet control with HbA1c (DCCT) > 8% (OR HbA1c (IFCC) > 64 mmol/mol), at screening. Diabetics on insulin are excluded from the study.
- Contraindication to IL-2 treatment or hypersensitivity to IL-2 or to any of its excipients
- Participation in a previous research trial in the last 3 years which involved exposure to significant ionising radiation (i.e. cumulative research radiation dose >5 mSv)
- Participation in a clinical trial where the patient has received a drug or new chemical entity within 30 days or 5 half-lives, or twice the duration of the biological effect of the drug (whichever is longer) prior to the first dose of trial medication, Visit 3 (Day 1).

- Any medical history or clinically relevant abnormality that is deemed by the principal investigator/delegate to make the patient ineligible for inclusion because of a safety concern
- Pregnant women or breast feeding women
- Patients who are COVID-19 PCR positive at the time of screening
- Known severe allergy to the CT-contrast agents.

6.3 Randomisation and Blinding

A sufficient number of patients will be randomised so that approximately 60 patients will complete the trial. Stratification at randomisation based on an ECG based ST-elevation status (to define non-STEMI or STEMI status) will be utilised to balance the groups. Patients will be randomised in a 1:1 fashion to either low dose interleukin-2 or placebo using an online randomisation system (Sealed Envelope).

The trial will be double-blind, with active and placebo doses appearing identical at point of issue and administration. The CUH central pharmacy will be unblinded and provided with a copy of the concealment list. A small team from the Cambridge Clinical Research Centre will also prepare maintenance doses in an unblinded manner but the investigator team (doctors and nurses) will remain blinded. Data analysis for the trial will be performed by a statistician who will be unblinded after the database lock.

The statistician, or delegate, may be unblinded for individual patients after their treatment period has concluded, to facilitate rapid reporting of safety events to the IDMC.

6.4 Study Variables

The table shows the schedule of assessments.

Visit no ->	1	2	3	4	5	6	7	8	9	10	11	12	13	14	15	16 (Remote where possible)
**Treatment Day**	**-14 to 0**	**-13 to 0**	**1 ^**	**2 ^**	**3 ^**	**4 ^**	**5 ^**	**12[a]**	**19[a]**	**26[a]**	**33[a]**	**40[a]**	**47[a]**	**54[a]**	**61[#]**	**82[a]**
Informed consent	x															
18F-FDG PET/CT Scan[b]		x													X[#]	
Demography[c]	x															
Height/weight	x															
Medical history	x															
Medication history	x															
Inclusion/exclusion	x	x	x													
Adverse events	x	x	x	x	x	x	x	x	x	x	x	x	x	x	x	x
Concomitant medications	x	x	x	x	x	x	x	x	x	x	x	x	x	x	x	x
Serum / urinary pregnancy test	x									x						X*
HbA1c	X															
Physical examination[d]	x		x	x	x	x	x	x	x	x	x	x	x	x	x	X*
Vital observations[e]	x		x	x	x	x	x	x	x	x	x	x	x	x	x	X*
ECG	x[f]		x	x	x	x	x	x	x	x	x	x	x	x	x	X*
ECHO	x[g]													x[h]	x[h]	
IMP administration			x	x	x	x	x	x	x	x	x	x	x	x		
Safety bloods[i]	x		x	X +	x	X +	x	x	x	x	x	x	x	x	x	X*
OPTIONAL Point of care bloods			x					x	x	x	x	x	x	x		
TSH	x														x	X*
Tregs and Lymphocytes			x				x	x		x		x		x	x	X*
PBMC assay			x				x	x		x		x		x	x	X*
Cardiac biomarkers[j]			x				x	x		x		x		x	x	X*
hsCRP	x															
Full lipid profile			X												x	
Stool sample (where feasible/possible)			X												X	
CTA															X[k]	
CTCA															X[l]	

^ These visits can be scheduled +/- 1 day.
[a] These visits can be scheduled +/- 3 days.
[b] PET/CT assessment to be preceded by a fingerprick blood glucose test.
[c] Including DOB, age, gender, race.
[d] Including cardiovascular, respiratory, gastrointestinal, neurological and skin examinations.
[e] Temperature, blood pressure, heart rate, respiratory rate and oxygen saturations.
[f] 12-lead ECG in triplicate with average QTcB.
[g] May be done anytime after screening prior to dosing (this can occur at an ad hoc visit). If a research ECHO cannot be performed, a clinical ECHO undertaken during the index admission can be used.
[h] May be done anytime between V14 and V15.
[i] Safety bloods (including but not limited to): haematology (FBC and differentials), clinical biochemistry (electrolytes, urea, creatinine), liver function (ALT, ALP, albumin, bilirubin). POC bloods may be done to aid dosing administration prior to review of formal bloods - as a minimum, review of Hb, sodium, potassium, creatinine should be sufficient.
[j] hsCRP, IL-6, Troponin I.
[k] May be done as separate visits. The CT carotid angiogram can be done in visit 2.
[l] Can be done as a separate visit.
* Assessments to be done physically at the hospital only if it is clinically indicated.
# PET/CT can be scheduled +/- 6 days.
+ Safety bloods will only be done if deemed clinically necessary.

7 Sample Size

Per protocol, sample size is based on an absolute difference of 0.2 in the primary endpoint (mean TBR_{max} in the index vessel) between placebo and active treatment at the end of the treatment period. This is equivalent to a 10% difference from a reference value of 2.02 and equivalent to the size effect observed after atheroprotective therapy[5,6,7]. Interventions that have reported less than 10% difference in mean TBR_{max} in the index vessel between active treatment and placebo at the end of the treatment period[8,9] failed to reduce CV outcomes[10-12].

Assuming a SD of 0.24[5], 24 patients per arm, testing at 2-sided 5% significance level, will provide 80% power. Therefore, a sample size of 30 completed patients per arm was selected to account for scans which may not be analysable, or poor scans due to patient habitus, movement and so on.

This sample size of n=30 per group also allows the detection of a baseline-corrected 6% difference in mean TBR_{max} in the index vessel (mean 0.125, SD 0.166)[5] between placebo and active treatment at the end of the treatment period, at 2-sided 5% significance level and 80% power.

8 General Considerations

8.1 Timing of Analyses

The total trial duration for each patient will be approximately 13 weeks. This will include 2 weeks for recruitment and screening, 8 weeks of treatment and a follow up period of approximately 4 weeks after the last treatment visit. The follow-up PET/CT and CTCA will be scheduled within 2 weeks after the last treatment visit.

The final analysis will occur once the planned assessments for all randomised participants are completed and the data has been cleaned.

8.2 Analysis Populations

8.2.1 Full Analysis Population

Will include all patients who met the inclusion/exclusion criteria, received the trial drug or placebo, completed the treatment course after randomisation and underwent the first and last PET/CT scans.

8.2.2 Randomised Population

Anyone allocated to any trial drug or placebo dose and met the inclusion/exclusion criteria

8.2.3 Safety population

Anyone who consented to taking part in the trial, met the inclusion/exclusion criteria and received any single/multiple doses of drug or placebo.

8.2.4 Non-safety population

This complex trial involved recruiting sick patients presenting ad hoc to the emergency door and organising complex tests including PET-CT at very short notice and an intricate setup for drug preparation in Pharmacy. For this reason, randomisation often was done very early prior to all baseline tests being successfully completed in order to prepare the drug in Pharmacy and to book PET-CT scans in advance. This meant that a number of eligible patients were randomised who were subsequently withdrawn after their eligibility changed during the screening period. The withdrawn population consist of those who were:

A) withdrawn prior to randomisation and dosing

B) withdrawn post randomisation and prior to dosing

C) withdrawn after dosing commenced

Patients withdrawn in subset (C) will be included in the safety population as defined in section 8.2.3.

Patients withdrawn in subsets (A) and (B) and who met the inclusion/exclusion criteria, define the Non-Safety population

8.3 Covariates and Subgroups

No covariates apart from the treatment group will be used by default in analyses.

8.3.1 Subgroup analysis

The following subgroups will be explored:

- ST-elevation (STEMI) status
- Baseline (screening) Inflammatory status based on hsCRP levels: 2-3, 3-10, >10 mg/L[13]
- Participants on high dose statins at follow-up scans Vs Participants not on high dose statins at follow-up scans.

8.4 Missing Data

We do not anticipate a high level of drop out after treatment initiation. Hence we will report the number of non-missing values for comparison to the relevant population size. Statistics will be calculated based on complete cases, which assumes there is no association between a value being unobserved, and the underlying value.

If there was 5% or more missing data for the primary endpoint, multiple imputation will be performed and the primary analysis run again as a sensitivity analysis.

9 Summary of Study Data

Summary statistics of endpoints broken down by treatment arm and visit where appropriate will be provided. Categorical variables will report percentages and counts (p% (x/n)); continuous variables will report, mean, median, SD, min and max. Exploratory figures in the form of stacked bar-charts and box-and-whisker plots for categorical and continuous variables respectively will be provided. If appropriate, spaghetti plots will be provided, to show the change in variables over time (visits), and colour coded by treatment.

9.1 Participant Disposition

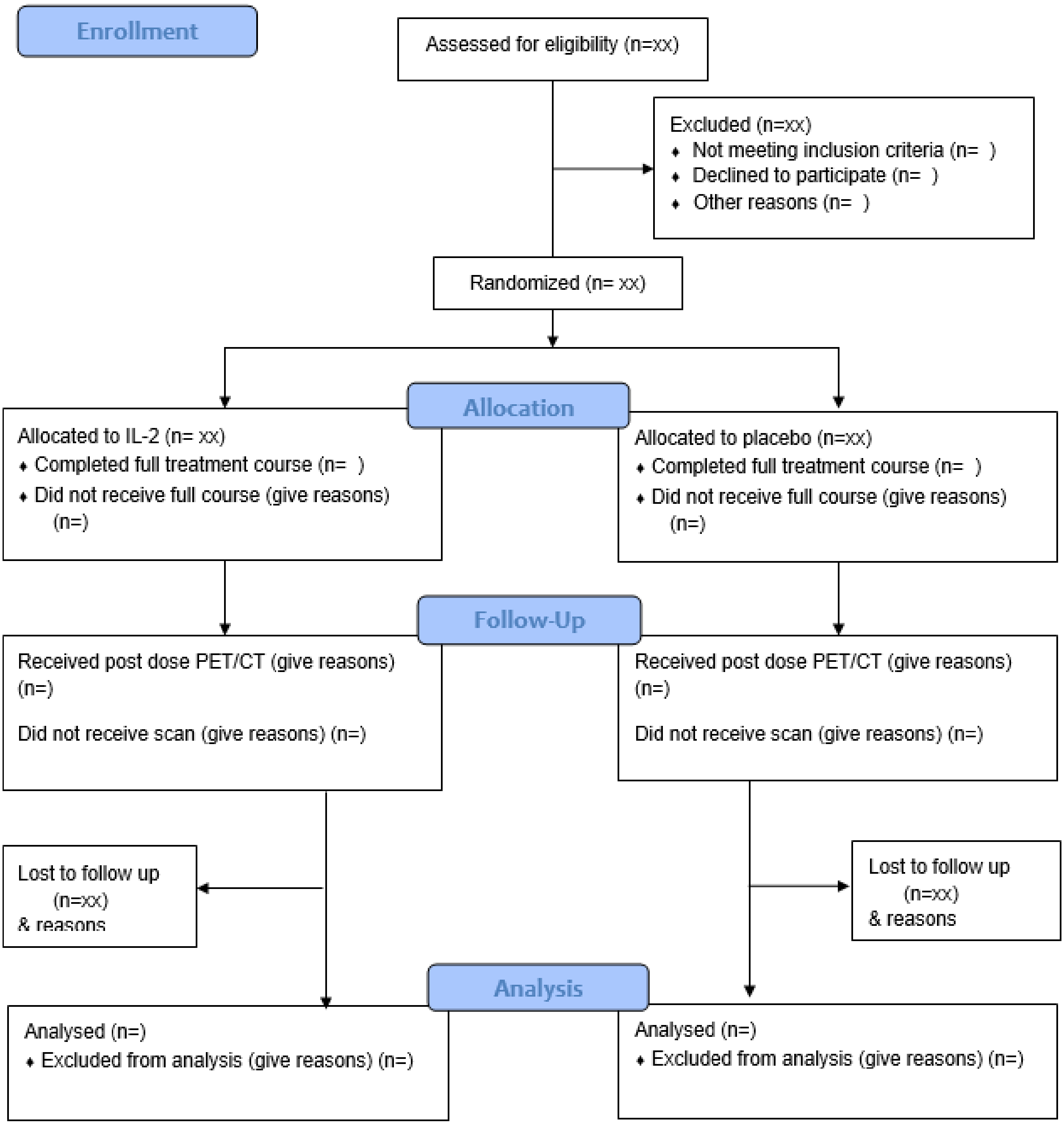

A table will present the count of participants in each population broken down by treatment arm, and also the count of PET/CT scans at baseline and V15.

9.2 Derived variables

9.2.1 Primary endpoint

^{18}F-FDG PET/CT Scans are performed at visit 2 (Baseline) and 15. For each scan the injection time and the injected activity are recorded in MBq, as well as the count at the start of the scan in the aorta and carotid arteries in Kcps will be recorded.

In the FDG PET-CT scans, for each cross-sectional slice of each vessel, mean and maximum SUV values will be calculated.

Scan quality: Scans are quality scored 1, 2 or 3, as assessed by a reader blinded to treatment. Scan regions (complete or single vessel scan) with quality score 3 (low quality) will be excluded from analysis.

Vessel: Data from slices in the following vessels will be analysed:

- LCC – Left common carotid
- RCC – Right common carotid
- IJV – Internal Jugular Vein
- SVC – superior vena cava
- BM– thoracic bone marrow
- ASA – ascending aorta

The acronym on the left is used in the scan analysis output to identify to which vessel a slice belongs.

The derived variables for at least 10% of the scans will be checked with a clinical member of the TMG prior to the analysis to ensure correct derivations.

9.2.1.1 Maximum tissue to blood ratio (TBRmax)

Maximum Tissue to Blood Ratio (Max TBR) will be calculated for each arterial slice and bone marrow within the thoracic vertebrae.

The equation used to calculate the TBRmax for each slice is as follows:

TBRmax = Max SUV (of each slice) / Average blood activity (mean SUV of the venous pool)

The different vessels of interest have different venous pool vessel corrections as per the table below

Vessel of Interest	Venous Pool Vessel
Aorta	Superior Vena Cava
Left common carotid	Internal Jugular Vein
Right common carotid	Internal Jugular Vein
Bone Marrow	Superior Vena Cava

9.2.1.2 Index vessel

For each participant at baseline the vessel with the highest mean TBR max value, will be termed the **"index vessel".**

9.2.1.3 Mean TBRmax

The Mean of the Max TBR will be calculated for the index vessel in each patient, for both scans

9.2.2 Active slice analysis

In the literature the definition of an active slice is when TBR max >1.6 . However in this acute coronary syndrome population, the proportion of slices meeting this definition is anticipated to be too high, and near 100%, thus offering no level of stratification. Hence the analyses will consider a range of thresholds greater than 1.6 to consider how the treatment effects vary according to the choice of threshold. The preferred threshold for reporting the active slice analysis if ~~the~~ almost all slices are >1.6, is 2 as per previous publications [16,6,17], however values will be reported for a range of thresholds as above.

9.2.2.1 Proportion of active slices

Proportion of vessel being active (in index vessel) = number of active slices in index vessel / total number of slices in index vessel

9.2.3 Slice Matching

Although conceptually simple to consider the same slice matched between the two visits, pre- and post-treatment, in reality ensuring that the observations made over the two visits really are capturing the same position in the aorta is not trivial to

achieve. The sets of slices were all reviewed, within the index vessel, and an expert judgement made to match the V2 and V15 slices so as to allow analysis of pairs of slices that were an exact match.

For some of the analyses the slices at V15 will only be included if they were classified as active at baseline (V2), regardless of their value at V15. In other analyses, the value observed at V15 will be used to classify the slice as active. The former case will be labelled as "Baseline-Active", to distinguish from the latter, which is labelled as "Active"

9.2.4 High Dose Statins

The subgroup listed in section 8.3.1is derived by search medication terms in the concomitant medication for any of the following terms

- statin
- ezitimibe

If there is also no stop date for the medication then the participant is included in the sub-group defined as "high dose statins".

9.2.5 CTCA, stool etc.

This exploratory work is planned and will be reported post hoc, therefore not in the scope of this statistical analysis plan.

9.3 Protocol Deviations

A listing of all major protocol deviation (eligibility, treatment error) will be provided. Additional information not recorded in the CRFs will be provided by the trial coordinators.

9.4 Demographic and Baseline Variables

The following demographic and baseline variables will be summarised according to Section 9.

9.4.1 Demographics

- Age
- Sex
- Weight
- Height
- Ethnicity
- hsCRP

9.4.2 Medical history

- Current diagnosis at admission
- Past medical history (IHD, hypertension, hypercholesterolemia, stroke/TIA, etc. according to medical history questionnaire CRF)
- Medication history (asprin, clopidogrel, ticagrelor, statin, etc. according to medical history questionnaire CRF)
- Smoking status
- GRACE score at admission

9.4.3 Intervention log details

- Whether the patient had an angiogram and what it showed
- The treatment plan
- Whether they are stented
- The type of intervention
- Diseased vessels

9.5 Concurrent Illnesses and Medical Conditions

MedDRA v25.1 was used for the adverse events, system organ class and preferred terms.

9.6 Treatment Compliance

The detailed treatment received will be summarised. A record of whether the subjects received all doses will be maintained with reasoning why doses had not been received.

10 Efficacy Analyses

The analysis will be based on the full analysis population.

10.1 Primary Efficacy Analysis

The primary efficacy analysis will be based on the full analysis population.

Vascular inflammation for the primary efficacy analysis will be analysed and reported in 3 ways using all slices of the index vessel. In part, this will follow a previous analysis from our group as published in Elkhawad et al[5] and others, whilst recognising this specific ACS population has not been studied in clinical trials utilising FDG PETCT previously.

The mean TBR_{max} for all slices in the index vessel, at baseline, post treatment PET/CT scans, and the change from baseline, will be summarised using mean ± SD and split by treatment group.

The primary efficacy analyses will include

a) A change from baseline in mean TBRmax in the index vessel in each treatment arm. The mean TBRmax in all slices in the index vessel (within each treatment group at pre-dose (v2) and post-dose (v15) will be analysed using a linear regression model. Point estimates and corresponding 95% confidence intervals (CI) and P-values will be reported for the change in mean TBRmax in each treatment arm.

   Example of model coding in R : V15 mean TBR max - V2 mean TBR max ~ Treatment

b) The difference in mean TBRmax at the end of treatment (the "on treatment" effect) between the treatment arms. This recognises that this is the basis that this study was powered for (IVORY protocol V4.216May2023 Section 16.3). Point estimates and corresponding 95% confidence intervals (CI) and P-values will be reported.

   Example of model coding in R : V15 mean TBR max ~ Treatment

c) The difference in change from baseline in mean TBRmax in all slices in the index vessel between the two treatment arms will also be calculated. The mean TBRmax in all slices in the index vessel (within each treatment group at pre-dose (v2) and post-dose (v15) will be analysed using a linear regression model with the mean TBRmax at v2 as a covariate.
   Example of model coding in R : Change from baseline mean TBRmax (V15-V2) ~ Mean TBRmax (v2) + Treatment

10.2Secondary Analyses

10.2.1 Baseline-Active Slice Analysis using mean TBRmax

The secondary efficacy imaging analyses will be based on baseline-active slices in the index vessel. The reference value of the threshold, 1.6, as quoted in the

protocol will be used alongside several other values. The preferred threshold for reporting the active slice analysis will be a threshold of 2 if the threshold of 1.6 captures almost 100% of the slices at baseline as this will provide values almost identical to the index vessel analysis involving all slices.

The mean TBRmax at V15 within each treatment group will be tracked for slices above a threshold at V2 (baseline-active) as per all previous publications involving active slice analysis.[6,9]

Index vessel active slice analysis using mean TBRmax of baseline-active slices

a) A change from baseline in mean TBRmax in the index vessel in each treatment arm for the baseline-active slices. Point estimates and corresponding 95% confidence intervals (CI) and P-values will be reported for the change in mean TBRmax in each treatment arm.
   Example of model coding in R : V15 mean TBR max in active slices identified in V2 - V2 mean TBR max in active slices ~ Treatment

b) The difference in mean TBRmax at the end of treatment (the "on treatment" effect) between the treatment arms will be presented will also be calculated for each threshold. Point estimates and corresponding 95% confidence intervals (CI) and P-values will be reported.
   Example of model coding in R : V15 mean TBR max in active slices identified in V2 ~ Treatment

c) The difference in change from baseline in mean TBRmax in baseline-active slices in the index vessel between the two treatment arms will also be calculated for each threshold. The mean TBRmax in active slices in the index vessel identified at V2 and tracked to post-dose (v15) will be analysed using a linear regression model with the mean TBRmax at v2 as a covariate. Point estimates and corresponding 95% confidence intervals (CI) and P-values will be reported.

   Example of model coding in R : Change from baseline (V15-V2) mean TBRmax in active slices identified in V2 ~ Mean TBRmax in active slices (v2) + Treatment

Sensitivity analyses with varying thresholds will be performed for analysis b) and c) above. A forest plot will be presented that depicts the baseline corrected change in mean TBR max per threshold and associated CI, with the p-values

10.2.2 Probability of a slice being active within the index vessel

This analysis will look at all slices within the index vessel and classify them as active or not, compared to a threshold. This is derived at V2 and V15 separately, and does not match slices between V2 and V15.

a) change from baseline in % of active slice in each treatment arm. Point estimates and corresponding 95% confidence intervals (CI) and P-values will be reported for the change in mean TBRmax in each treatment arm. A linear regression will be used and the absolute % difference estimated.

Example of model coding in R : % active slices in V15 – % of active slices in V2 ~ Treatment

The percentage of active slices at baseline and follow-up will be summarised using % ± SD and split by treatment group.

b) The difference % of active slices at the end of treatment (the "on treatment" effect) between the treatment arms for each threshold will be presented. Point estimates and corresponding 95% confidence intervals (CI) and P-values will be reported. A linear regression will be used and the absolute % difference estimated.

Example of model coding in R : % of active slices in V15 mean ~ Treatment

c) The number of active slices and the total number of slices at Visit 15 will be analysed using logistic regression to estimate the treatment effect comparing the probability of a slice being active. Baseline covariates to adjust for will be baseline

proportion of active slices. Generalised Estimating Equations, with an exchangeable working correlation matrix, will be used to adjust for multiple slices being observed within the same patient-vessel.

Example of model coding in R: Individual slice being active (v15) ~ Proportion of slice being active (v2) + Treatment

Odds ratios and corresponding 95% confidence intervals (CI) and P-values will be reported. A forest plot will be presented that depicts the odds ratios and associated CI, with the p-values.

Sensitivity analyses with varying thresholds will be performed for analysis b) and c) above.

10.2.3 Subgroup Analysis

Subgroup analysis on the endpoints above will be carried out, as defined in section 8.3. Interactions with the subgroup and treatment effect will be estimated, and summary tables broken down by treatment arm and subgroup provided.

10.2.4 Sensitivity Analyses:

The active slice analysis depends on the definition of an active slice, with a TBR max above a threshold. This is the first FDG PET-CT interventional drug study to be conducted in acutely presenting cardiovascular patients where the values may be out of range to previously studied groups. This is clearly demonstrated in a previous publication[8]. Therefore, we will consider sensitivity analyses using a range of different thresholds. The treatment effect estimates and confidence intervals at a sequence of 12 thresholds spaced evenly from 1.6,1.7,...,2.7 will be calculated, and presented in a figure.

10.2.4.1 Multiple Testing for Sensitivity Analyses

In the sensitivity analysis where the threshold used to define an active slice is varied, to control the false discovery rate to 5% or less the Benjamini-Hochberg process will be used, whereby

1. The m p-values are put into ascending order: $p\_1 < p\_2 < ... < p\_m$.
2. The largest value k is identified where $p\_k < k/m$ 5%.
3. Any null hypotheses p_1, p_2, ..., p_k are rejected.

This is equivalent to calculating adjusted p-values and directly comparing to 5%, or saying that a test would be accepted as family-wise significant if the family-wise significance level exceeds the adjusted p-value. The adjustment is calculated:

1. The *m* p-values are put into ascending order: $p_1 < p_2 < ... < p_m$.
2. The adjusted p-values $p^*_k = \min_{i \geq k} p_i . m/i$

The adjusted and unadjusted p-values will be presented as outputs. The estimated parameters from the models at thresholds of 1.6 and 2 will be presented in full, in addition to the forest plot of just the treatment effect estimate across multiple thresholds.

10.3Other analyses using the index vessel

10.3.1 The distribution of all slices from the index vessels

The group distribution of individual slices' TBRmax will be presented using a frequency histogram, for pre- and post-treatment for each treatment arm in order to visualise the distribution of individual datapoints. The Kolmogorov-Smirnov statistic will be applied to measure the effect of treatment on TBRmax distribution. Bootstrapping at the participant level will be used to calculate p-values, to account for the dependency between slides within a participant.

10.4All-vessel analysis

An analysis of the change from baseline in mean TBR max within all of the 3 vessels will be provided. A generalised least square model will be used to allow for within-participant correlation, and heteroscedasticity across the vessels, and fixed effects for the three baseline values for the three vessels and treatment. An interaction between vessel and treatment will be consider to check the assumption of a constant treatment effect.

10.5 Lymphocyte Subsets

The following variables are captured at multiple visits over time

- CD3 %
- CD3 absolute
- CD4 %
- CD4 absolute

- CD8 %
- CD8 absolute
- Treg %
- Treg absolute
- Th1 %
- Th1 absolute
- Th2 %
- Th2 absolute
- Th17 %
- Th 17 absolute
- Tfh %
- Tfh absolute
- Teff total %
- Teff total absolute
- Teff memory %
- Teff memory absolute
- Teff central memory %
- Teff central memory absolute
- Non-Treg %
- Non-Treg absolute

Spaghetti plots over time and split by treatment group will be presented. At each time point, the values will be summarised using mean values and corresponding confidence intervals, and split by treatment group. The mean values will be plotted with time on the x-axis and endpoint scale on the y-axis.

For Tregs (% and absolute) a mixed model repeated measures analysis with an unstructured covariance matrix for the within – patient residual errors will be fitted between visits 3 and visits 15. The model will specify fixed effects of treatment, baseline value, timing of assessment, and an interaction between treatment allocation and timing. A second model will be fitted that only considers the treatment period up to visit 15 and does not have an interaction between treatment and time: this estimates the treatment effects averaged over the treatment period.

Scatter plots of the Area Under the Curve of each of the variables above, vs the change-from-baseline in TBRmax, or achieved TBR max at V15, in the index vessel will be provided.

10.6Exploratory Efficacy Analyses

The exploratory endpoints 3, 4, 5, 6 in section 5.2.3 are not available at the time of writing and may be analysed later, outside the scope of this SAP.

10.6.1 Cardiac Biomarkers

Troponin spaghetti plots over time will be presented as well as a mixed model for repeated measures analysis, as described in section 10.5

The cardiac biomarkers apart from Troponin will be available after hard lock along with the data below. This analysis is planned but not in the scope of this report.

10.6.2 Ejection Fraction

For the change in ejection fraction, summaries for both visits will be given with box plots to show the distribution. A t-test will be performed comparing the change from pre- and post-treatment, and the post-treatment directly, across treatment groups.

10.6.3 Full Lipid Profile

This will be summarised as a spaghetti plot split by treatment group, and by the subgroups defined in section 8.3.1

- Total cholesterol
- LDL cholesterol
- Triglycerides

11 Safety Analyses

The safety analyses will be based on the safety population.

This will exclude a small number of participants who consented but did not receive any drug: the Non-Safety population. Any adverse events for these participants will be listed separately.

11.1 Adverse Events

- Listings of AEs without ISRs
- AE grouping by preferred term and split by treatment group
- Listing of cardiovascular events

11.2 Deaths, Serious Adverse Events and other Significant Adverse Events

- Incidence and listing of SAEs

11.3 Pregnancies

A listing broken down by treatment group will be provided.

11.4 Clinical Laboratory Evaluations

11.4.1 Safety bloods

Spaghetti plots split by treatment group will be provided for the following safety get

   a. TWBC
   b. Haemoglobin
   c. Platelets
- White blood cell count differential
   a. Neutrophils
   b. Lymphocytes
   c. Monocytes
   d. Eosinophils
   e. Basophils
- Electrolytes
- Urea
- Creatinine
- ALT
- ALP
- Albumin
- Bilirubin

11.4.2 Vital signs

Spaghetti plots split by treatment group will be provided for the following vital signs:

- Temperature
- Systolic blood pressure
- Diastolic blood pressure
- Heart rate
- Respiratory rate
- Oxygen saturation

11.5 Other Safety Measures

11.5.1 Physical examination

Any abnormal results will be reported and summaries of the physical examination will be given. This includes results from the cardiovascular, respiratory, gastrointestinal, neurological and skin examinations. The results will be split by treatment group.

11.5.2 Concomitant medications

- Listing of concomitant medications

- Concomitant medications grouped by medication name and split by treatment group

11.5.3 12-lead electrocardiogram (ECGs) recordings

The results that will be summarised by treatment group include:

- Any abnormalities of results
- Average QTcB

12 Figures

The following figures will be included:

- CONSORT diagram
- Spaghetti plots of bloods / cell counts / vital signs over time and split by treatment group
- PET/CT data to be presented as[5]:
  - Frequency histograms of all slices from index vessel
  - Forest plots of treatment comparison of mean TBRmax and probability of slice being active for a number of thresholds (both adjusted and non-adjusted p-values will be presented for each threshold)

- T cell subset data to be presented as[14]:
  - Scatter plots of change in percentage over time, with bars representing the standard error or IQR

13 Reporting Conventions

P-values ≥0.001 will be reported to 3 decimal places; p-values less than 0.001 will be reported as "<0.001". The mean, standard deviation, and any other statistics other than quantiles, will be reported to one decimal place greater than the original data. Quantiles, such as median, or minimum and maximum will use the same number of decimal places as the original data. Estimated parameters, not on the same scale as raw observations (e.g. regression coefficients) will be reported to 3 significant figures.

14 Technical Details

The SAP is based on Version 4.2 (16 May 2023) of the protocol. The software package R version 4.1.3 is in use at the time of writing, but if upgraded, this will be documented. Copies of the code written will be stored. Each report and individual table of graph will have:

- The date and time included
- The name of the code file that produced the analysis
- The author
- A log capturing the version of the software and any external add on code used.
- Population used

The version control system Git will be used and individual code files will also have comments that convey:

- the author
- the date and time of writing
- description of any revisions
- references to inputs and outputs
- reference to any parent code file that runs the child code file

A reviewing statistician will independently reproduce the following:

Primary outcome summary statistics

15 Summary of Changes to the Protocol or from Previous Version of the SAP

This is to be provided by trial coordinators.

Version No.	History	Date
v1.0	Final Protocol	08 May 2019
v1.1	Incorporating REC/HRA comments	15 Jul 2019
v2.0	Incorporation MHRA feedback on initial submission	Aug 2019
V3.0	Changes to endpoints, visit schedule	01 March 2021
V4.0	Changes to exploratory endpoints, changes to angiograms, addition of optional stool sample	08Jun2022
V4.1	Clarification of basis on which sample size calculation was made, correction of typographical errors for pre-bloods in induction phase of dosing	01Nov2022
V4.2	Deletion to sentences with transcription errors, addition of a reference to secondary endpoint 1, correction of contact details of investigators	16May2023

15.1 Changes from SAP V1

- Clarification to the population definitions to be explicit that only eligible participants are considered, and screening failures are excluded.
- Detailed description of primary and secondary efficacy analyses and re-structuring to align with protocol
- Detailed description of active slice analysis as per previous publications
- Clarification of the thresholds to be considered are 1.6,1.7,...,2.7.
- Clarification that the preferred threshold for reporting the active slice analysis will be a threshold of 2 if the threshold of 1.6 captures almost 100% of the slices at baseline.
- Use of GEE methods in the logistic regressions to account for multiple slices observed within the same patient-vessel.
- Scatter plot added for AUC of lymphocyte subsets vs change-from-baseline in TBR max and vs achieved TBR max at V15.
- Additional slice-matched analysis using generalised least squares.
- Primary and secondary efficacy analyses have been re-structured to align with protocol
- Clarification that STEMI status will not be used as a covariate. STEMI was used as a stratification factor a randomisation as it is known to influence important clinical endpoints, including ejection fraction, mortality, but has no predictive value for vascular inflammation.
- Clarification regarding sensitivity analysis and multiple testing with respect to the active slice analysis.

16 References

1 Libby, P. Mechanisms of acute coronary syndromes and their implications for therapy. *The New England journal of medicine* **368**, 2004-2013, doi:10.1056/NEJMra1216063 (2013).

2 Arbab-Zadeh, A., Nakano, M., Virmani, R. & Fuster, V. Acute coronary events. *Circulation* **125**, 1147-1156, doi:10.1161/circulationaha.111.047431 (2012).

3 Mega, J. L. *et al.* Rivaroxaban in patients with a recent acute coronary syndrome. *The New England journal of medicine* **366**, 9-19, doi:10.1056/NEJMoa1112277 (2012).

4 Wiviott, S. D. *et al.* Prasugrel versus clopidogrel in patients with acute coronary syndromes. *The New England journal of medicine* **357**, 2001-2015, doi:10.1056/NEJMoa0706482 (2007).

5 Elkhawad, M. et al. Effects of p38 mitogen-activated protein kinase inhibition on vascular and systemic inflammation in patients with atherosclerosis. JACC. Cardiovascular imaging 5, 911-922, doi:10.1016/j.jcmg.2012.02.016 (2012).

6 Maki-Petaja, K. M. et al. Anti-tumor necrosis factor-alpha therapy reduces aortic inflammation and stiffness in patients with rheumatoid arthritis. Circulation 126, 2473-2480, doi:10.1161/circulationaha.112.120410 (2012).

7 Tahara, N. et al. Simvastatin attenuates plaque inflammation: evaluation by fluorodeoxyglucose positron emission tomography. Journal of the American College of Cardiology 48, 1825-1831, doi:10.1016/j.jacc.2006.03.069 (2006).

8 Fayad, Z. A. et al. Safety and efficacy of dalcetrapib on atherosclerotic disease using novel non-invasive multimodality imaging (dal-PLAQUE): a randomised clinical trial. Lancet (London, England) 378, 1547-1559, doi:10.1016/s0140-6736(11)61383-4 (2011).

9 Tawakol, A. et al. Effect of treatment for 12 weeks with rilapladib, a lipoproteinassociated phospholipase A2 inhibitor, on arterial inflammation as assessed with 18F-fluorodeoxyglucose-positron emission tomography imaging. Journal of the American College of Cardiology 63, 86-88, doi:10.1016/j.jacc.2013.07.050 (2014).

10 O'Donoghue, M. L., Braunwald, E., White, H. D. & et al. Effect of darapladib on major coronary events after an acute coronary syndrome: The solid-timi 52 randomized clinical trial. JAMA 312, 1006-1015, doi:10.1001/jama.2014.11061 (2014).

11 Schwartz, G. G. et al. Effects of dalcetrapib in patients with a recent acute coronary syndrome. The New England journal of medicine 367, 2089-2099, doi:10.1056/NEJMoa1206797 (2012).

12 White, H. D. et al. Darapladib for preventing ischemic events in stable coronary heart disease. The New England journal of medicine 370, 1702-1711, doi:10.1056/NEJMoa1315878 (2014).

13 Ridker, P.M. (2003) "C-reactive protein," *Circulation*, 108(12). Available at: https://doi.org/10.1161/01.cir.0000093381.57779.67.

14 He, Jing, et al. Low-dose interleukin-2 treatment selectively modulates CD4+ T cell subsets in patients with systemic lupus erythematosus. Nature medicine 22.9 (2016): 991-993.

15 Praestgaard, Jens Thomas. "Permutation and bootstrap Kolmogorov-Smirnov tests for the equality of two distributions." Scandinavian Journal of Statistics (1995): 305-322.

16 WU, Y. W., KAO, H. L., CHEN, M. F., LEE, B. C., TSENG, W. Y., JENG, J. S., TZEN, K. Y., YEN, R. F., HUANG, P. J. & YANG, W. S. (2007) Characterization of plaques using 18F-FDG PET/CT in patients with carotid atherosclerosis and correlation with matrix metalloproteinase-1. J Nucl Med, 48, 227-33

17 TAWAKOL, A., MIGRINO, R. Q., BASHIAN, G. G., BEDRI, S., VERMYLEN, D., CURY, R. C., YATES, D., LAMURAGLIA, G. M., FURIE, K., HOUSER, S., GEWIRTZ, H., MULLER, J. E., BRADY, T. J. & FISCHMAN, A. J. (2006) In vivo 18F-fluorodeoxyglucose positron emission tomography imaging provides a noninvasive measure of carotid plaque inflammation in patients. J Am.Coll.Cardiol., 48, 1818-1824,

CCTU/TPL007 V5 Approved: 27/09/2021 Reviewed: 27/09/2021

Statistical Analysis Plan

TRIAL FULL TITLE	Low-dose interleukin-2 for the reduction of vascular inflammation in Acute Coronary Syndromes (IVORY)
IRAS ID	220945
SAP VERSION	1.0
ISRCTN NUMBER	
SAP VERSION DATE	27JUL2023
TRIAL STATISTICIAN	Simon Bond
TRIAL CHIEF INVESTIGATOR	Dr Joseph Cheriyan
SAP AUTHOR	Simran Vaja , Simon Bond

1 SAP Signatures

I give my approval for the attached SAP entitled IVORY dated 27JUL2023

Chief Investigator

Name: Dr Joseph Cheriyan

Signature: ______________________________

Date: ______________________________

Statistician

Name: Simon Bond

Signature: ______________________________

Date: ______________________________

2 Table of Contents

1 SAP Signatures 1
2 Table of Contents 2
3 Abbreviations and Definitions 4
4 Introduction 6
4.1 Preface 6
4.2 Purpose of the analyses 6
5 Study Objectives and Endpoints 6
5.1 Study Objectives 6
5.2 Endpoints 7
5.2.1 Primary endpoint: 7
5.2.2 Secondary endpoints: 7
5.2.3 Exploratory endpoints: 8
6 Study Methods 9
6.1 General Study Design and Plan 9
6.2 Inclusion-Exclusion Criteria and General Study Population 10
6.2.1 Inclusion Criteria 10
6.2.2 Exclusion Criteria 11
6.3 Randomisation and Blinding 13
6.4 Study Variables 13
7 Sample Size 15
8 General Considerations 16
8.1 Timing of Analyses 16
8.2 Analysis Populations 16
8.2.1 Full Analysis Population 16
8.2.2 Randomised Population 16
8.2.3 Safety population 16
8.2.4 Non-safety population 16

8.3 Covariates and Subgroups 17
8.3.1 Subgroup analysis 17
8.4 Missing Data 17
8.5 Multiple Testing 17
9 Summary of Study Data 18
9.1 Participant Disposition 19
9.2 Derived variables 20
9.2.1 Primary endpoint 20
9.2.2 High Dose Statins 21
9.2.3 CTCA, stool etc. 21
9.3 Protocol Deviations 22
9.4 Demographic and Baseline Variables 22
9.4.1 Demographics 22
9.4.2 Medical history 22
9.4.3 Intervention log details 22
9.5 Concurrent Illnesses and Medical Conditions 22
9.6 Treatment Compliance 22
10 Efficacy Analyses 23
10.1 Primary Efficacy Analysis 23
10.1.1 Sensitivity Analyses: 25
10.1.2 Subgroup Analysis 25
10.2 Secondary Efficacy Analyses 25
10.2.1 Vascular Inflammation 25
10.2.2 Lymphocyte Subsets 25
10.3 Exploratory Efficacy Analyses 26
10.3.1 Cardiac Biomarkers 26
10.3.2 Ejection Fraction 27
10.3.3 Bone Marrow 27

10.3.4 Full Lipid Profile 27
11 Safety Analyses 27
11.1 Adverse Events 27
11.2 Deaths, Serious Adverse Events and other Significant Adverse Events 27
11.3 Pregnancies 28
11.4 Clinical Laboratory Evaluations 28
11.4.1 Safety bloods 28
11.4.2 Vital signs 28
11.5 Other Safety Measures 28
11.5.1 Physical examination 29
11.5.2 Concomitant medications 29
11.5.3 12-lead electrocardiogram (ECGs) recordings 29
12 Figures 29
13 Reporting Conventions 29
14 Technical Details 30
15 Summary of Changes to the Protocol or from Previous Version of the SAP 30
16 References 31

3 Abbreviations and Definitions

AE/AR	Adverse event/Adverse Reaction
ACS	Acute Coronary Syndrome
ALP	Alkaline phosphatase
ALT	Alanine aminotransferase
AST	Aspartate aminotransferase
BNP	B-type Natriuretic Peptide
CA	Competent Authority
CABG	Coronary artery bypass graft
CCTU	Cambridge Clinical Trials Unit
CI	Chief investigator
CNS	Central nervous system
CRF	Case Report Form

CT	Computed Tomography
CV	cardiovascular
DMC	Data Monitoring Committee
DSUR	Development Safety Update Report
ECG	Electrocardiogram
FDG - PET/CT	Fluorodeoxyglucose - Positron emission tomography/ computed tomography
GCP	Good Clinical Practice
GP	General Practitioner
hs-CRP	High-Sensitivity C-Reactive Protein
ICF	Informed Consent Form
IHD	Ischaemic heart disease
IMP	Investigational Medicinal Product
ISR	Injection site reaction
IU	International Unit
MHRA	Medicines and Healthcare products Regulatory Agency
NIMP	Non Investigational Medicinal Product
NSTEMI	Non-ST Elevation Myocardial Infarction
PBMC	Peripheral Blood Mononuclear Cell assay
PCI	Percutaneous coronary intervention
PET	Positron Emission Tomography
PIS	Patient Information Sheet
QTcB	Corrected QT using Bazett's formula
R&D	Research and Development
RA	Regulatory Agency
REC	Research Ethics Committee
RSI	Reference Safety Information
SAE/SAR	Serious Adverse Event/Serious Adverse Reaction
SmPC	Summary of Product Characteristics
STEMI	ST elevation myocardial infarction
ST	ECG parameter
SUSAR	Suspected Unexpected Serious Adverse Reaction
SUV	Standardized Uptake Value
TBR	Tissue-to-blood ratio
TMG	Trial Management Group
TnI	Troponin I
Treg	Regulatory T cells
TSH	Thyroid Stimulating Hormone
TTE	Transthoracic echocardiogram
ULN	Upper Limit of Normal

4 Introduction

4.1 Preface

Acute coronary syndromes (ACS) result from coronary plaque(s) disruption, which initiates a thrombotic process leading to partial or complete obstruction of the vessel lumen with subsequent myocardial ischaemia and necrosis[1,2]. The mainstay of treatment is currently focussed on the re-establishment and maintenance of coronary artery patency using anti-platelets and anticoagulants with or without mechanical dilatation and stenting of the culprit artery[1,2]. Despite important advances in management, ACS still carries a risk of substantial morbidity and mortality[1]. The improved efficacy of novel anti-platelet and anticoagulant agents have been limited by increased risk of haemorrhagic events[3,4]. Thus, future breakthroughs in management are most likely to arise from targeting other relevant pathophysiological pathways. Particularly, we believe that the immune response is an important process that has been neglected in the management of patients with ACS.

4.2 Purpose of the analyses

To investigate the efficacy of repeated low doses of interleukin-2 (IL-2) in reducing vascular inflammation in ACS.

5 Study Objectives and Endpoints

5.1 Study Objectives

Primary objective

- To compare the effect of low dose IL-2 against placebo on vascular inflammation using 18F-FDG PET/CT in ACS patients.

Secondary objectives

- To determine if low dose IL-2 can increase Treg and alter Teff cell numbers over extended treatment
- To determine the safety and tolerability of extended dosing of low dose IL-2 in patients with an acute coronary syndrome

Exploratory objectives

- To determine the impact of low dose IL-2 on peripheral blood mononuclear cell subsets which may include (but not limited to) B cells, NK cells, Th1, Th2, Th17 and TFH cells.
- To determine the effect of low dose IL-2 on left ventricular systolic function.
- To determine the effect of low-dose IL-2 on systemic inflammation measured by cardiovascular biomarkers (including but not limited to hsCRP, IL-6, Troponin I).
- To determine the effect of low-dose IL-2 on the gut microbiome
- To determine the effect of low-dose IL-2 on coronary artery inflammation
- To compare the effect of low-dose IL-2 against placebo on 18F-FDG uptake in bone marrow in ACS patients

5.2 Endpoints

5.2.1 Primary endpoint:

Change in vascular inflammation (as measured by mean maximum tissue-to-blood ratio (mean TBR_{max}) in the index vessel) on ^{18}F-FDG PET/CT from baseline to follow up scans.

(In detail, a region of interest (ROI) including arterial wall and lumen will be drawn on each axial slice of artery (ascending aorta and both carotid arteries) on the co-registered PET/CT scan and the maximum standardised uptake value (SUVmax) recorded. Subsequently, each ROI will be normalised by the blood FDG concentration in the superior vena cava or jugular vein (for carotids), to yield an arterial mean maximum tissue-to-blood ratio (TBRmax) as a quantitative measure of arterial tracer uptake. The "index vessel" (defined as the arterial territory with the highest mean TBR_{max} at baseline - left carotid, right carotid or ascending aorta) will be the primary outcome variable.

All scans will be analysed by an experienced reader, anonymised to patient identifiable information (name, treatment group, and visit number.)

We note that in several documents related to this study the terms "slice" and "segment" have been used interchangeably. We clarify that there is no distinction intended between these two terms.

5.2.2 Secondary endpoints:

1. Change in mean TBR_{max} in each arterial region individually restricted to slices designated as active. Sensitivity analyses will vary the threshold used to define an active slice, incorporating the reference value of 1.6.
2. Change in lymphocyte subsets: T effector (Teffs) cells, defined as central memory and effector memory T cells in the non-Treg gated T cells will be evaluated by flow cytometry, Tfh, Th17, Th1, and Th2 cells.

3. Change in percentage of Treg cells (defined as CD3+CD4+CD25highCD127low cells within the CD3+CD4+ T cell gate) between low dose IL-2 and placebo throughout the treatment period will be evaluated by flow cytometry.
4. The safety and tolerability of extended dosing of IL-2 in ACS patients will be evaluated by:
   - Adverse events
   - Further cardiovascular events
   - Concomitant medications.
   - Physical examination defined as examination of the cardiovascular, respiratory, gastrointestinal, limited skin and brief neurological examinations
   - Examination of injection site reactions
   - Vital observations which include blood pressure, heart rate, temperature, respiratory rate and oxygen saturation
   - Safety clinical blood tests (defined in Protocol section 11.4)
   - Thyroid function blood test (defined in Protocol section 11.4)
   - 12-lead electrocardiogram (ECGs) recordings

5.2.3 Exploratory endpoints:

1. Change in serum cardiac biomarkers.
2. Change in ejection fraction as measured on transthoracic echocardiograms
3. Change in phenotype and function of peripheral blood mononuclear cell (PBMC) subsets (such as B lymphocytes and Natural Killer cells) as assessed by flow cytometry, gene expression, and in vitro activation and suppression assays.
4. Differences in gut microbiota composition between low-dose IL-2 vs placebo will be identified using 16S- RNAseq
5. The effect of low-dose IL-2 on coronary artery inflammation will be measured by perivascular fat attenuation using computed tomography coronary angiography
6. The effect of low-dose IL-2 on 18F-FDG uptake in thoracic vertebrae (bone marrow).

6 Study Methods

6.1 General Study Design and Plan

This is a randomised, double-blind, placebo controlled, parallel group experimental medicine trial. The aim of the trial is to test the superiority of low dose IL-2 compared to placebo in reducing vascular inflammation in ACS patients with hs-CRP>2mg/l.

Stratification at randomisation based on an ECG based ST-elevation status be utilised to balance the groups. Patients will be randomised in a 1:1 fashion to either low dose interleukin-2 or placebo using an online randomisation system (Sealed Envelope).

The trial will be double-blind, with active and placebo doses appearing identical at point of issue and administration. The CUH central pharmacy will be unblinded and provided with a copy of the concealment list.

The study flow chart includes details of when participants are randomised to relevant treatments, events and study periods.

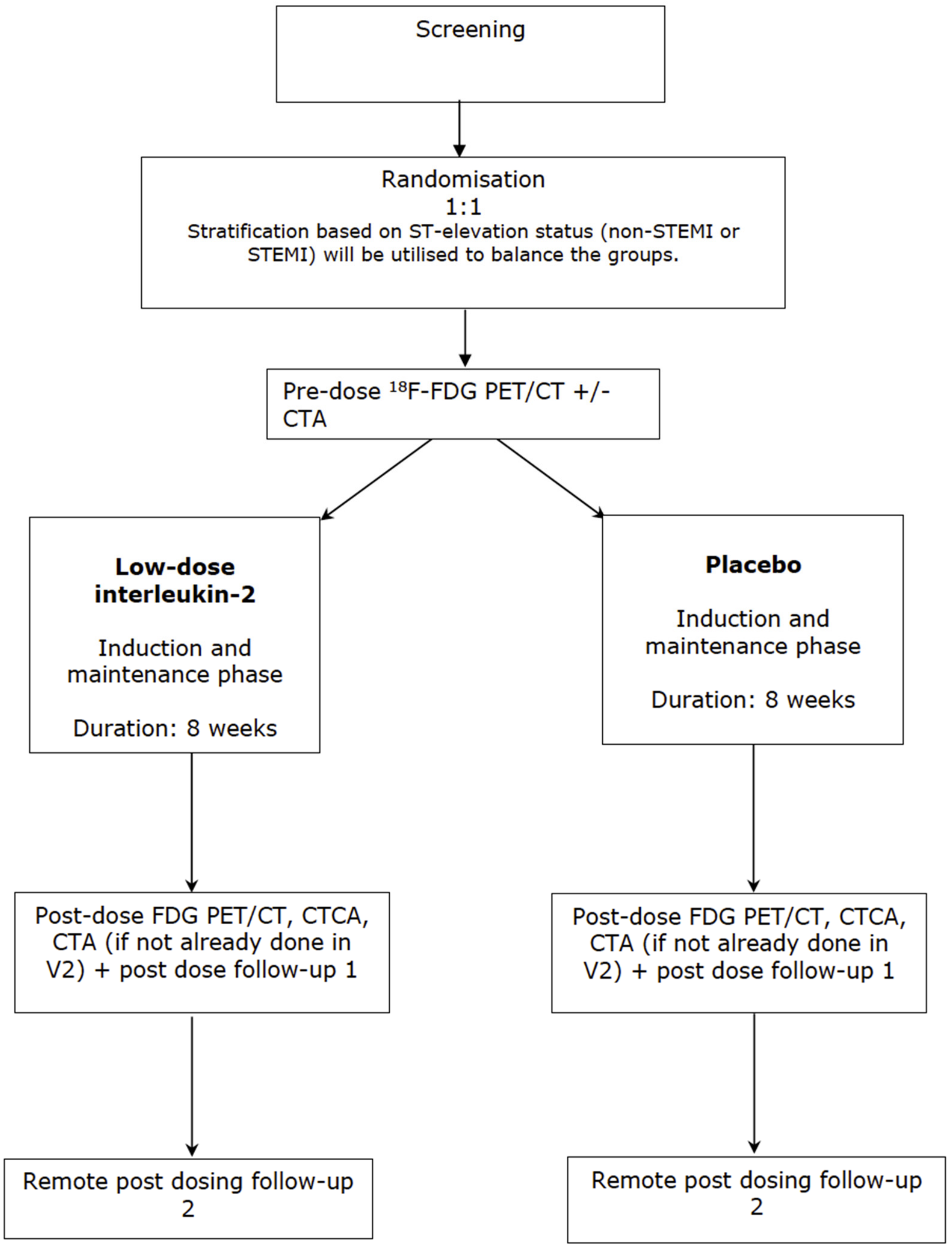

6.2 Inclusion–Exclusion Criteria and General Study Population

(ICH E3; 9.3. ICH E9; 2.2.1)

6.2.1 Inclusion Criteria

To be included in the trial the patient must meet the following criteria:

- Able to provide written informed consent to participate

- Aged between 18 and 85
- Current admission (on the screening visit) with an acute coronary syndrome – ST elevation myocardial infarction (STEMI), non-ST elevation myocardial infarction (NSTEMI), or unstable angina (UA) with symptoms suggestive of myocardial ischaemia lasting 10 minutes or longer with the patient at rest or with minimal effort

  AND EITHER

  i. elevated levels of TnI on admission

  OR

  ii. dynamic changes in ECG (new ST-T changes or T-wave inversion).
- Where applicable, to be included in the trial women must be

1) Postmenopausal (for the purposes of this trial, postmenopausal is defined as being amenorrhoeic for greater than 2 years with an appropriate clinical profile, e.g. age appropriate, history of vasomotor symptoms)

   1. OR

2) Have had a documented hysterectomy and/or bilateral oophorectomy or sterilised

   1. OR

3) Peri-menopausal with a negative pregnancy test at screening (for the purposes of inclusion in this trial. Peri-menopausal is defined as women with an appropriate clinical profile, e.g. age appropriate, history of vasomotor symptoms, irregular periods). They will also have to comply with the use of contraception for the duration of the trial and undergo additional pregnancy tests during and after treatment.

- High sensitivity C-reactive protein of >2 mg/L at screening
- Willingness and possibility to start dosing within 8 days from initial date of admission to the primary hospital for ACS
- Able to comply with all trial mandated visits.

6.2.2 Exclusion Criteria

The presence of any of the following will preclude patient inclusion:

- Current presentation (at screening) with cardiogenic shock (systolic blood pressure <80 mm Hg, unresponsive to fluids, or necessitating catecholamines).
- Current presentation with cardiac arrest

- Signs or symptoms of active infection requiring intravenous antibiotic treatment at screening
- History of malignancies requiring active treatment (However, patients with a history of treated localised basal or squamous cell skin cancer are not excluded from participation in this trial)
- History of solid organ transplantation or other bone marrow transplantation
- History of recurrent epileptic seizures in the previous 4 years; repetitive or difficult to control seizures, coma or toxic psychosis lasting >48 hours
- Uncontrolled hypotension (Systolic BP (SBP)<80mmHg or DBP<50mmHg) OR uncontrolled hypertension (SBP>180 or DBP>120 mmHg) at screening
- Average corrected QT interval (QTc) > 450 msecs using Bazett's formula from average of triplicate ECGs (or > 480 msecs if bundle branch block)
- Renal impairment defined as Creatinine clearance [Cockcroft-Gault] <45ml/min at screening
- Liver dysfunction (defined as ALT > 2xULN) at screening
- Evidence of cholestasis defined as elevated Total Bilirubin Levels, (TBL > 1.5 x ULN) and Alkaline Phosphatase, ALP (ALP > 1.5 x ULN), at screening
- Known hypothyroidism or hyperthyroidism
- Known autoimmune disease requiring active immunosuppressive treatment
- Any oral or intravenous immunosuppressive treatment including regular prednisolone, hydrocortisone or disease modifying drugs. [Inhaled or topical steroids are permissible]
- Patients on cytotoxic drugs and interferon-alpha
- Diabetics on oral hypoglycaemics/diet control with HbA1c (DCCT) > 8% (OR HbA1c (IFCC) > 64 mmol/mol), at screening. Diabetics on insulin are excluded from the study.
- Contraindication to IL-2 treatment or hypersensitivity to IL-2 or to any of its excipients
- Participation in a previous research trial in the last 3 years which involved exposure to significant ionising radiation (i.e. cumulative research radiation dose >5 mSv)
- Participation in a clinical trial where the patient has received a drug or new chemical entity within 30 days or 5 half-lives, or twice the duration of the biological effect of the drug (whichever is longer) prior to the first dose of trial medication, Visit 3 (Day 1).

- Any medical history or clinically relevant abnormality that is deemed by the principal investigator/delegate to make the patient ineligible for inclusion because of a safety concern
- Pregnant women or breast feeding women
- Patients who are COVID-19 PCR positive at the time of screening
- Known severe allergy to the CT-contrast agents.

6.3 Randomisation and Blinding

A sufficient number of patients will be randomised so that approximately 60 patients will complete the trial. Stratification at randomisation based on an ECG based ST-elevation status (to define non-STEMI or STEMI status) will be utilised to balance the groups. Patients will be randomised in a 1:1 fashion to either low dose interleukin-2 or placebo using an online randomisation system (Sealed Envelope).

The trial will be double-blind, with active and placebo doses appearing identical at point of issue and administration. The CUH central pharmacy will be unblinded and provided with a copy of the concealment list. A small team from the Cambridge Clinical Research Centre will also prepare maintenance doses in an unblinded manner but the investigator team (doctors and nurses) will remain blinded. Data analysis for the trial will be performed by a statistician who will be unblinded after the database lock.

The statistician, or delegate, may be unblinded for individual patients after their treatment period has concluded, to facilitate rapid reporting of safety events to the IDMC.

6.4 Study Variables

The table shows the schedule of assessments.

Visit no ->	1	2	3	4	5	6	7	8	9	10	11	12	13	14	15	16 (Remote where possible)
**Treatment Day**	**-14 to 0**	**-13 to 0**	**1 ^**	**2 ^**	**3 ^**	**4 ^**	**5 ^**	**12[a]**	**19[a]**	**26[a]**	**33[a]**	**40[a]**	**47[a]**	**54[a]**	**61[#]**	**82[a]**
Informed consent	x															
18F-FDG PET/CT Scan[b]		x													X[#]	
Demography[c]	x															
Height/weight	x															
Medical history	x															
Medication history	x															
Inclusion/exclusion	x	x	x													
Adverse events	x	x	x	x	x	x	x	x	x	x	x	x	x	x	x	x
Concomitant medications	x	x	x	x	x	x	x	x	x	x	x	x	x	x	x	x
Serum / urinary pregnancy test	x									x						X*
HbA1c	X															
Physical examination[d]	x		x	x	x	x	x	x	x	x	x	x	x	x	x	X*
Vital observations[e]	x		x	x	x	x	x	x	x	x	x	x	x	x	x	X*
ECG	x[f]		x	x	x	x	x	x	x	x	x	x	x	x	x	X*
ECHO	x[g]													x[h]	x[h]	
IMP administration			x	x	x	x	x	x	x	x	x	x	x	x		
Safety bloods[i]	x		x	X +	x	X +	x	x	x	x	x	x	x	x	x	X*
OPTIONAL Point of care bloods			x					x	x	x	x	x	x	x		
TSH	x														x	X*
Tregs and Lymphocytes			x				x	x		x		x		x	x	X*
PBMC assay			x				x	x		x		x		x	x	X*
Cardiac biomarkers[j]			x				x	x		x		x		x	x	X*
hsCRP	x															
Full lipid profile			X												x	
Stool sample (where feasible/possible)			X												X	
CTA															X[k]	
CTCA															X[l]	

^ These visits can be scheduled +/- 1 day.
[a] These visits can be scheduled +/- 3 days.
[b] PET/CT assessment to be preceded by a fingerprick blood glucose test.
[c] Including DOB, age, gender, race.
[d] Including cardiovascular, respiratory, gastrointestinal, neurological and skin examinations.
[e] Temperature, blood pressure, heart rate, respiratory rate and oxygen saturations.
[f] 12-lead ECG in triplicate with average QTcB.
[g] May be done anytime after screening prior to dosing (this can occur at an ad hoc visit). If a research ECHO cannot be performed, a clinical ECHO undertaken during the index admission can be used.
[h] May be done anytime between V14 and V15.
[i] Safety bloods (including but not limited to): haematology (FBC and differentials), clinical biochemistry (electrolytes, urea, creatinine), liver function (ALT, ALP, albumin, bilirubin). POC bloods may be done to aid dosing administration prior to review of formal bloods - as a minimum, review of Hb, sodium, potassium, creatinine should be sufficient.
[j] hsCRP, IL-6, Troponin I.
[k] May be done as separate visits. The CT carotid angiogram can be done in visit 2.
[l] Can be done as a separate visit.
* Assessments to be done physically at the hospital only if it is clinically indicated.
[#] PET/CT can be scheduled +/- 6 days.
[+] Safety bloods will only be done if deemed clinically necessary.

7 Sample Size

Sample size is based on an absolute difference of 0.2 in the primary endpoint (mean TBR_{max} in the index vessel) between placebo and active treatment. This is equivalent to a 10% difference from a reference value of 2.02 and equivalent to the size effect observed after atheroprotective therapy[5,6,7]. Interventions that have reported less than 10% difference in mean TBR_{max} in the index vessel between active treatment and placebo at the end of the treatment period[8,9] failed to reduce CV outcomes[10-12].

Assuming a SD of 0.24[5], 24 patients per arm, testing at 2-sided 5% significance level, will provide 80% power. Therefore, a sample size of 30 completed patients per arm was selected to account for scans which may not be analysable, or poor scans due to patient habitus, movement and so on.

This sample size of n=30 per group also allows the detection of a baseline-corrected 6% difference in mean TBR_{max} in the index vessel (mean 0.125, SD 0.166)[5] between placebo and active treatment at the end of the treatment period, at 2-sided 5% significance level and 80% power.

The two estimates were obtained from a previous publication[5] that compared a high and low dose intervention. The sample size is conservatively based assuming the higher SD estimate.

8 General Considerations

8.1 Timing of Analyses

The total trial duration for each patient will be approximately 13 weeks. This will include 2 weeks for recruitment and screening, 8 weeks of treatment and a follow up period of approximately 4 weeks after the last treatment visit. The follow-up PET/CT and CTCA will be scheduled within 2 weeks after the last treatment visit.

The final analysis will occur once the planned assessments for all randomised participants are completed and the data has been cleaned.

8.2 Analysis Populations

8.2.1 Full Analysis Population

Will include all patients who received the trial drug or placebo, completed the treatment course after randomisation and underwent the first and last PET/CT scans.

8.2.2 Randomised Population

Anyone allocated to any trial drug or placebo dose

8.2.3 Safety population

Anyone who consented to taking part in the trial and received any drug or placebo.

8.2.4 Non-safety population

This complex trial involved recruiting sick patients presenting ad hoc to the emergency door and organising complex tests including PET-CT at very short notice and an intricate setup for drug preparation in Pharmacy. For this reason, randomisation often was done very early prior to all baseline tests being successfully completed. This meant that a number of patients were randomised who were subsequently withdrawn. The withdrawn population consist of those who were:

A) withdrawn prior to randomisation and dosing

B) withdrawn post randomisation and prior to dosing

C) withdrawn after dosing commenced

Patients withdrawn in subset (C) will be included in the safety population as defined in section 8.2.3.

Patients withdrawn in subsets (A) and (B) define the Non-Safety population

8.3 Covariates and Subgroups

The following variables will be included in the primary analysis models:

- Treatment group
- ST-elevation (STEMI) status

8.3.1 Subgroup analysis

The following subgroups will be explored:

- ST-elevation (STEMI) status
- Baseline (screening) Inflammatory status based on hsCRP levels: 2-3, 3-10, >10 mg/L[13]
- Post-baseline incidence of high dose statins.

8.4 Missing Data

We do not anticipate a high level of drop out after treatment initiation. Hence we will report the number of non-missing values for comparison to the relevant population size. Statistics will be calculated based on complete cases, which assumes there is no association between a value being unobserved, and the underlying value.

If there was 5% or more missing data for the primary endpoint, multiple imputation will be performed and the primary analysis run again as a sensitivity analysis.

8.5 Multiple Testing

In the sensitivity analysis where the threshold used to define an active slice is varied, to control the false discovery rate to 5% or less the Benjamini-Hochberg process will be used, whereby

1. The *m* p-values are put into ascending order: $p_1 < p_2 < \ldots < p_m$.

2. The largest value k is identified where $p_k < k/m$ 5%.
3. Any null hypotheses p_1, p_2, …, p_k are rejected.

Multiple testing across analyses will be controlled using a gate-keeping approach, where the four primary analyses will be considered in the order given in section 10.1 and formal statistical significance obtains only all the preceding analyses have a nominal p-value less than 5%. For the sensitivity analyses that use the Benjamini-Hochberg process described above, they will collectively obtain formal statistical significance if 1 or more individual tests reject their null hypothesis.

9 Summary of Study Data

Summary statistics of endpoints broken down by treatment arm and visit where appropriate will be provided. Categorical variables will report percentages and counts (p% (x/n)); continuous variables will report, mean, median, SD, min and max. Exploratory figures in the form of stacked bar-charts and box-and-whisker plots for categorical and continuous variables respectively will be provided. If appropriate, spaghetti plots will be provided, to show the change in variables over time (visits), and colour coded by treatment.

9.1 Participant Disposition

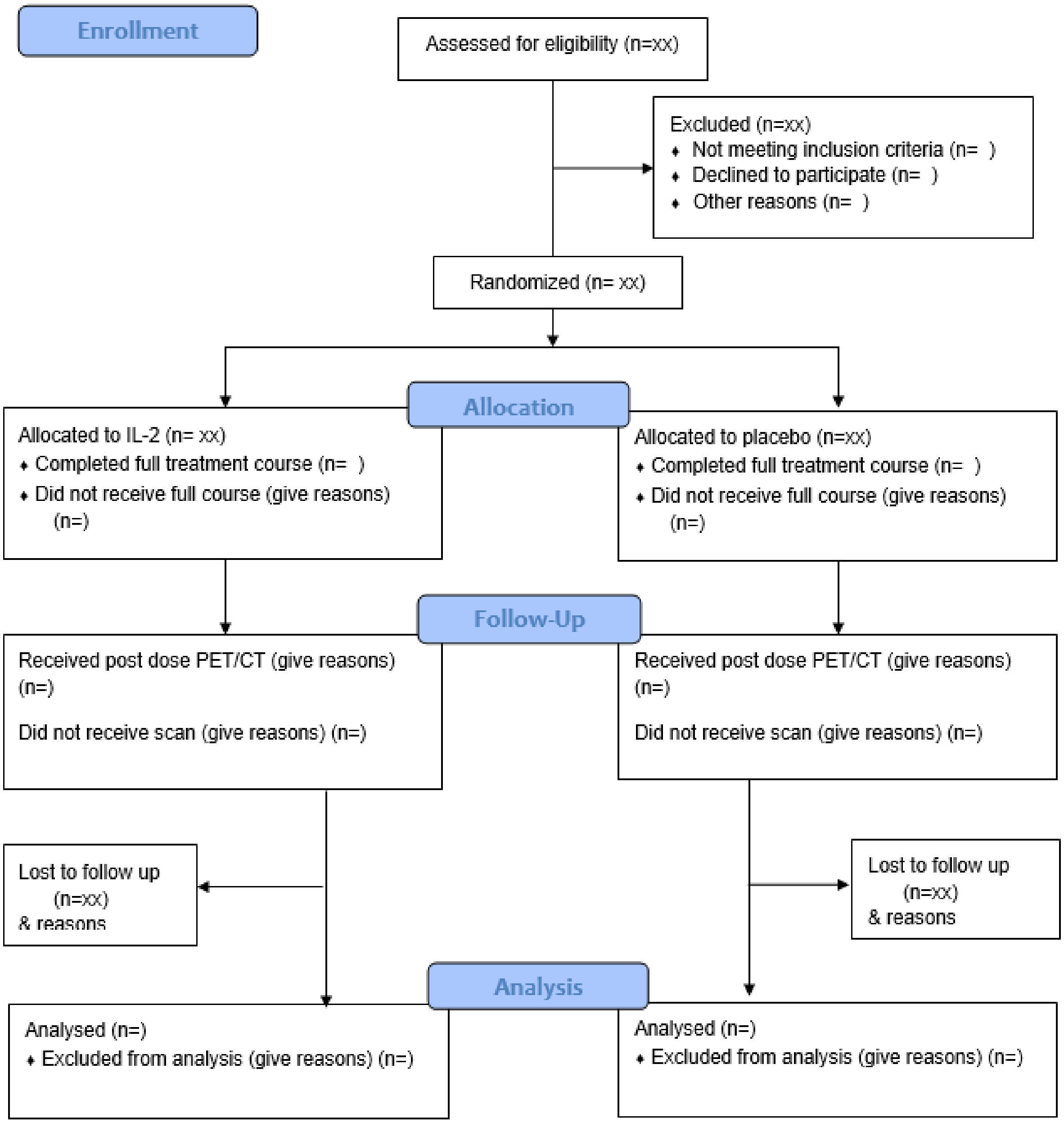

9.2 Derived variables

9.2.1 Primary endpoint

^{18}F-FDG PET/CT Scans are performed at visit 2 (Baseline) and 15. For each scan the injection time and the injected activity are recorded in MBq, as well as the count at the start of the scan in the aorta and carotid arteries in Kcps.

In the FDG PET-CT scans, for each cross-sectional slice of each vessel, mean and maximum SUV values will be calculated.

Scan quality: Scans are quality scored 1, 2 or 3, as assessed by a reader blinded to treatment. Scan regions (complete or single vessel scan) with quality score 3 (low quality) will be excluded from analysis.

Vessel: Data from slices in the following vessels will be analysed:

- LCC – Left common carotid
- RCC – Right common carotid
- IJV – Internal Jugular Vein
- SVC – superior vena cava
- BM– thoracic bone marrow
- ASA – ascending aorta

The acronym on the left is used in the scan analysis output to identify to which vessel a slice belongs.

The derived variables for at least 10% of the scans will be checked with a clinical member of the TMG prior to the analysis to ensure correct derivations.

9.2.1.1 Maximum tissue to blood ratio (TBRmax)

Maximum Tissue to Blood Ratio (Max TBR) will be calculated for each arterial slice and bone marrow within the thoracic vertebrae. v.

The equation used to calculate the TBRmax for each slice is as follows:

TBRmax = Max SUV (of each slice) / Average blood activity (mean SUV of the venous pool)

The different vessels of interest have different venous pool vessel corrections as per the table below

Vessel of Interest	Venous Pool Vessel
Aorta	Superior Vena Cava
Left common carotid	Internal Jugular Vein
Right common carotid	Internal Jugular Vein
Bone Marrow	Superior Vena Cava

9.2.1.2 Index vessel

For each participant at baseline the vessel with the highest mean TBR max value, will be termed the **"index vessel".**

9.2.1.3 Mean TBRmax

The Mean of the Max TBR will be calculated for the index vessel in each patient, for both scans

9.2.1.4 Active slices

In the literature the definition of an active slice is when TBR max >1.6 . However in this acute coronary syndrome population, the proportion of slices meeting this definition is anticipated to be too high, and near 100%, thus offering no level of stratification. Hence the analyses will consider a range of thresholds greater than 1.6 to consider how the treatment effects vary according to the choice of threshold.

9.2.1.5 Proportion of active slices

Proportion of vessel being active (in index vessel) = number of active slices in index vessel / total number of slices in index vessel

9.2.2 High Dose Statins

The subgroup listed in section 8.3.1is derived by search medication terms in the concomitant medication for any of the following terms

- statin
- ezitimibe

If there is also no stop date for the medication then the participant is included in the sub-group defined as "high dose statins".

9.2.3 CTCA, stool etc.

This exploratory work is planned and will be reported post hoc, therefore not in the scope of this statistical analysis plan.

9.3 Protocol Deviations

A listing of all major protocol deviation (eligibility, treatment error) will be provided. Additional information not recorded in the CRFs will be provided by the trial coordinators.

9.4 Demographic and Baseline Variables

The following demographic and baseline variables will be summarised according to Section 9.

9.4.1 Demographics

- Age
- Sex
- Weight
- Height
- Ethnicity
- hsCRP

9.4.2 Medical history

- Current diagnosis at admission
- Past medical history (IHD, hypertension, hypercholesterolemia, stroke/TIA, etc. according to medical history questionnaire CRF)
- Medication history (asprin, clopidogrel, ticagrelor, statin, etc. according to medical history questionnaire CRF)
- Smoking status
- GRACE score at admission

9.4.3 Intervention log details

- Whether the patient had an angiogram and what it showed
- The treatment plan
- Whether they are stented
- The type of intervention
- Diseased vessels

9.5 Concurrent Illnesses and Medical Conditions

MedDRA v25.1 was used for the adverse events, system organ class and preferred terms.

9.6 Treatment Compliance

The detailed treatment received will be summarised. Whether they received all doses with reasoning why doses had not been received.

10 Efficacy Analyses

The analysis will be based on the full analysis population.

10.1 Primary Efficacy Analysis

The primary analysis will be based on the full analysis population. The formal significance tests will be carried out in the order below, see section 8.5 on multiple testing.

Vascular inflammation will be quantified in 4 ways (2 using only active slices and 2 using all slices of the index vessel). This will follow a previous analysis from our group as published in Elkhawad et al[5]. This is focused on three vessels of interest (aorta, left common carotid, right common carotid), whilst bone marrow uptake is an exploratory endpoint and thus excluded.

1. Change from baseline (visit 2 to visit 15 in mean TBRmax in **all slices** within the index vessel

The mean TBR_{max} for all slices, at baseline, post treatment PET/CT scans, and the change from baseline, will be summarised using mean ± SD and split by treatment group.

The mean TBRmax in all slices in the index vessel (within each treatment group at pre-dose (v2) and post-dose (v15) will be analysed using a linear regression model with the variables in section 8.3 as covariates as well as the mean TBRmax at v2. The outcome variable will be the change from baseline mean TBRmax.

Example of model coding in R : Change from baseline mean TBRmax in all slices ~ Mean TBRmax in all slices (v2) + Treatment + STEMI status

Point estimates and corresponding 95% confidence intervals (CI) and P-values will be reported.

2. The distribution from **all slices** from all index vessels

   The group distribution of individual slices' TBRmax will be presented using a frequency histogram, comparing post-dose visit 15 between each treatment

group. The Kolmogorov-Smirnov statistic will be applied to measure the effect of treatment on TBRmax distribution. Bootstrapping at the participant level will be used to calculate p-values, to account for the dependency between slides within a participant.

3. Change from visit 2 to visit 15 in mean TBRmax in **active slices** within the index vessel

The mean TBR_{max} for active slices at baseline and post treatment PET/CT scans will be summarised using mean ± SD and split by treatment group.

The mean uptake of slices with TBRmax above a threshold within each treatment group at pre-dose (v2) and post-dose (v15) will be analysed using a linear regression model with the variables in section 8.3 as covariates as well as the mean TBRmax at v2. The outcome variable will be the change from baseline mean TBRmax.

Example of model: Change from baseline mean TBRmax in active slices ~ Mean TBRmax in active slices (v2) + Treatment + STEMI status

Point estimates and corresponding 95% confidence intervals (CI) and P-values will be reported

The reference value of the threshold, 1.6, as quoted in the protocol will be used alongside several other values (10.1.1). See section 8.5 for details of a Benjamini-Hochberg approach to multiple testing within this set of thresholds.

4. Probability of a slice being **active** within the index vessel

The percentage of active slices at baseline and post treatment PET/CT scans will be summarised using % ± SD and split by treatment group.

The number of active slices and the total number of slices at Visit 15 will be analysed using logistic regression to estimate the treatment effect comparing the probability of a slice being active. Baseline covariates to adjust for will include those specified in section 8.3, including baseline proportion of active slices.

Example of model: Proportion of slice being active (v15) ~ Proportion of slice being active (v2) + Treatment + STEMI status

Odds ratios and corresponding 95% confidence intervals (CI) and P-values will be reported. A forest plot will be presented that depicts the odds ratios and associated CI, with the p-values.

The reference value of the threshold, 1.6, as quoted in the protocol will be used alongside several other values (10.1.1). See section 8.5 for details of a Benjamini-Hochberg approach to multiple testing within this set of thresholds.

10.1.1 Sensitivity Analyses:

Analysis 3 and 4 above depend on the definition of an active slice (5.2.2 point 1), with a TBR max above a threshold. This is the first FDG PET-CT interventional drug study to be conducted in acutely presenting cardiovascular patients where the values may be out of range to previously studied groups. This is clearly demonstrated in a previous publication[8]. So we will consider analyses that repeat analyses 3 & 4 using a range of different thresholds. The treatment effect estimates and confidence intervals at a sequence of 10 thresholds spaced evenly from 1 to 3 will be calculated, and presented in a figure. The choice of range of thresholds may be changed should the variation in estimates need further exploration (expand the range) or focus (contract the range).

10.1.2 Subgroup Analysis

Subgroup analysis on the endpoints above will be carried out, as defined in section 8.3. Interactions with the subgroup and treatment effect will be estimated, and summary tables broken down by treatment arm and subgroup provided.

10.2 Secondary Efficacy Analyses

10.2.1 Vascular Inflammation

An analysis of the change from baseline in mean TBR max within all of the 3 vessels will be provided. A generalised least square model will be used to allow for within-participant correlation, and heteroscedasticity across the vessels, and fixed effects for the three baseline values for the three vessels, STEMI status, and treatment. An interaction between vessel and treatment will be consider to check the assumption of a constant treatment effect.

10.2.2 Lymphocyte Subsets

The following variables are captured at multiple visits over time

- CD3 %
- CD3 absolute
- CD4 %
- CD4 absolute
- CD8 %
- CD8 absolute
- Treg %
- Treg absolute
- Th1 %
- Th1 absolute
- Th2 %
- Th2 absolute
- Th17 %
- Th 17 absolute
- Tfh %
- Tfh absolute
- Teff total %
- Teff total absolute
- Teff memory %
- Teff memory absolute
- Teff central memory %
- Teff central memory absolute
- Non-Treg %
- Non-Treg absolute

Spaghetti plots over time and split by treatment group will be presented. At each time point, the values will be summarised using mean values and corresponding confidence intervals, and split by treatment group. The mean values will be plotted with time on the x-axis and endpoint scale on the y-axis.

For Tregs (% and absolute) a mixed model repeated measures analysis with an unstructured covariance matrix for the within – patient residual errors will be fitted between visits 3 and visits 15. The model will specify fixed effects of treatment, timing of assessment, STEMI status, and an interaction between treatment allocation and timing. A second model will be fitted that only considers the treatment period up to visit 15 and does not have an interaction between treatment and time: this estimates the treatment effects averaged over the treatment period.

10.3 Exploratory Efficacy Analyses

The exploratory endpoints 3, 4, 5, 6 in section 5.2.3 are not available at the time of writing and may be analysed later, outside the scope of this SAP.

10.3.1 Cardiac Biomarkers

For the change in Troponin spaghetti plots over time will be presented as well as a mixed model for repeated measures analysis, as described in section 10.2.2

The cardiac biomarkers apart from Troponin will be available after hard lock along with the data below. This analysis is planned but not in the scope of this report.

10.3.2 Ejection Fraction

For the change in ejection fraction, summaries for both visits will be given with box plots to show the distribution. A t-test will be performed comparing the change from pre- and post-treatment across treatment groups.

10.3.3 Bone Marrow

The mean TBRmax in slices from Bone Marrow will be summarised by visit (baseline and visit 14) along with change from baseline, broken down by treatment arm.

10.3.4 Full Lipid Profile

This will be summarised as a spaghetti plot split by treatment group, and by the subgroups defined in section 8.3.1

- Total cholesterol
- LDL cholesterol
- Triglycerides

11 Safety Analyses

The safety analyses will be based on the safety population.

This will exclude a small number of participants who consented but did not receive any drug: the Non-Safety population. Any adverse events for these participants will be listed separately.

11.1 Adverse Events

- Listings of AEs without ISRs
- AE grouping by preferred term and split by treatment group
- Listing of cardiovascular events
- Summary of cardiovascular events, split by treatment group
- Incidence of ISRs, split by treatment group
- Examination of ISRs

11.2 Deaths, Serious Adverse Events and other Significant Adverse Events

- Summary of SARs

- Incidence and listing of SAEs

11.3 Pregnancies

A listing broken down by treatment group will be provided.

11.4 Clinical Laboratory Evaluations

11.4.1 Safety bloods

Spaghetti plots split by treatment group will be provided for the following safety bloods:

- FBC
    a. TWBC
    b. Haemoglobin
    c. Platelets
- White blood cell count differential
    a. Neutrophils
    b. Lymphocytes
    c. Monocytes
    d. Eosinophils
    e. Basophils
- Electrolytes
- Urea
- Creatinine
- ALT
- ALP
- Albumin
- Bilirubin

11.4.2 Vital signs

Spaghetti plots split by treatment group will be provided for the following vital signs:

- Temperature
- Systolic blood pressure
- Diastolic blood pressure
- Heart rate
- Respiratory rate
- Oxygen saturation

11.5 Other Safety Measures

11.5.1 Physical examination

Any abnormal results will be reported and summaries of the physical examination will be given. This includes results from the cardiovascular, respiratory, gastrointestinal, neurological and skin examinations. The results will be split by treatment group.

11.5.2 Concomitant medications

- Listing of concomitant medications
- Concomitant medications grouped by medication name and split by treatment group

11.5.3 12-lead electrocardiogram (ECGs) recordings

The results that will be summarised by treatment group include:

- Any abnormalities of results
- Average QTcB

12 Figures

The following figures will be included:

- CONSORT diagram
- Spaghetti plots of bloods / cell counts / vital signs over time and split by treatment group
- PET/CT data to be presented as[5]:
  - Frequency histograms of all slices from index vessel
  - Forest plots of change in probability of slice being active
- T cell subset data to be presented as[14]:
  - Scatter plots of change in percentage over time, with bars representing the standard error or IQR
  - Plot representing the result of the MMRM with corresponding p-value

13 Reporting Conventions

P-values ≥0.001 will be reported to 3 decimal places; p-values less than 0.001 will be reported as “<0.001”. The mean, standard deviation, and any other statistics other than quantiles, will be reported to one decimal place greater than the original data. Quantiles, such as median, or minimum and maximum will use the same number of decimal places as the original data. Estimated parameters, not on the

same scale as raw observations (e.g. regression coefficients) will be reported to 3 significant figures.

14 Technical Details

The SAP is based on Version 4.2 (16 May 2023) of the protocol. The software package R version 4.1.3 is in use at the time of writing, but if upgraded, this will be documented. Copies of the code written will be stored. Each report and individual table of graph will have:

- The date and time included
- The name of the code file that produced the analysis
- The author
- A log capturing the version of the software and any external add on code used.
- Population used

The version control system Git will be used and individual code files will also have comments that convey:

- the author
- the date and time of writing
- description of any revisions
- references to inputs and outputs
- reference to any parent code file that runs the child code file

A reviewing statistician will independently reproduce the following:

Primary outcome summary statistics

15 Summary of Changes to the Protocol or from Previous Version of the SAP

This is to be provided by trial coordinators.

Version No.	History	Date
v1.0	Final Protocol	08 May 2019
v1.1	Incorporating REC/HRA comments	15 Jul 2019
v2.0	Incorporation MHRA feedback on initial submission	Aug 2019
V3.0	Changes to endpoints, visit schedule	01 March 2021
V4.0	Changes to exploratory endpoints, changes to angiograms, addition of optional stool sample	08Jun2022

V4.1	Clarification of basis on which sample size calculation was made, correction of typographical errors for pre-bloods in induction phase of dosing	01Nov2022
V4.2	Deletion to sentences with transcription errors, addition of a reference to secondary endpoint 1, correction of contact details of investigators	16May2023

16 References

1 Libby, P. Mechanisms of acute coronary syndromes and their implications for therapy. *The New England journal of medicine* **368**, 2004-2013, doi:10.1056/NEJMra1216063 (2013).

2 Arbab-Zadeh, A., Nakano, M., Virmani, R. & Fuster, V. Acute coronary events. *Circulation* **125**, 1147-1156, doi:10.1161/circulationaha.111.047431 (2012).

3 Mega, J. L. *et al.* Rivaroxaban in patients with a recent acute coronary syndrome. *The New England journal of medicine* **366**, 9-19, doi:10.1056/NEJMoa1112277 (2012).

4 Wiviott, S. D. *et al.* Prasugrel versus clopidogrel in patients with acute coronary syndromes. *The New England journal of medicine* **357**, 2001-2015, doi:10.1056/NEJMoa0706482 (2007).

5 Elkhawad, M. et al. Effects of p38 mitogen-activated protein kinase inhibition on vascular and systemic inflammation in patients with atherosclerosis. JACC. Cardiovascular imaging 5, 911-922, doi:10.1016/j.jcmg.2012.02.016 (2012).

6 Maki-Petaja, K. M. et al. Anti-tumor necrosis factor-alpha therapy reduces aortic inflammation and stiffness in patients with rheumatoid arthritis. Circulation 126, 2473-2480, doi:10.1161/circulationaha.112.120410 (2012).

7 Tahara, N. et al. Simvastatin attenuates plaque inflammation: evaluation by fluorodeoxyglucose positron emission tomography. Journal of the American College of Cardiology 48, 1825-1831, doi:10.1016/j.jacc.2006.03.069 (2006).

8 Fayad, Z. A. et al. Safety and efficacy of dalcetrapib on atherosclerotic disease using novel non-invasive multimodality imaging (dal-PLAQUE): a randomised clinical trial. Lancet (London, England) 378, 1547-1559, doi:10.1016/s0140-6736(11)61383-4 (2011).

9 Tawakol, A. et al. Effect of treatment for 12 weeks with rilapladib, a lipoproteinassociated phospholipase A2 inhibitor, on arterial inflammation as assessed with 18F-fluorodeoxyglucose-positron emission tomography imaging.

Journal of the American College of Cardiology 63, 86–88, doi:10.1016/j.jacc.2013.07.050 (2014).

10 O'Donoghue, M. L., Braunwald, E., White, H. D. & et al. Effect of darapladib on major coronary events after an acute coronary syndrome: The solid-timi 52 randomized clinical trial. JAMA 312, 1006–1015, doi:10.1001/jama.2014.11061 (2014).

11 Schwartz, G. G. et al. Effects of dalcetrapib in patients with a recent acute coronary syndrome. The New England journal of medicine 367, 2089–2099, doi:10.1056/NEJMoa1206797 (2012).

12 White, H. D. et al. Darapladib for preventing ischemic events in stable coronary heart disease. The New England journal of medicine 370, 1702–1711, doi:10.1056/NEJMoa1315878 (2014).

13 Ridker, P.M. (2003) "C-reactive protein," *Circulation*, 108(12). Available at: https://doi.org/10.1161/01.cir.0000093381.57779.67.

14 He, Jing, et al. Low-dose interleukin-2 treatment selectively modulates CD4+ T cell subsets in patients with systemic lupus erythematosus. Nature medicine 22.9 (2016): 991-993.

The Low-Dose Interleukin-2 For The Reduction Of Vascular Inflammation In Acute Coronary Syndromes -Clinical Outcomes And Follow-up (IVORY-FINALE) Study

IRAS Ref: 339102

Version No and Date: 3.0, 10 May 2024

Chief / Principal Investigator:	Dr. Joseph Cheriyan
Co-Investigators:	Dr. Rouchelle Sriranjan, Professor Ziad Mallat, Professor James Rudd, Dr. Stephen Hoole, Dr. Jacob Brubert
Sponsor:	Cambridge University Hospitals NHS Foundation Trust

AMENDMENT HISTORY

Amendment No.	Protocol Version No.	Date issued	Author(s) of changes	Details of Changes made
1	2.1	10Apr2024	Heike Templin	Addition of signature page
2	3.0	10May2024	Rouchelle Sriranjan	Addition of study endpoints, addition of clinical adjudication group

List details of all protocol amendments here whenever a new version of the protocol is produced.

SIGNATURE PAGE

The undersigned confirm that the following protocol has been agreed and accepted and that the Chief Investigator agrees to conduct the study in compliance with the approved protocol and will adhere to the principles outlined in the Declaration of Helsinki, the Sponsor's SOPs, and other regulatory requirement.

I agree to ensure that the confidential information contained in this document will not be used for any other purpose other than the evaluation or conduct of the investigation without the prior written consent of the Sponsor

I also confirm that I will make the findings of the study publically available through publication or other dissemination tools without any unnecessary delay and that an honest accurate and transparent account of the study will be given; and that any discrepancies from the study as planned in this protocol will be explained.

For and on behalf of the Study Sponsor:

Signature: .... Date: ....../....../......

Name (please print): ....

Position: ....

Chief Investigator:

Signature: .... Date: ....../....../......

Name: (please print): ....

KEY STUDY CONTACTS

**Chief Investigator**	Dr Joseph Cheriyan<br>Cambridge University Hospitals NHS Foundation Trust and Division of Experimental Medicine & Immunotherapeutics, Dept of Medicine, Univ. of Cambridge, Box 98, Level 3, ACCI Building, Hills Road, Cambridge, CB2 0QQ<br>Tel.: 01223 256653
**Investigators**	Dr Rouchelle Sriranjan<br>Division of Cardiovascular Medicine, Dept of Medicine, Level 2, Heart and Lung Research Institute, Cambridge Biomedical Campus, Papworth Road, Trumpington, Cambridge CB2 0AY
	Professor Ziad Mallat<br>Division of Cardiovascular Medicine, Dept of Medicine, Level 2, Heart and Lung Research Institute, Cambridge Biomedical Campus, Papworth Road, Trumpington, Cambridge CB2 0AY
	Professor James Rudd<br>Division of Cardiovascular Medicine, Dept of Medicine, Level 2, Heart and Lung Research Institute, Cambridge Biomedical Campus, Papworth Road, Trumpington, Cambridge CB2 0AY
	Dr Stephen Hoole<br>Royal Papworth Hospital NHS Foundation Trust, Papworth Road, Cambridge Biomedical Campus, Cambridge CB2 0AY
	Dr Jacob Brubert<br>Division of Cardiovascular Medicine, Dept of Medicine, Level 2, Heart and Lung Research Institute, Cambridge Biomedical Campus, Papworth Road, Trumpington, Cambridge CB2 0AY
	Dr Xiaohui Zhao, Victor Phillip Dahdaleh Heart and Lung Research Institute, Papworth Road, Cambridge Biomedical Campus, Cambridge CB2 0BB
**Study Co-ordinator**	Heike Templin<br>Cardiovascular Trials Office, Cambridge Clinical Trials Unit, Box 401, Cambridge University Hospitals NHS Foundation Trust, Hills Road, Cambridge, CB2 0QQ<br>Email: heike.templin@nhs.net
**Sponsor**	Cambridge University Hospitals NHS Foundation Trust<br>Research & Development Department, Box 277,<br>Addenbrooke's Hospital, Hills Road,<br>Cambridge CB2 0QQ<br>Tel: 01223 245151<br>Email: cuh.research@nhs.net

LIST OF CONTENTS

GENERAL INFORMATION	Page No.
TITLE PAGE	1
AMENDMENT HISTORY	2
SIGNATURE PAGE	3
KEY STUDY CONTACTS	4
LIST of CONTENTS	5
STUDY SYNOPSIS	6
ABBREVIATIONS	7
STUDY FLOW CHART	8
**SECTION**	
1. BACKGROUND AND RATIONALE	9
2. OBJECTIVES	10
3. ENDPOINTS	10
4. STUDY DESIGN/METHODS	12
5. STUDY PROCEDURES AND INTERVENTIONS	13
6. STATISTICS	15
7. ETHICAL AND REGULATORY COMPLIANCE	16
8. DISSEMINATION POLICY	18
REFERENCES	19
APPENDIX	20

SYNOPSIS

The IVORY trial was a randomized controlled trial in which low dose IL-2 was compared to placebo in patients following an acute coronary syndrome. IVORY-FINALE is a follow-up study to review the medium to long-term clinical outcomes from the IVORY study.

**Study Title**	The Low-Dose Interleukin-2 For The Reduction Of Vascular Inflammation In Acute Coronary Syndromes - Clinical Outcomes And Follow-Up (IVORY-FINALE) Study
**Internal ref. no.**	A096877
**Study Design**	Observational study
**Study Participants**	Participants who were randomised into and completed the IVORY trial
**Planned Sample Size (if applicable)**	60
**Follow-up duration (if applicable)**	5 years from first dose of IL2 or placebo administration for patients who completed the IVORY trial.
**Planned Study Period**	6 years
**Primary Objective**	To study the medium-term to long-term effects of low dose IL2 treatment compared to placebo on major adverse cardiovascular outcomes (composite of CV death, non-fatal myocardial infarction, resuscitated cardiac arrest, ischaemic stroke, or unplanned coronary revascularisation) in participants who completed the IVORY trial.
**Secondary Objectives**	To collect data on additional clinical outcomes including all cause death, haemorrhagic stroke, new atrial fibrillation, ventricular arrhythmias, hospitalisation due to cardiovascular causes (heart failure, stable and unstable angina, TIAs, valve related), amputations and revascularisation due to peripheral vascular disease.

ABBREVIATIONS

ACS	Acute coronary syndrome
eCRF	Electronic case report form
CRF	Case Report Form
CT	Computerized tomography
CV	Cardiovascular
18f-FDG	2-Deoxy-2-[fluorine-18] fluorodeoxyglucose
IL2	Interleukin 2
IL2Rα	Interleukin 2 receptor
IVORY	Low-dose interleukin-2 for the reduction of vascular inflammation in Acute Coronary Syndromes
MACE	Major adverse cardiovascular events
MI	Myocardial infarction
PET	Positron emission tomography
Teffs	Effector T cells
Tregs	Regulatory T cells
TIA	Transient Ischaemic Attack

STUDY FLOW CHART

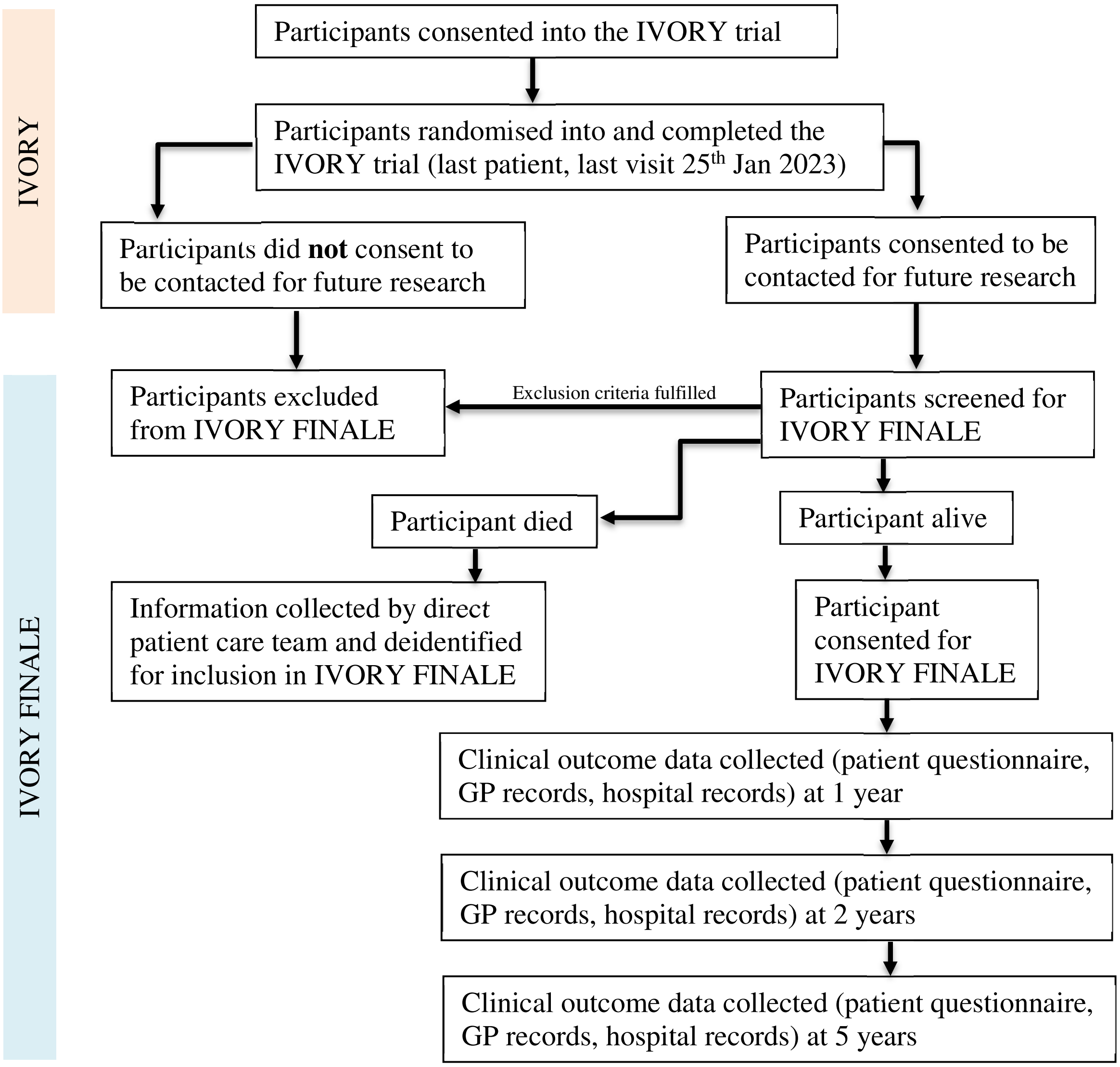

Figure 1: IVORY and IVORY-FINALE study schematic

1. BACKGROUND AND RATIONALE

Inflammation plays a pivotal role in the initiation and progression of atherosclerosis, which accounts for an overwhelming majority of cardiovascular events. The role of the immune system in the pathophysiology of atherosclerosis and plaque instability is multifaceted and orchestrated by both the innate and adaptive components of the immune system (1). Due to their critical role in mediating immune tolerance and antiatherosclerotic effects, natural and inducible regulatory T cells (Tregs) (CD4+CD25+FOPX3) are a focus of novel therapeutic strategies (2).

Initial studies in preclinical models showed that Tregs reduced plaque inflammation, slowed progression and even led to the regression of atherosclerosis (3,4). Tregs have also been implicated in moderating the post-ischaemic immune responses triggered by self--antigens presented by necrotic myocardium. In line with this finding, it has also been demonstrated that Tregs promote myocardial wound healing, attenuate adverse left ventricular remodelling and improve cardiac function (5,6,7,8). Low circulating levels of Treg cells and their impaired function is associated with an increased risk of acute coronary events and plaque instability (9,10).

Interleukin 2 (IL--2) plays a crucial role in immune homeostasis by determining the balance between Teffector cells (Teffs) and Tregs. While IL--2 is implicated in the development of both Teffs and Tregs, unlike Teffs, Treg cells constitutively express an IL--2 receptor (IL--2Rα) which binds to IL--2 with high affinity, making this subset very sensitive to IL--2. Of the cytokines involved in Treg development and stability, IL--2 is the most essential.

The Low-Dose Interleukin-2 (IL2) For The Reduction Of Vascular Inflammation In Acute Coronary Syndromes (IVORY) Trial, was a double-blind, randomised, placebo-controlled, phase II trial (NCT04241601). In that trial, we hypothesized that treatment with low-dose IL2 would reduce vascular inflammation measured by 2-Deoxy-2-[fluorine-18] fluorodeoxyglucose (18F-FDG) positron emission tomography/computed tomography (PET/CT). Patients with acute coronary syndrome (ACS) and high-sensitivity C-reactive protein (CRP) levels >2 mg/L were randomised to receive either 1.5×10^6 IU of aldesleukin or placebo (1:1), within 14 days of ACS. Dosing included a once daily induction phase (5 days) and a once weekly maintenance phase (7 weeks). 18F-FDG-PET/CT imaging of the ascending aorta and carotid arteries was performed before and after treatment.

Sixty (60) patients completed the IVORY trial. The results have shown that extended treatment with low-dose IL2 was safe and reduced vascular inflammation in patients with ACS. As atherosclerosis and its complications are driven by inflammation we hypothesise that extended treatment with low-dose IL2 may reduce adverse cardiovascular outcomes compared to placebo.

The IVORY-FINALE study is an observational study which will follow participants who completed follow-up in the main IVORY clinical trial.

In this follow-up study we aim to collect cardiovascular clinical outcome data for patients who completed the IVORY clinical trial (IVORY visit schematic as above, Fig. 1). Clinical outcome data will include major adverse cardiovascular events (MACE), defined as cardiovascular death, non-fatal myocardial infarction, resuscitated cardiac arrest, ischaemic stroke, or unplanned coronary revascularisation. In addition, data on adverse events such as all cause death, haemorrhagic stroke, new atrial fibrillation, ventricular arrhythmias, hospitalisation due to cardiovascular causes (e.g. stable and unstable angina, TIAs, heart failure), amputations and revascularisation due to peripheral vascular disease.

2. OBJECTIVES

2.1 Primary Objective

To study the effects of low dose IL2 treatment compared to placebo on major adverse cardiovascular outcomes (composite of cardiovascular death, resuscitated cardiac arrest, non-fatal MI, ischaemic stroke, or unplanned coronary revascularization*) in patients who completed the IVORY clinical trial.

*Unplanned revascularisation includes any procedures that were not done as elective staged procedures planned at the index admission.

2.2 Secondary Objectives

- To evaluate the medium to long term effects of extended treatment with IL2 on death due to cardiovascular causes.
- To evaluate the medium to long term effects of extended treatment with IL2 on resuscitated cardiac arrest.
- To evaluate the medium to long term effects of extended treatment with IL2 on non-fatal MI. This will include non-ST elevation myocardial infarction (NSTEMI) and ST elevation myocardial infarction (STEMI).
- To evaluate the medium to long term effects of extended treatment with IL2 on ischaemic stroke.
- To evaluate the medium to long term effects of extended treatment with IL2 on unplanned coronary revascularization.
- To evaluate the medium to long term effects of IL2 treatment on hospitalisations due to cardiovascular causes (e.g angina (stable and unstable), TIAs, valve related).
- To evaluate the medium to long term effects of extended IL2 treatment on all-cause death.
- To evaluate the medium to long term effects of extended IL2 treatment on hospitalisations due to symptoms from heart failure (these will include admission due to pulmonary oedema and congestive cardiac failure).
- To evaluate the medium to long term effects of extended IL2 treatment on revascularisation for peripheral vascular disease.
- To evaluate the medium to long term effects of extended IL2 treatment on amputations due to peripheral vascular disease.
- To evaluate the medium to long term effects of extended IL2 treatment on haemorrhagic stroke.
- To evaluate the medium to long term effects of extended IL2 treatment on new atrial fibrillation diagnosis.
- To evaluate the medium to long term effects of extended IL2 treatment on ventricular arrhythmia (sustained ventricular tachycardia and ventricular fibrillation).

3. STUDY ENDPOINTS

3.1 Primary Endpoint

The difference in major adverse cardiovascular outcomes (composite of cardiovascular death, resuscitated cardiac arrest, non-fatal MI, ischaemic stroke, or unplanned coronary revascularisation*) who completed the IVORY clinical trial between low-dose IL-2 and placebo at the various time points (1, 2 and 5 years).

* Unplanned revascularisation includes any procedures that were not done as elective staged procedures planned at the index admission.

3.2 Secondary Endpoints

- The difference in the number of deaths due to cardiovascular causes between those treated with IL2 and placebo at follow-up (1, 2 and 5 years).
- The difference in the number of resuscitated cardiac arrests between those treated with IL2 and placebo at follow-up (1, 2 and 5 years).
- The difference in the number of non-fatal MI between those treated with IL2 and placebo at follow-up (1, 2 and 5 years). This will include non-ST elevation myocardial infarction (NSTEMI) and ST elevation myocardial infarction (STEMI).
- The difference in the number of ischaemic stroke arrest between those treated with IL2 and placebo at follow-up (1, 2 and 5 years).
- The difference in the number of unplanned coronary revascularization between those treated with IL2 and placebo at follow-up (1, 2 and 5 years).
- The difference in the number of hospitalisations due to cardiovascular causes (e.g angina (stable and unstable), TIAs, valve related) between those treated with IL2 and placebo at follow-up (1, 2 and 5 years).
- The difference in the number of all-cause deaths between those treated with IL2 and placebo at follow-up (1, 2 and 5 years).
- The difference in the number of hospitalisations due to symptoms from heart failure (these will include admission due to pulmonary oedema and congestive cardiac failure) between those treated with IL2 and placebo at follow-up (1, 2 and 5 years).
- The difference in the number of revascularisation for peripheral vascular disease between those treated with IL2 and placebo at follow-up (1, 2 and 5 years).
- The difference in the number of amputations due to peripheral vascular disease between those treated with IL2 and placebo at follow-up (1, 2 and 5 years).
- The difference in the number of haemorrhagic stroke between those treated with IL2 and placebo at follow-up (1, 2 and 5 years).
- The difference in the number of new atrial fibrillation diagnoses between those treated with IL2 and placebo at follow-up (1, 2 and 5 years).
- The difference in the number of ventricular arrhythmia (sustained ventricular tachycardia and ventricular fibrillation) episodes between those treated with IL2 and placebo at follow-up (1, 2 and 5 years).

STUDY DESIGN AND METHODS

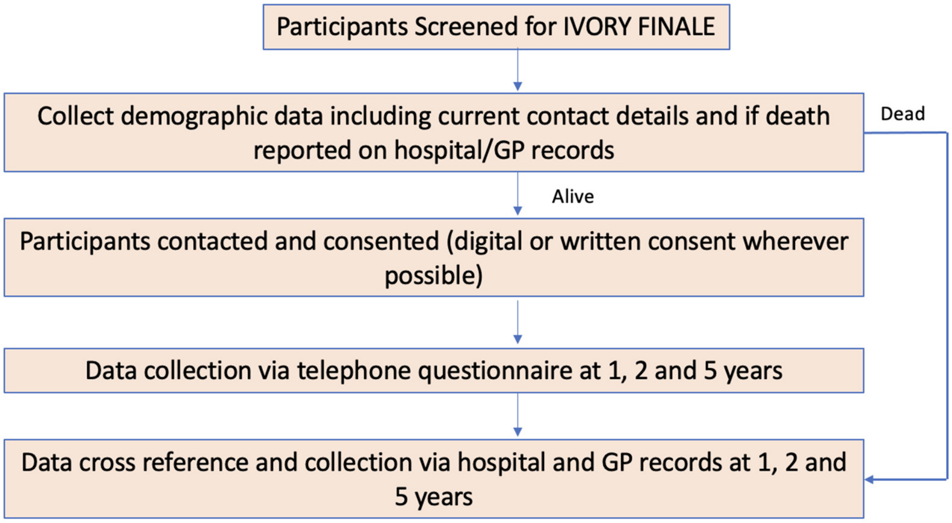

Figure 2: Schematic flow chart for IVORY-FINALE

IVORY-FINALE is a follow-up study for patients in the IVORY trial, as shown in Figure 1. Screening, consent, data collection and entry will be performed by a research nurse or research clinician, as shown in Figure 2.

The IVORY trial IDs will be used for this follow-up observational study.

Data analysis will include the comparison of clinical outcome data between the placebo and low dose IL2 arm. These will be done for each defined event as well as a composite of events as described.

4.1 STUDY PARTICIPANTS

4.2 Inclusion Criteria

Participants who completed the full per-protocol treatment regime of low-dose IL2 or placebo having attended the final dosing visit in the IVORY trial. IVORY patients who previously consented to have their medical records inspected in the IVORY trial and who have already passed away at the commencement of IVORY-FINALE will also be included in analyses - see section 5.2.

4.3 Exclusion Criteria

- Patients who decline participation
- Patients who did not consent to being contacted about future research
- Patients who were withdrawn from the IVORY trial for any reason

5. STUDY PROCEDURES AND INTERVENTIONS

5.1 Recruitment

Participants will be screened using the IVORY trial participant database. Participants who consented to be contacted for future research received all scheduled doses of either placebo or IL2 and are alive at the time of data collection will be approached either via telephone or email for inclusion in this study.

5.2 Informed Consent

Participants in the IVORY trial were asked whether they consented to be contacted for further trials and research studies as shown by the IVORY trial consent form included in additional materials.

For participants who consented to be contacted regarding future research and are alive at the time of recruitment of the IVORY-FINALE study, these patients will be contacted via telephone or email. Participants will be given an opportunity and time to be able to raise questions regarding the study. Participants will receive a Participant Information Sheet (PIS) via email and post.

Informed consent will be obtained prior to data collection for every patient who is alive at the time of commencement of the IVORY-FINALE study.

In the instance where a patient dies between data collection timepoints (at 1, 2, and 5 years) in IVORY-FINALE, their consent given at inclusion in IVORY-FINALE is believed to extend beyond death (Page 39 of UKRI/MRC/NHS HRA Consent and Participant Information Guidance, 28/08/2020 and by NHS England), and their data will be collected at the timepoint following death.

Given high risk ACS patients were recruited into the IVORY trial, it is likely that some participants who completed the trial will have died following the IVORY trial, but before inclusion in IVORY-FINALE.

The NHS Health Research Authority Confidentiality Advisory Group have published guidance to address this commonly-arising situation. The Precedent Set Criteria, updated 14th November 2023, are published here: https://www.hra.nhs.uk/about-us/committees-and-services/confidentiality-advisory-group/cag-precedent-set-review-pathway/precedent-set-criteria/. Category 2 addresses access to deceased person's confidential patient information. As per HRA precedent set criteria, where an IVORY participant has died prior to commencement of IVORY-FINALE and is unable to give consent the minimum of confidential information shall be disclosed. The team involved in the direct care of the participant during IVORY will extract the information in a deidentified format for analysis by the research team in IVORY-FINALE.

This is illustrated in Figure 1.

5.3 Study Assessments/Interventions

This is an observational study. No intervention will be performed.

Clinical outcome data will be collected from GP and hospital databases.
Participant questionnaires (see Appendix A) will be distributed to living participants via paper or telephone, whichever is more convenient for the patient. Outcomes reported in the questionnaires will be cross-referenced with GP or hospital records.

Data will be anonymised, and then recorded using the IVORY trial IDs.

Any trial related documentation that is sent to the study coordination centre must not contain participant identifiable data.

5.4 Clinical endpoint adjudication

An independent clinical endpoint adjudication committee (composed of 3 clinicians not involved with either IVORY or IVORY-FINALE) will review the end-point data in a blinded manner prior to final data lock.

5.5 Definition of End of Study

The end of the study will be 12 months after the 5 year timepoint that the last IVORY trial patient was initially dosed. This is defined as 15 November 2028.

6. STATISTICS

6.1 The Number of Participants

Sixty patients who completed the IVORY trial. IVORY-FINALE aims to recruit those patients. Patients who are alive will be approached to participate in IVORY-FINALE. Patients who have died at the time of commencement of IVORY-FINALE but previously provided consent will also be included in the IVORY-FINALE analyses.

6.2 Sampling

Not applicable.

6.3 Analysis of Endpoints

A composite primary endpoint will be analysed using a win-ratio method (11). Fatal outcome(s) (i.e. cardiovascular-related death) are prioritised over less severe or non-fatal outcomes (i.e. resuscitated cardiac arrest, ischaemic stroke, non-fatal myocardial infarction, and unplanned coronary revascularisation) within the composite outcome.

Simulations in Dong et al. (12) show that the normal approximation of the logarithm of the win ratio is satisfactory with sample sizes as small as 25 per group. Secondary endpoints will be analysed using ANOVA, T-test and regression methods where appropriate.

7. ETHICAL AND REGULATORY COMPLIANCE

7.1 Data Protection and Patient Confidentiality

All investigators and study site staff involved in this study must comply with the requirements of the Data Protection Act 2018 and Trust Policy with regards to the collection, storage, processing, transfer and disclosure of personal information and will uphold the Act's core principles.

Personal data will be recorded electronically on data forms that are entered into an access-restricted computer and encrypted and located on the secure servers. The participants will be identified by a study-specific participant number in any database.

During the study, data containing personal information such as name of the participant and contact details will be stored securely with restricted user access. Personal information will be removed from study documents such as source documents as soon as practical then replaced with an ID number from a linkage document kept separately and securely. Documentation containing such personal information will be destroyed or de-identified 6-12 months after the end of the study.

Documents containing personal or identifying data such as the code break and consent forms will also be stored in cabinets with restricted user access during the study.

At the end of ethical approval the study files will be archived in Cambridge University Hospital for fifteen years, after which time the custodian will agree a date for destruction and arrangements for confidential destruction will then be made.

Electronic data will be held on secure network drives/hard disks/servers on password protected computers within locked offices. Backup copies of files will be made regularly weekly and stored on a different server/external hard drive. These back up locations will be subject to the same security principles as the primary locations. When datasets are complete, the primary copy will remain at the study site where it will be transferred onto optical media, e.g. DVD/external drive and undergo archiving for hard copy data. Any copies leaving the study site will be completely anonymised/de-identified.

7.2 Indemnity

The study is sponsored by Cambridge University Hospitals NHS Foundation Trust.

Cambridge University Hospitals NHS Foundation Trust, as a member of the NHS Clinical Negligence Scheme for Trusts, will accept full financial liability for harm caused to participants in the study caused through the negligence of its employees and honorary contract holders. There are no specific arrangements for compensation should a participant be harmed through participation in the study, but no-one has acted negligently.

7.3 Protocol Amendments

Protocol amendments must be reviewed and agreement received from the Sponsor for all proposed amendments prior to submission.

7.4 Ethical committee review

Before the start of the study or implementation of any amendment we will obtain approval of the study protocol, protocol amendments, informed consent forms and other relevant documents e.g., advertisements and GP information letters if applicable, from the REC. All correspondence with the REC will be retained in the Study Master File/Investigator Site File.

Annual reports will be submitted to the REC in accordance with national requirements. It is the Chief Investigator’s responsibility to produce the annual reports as required.

7.5 Declaration of Helsinki and Good Clinical Practice

The study will be performed in accordance with the spirit and the letter of the declaration of Helsinki, the conditions and principles of Good Clinical Practice, the protocol and applicable local regulatory requirements and laws.

7.6 GCP Training

All study staff must hold evidence of appropriate GCP training or undergo GCP training prior to undertaking any responsibilities on this trial. This training should be updated every 2 years or in accordance with your Trust’s policy.

8. DISSEMINATION POLICY

Ownership of the data arising from this trial resides with the study team. On completion of the study the data will be analysed and tabulated and a Final Study Report prepared.

REFERENCES

1. Roy, Payel, Marco Orecchioni, and Klaus Ley. "How the immune system shapes atherosclerosis: roles of innate and adaptive immunity." *Nature Reviews Immunology* 22.4 (2022): 251-265
2. Sriranjan R, Zhao TX, Tarkin J, Hubsch A, Helmy J, Vamvaka E, Jalaludeen N, Bond S, Hoole SP, Knott P, Buckenham S. Low-dose interleukin 2 for the reduction of vascular inflammation in acute coronary syndromes (IVORY): protocol and study rationale for a randomised, double-blind, placebo-controlled, phase II clinical trial. BMJ open. 2022 Oct 1;12(10):e062602.
3. Ait-Oufella H, Salomon BL, Potteaux S, et al. Natural regulatory T cells control the development of atherosclerosis in mice. Nat Med 2006;12:178–80.
4. Sharma M, Schlegel MP, Afonso MS, et al. Regulatory T cells license macrophage pro-resolving functions during atherosclerosis regression. Circ Res 2020;127:335–53.
5. Hofmann U, Beyersdorf N, Weirather J, et al. Activation of CD4+ T lymphocytes improves wound healing and survival after experimental myocardial infarction in mice. Circulation 2012;125:1652–63.
6. Matsumoto K, Ogawa M, Suzuki J-ichi, et al. Regulatory T lymphocytes attenuate myocardial infarction-induced ventricular remodeling in mice. Int Heart J 2011;52:382–7.
7. Dobaczewski M, Xia Y, Bujak M, et al. Ccr5 signaling suppresses inflammation and reduces adverse remodeling of the infarcted heart, mediating recruitment of regulatory T cells. Am J Pathol 2010;176:2177–87. 11
8. Yu X, Newland SA, Zhao TX, et al. Innate Lymphoid Cells Promote Recovery of Ventricular Function After Myocardial Infarction. J Am Coll Cardiol 2021;78:1127–42.
9. Wigren M, Björkbacka H, Andersson L, et al. Low levels of circulating CD4+Foxp3+ T cells are associated with an increased risk for development of myocardial infarction but not for stroke. Arterioscler Thromb Vasc Biol 2012;32:2000–4. 13
10. Cheng X, Yu X, Ding Y-J, et al. The Th17/Treg imbalance in patients with acute coronary syndrome. Clin Immunol 2008;127:89–97.
11. Pocock, S. J., Ariti, C. A., Collier, T. J., & Wang, D. (2012). The win ratio: A new approach to the analysis of composite endpoints in clinical trials based on clinical priorities. *European Heart Journal*, *33*(2), 176–182. https://doi.org/10.1093/eurheartj/ehr352
12. Dong, G., Li, D., Ballerstedt, S., Vandemeulebroecke, M. (2016). A generalized analytic solution to the win ratio to analyze a composite endpoint considering the clinical importance order among components. *Pharmaceutical Statistics* 15(5):430–437. doi:10.1002/pst.1763

APPENDICES

Schedule of Events

Procedures	Time events (time post-IVORY randomisation)				
	**Received first dose of IL2 or placebo on t_0**	**Screening**	**1 year call (t_0+1y)**	**2 year call (t_0+2y)**	**5 year call (t_0+5y)**
Demographic review		x	x	x	x
Consent form			x		
Telephone/email contact questionnaire			x	x	x
Review of medical record			x	x	x
