# Supplementary material for: Anti-inflammatory therapy with low-dose IL-2 in acute coronary syndromes: a randomized phase 2 trial
Source: Nat Med. 2026 Jan 8;32(2):624–32. doi: 10.1038/s41591-025-04090-y (PMC12920103; doi:10.1038/s41591-025-04090-y)
Supplement: Supplementary file 2 — Reporting Summary [file 41591_2025_4090_MOESM2_ESM.pdf]

Corresponding author(s): Ziad Mallat, Rouchelle Sriranjan-Rothwell

Last updated by author(s): Oct 19, 2025

Reporting Summary

Nature Portfolio wishes to improve the reproducibility of the work that we publish. This form provides structure for consistency and transparency in reporting. For further information on Nature Portfolio policies, see our Editorial Policies and the Editorial Policy Checklist.

Statistics

For all statistical analyses, confirm that the following items are present in the figure legend, table legend, main text, or Methods section.

n/a	Confirmed	
☐	☒	The exact sample size (*n*) for each experimental group/condition, given as a discrete number and unit of measurement
☐	☒	A statement on whether measurements were taken from distinct samples or whether the same sample was measured repeatedly
☐	☒	The statistical test(s) used AND whether they are one- or two-sided<br>*Only common tests should be described solely by name; describe more complex techniques in the Methods section.*
☐	☒	A description of all covariates tested
☐	☒	A description of any assumptions or corrections, such as tests of normality and adjustment for multiple comparisons
☐	☒	A full description of the statistical parameters including central tendency (e.g. means) or other basic estimates (e.g. regression coefficient) AND variation (e.g. standard deviation) or associated estimates of uncertainty (e.g. confidence intervals)
☐	☒	For null hypothesis testing, the test statistic (e.g. *F*, *t*, *r*) with confidence intervals, effect sizes, degrees of freedom and *P* value noted<br>*Give P values as exact values whenever suitable.*
☒	☐	For Bayesian analysis, information on the choice of priors and Markov chain Monte Carlo settings
☐	☒	For hierarchical and complex designs, identification of the appropriate level for tests and full reporting of outcomes
☒	☐	Estimates of effect sizes (e.g. Cohen's *d*, Pearson's *r*), indicating how they were calculated

Our web collection on statistics for biologists contains articles on many of the points above.

Software and code

Policy information about availability of computer code

Data collection	Not Applicable
Data analysis	Not applicable

For manuscripts utilizing custom algorithms or software that are central to the research but not yet described in published literature, software must be made available to editors and reviewers. We strongly encourage code deposition in a community repository (e.g. GitHub). See the Nature Portfolio guidelines for submitting code & software for further information.

Data

Policy information about availability of data

All manuscripts must include a data availability statement. This statement should provide the following information, where applicable:

- Accession codes, unique identifiers, or web links for publicly available datasets
- A description of any restrictions on data availability
- For clinical datasets or third party data, please ensure that the statement adheres to our policy

All data supporting the findings of this study are available within the paper.

Human research participants

Policy information about studies involving human research participants and Sex and Gender in Research.

Reporting on sex and gender	Findings of this trial apply to both sexes.<br>Sex was considered in the study design. As this is a phase 2 trial with limited safety data on females during pregnancy, breast feeding and women of childbearing potential, they were excluded. Additionally, the primary endpoint was based on an imaging technique involving radiation - the use of which is contraindicated in women of childbearing potential from a regulatory perspective. These limited the population of women who could be included for scientific, ethical and regulatory perspectives.<br>We have reported the sex of the participants in the demographics table. No sex-based analysis was not carried out either priori or post-hoc, as no significant differences were noted nor expected between the groups with respect to sex. Furthermore, given this was Phase 2 experimental medicine study, the small sample size would not have enabled any meaningful conclusions on the basis of sex. Information with respect to gender was not collected<br>Information with respect to sex was collected from self-reporting and corroborated with medical records. Information with respect to gender was not collected.
Population characteristics	A detailed list of baseline characteristics are supplied in the manuscript (page 16. Table 1) . For all imaging analyses, subgroup interactions for ST elevation status, baseline hsCRP levels, and high-dose versus non-high-dose statin use were explored. In any change from baseline analyses presented, baseline values were used as a covariate.
Recruitment	The trial design is outlined in the manuscript (pages 7-9) and the full clinical trial protocol has been submitted on the submission portal. These describe in detail the full inclusion and exclusion criteria for participants. In summary, patients diagnosed with acute coronary syndromes and had a hsCRP >2mg/L were recruited into the trial if they fulfilled eligibility criteria and passed screening. Dosing was commenced within 14 days of their index admission with ACS. As ST elevation status, baseline HsCRP and variation in statin use could have impacted the results, subgroup interactions were explored for these groups as described in the manuscript and the statistical analysis plan for IVORY. Patients were recruited sequentially and no self-selection bias was anticipated or observed.
Ethics oversight	The IVORY Trial was approved by Yorkshire & The Humber - Sheffield Research Ethics Committee, Health Research Authority (REC ref. 19/YH/0171) and IVORY FINALE study (West Midlands - Edgbaston Research Ethics Committee , Health Research Authority(REC ref 24/WM/0059)<br><br>For IVORY, an independent Data Monitoring Committee (unblinded) reviewed the safety data on a regular basis as predefined in the protocol.

Note that full information on the approval of the study protocol must also be provided in the manuscript.

Field-specific reporting

Please select the one below that is the best fit for your research. If you are not sure, read the appropriate sections before making your selection.

☒ Life sciences ☐ Behavioural & social sciences ☐ Ecological, evolutionary & environmental sciences

For a reference copy of the document with all sections, see nature.com/documents/nr-reporting-summary-flat.pdf

Life sciences study design

All studies must disclose on these points even when the disclosure is negative.

Sample size	The sample size for the IVORY trial was based on an absolute difference of 0.2 in mean TBRmax of the index vessel between placebo and low-dose IL-2 at the end of the treatment period. This is equivalent to the effects observed with therapies such as high-dose statin therapy (when compared to low-dose statin therapy), which reduce MACE in ACS.
Data exclusions	No data exclusions were made in analysing the primary endpoints as all post dose scans which were analysable. For the MACE follow-up in IVORY-FINALE only those who completed the study were included. The reason for this was a) we anticipated a low-dropout due to treatment side-effects (which was the case) and b) given this is a phase two study in high-risk individuals, we anticipated and observed MACE prior to commencement of treatment in those who were randomised and withdrawn (e.g readmission to hospital and inability to dose within 14 days of admission or CV death)
Replication	There was excellent intra-observer [ICC (intraclass coefficient) = 0.97 (0.96 – 0.98)] and inter-observer [ICC= 0.93 (0.92 – 0.94)] agreement between measurements using 18F-FDG PET/CT scans. Second person checks and intra-observer checks were done for 10% of all scans as per previous studies.
Randomization	Patients were randomly allocated to subcutaneously administered placebo (5% dextrose) or 1.5 x 106 IU IL-2 in a 1:1 ratio using an independent, web-based application (www.sealedenvelope.com). Randomization was stratified by ST segment elevation status.
Blinding	Participants were blinded to treatment allocation. All investigators carrying out study visits, data collection and analysis were blinded to treatment allocation for study participants. Those analysing the PETCT imaging were also additionally blinded to patient details and scan date.

Reporting for specific materials, systems and methods

We require information from authors about some types of materials, experimental systems and methods used in many studies. Here, indicate whether each material, system or method listed is relevant to your study. If you are not sure if a list item applies to your research, read the appropriate section before selecting a response.

Materials & experimental systems

n/a	Involved in the study	
☐	☒	Antibodies
☒	☐	Eukaryotic cell lines
☒	☐	Palaeontology and archaeology
☒	☐	Animals and other organisms
☐	☒	Clinical data
☒	☐	Dual use research of concern

Methods

n/a	Involved in the study	
☒	☐	ChIP-seq
☐	☒	Flow cytometry
☒	☐	MRI-based neuroimaging

Antibodies

Antibodies used

(BD) CD196 PE 20µl 551773 Clone: 11A9 BD Pharmingen
(BD) CD25 BB515 5µl 564467 Clone: 2A3 BD Horizon
(BD) CD194 BB700 5µl 566475 Clone: 1G1 BD Pharmingen
(BD) CD197 Pe-Cy7 (RAT) 5µl 557648 Clone: 3D12 BD Pharmingen
(BD) CD185 AF647 (RAT) 5µl 558113 Clone: RF8B2 BD Pharmingen
(BD) CD4 AF700 5µl 557922 Clone: RPA-T4 BD Pharmingen
(BD) CD45RA APC-H7 5µl 560674 Clone: HI100 BD Pharmingen
(BD) CD183 BV421 5µl 562558 Clone: 1C6/CXCR3 BD Horizon
(BD) CD3 BV510 5µl 563109 Clone: UCHT1 BD Horizon
(BD) CD127 BV605 5µl 562662 Clone: HIL-7R-M21 BD Horizon
(BD) CD8 BV711 5µl 563677 Clone:RPA-T8 BD Horizon
(BD) CD279 BV786 5µl 563789 Clone: EH12.1 BD Horizon

Validation

The antibodies are validated using BD compensation beads (mouse and Rat). The BD FACS Lyric has automatic compensation software set by CST beads and then BD Compensation beads incubated with each antibody/flurochrome. Manual adjustments can be made if required as reference settings are unique to this panel (ie not the pre set Lyse wash settings). Reference settings (compensation) is updated every 60 days.

Clinical data

Policy information about clinical studies

All manuscripts should comply with the ICMJE guidelines for publication of clinical research and a completed CONSORT checklist must be included with all submissions.

Clinical trial registration

ClinicalTrials.gov number, IVORY- NCT04241601; IVORY-FINALE NCT06427694

Study protocol

The first and last versions of the protocol for IVORY, IVORY-FINALE and the statistical analysis plan (SAP) have been submitted with the manuscript in the supplementary information file

Data collection

IVORY was conducted across two sites in the United Kingdom, Cambridge University Hospitals and Royal Papworth Hospital. Patients were recruited to the IVORY trial between August 2020 and November 2022; the last trial visit was in January 2023. Data collection for the IVORY trial ceased at the last visit of the last participant. IVORY FINALE study is ongoing and interim results are presented for this study.

Outcomes

These are defined in the protocol and statistical analysis plan.The primary outcome of the trial was the absolute difference in mean of the maximum target to background ratio (TBRmax) of the index vessel at the end of treatment between the low-dose IL-2 and placebo group. This analysis included all segments of the index vessel. The index vessel was defined as the most inflamed vessel (the vessel with the highest mean TBRmax) on the pre-treatment 18F-FDG PET/CT scan.

The secondary outcomes included the difference in mean TBRmax for more inflamed areas (active segments/most disease segments). Additional secondary outcomes included changes in circulating Treg cells, T effector (eff) cells and safety/tolerability of treatment. The exploratory outcomes included analysis of the additive treatment effect of low-dose IL-2 over placebo for various baseline levels of inflammation, cardiac biomarkers (e.g. troponin, hsCRP) and left ventricular ejection fraction.

In IVORY-FINALE, the primary outcome was the difference in composite of MACE including cardiovascular death, non-fatal myocardial infarction, resuscitated cardiac arrest, ischemic stroke, or unplanned coronary revascularization; between low-dose IL-2 and placebo.

Flow Cytometry

Plots

Confirm that:

- [x] The axis labels state the marker and fluorochrome used (e.g. CD4-FITC).
- [x] The axis scales are clearly visible. Include numbers along axes only for bottom left plot of group (a 'group' is an analysis of identical markers).
- [x] All plots are contour plots with outliers or pseudocolor plots.
- [x] A numerical value for number of cells or percentage (with statistics) is provided.

Methodology

Sample preparation	Biological source of samples are EDTA blood samples from IVORY trial participants, analysed within 4 hours of collection. The volume of each antibody in the table as per manufacturer instructions (20 or 5 ul) are added to 100ul of blood. They are incubated in the dark for 15mins and then 2ml of 1:10 BD FACS lyse is added to remove the red cells. They are then spun at 1200rpm for 5 mins and the supernatant decanted , 2ml of BD cell wash is then added and spun again for 5 mins at 1200 rpm. Again the supernatant is decanted and 0.5ml of BD FACS flow added (saline solution) to run on the cytometer.
Instrument	BD (Becton Dickinson) 12 colour FACS Lyric
Software	Not applicable.
Cell population abundance	Not applicable.
Gating strategy	Described in Extended data figure 5.

- [x] Tick this box to confirm that a figure exemplifying the gating strategy is provided in the Supplementary Information.
